# Expanding the Repertoire of Low‐Molecular‐Weight Pentafluorosulfanyl‐Substituted Scaffolds

**DOI:** 10.1002/cmdc.202100641

**Published:** 2022-02-22

**Authors:** Arathy Jose, Daniel Guest, Remi LeGay, Graham J. Tizzard, Simon J. Coles, Mariliza Derveni, Edward Wright, Lester Marrison, Alpha A. Lee, Aaron Morris, Matt Robinson, Frank von Delft, Daren Fearon, Lizbé Koekemoer, Tetiana Matviuk, Anthony Aimon, Christopher J. Schofield, Tika R. Malla, Nir London, Barnaby W. Greenland, Mark C. Bagley, John Spencer, The Covid Moonshot Consortium

**Affiliations:** ^1^ Chemistry Department School of Life Sciences, Falmer Brighton BN1 9QJ UK; ^2^ Normandie Université Laboratoire de Chimie Moléculaire et Thioorganique LCMT UMR 6507 ENSICAEN, UNICAEN, CNRS 6 Bd. Du Marechal Juin, 14050 Caen France; ^3^ National Crystallography Service, School of Chemistry University of Southampton Southampton SO17 1BJ UK; ^4^ Biochemistry School of Life Sciences, Falmer Brighton BN1 9QG UK; ^5^ eMolecules, 3430 Carmel Mountain Road, Suite 250 San Diego CA 92121 USA; ^6^ PostEra Inc., 2 Embarcadero Centre San Franciso CA 94111 USA; ^7^ Diamond Light Source (DLS) Harwell Science and Innovation Campus Didcot OX11 0DE UK; ^8^ Centre of Medicines Discovery (CMD) University of Oxford Department of Biochemistry Oxford OX1 3QU UK; ^9^ Department of Biochemistry University of Johannesburg Auckland Park 2006 South Africa; ^10^ Enamine Chervonotkatska St, 67 Kyiv 02094 Ukraine; ^11^ Chemistry Research Laboratory The Department of Chemistry and the Ineos Oxford Institute for Antimicrobial Research, 12 Mansfield Road OX1 3TA Oxford UK; ^12^ Department of Chemical and Structural Biology Weizmann Institute of Science Rehovot 76100 Israel; ^13^ Members list: https://tinyurl.com/y3r7redd

**Keywords:** SF_5_ group, DMARDs, COVID-19, SARS-COV-2 main protease (Mpro)

## Abstract

The pentafluorosulfanyl (‐SF_5_) functional group is of increasing interest as a bioisostere in medicinal chemistry. A library of SF_5_‐containing compounds, including amide, isoxazole, and oxindole derivatives, was synthesised using a range of solution‐based and solventless methods, including microwave and ball‐mill techniques. The library was tested against targets including human dihydroorotate dehydrogenase (HDHODH). A subsequent focused approach led to synthesis of analogues of the clinically used disease modifying anti‐rheumatic drugs (DMARDs), Teriflunomide and Leflunomide, considered for potential COVID‐19 use, where SF_5_ bioisostere deployment led to improved inhibition of HDHODH compared with the parent drugs. The results demonstrate the utility of the SF_5_ group in medicinal chemistry.

## Graphical abstract

**A range of molecules** containing a pentafluorosulfanyl group have been made and tested versus known drug‐like entities. The SF_5_ functional group is of increasing interest as a bioisostere in medicinal chemistry. This library was tested against targets including human dihydroorotate dehydrogenase (HDHODH). A subsequent focused approach led to analogues of Teriflunomide and Leflunomide, considered for potential COVID‐19 treatment, where SF_5_ bioisostere deployment led to improved inhibition of HDHODH over the parent drugs.

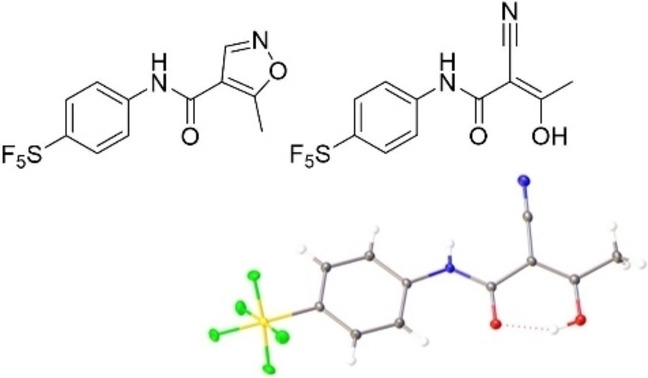

## Introduction

**Figure 1:**
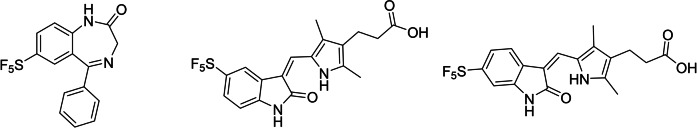
SF_5_‐benzodiazepine and oxindole analogues previously made in our group.

Small‐molecule organic compounds are often used as tool compounds and chemical probes for functional validation in biological systems and as therapeutics.^[1]^ Halogen containing fragments and higher molecular weight derivatives form a large proportion of drug‐like molecules.[^2^, ^3^, ^4^, ^5^] The pentafluorosulfanyl group is gaining popularity as a bioisostere in bioactive compounds[^6^, ^7^] and in materials^[8]^ as it is considered to be relatively stable, electronegative and lipophilic alternative to a CF_3_ group. Recent years have seen a rise in SF_5_‐substituted compounds as direct access to aryl‐ and alkyl‐SF_5_ building blocks has been achieved.[^9^, ^10^] We have recently incorporated the SF_5_ group in benzodiazepine and oxindole analogues (Figure 1).[^11^, ^12^] In the former example, significant activity was lost, likely due to the steric size of the SF_5_ group compared to a Cl substituent.

Owing to the relative dearth of bioactive pentafluorosulfanyl containing compounds, yet commercial availability of a number of attractive building blocks, we set out to synthesise libraries of SF_5_‐phenyl derivatives endowed with further functionality. This work was intended to serve two purposes: the synthesis of novel small libraries to show synthetic scope and possible interest for screening programmes and the directed synthesis of SF_5_‐analogues as a comparison with known, electron withdrawing, CF_3_, Cl− and NO_2_‐substituted bioactive molecules.

## Results and Discussion

**Scheme 1:**
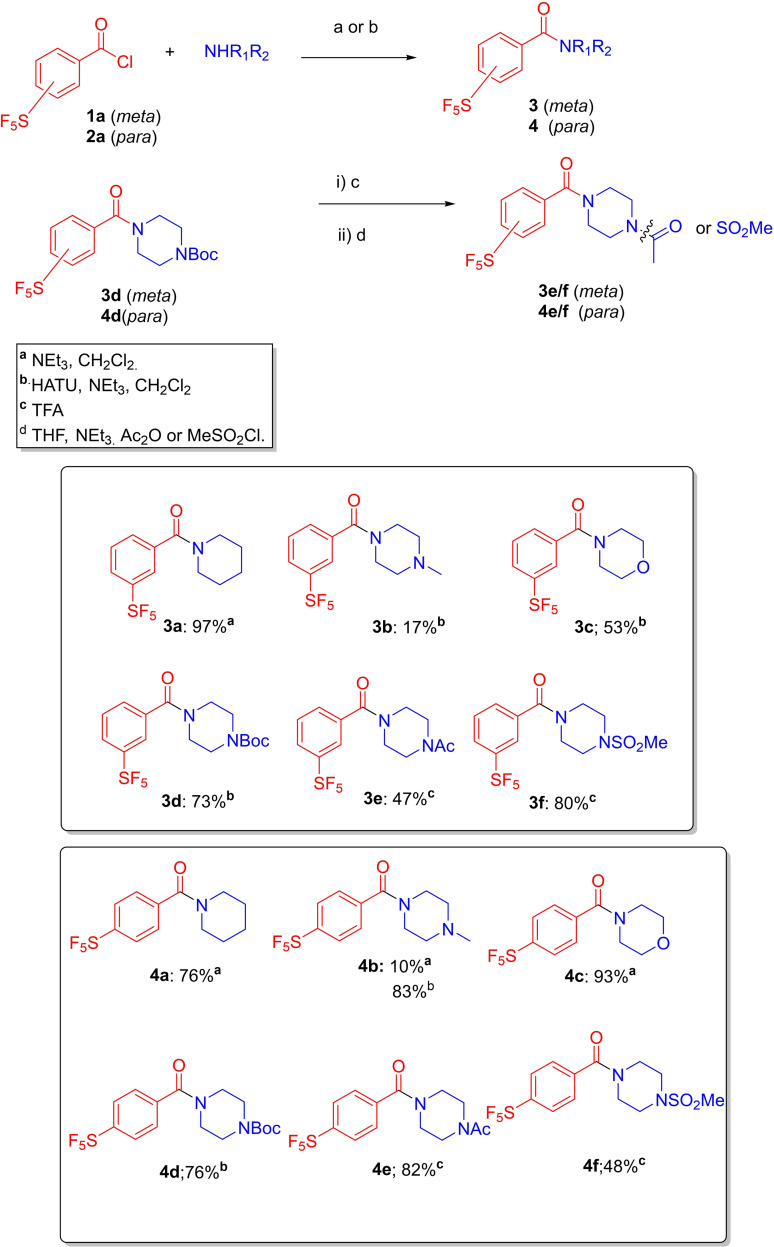
Synthesis of a small SF_5_‐subsituted amide library.

Initially, SF_5_‐containing small molecules were constructed via a simple amide bond‐forming reaction, using trimethylamine in dichloromethane (conditions “a”); in general, were satisfactory (Scheme 1). Purifications varied according to the reaction, but typically involved flash silica chromatography or the use of the nucleophilic scavenger, MP‐*Trisamine* (macroporous polystyrene‐bound nucleophilic scavenger), to remove unreacted acid or acid chloride. Attempted reactions with 1‐methylpiperazine led to poor yields, e. g., **3** **b**, and the free benzoic acid was detected in the crude reaction mixture, suggesting competing acid chloride hydrolysis and poor yields for the amide coupling. Hence, the coupling reagent, HATU (hexafluorophosphate azabenzotriazole tetramethyl uronium), was added (conditions “b”) to mitigate for any benzoic acid formed *in* *situ*; this led to mainly improved yields, e. g. **4** **b** (83 % vs 10 %). The *Boc*‐piperazine analogues **3** **d** and **4** **d** were successfully deprotected and further functionalised as their amide and sulphonamide derivatives **3** **e**, **4** **e** and **3** **f**, **4** **f** respectively. In total, a dozen new SF_5_‐containing amide analogues were made, which may have useful applications as halogen‐rich screening library compounds, for example.^[13]^, 1

**Scheme 2:**
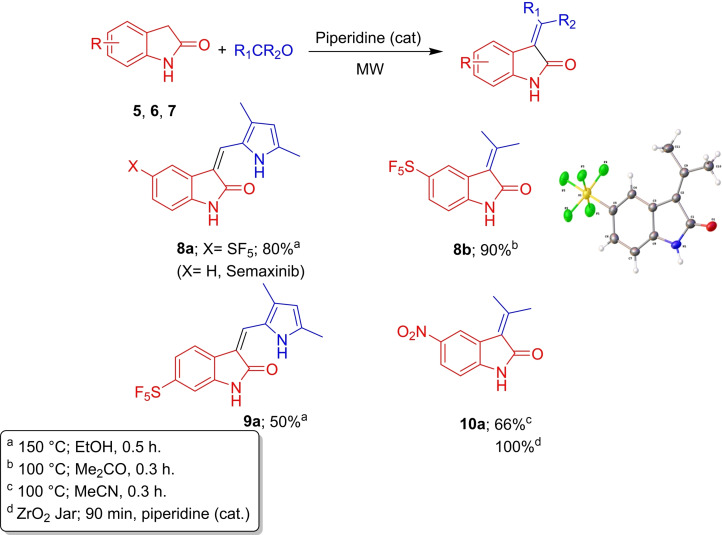
Oxindole synthesis via a Knoevenagel condensation.

Next, we focussed our attention on to oxindole derivatives, having recently reported SF_5_‐containing analogues with kinase inhibitory properties. Microwave heating was employed as in our previous work^[12]^ and, for acetone‐derived products, this acted both as a solvent and a reagent. In the synthesis of **8** **b**, soon after combining the oxindole, acetone and piperidine, product formation was observed, which precipitated out of solution. Nevertheless, the reaction mixture was heated under microwave irradiation until the reaction was complete and pure product was obtained, in 90 % yield. Crystallisation from acetone afforded single crystals of **8** **b** suitable for X‐ray structure determination (Scheme 2). In essence, these Knoevenagel condensations utilized a variety of solvents and nitro analogue, **10** **a**, was prepared to demonstrate the applicability of this reaction to other electron‐withdrawing systems. (Note; to avoid overpressure, ensure the microwave tube is less than half‐full).

Mechanochemical synthesis is an attractive method that is an environmentally friendly alternative to traditional routes^[14]^ which has been applied to Knoevenagel condensations.[^15^, ^16^] We attempted the synthesis of **10** **a** in a vibratory ball mill (VBM) in steel jars (SST) and zirconium oxide jars (ZrO_2_); with and without catalyst, and for various reaction times. Zirconia jars were best suited for mechanochemical condensation as opposed to steel jars. ^[17][18]^ Reactions performed in the absence of catalyst did not yield any product.

**Figure 2:**
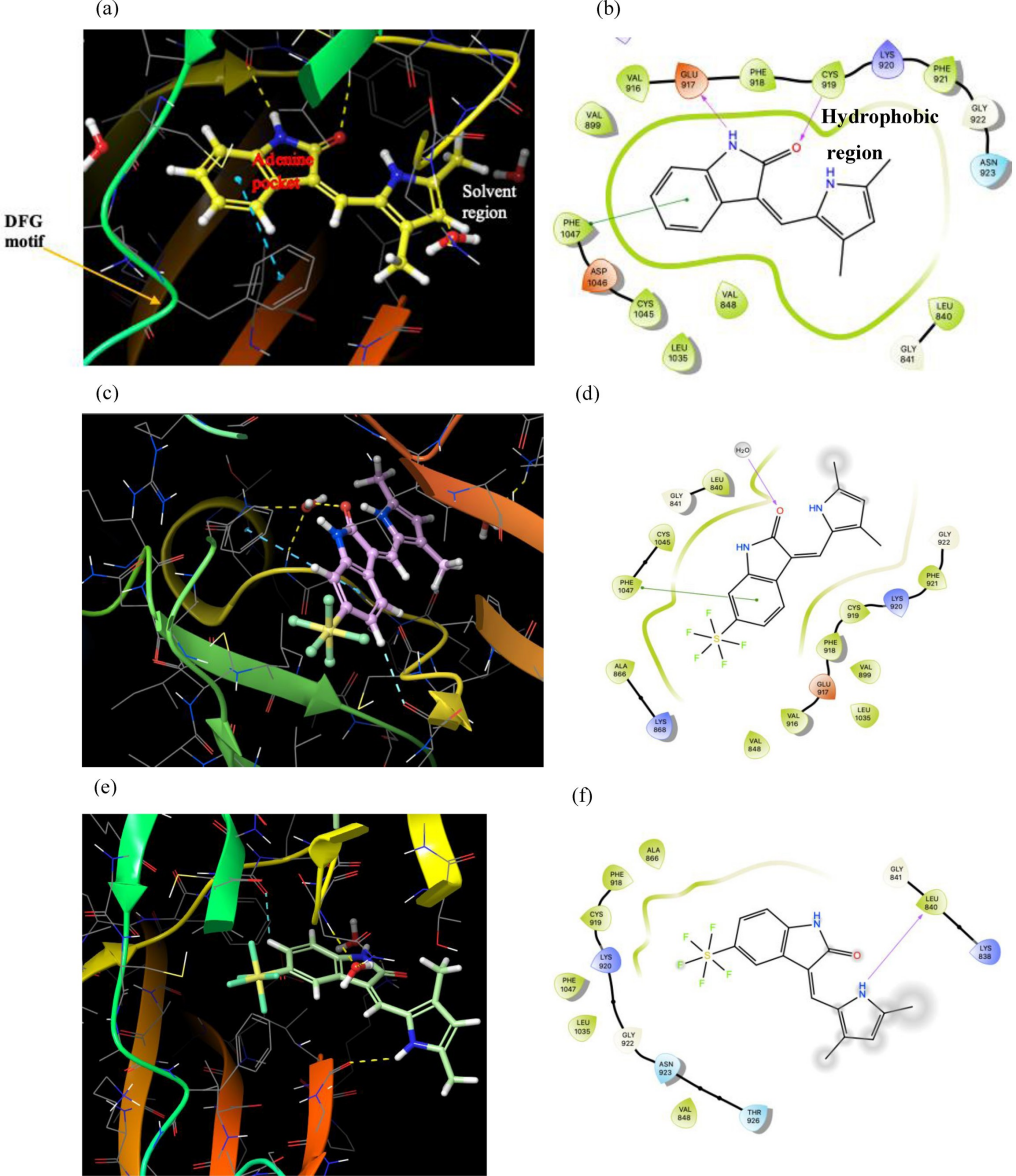
a,b) Docking studies on VEGFR2. (Schrodinger Maestro) show Semaxinib (yellow) in complex with VEGFR2, adenine pocket, hydrophobic pocket and solvent region; c,d) **9** **a** (pink) in complex with VEGFR2; e) **8** **a** (green) in complex with VEGFR2; f) lost π–π interactions due to displaced pose. Blue dashed lines indicate aromatic π–π interactions with the DFG motif and hydrogen bonds to Tyr phenol Trp. Hydrogen bonds are shown as yellow dashes.

We compared **8** **a** and **9** **a** with Semaxinib (Scheme 2), a selective inhibitor of VEGFR^[19]^, using both molecular docking and in vitro analysis in vascular endothelial growth factor receptor‐2 VEGFR2 (Figure 2). Semaxinib inhibits VEGFR2 by binding in the ATP binding site, where the pyrrole ring occupies a hydrophobic pocket and forms van der Waals interactions. The oxindole ring binds in a similar fashion to the ATP's purine and provides a hydrogen bond donor and an acceptor to form hydrogen bond interactions. The bulk of the SF_5_ group seemingly pushes the oxindole ring into the hydrophobic pocket and the pyrrole into the solvent region, thus the oxindole is no longer able to occupy the adenine pocket. **9** **a** retains a key aromatic π–π interaction, between Phe1047 (DFG motif) and the oxindole group. However, **8** **a** does not form an interaction between the oxindole ring and the adenine pocket except for a hydrogen bond to an aromatic hydrogen. The SF_5_ moiety is likely to form van der Waals interactions with Lys868 and Ala866 in the hydrophobic binding pocket. A biochemical assay showed Semaxinib to inhibit VEGFR2 with an IC_50_ of 1.5 μM (lit. 1.04±0.53 μM).^[20]^ Compounds **8** **a** and **9** **a** did not manifest sufficient inhibition to generate IC_50_ values. At 10 μM, the former exhibited 27 % inhibition of VEGFR2 while **9** **a** gave 23 % inhibition at the same concentration (Table 1).

**Table 1:** Biochemical kinase assays of semaxanib and its SF_5_ counterparts.

Entry	Compound	IC_50_ [μM]^[a]^
1	semaxanib	1.5
2	**8** **a**	_—_^[b]^
3	**9** **a**	_—_^[b]^

[a] *n*=1; 10‐dose IC_50_ mode with twofold serial dilutions, starting at 10 μM. [b] Percent inhibition at 10 μM; **8** **a** (27 %), **9** **a** (23 %).

**Scheme 3:**
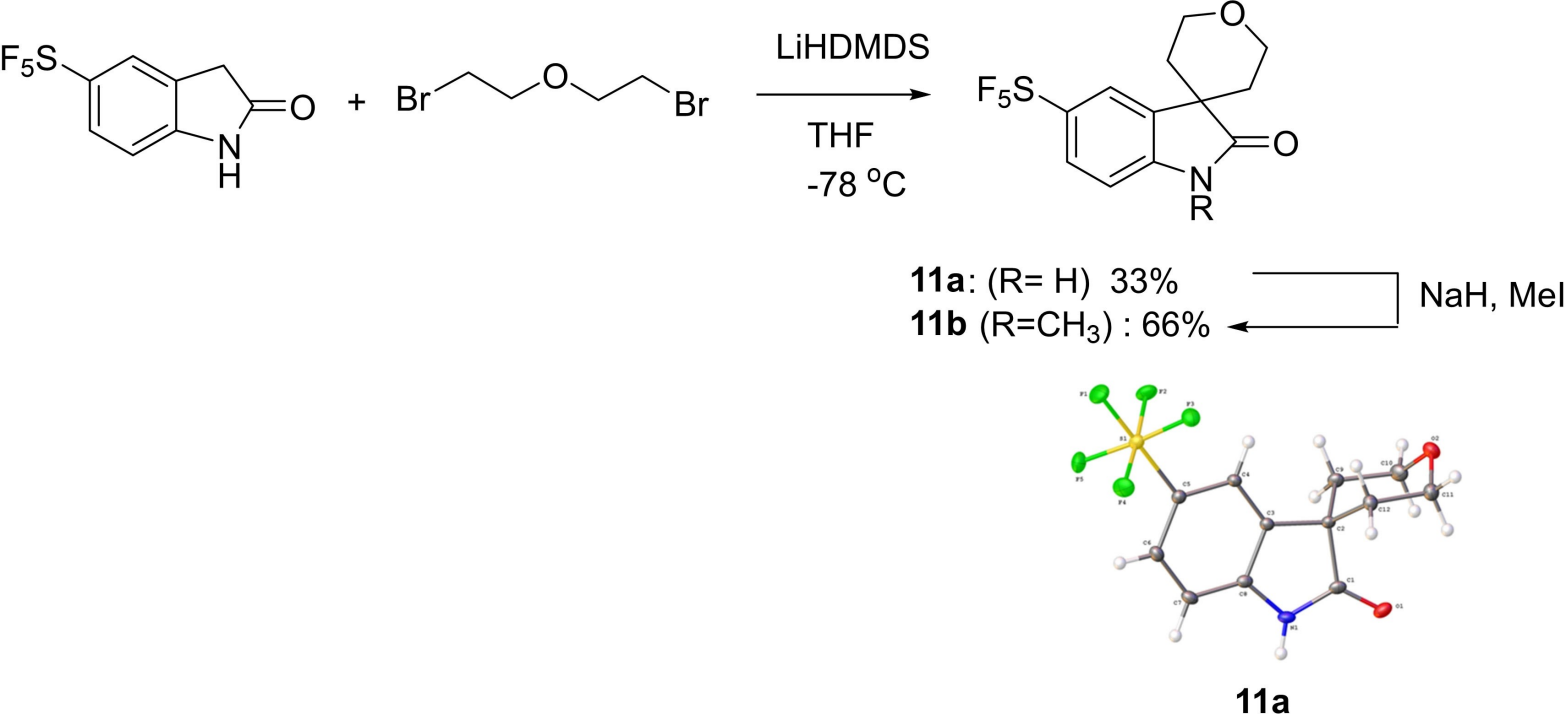
Spirocycle formation.

A spirocyclisation reaction led to the product **11** **a** in poor yield, whose structure was determined by X‐ray crystal analysis. **11** **a** was then methylated to give **11** **b** (Scheme 3). Such high Csp^3^ content compounds may offer more diversity to compound libraries and improved physiochemical properties.

### Biological studies

**Figure 3:**
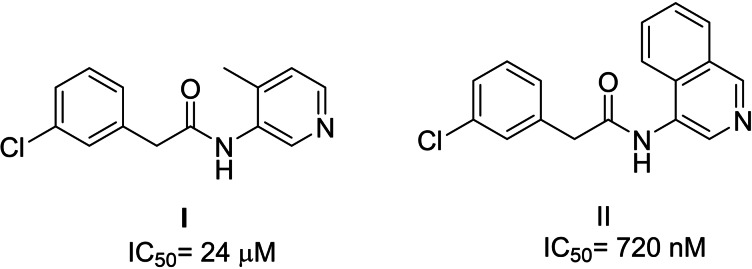
M^Pro^ inhibitors based on 3‐chlorophenylacetamides.

**Scheme 4:**
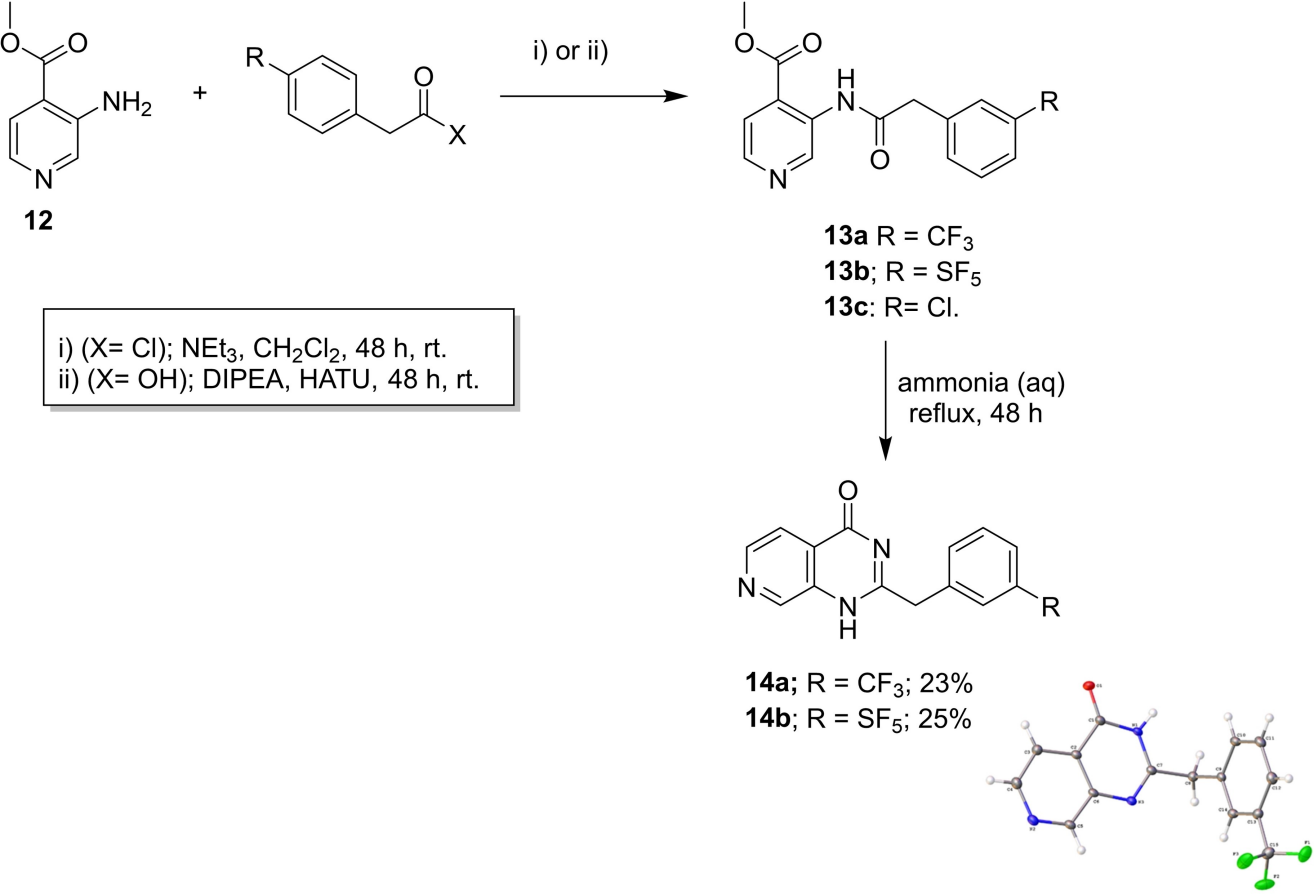
Synthesis of a small‐molecule library with CF_3_/SF_5_ groups.

A recently initiated open source effort, termed the Covid Moonshot Consortium, has led to the design of nM‐potent inhibitors of M^Pro^ (or CLPro), a vital enzyme in SARS‐ CoV‐2 replication and transcription.^[21]^ M^pro^ releases the functional polypeptides from the polyproteins by extensive proteolytic processing while digesting the polyproteins at 11 cleavage sites, starting with autolytic cleavage^[22]^ and exclusively cleaves polypeptides after a glutamine residue. Its functional importance and the absence of closely related homologues in humans, renders M^pro^ an attractive target for antiviral drug design as an example of a direct acting antiviral agent.^[23]^ Pyridine substituted 3‐Cl‐phenylacetamide analogues **I** and **II** were found to be moderate to active analogues and we wished to explore both alternatives to the Cl substituent and more water solubilising groups such as CO_2_H as opposed to fused aryl or methyl groups. (Figure 3 and Scheme 4).

The aminopyridine **12** was coupled with acid chlorides or an acid to yield the ester analogues **13**, substituted with CF_3_, SF_5_, and Cl groups (Scheme 4). Surprisingly, reaction of the latter with ammonia led to the cyclised compounds **14**, one of which, **14** **a**, was crystallised, by a diffusion method, using hexane and dichloromethane, to obtain clear crystals which enabled confirmation of its structure by X‐ray analysis.^[24]^ The reaction likely involves the formation of a carboxamide, from the methyl ester, which cyclises onto the amide carbonyl group, followed by water elimination.

Compounds **13–14** were tested for inhibition versus M^Pro^, but none had any appreciable enzyme activity (IC_50_>99 μM).

**Figure 4:**
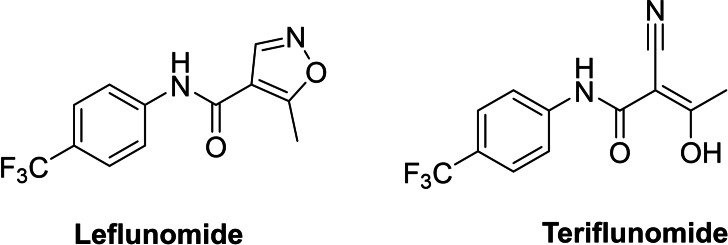
Examples of CF_3_‐containing DMARDs.

**Figure 5:**
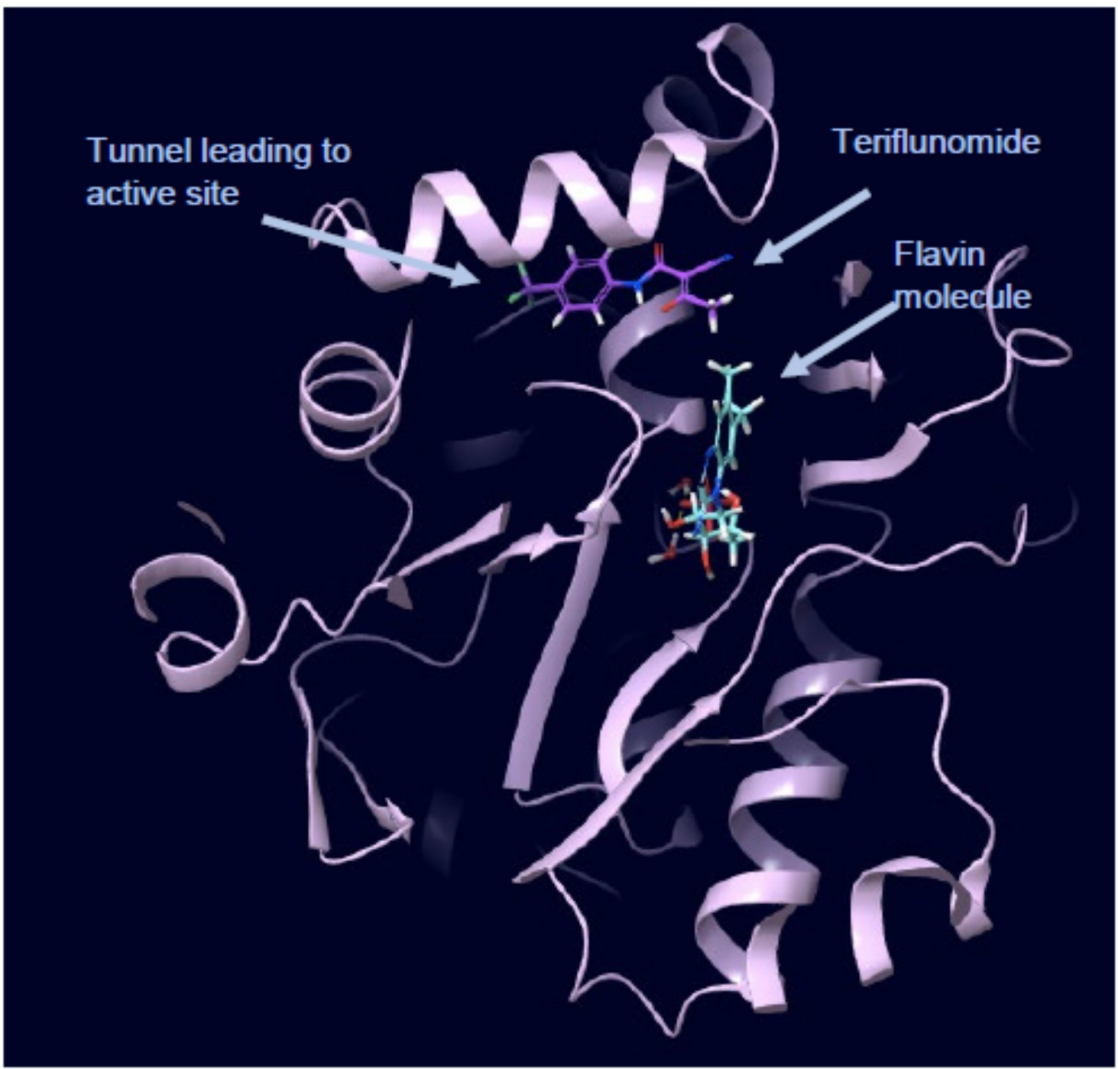
View from a crystal structure of Teriflunomide and FMN in complex with HDHODH. PDB ID: 1D3H. FMN: teal, Teriflunomide: purple.

Finally, we focused our efforts on Leflunomide (Arava®), a DMARD (disease modifying antirheumatic drug) with potential COVID‐19 use^[25]^ and its active metabolite Teriflunomide (Aubagio®), which is used for multiple sclerosis, (Figure 4). Teriflunomide inhibits human DHODH in the low μM range and binds in the same region as ubiquinone, a redox cofactor. Both Teriflunomide and ubiquinone occupy a narrow cleft near a flavin molecule (another redox cofactor), which leads to the active site (Figure 5).^[26]^ As Teriflunomide competes with ubiquinone,^[27]^ it is regarded as a redox silent coenzyme Q antagonist of DHODH.^[28]^ Teriflunomide has a polar head consisting of one hydrogen bond donor; an enol and two hydrogen bond acceptors, a nitrile and a carbonyl group, while the tail of Teriflunomide, which occupies the entrance of the tunnel, is a hydrophobic CF_3_‐substituted aromatic group.

**Scheme 5:**
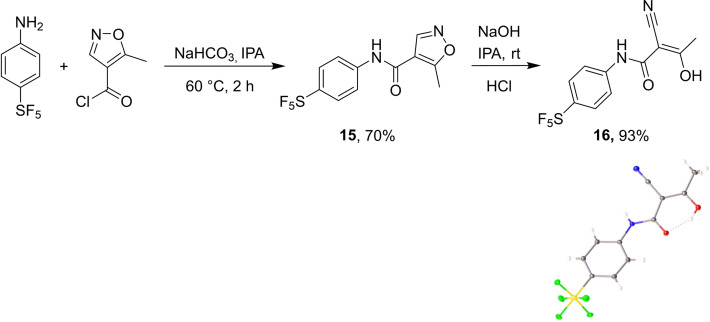
Synthesis of potential SF_5_‐containing DMARDs.

We first prepared the SF_5_ derivative of Leflunomide **15**, which, when treated with sodium hydroxide, provided its Teriflunomide equivalent **16** in good yields (Scheme 5).^[29]^ The latter was further characterised in the solid state by crystallography, proving its molecular structure and regiochemistry.

**Figure 6:**
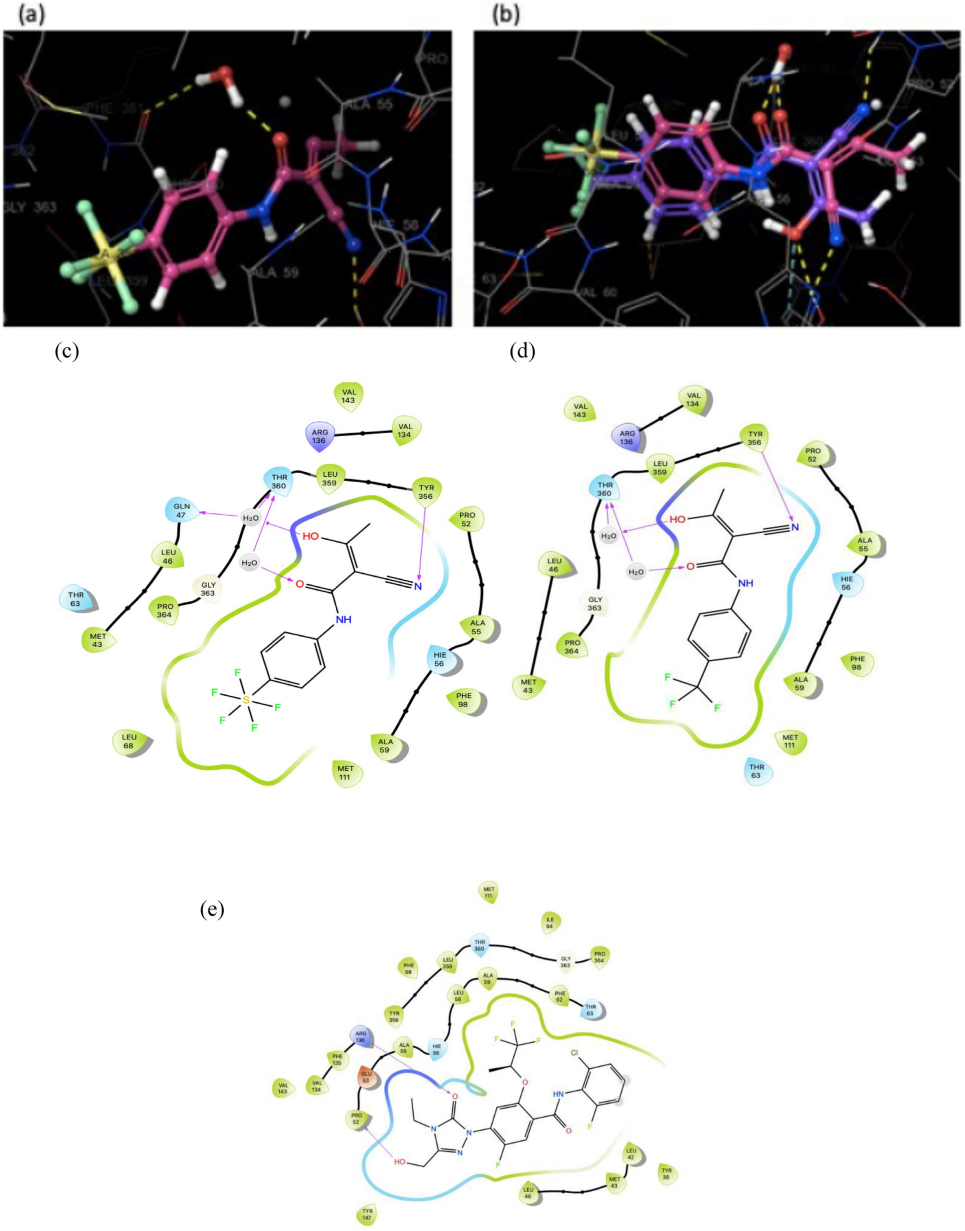
a) SF_5_‐substituted Teriflunomide in complex with HDHODH, docked using Schrödinger Maestro. b) Teriflunomide and SF_5_‐Teriflunomide located in the HDHODH binding pocket. Purple: Teriflunomide, salmon pink: SF_5_‐Teriflunomide Ligand interaction diagram, comparing c) SF_5_‐Teriflunomide and d) Teriflunomide; showing that the bulkier SF_5_ is able to fill the binding pocket better than a CF_3_ group. e) **BAY‐2402234**, a Bayer clinical trial candidate that inhibits HDHODH with an IC_50_ value of 1.2 nM.

**Figure 7:**
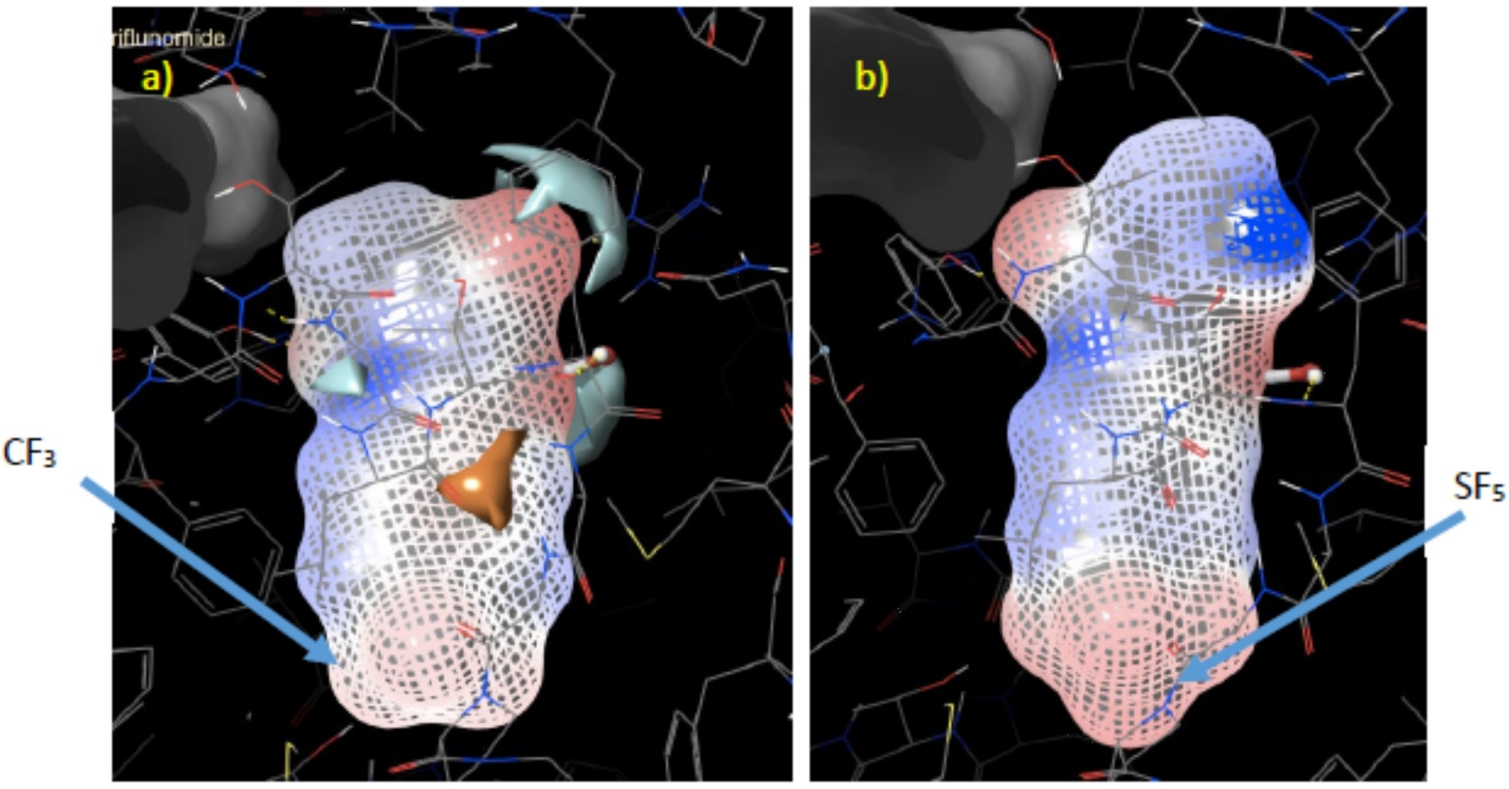
a) Electrostatic potential of Teriflunomide; b) Electrostatic potential of SF_5‐_Teriflunomide.

Docking of SF_5_‐Teriflunomide **16** in HDHODH was performed using Schrodinger Maestro. Interactions predicted between the ligand and the binding pocket are similar to those for Teriflunomide in the same binding site (Figure 6). The hydroxyl group of **16** is hydrogen bonded to a water molecule, which, in turn is bound to Gln47 and Thr360. The hydroxyl also makes a polar interaction with Arg136. The nitrile group interacts with Tyr356 via a H‐bond. Finally, the carbonyl of **16** is positioned to form a H‐bond with a water molecule that H‐bonds with Thr360. Several hydrophobic interactions are formed between the aromatic rings and amino acid residues lining the hydrophobic pocket. The electrostatic potential diagram (Figure 7) shows the small difference in the size of CF_3_ and SF_5_ groups, and that SF_5_‐Teriflunomide is slightly larger than its parent analogue.

[Following our docking studies, we tested SF_5_‐Teriflunomide and SF_5_‐Leflunomide against human HDHODH using Teriflunomide and **BAY‐2402234** (a DHODH inhibitor in phase I clinical trials) as positive controls. A comparison of IC_50_ and pIC_50_ values of SF_5_‐Teriflunomide, SF_5_‐Leflunomide, Leflunomide against Teriflunomide and **BAY‐2402234** (Table 2) was undertaken. As anticipated, SF_5_‐Leflunomide is more potent than Leflunomide. SF_5_‐Teriflunomide is approximately twice as active as Teriflunomide. The simpler analogue, **17**, tested in this assay, due to its MPro affinity (*vide infra*), was inactive. Compound, **BAY‐2402234**, gave the best potency with an IC_50_ of 1.8 nM, which is comparable to its literature value (1.2 nM).^[30]^ From similar docking, as well as the hydrophilic interactions, **BAY‐2402234** makes hydrophobic interactions with a large set of non‐polar residues such as Leu42, Met43, Leu46, etc. and, with its bigger size, and the combination of hydrophobic and hydrophilic groups, it is able to fill the HDHODH active site to a greater degree.

**Table 2:** HDHODH inhibition; comparing SF_5_‐Teriflunomide, SF_5_‐Leflunomide and Leflunomide vs. Teriflunomide and **BAY‐2402234**.

Entry	Structure	IC_50_ [nM]	pIC_50_
1	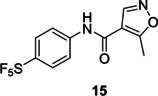	365	6.4
2	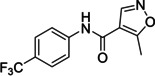	982	6.0
	Leflunomide		
3	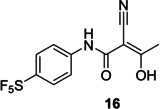	27^[a]^	7.6
4	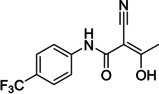	50^[b]^	7.3
	Teriflunomide		
5	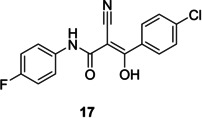	–^[c]^	
6	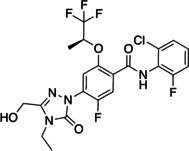	1.8^[d]^	8.7
	BAY‐2402234		

[a] Mean (*n*=2); 29 nM and 25 nM. [b] Unless stated otherwise; in vitro fluorescence‐based assays run by Reaction Biology. [c] Percent inhibition at 10 μM <10 %. [d] In vitro control (*n*=1).

**Figure 8:**
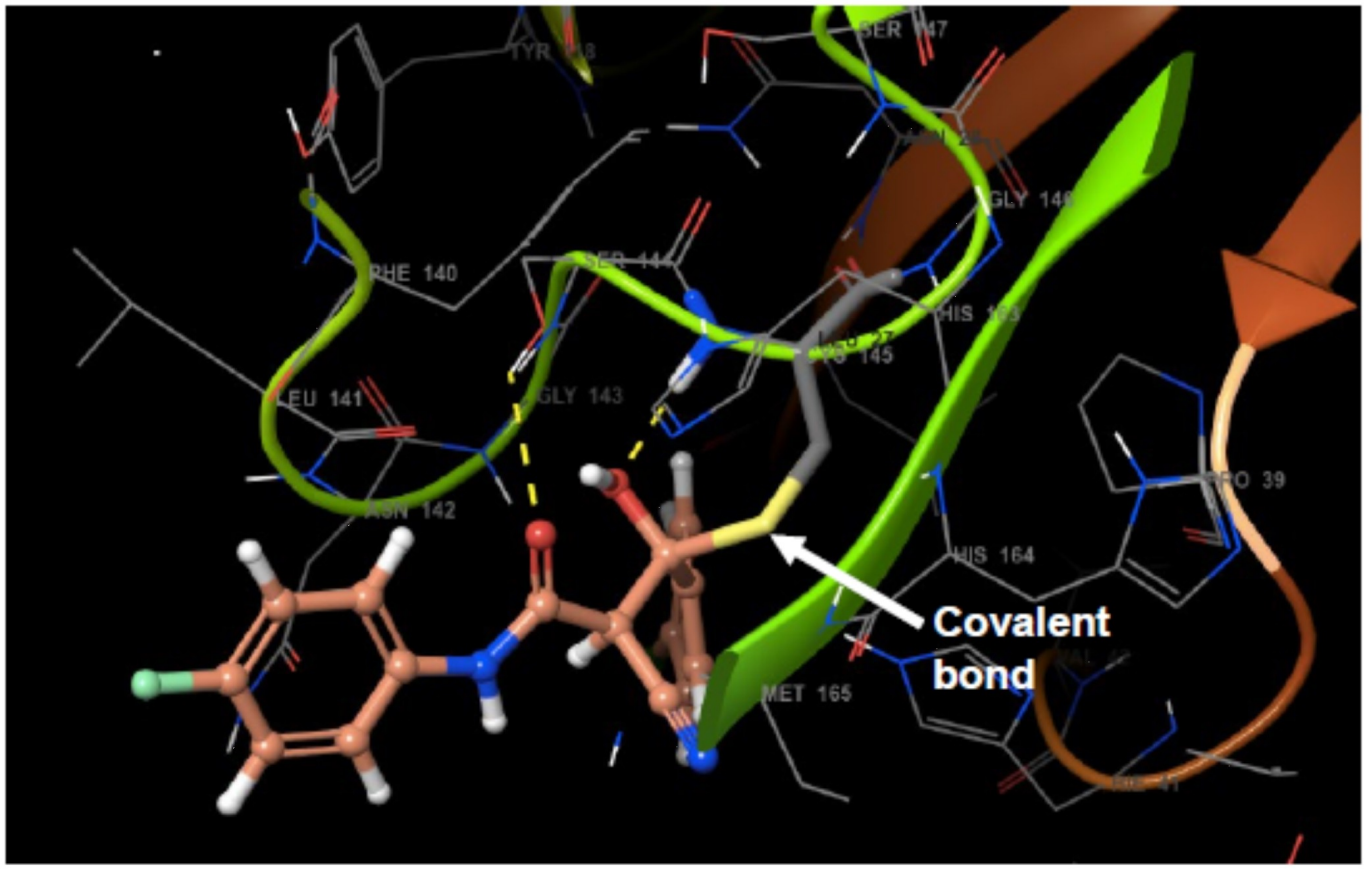
Compound **17** docked in SARS‐CoV‐2 M^pro^.

We envisioned Teriflunomide and its SF_5_ analogues such as **16** to have a covalent warhead capable of potentially acting as a Michael acceptor with the Sγ atom of Cys145 of M^Pro^, as precedented with other M^Pro^ inhibitors; other modes of covalent reaction are also possible including with the nitrile, as with an Mpro inhibitor in clinical trials,^[31]^ or the carbonyl of the keto form of **16**. In addition to Teriflunomide and **16**, fourteen other potential candidates were assayed. Out of the sixteen compounds tested, a single hit (**17**, Table 2) was identified which gave a promising IC_50_ of 0.23 μM using the fluorescence‐based turnover assay (ESI, Table S1).^[32]^ Docking studies imply ligand **17**, (Figure 8, shown in orange) could covalently react with Sγ of the nucleophilic Cys145 residue (Sγ in yellow, Figure 8), resulting in Michael addition. Besides the covalent bond, the modelling suggests formation of two hydrogen bonds (yellow dashes) are formed between the O−H of **17** and Cys145, as well as the amide carbonyl of **17** and Ser144. In addition, several hydrophobic interactions are predicted.

**Figure 9:**
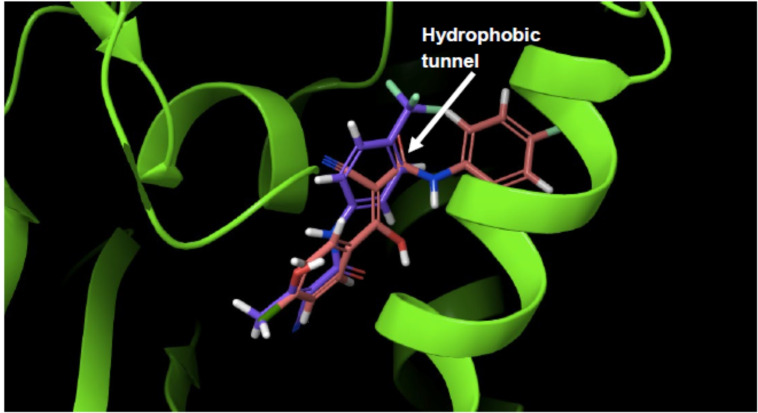
HDHODH in complex with Teriflunomide (purple) and **17** (orange).

Although **17** has structural similarities to Teriflunomide, it was found to be inactive versus HDHODH (Table 2). Docking in HDHODH suggests that **17** occupies the binding pocket differently to Teriflunomide (Figure 9). Evidently, the hydrogen bond donors and acceptors on **17** lie in a hydrophobic tunnel of HDHODH.

The outer surface of coronaviruses has a spike (S) protein which enables its entry into host cells. The S1 subunit of S protein attaches to ACE2 receptor, which is found on the surface of target cells. In addition to this, the transmembrane protease, serine 2 (TMPRSS2) processes S protein into its constituent subunits, S1 and S2, thus allowing the virus to fuse into the plasma membrane of the host cell. As HDHODH is a druggable target against SARS‐CoV‐2, we were interested in probing the inhibitory effect of Teriflunomide and compounds **16** and **17** against SARS‐CoV‐2. Teriflunomide was the most cytostatic with effects at concentrations above 0.78 μM. **16** had cytostatic effects at concentrations >3.1 μM and DMSO had no measurable effect. An infection inhibition assay showed that none of the compounds had an inhibitory effect against SARS‐CoV‐2 infection in the infected cells. The normalised data show that Teriflunomide and **16** have no effect on the efficiency of infection (Fig S2).

## Conclusion

SF_5_‐analogues of Leflunomide and Teriflunomide, **15** and **16** respectively, have been synthesised and tested for affinity towards HDHODH. Biophysical assays revealed an approximately two‐fold greater affinity for SF_5_‐Teriflunomide (**15**) towards HDHODH compared with Teriflunomide. Molecular binding studies revealed that the bulky SF_5_ group fills the binding pocket better than the CF_3_ group. A singleton hit **17** with structural similarities to Teriflunomide was identified as inhibiting SARS‐CoV‐2 M^pro^ the low micromolar IC_50_ range whereas a commercial library of similar analogues as well as an in‐house library showed no affinity. Testing **17** against HDHODH, however, revealed no affinity. Neither Teriflunomide nor compounds **15** and **16** gave satisfactory inhibition of SARS‐CoV‐2 infection. Indeed, they were found to be cytostatic in HEK293T/17 cells. Nevertheless, the SF_5_ analogue of Teriflunomide, **16**, might be a useful DHODH probe molecule for future investigation.

## Experimental Section

4‐(Pentafluorosulfanyl)aniline was obtained from Fluorochem and 5‐methyl‐4‐isoxazolecarbonyl chloride was purchased from Apollo Scientific. Magnesium sulphate and sodium bicarbonate were obtained from Fisher Scientific. Preparative TLC plates were obtained from Analtech. Solvents and reagents were purchased from commercial suppliers and were used without purification. All reactions were performed in a fume hood. NMR spectra were recorded on Varian 500 MHz or 400 MHz spectrometers and chemical shifts are reported in ppm, usually referenced to TMS as an internal standard. LCMS measurements were performed on a Shimadzu LCMS‐2020 equipped with a Gemini® 5 μm C18 110 Å column and percentage purity measurements were run over 30 minutes in water/acetonitrile with 0.1 % formic acid (5 min at 5 %, 5 %–95 % over 20 min, 5 min at 95 %) with the UV detector at 254 nm. Mass spectrometry: ESI mass spectra were obtained using a Bruker Daltonics Apex III, using Apollo ESI as the ESI source. For EI mass spectra, a Fissions VG Autospec instrument was used at 70 eV. All analyses were run by Dr. Alla K. Abdul Sada. Analyses are for the molecular ion peak [M]^+^ and are given in m/z, mass to charge ratio. Melting points were determined using a Stanford Research Systems Optimelt and are uncorrected.

### [3‐(Pentafluoro‐λ^6^‐sulfanyl)phenyl](piperidin‐1‐yl)methanone (3 a)

Triethylamine (247.0 mg, 2.44 mmol), was added dropwise to piperidine (175.8 mg, 2.07 mmol) dissolved in dichloromethane (2 mL) followed by the dropwise addition of 3‐(pentafluorosulfanyl)benzoyl chloride (500.0 mg, 1.88 mmol). The reaction mixture was stirred at rt overnight. It was then diluted with dichloromethane (5 mL) and washed with 2 M HCl (10 mL×3). The aqueous layers were extracted with dichloromethane (15 mL×3), dried over MgSO_4_, filtered and concentrated in *vacuo*. The crude was purified over a column of silica (dichloromethane:methanol, 9 : 1) to give **3** **a** as a colourless solid (572.0 mg, 97 %).^1^H NMR (600 MHz, CDCl_3_) δ 7.72 (m, 2H, ArH), 7.47 (m, 2H, ArH), 3.46 (m, 4H, 2CH_2_), 1.63 (m, 4H, 2CH_2_), 1.47 (m, 2H, CH_2_). ^13^C NMR (151 MHz, CDCl_3_) δ 167.9 (C=O), 153.7 (C‐SF_5_, p, ^2^ *J*_F,C_=17.5 Hz), 137.3 (ArC), 129.8 (ArC), 128.9 (ArC), 126.6 (ArC‐CSF_5_, m), 124.6 (ArC‐CSF_5_, m), 48.6 (N‐CH_2_), 43.2 (N‐CH_2_), 26.3 (2CH_2_), 25.4 (CH_2_); ^19^F NMR (400 MHz, CDCl_3_) δ 83.60 (p, *J*=150.0 Hz), 62.44 (d, *J*=150.0 Hz); LCMS Purity (UV)=96 %, *t*_R_ 21.0 min; HRMS‐ ESI (*m/z*) found 316.0785, calc. for [C_12_H_14_F_5_NOS][H]^+^: 316.0789; IR (neat) ν_max_/cm^−1^: 3680, 1633, 820; mp 88–90 °C.

### (3‐(Pentafluoro‐λ^6^‐sulfanyl)phenyl](4‐methylpiperazin‐1‐ yl)methanone (3 b)

To *N*‐methylpiperazine (207.0 mg, 2.07 mmol), dissolved in dichloromethane (2 mL) was added triethylamine (247.4 mg, 2.44 mmol) together with HATU (787.0 mg, 2.07 mmol) and, finally, 3‐(pentafluorosulfanyl)benzoyl chloride (500.0 mg, 1.88 mmol) was added dropwise. The reaction mixture was stirred at room temperature overnight, then filtered and the filtrate was stirred with MP Trisamine (3 equiv.) for 3 hours to remove the unreacted acid chloride. The crude product was purified over a column of silica (dichloromethane:methanol; 9 : 1) to give **3** **b** as a colourless gum (107 mg, 17 %).^1^H NMR (600 MHz, CDCl_3_) δ 7.79 (ArH, m, 2H), 7.53 (ArH, m, 2H), 3.80 (CH_2_, bs, 2H), 3.41 (CH_2_, bs, 2H), 2.50 (CH_2_, bs, 2H), 2.36 (CH_2_, bs, 2H), 2.32 (CH_3_, s, 3H); ^13^C NMR (151 MHz, CDCl_3_) δ 168.1 (C=O), 153.9 (ArC‐SF_5_, p, ^2^ *J*_FC_=17.8 Hz), 136.6 (ArC), 130.1 (ArC), 129.1 (ArC), 127.1 (ArC, p, ^2^ *J*_FC_= 4.4 Hz), 124.9 (ArC, p, ^2^ *J*_FC_=4.4 Hz), 55.0 (C), 54.5 (C), 47.6 (C), 45.8 (C), 42.2 (C); ^19^F NMR (400 MHz, CDCl_3_) δ 83.45 (1F, p, *J*=150.1 Hz), 62.83 (4F, d, *J*=150.1 Hz); LCMS Purity (UV)=97 %, *t*_R_ 7.1 min; HRMS‐ESI (*m/z*) found:331.0896, calc. for [C_12_H_15_F_5_N_2_OS][H]^+^:331.0898; IR (neat) ν_max_/cm^−1^ 3499 (C−H), 1614 (C=O), 823 (S−F).

### [3‐(Pentafluoro‐λ^6^‐sulfanyl)phenyl](morpholino)methanone (3 c)

To morpholine (180.30 mg, 2.07 mmol), dissolved in dichloromethane (2 mL), was added triethylamine (246.90 mg, 2.44 mmol) and the mixture was stirred for a few minutes. HATU (787.10 mg, 2.07 mmol) was added to this solution followed by dropwise addition of 3‐(pentafluorosulfanyl)benzoyl chloride (500.0 mg, 1.88 mmol). The reaction mixture was stirred at room temperature overnight then diluted with dichloromethane (5 mL) and the precipitate formed was discarded after gravity filtration. MP‐Trisamine (520.0 mg, 3.00 mmol) was added to the filtrate and stirred for 3 hours after which the scavenger was removed by gravity filtration. The crude was purified over a column of silica (dichloromethane:methanol, 9 : 1) to give **3** **c** as a colourless solid (317.0 mg, 53 %). ^1^H NMR (600 MHz, CDCl_3_) δ 7.75–7.71 (m, 2H, ArH), 7.50–7.43 (m, 2H, ArH), 3.75–3.63 (m, 4H, (CH_2_)_2_), 3.57–3.26 (m, 4H, (CH_2_)_2_); ^13^C NMR (151 MHz, CDCl_3_) δ 168.0 (C=O), 153.8 (p, *J*= 17.7 Hz, ArCSF_5_), 136.2 (ArC), 130.1(ArC), 129.1 (ArC), 127.2 (p, *J*=4.5 Hz, (ArC‐CSF_5_)), 124.9 (p, *J*=4.5 Hz, ArC‐CSF_5_), 66.6 (4 C); ^19^F NMR (400 MHz, CDCl_3_) δ 84.29–82.21 (m), 62.66 (d, *J*=150.4 Hz); LCMS Purity (UV)=97 %, *t*_R_ 18.3 min; HRMS‐ESI (*m/z*) found 340.0384, calc. for [C_11_H_12_F_5_NO_2_S][Na]^+^: 340.0401; IR (neat) ν_max_/cm^−1^ 2858, 1644, 821; mp 63–64 °C.

### tert‐Butyl 4‐(3‐pentafluoro‐λ^6^‐sulfanyl benzoyl)piperazine‐1‐ carboxylate (3 d)

To *N*‐Boc piperazine (385.5 mg, 2.07 mmol), dissolved in dichloromethane (3 mL) was added triethylamine (247.0 mg, 2.44 mmol) and the mixture was stirred for a few minutes. HATU (787.2 mg, 2.07 mmol) was added to this solution followed by dropwise addition of 3‐(pentafluorosulfanyl)benzoyl chloride (500.0 mg, 1.88 mmol) and the reaction mixture was stirred at room temperature overnight. After completion of the reaction, it was diluted with dichloromethane (10 mL) and the precipitate formed was removed by filtration. MP Trisamine (520 mg, 3 mmol) was added to the filtrate then removed by gravity filtration after 3 hours of stirring. The crude material was purified over a column of silica (dichloromethane:methanol, 9 : 1) to obtain **3** **d** as a colourless solid (573.0 mg, 73 %).

^1^H NMR (600 MHz, CDCl_3_) δ 7.81(m, 2H, ArH), 7.53 (m, 2H, ArH), 3.51 (m, 4H, (CH_2_)_2_), 3.39 (m, 4H, (CH_2_)_2_), 1.45 (s, 9H, (CH_3_)_3_); ^13^C NMR (151 MHz, CDCl_3_) δ 168.4 (C=O), 154.5 (C=O), 154.0 (ArC‐SF_5_), 136.3 (ArC), 130.1 (ArC), 129.2 (ArC), 127.4 (ArC‐CSF_5_), 125.0 (ArC‐CSF_5_), 80.5 (C), 47.5 (2 C, m), 42.9 (2 C, m), 28.3 (3 C); ^19^F NMR (400 MHz, CDCl_3_) δ 83.20 (m, F), 62.67 (d, *J*=150.1 Hz, 4F); LCMS Purity (UV)=99 %, *t*_R_ 20.4 min. HRMS‐ESI (*m/z*) found 439.1078, calc. for [C_16_H_21_F_5_N_2_O_3_S][Na]^+^: 439.1085; IR (neat) ν_max_/cm^−1^ 2977, 1685, 1626, 1243, 828; mp 153–155 °C.

### 1‐[4‐[3‐(Pentafluoro‐λ^6^‐sulfanyl)benzoyl]piperazin‐1‐yl] ethanone (3 e)

Compound **3** **d** (573 mg, 1.38 mmol) in dichloromethane (2 mL) was treated with HCl in dioxane (4 M, 5.0 equiv.) overnight. The reaction mixture was concentrated in *vacuo* to afford the HCl salt, **3** **d’** (4‐[3‐(pentafluoro‐λ^6^‐sulfanyl)benzoyl]piperazinyl hydrochloride) as a colourless solid. To crude **3** **d’** (0.100 g, 0.28 mmol), dissolved in THF (3 mL) was added triethylamine (0.028 g, 0.28 mmol) and stirred for 30 minutes. Acetic anhydride (0.028 g, 0.28 mmol) was added to the mixture and stirred overnight at room temperature. The reaction mixture was concentrated in *vacuo* and purified by flash chromatography (dichloromethane:methanol; 9 : 1) to obtain the purified **3** **e** as a colourless oil (47.0 mg, 47 %). ^1^H NMR (600 MHz, CDCl_3_) δ 7.84–7.81 (m, 1H, ArH), 7.80–7.79 (m, 1H, ArH), 7.57–7.51 (m, 2H, ArH), 3.88–3.28 (m, 8H, ArH), 2.11 (s, 3H, CH_3_); ^13^C NMR (151 MHz, CDCl_3_) δ 169.3 (C=O, MeCO), 168.4 (C=O), 154.0 (t, ^2^ *J*_FC_=18.0 Hz, ArC), 136.0 (ArC), 130.2 (ArC), 129.3 (ArC), 127.7–127.4 (m, ArC), 125.0 (ArC), 47.4 (bs, CH_2_), 45.9 (bs, CH_2_), 42.4 (bs, CH_2_), 41.4 (bs, CH_2_), 21.3 (CH_3_); ^19^F NMR (400 MHz, CDCl_3_) δ 83.19 (p, *J*=150.3 Hz, F), 62.78 (d, *J*=150.3 Hz, 4F); LCMS Purity (UV)=96 %, *t*_R_ 13.5 min; HRMS‐ESI (*m/z*) found 381.0662, calc. for [C_13_H_15_F_5_N_2_O_2_S][Na]^+^: 381.0667; IR (neat) ν_max_/cm^−1^ 2917 (C−H), 1633 (C=O), 1322 (S=O), 821 (S−F).

### (3‐Pentafluoro‐λ^6^‐sulfanyl phenyl)(4‐(methylsulfonyl)piperazin‐1‐yl)methanone (3 f)

To crude **3** **d’** (75 mg, 0.21 mmol), dissolved in THF (3 mL) was added triethylamine (21 mg, 0.21 mmol) and stirred for 30 min. Methylsulfonyl chloride (24 mg, 0.21 mmol) was added to the reaction mixture and stirred overnight at room temperature. The reaction was followed by TLC. After completion of the reaction, it was separated over dichloromethane (5 mL) and water (5 mL). The biphasic layers were passed through a hydrophobic frit to obtain the organic phase which was concentrated in *vacuo*. The crude was then purified over a column of silica, (dichloromethane:methanol; 7 : 3) to obtain **3** **f** as a colourless solid (66 mg, 80 %). ^1^H NMR (600 MHz, CDCl_3_) δ 7.86–7.82 (m, 1H, ArH), 7.82–7.80 (m, 1H, ArH), 7.57–7.54 (m, 2H, ArH), 4.03–3.47 (m, 4H, (CH_2_)_2_), 3.38–3.14 (m, 4H, (CH_2_)_2_), 2.81 (s, 3H, CH_3_); ^13^C NMR (151 MHz, CDCl_3_) δ 168.4 (C=O), 154.2–153.8 (m, C‐SF_5_), 135.7 (ArC), 130.2 (ArC), 129.4 (ArC), 127.8–127.6 (m, ArC), 125.2–125.0 (m, ArC), 47.4 (CH_2_), 45.5 (2 C, (CH_2_)_2_), 42.0 (CH_2_), 35.0 (CH_3_); ^19^F NMR (400 MHz, CDCl_3_) δ 83.32 (p, *J*=150.4 Hz, 1F), 62.96 (d, *J*=150.4 Hz, 4F); LCMS Purity (UV)=98 %, *t*_R_ 15.6 min; HRMS‐ESI (*m/z*) found 417.0349, calc. for [C_12_H_15_F_5_N_2_O_2_S][Na]^+^: 417.0342; IR (neat) ν_max_/cm^−1^ 2007 (C−H), 1616 (C=O), 1342 (S=O), 826 (S−F); mp 159–160 °C.

### [4‐(Pentafluoro‐λ^6^‐sulfanyl)phenyl](piperidin‐1‐yl)methanone (4 a)

Triethylamine (247.4 mg, 2.44 mmol) was added to piperidine (176.0 mg, 2.07 mmol) dissolved in dichloromethane (2 mL) followed by dropwise addition of 4‐(pentafluorosulfanyl) benzoyl chloride (500.0 mg, 1.88 mmol). The reaction mixture was stirred at room temperature overnight. It was then diluted with dichloromethane (5 mL) and washed with 2 M HCl (10 mL×3). The aqueous layers were extracted with dichloromethane (15 mL×3), dried over MgSO_4_, filtered and concentrated in *vacuo*. The crude was purified over a column of silica (dichloromethane:methanol, 9 : 1) to give **4** **a** as a colourless solid (451.0 mg, 76 %). ^1^H NMR (600 MHz, CDCl_3_) δ 7.74 (d, *J*=8.4 Hz, 2H, ArH), 7.43 (d, *J*=8.4 Hz, 2H, ArH), 3.66 (m, 2H, CH_2_), 3.24 (m, 2H, CH_2_), 1.63 (m, 4H, (CH_2_)_2_), 1.47(m, 2H, CH_2_). ^13^C NMR (151 MHz, CDCl_3_) δ 168.0 (C=O), 154.1(p, *J*=17.8 Hz, ArC‐SF_5_), 139.9 (ArC), 127.1 (2ArC), 126.2 (m, 2ArC‐CSF_5_), 48.6 (C), 43.1 (C), 26.4 (C), 25.4 (C), 24.3 (C); ^19^F NMR (400 MHz, CDCl_3_) δ 83.75 (m, F), 62.53 (d, *J*=150.0 Hz, 4F); LCMS Purity (UV)=95 %, *t*_R_ 19.5 min; HRMS‐ESI (*m/z*) found 316.0789, calc. for [C_12_H_14_F_5_NOS][H]^+^:316.0789; IR (neat) ν_max_/cm^−1^: 2945, 1627, 817; mp 84.5 ‐ 86.7 °C.

### (4‐Pentafluoro‐λ^6^‐sulfanylphenyl)(4‐methylpiperazin‐1‐ yl)methanone) (4 b)

To 1‐methyl piperazine (188.0 mg, 1.88 mmol), dissolved in dichloromethane (2 mL) was added triethylamine (190.3 mg, 1.88 mmol), followed by dropwise addition of 4‐(pentafluorosulfanyl) benzoyl chloride (250.0 mg, 0.94 mmol) and HATU (391.2 mg, 0.94 mmol). The reaction mixture was stirred at room temperature overnight and then diluted with dichloromethane (10 mL). The reaction mixture was filtered and the organic filtrate was washed with water (10 mL×3) and the aqueous layer extracted with dichloromethane (10 mL×3). The organic layer was then dried over MgSO_4_, filtered and concentrated in *vacuo*. The crude product was purified over a column of silica (dichloromethane:methanol, 9 : 1) to afford a yellow solid as the title compound (257 mg, 83 %). ^1^H NMR (600 MHz, CDCl_3_) δ 7.78 (m, 2H, ArH), 7.52 (m, 2H, ArH), 3.78 (m, 2H, CH_2_), 3.39 (m, 2H, CH_2_), 2.50 (m, 4H, (CH_2_)_2_), 2.30 (s, 3H, CH_3_). ^13^C NMR (151 MHz, CDCl_3_) δ 168.0 (C=O), 153.9 (m, ArC‐SF_5_), 136.6 (ArC), 130.1 (ArC), 129.1 (ArC), 127.1 (m, ArC‐CSF_5_), 124.9 (m, ArC‐CSF_5_), 54.8 (2 C), 47.6 (C), 45.9 (CH_3_), 42.2 (C); ^19^F NMR (400 MHz, CDCl_3_) δ 83.50 (m, F), 62.67 (d, *J*=150.3 Hz, 4F); LCMS Purity (UV)=98 %, *t*_R_ 11.6 min. HRMS‐ESI (*m/z*) found 331.0901, calc. for [C_12_H_15_F_5_N_2_OS][H]^+^:331.0898. IR (neat) ν_max_/cm^−1^ 2811.3, 1613.5, 822.4; mp 159.7–160.2 °C.

### [4‐(Pentafluorol‐λ^6^‐sulfanyl)phenyl)](morpholino)methanone (4 c)

To morpholine (180.3 mg, 2.07 mmol), dissolved in dichloromethane (2 mL) was added triethylamine (247.0 mg, 2.44 mmol), followed by dropwise addition of 4‐(pentafluorosulfanyl) benzoyl chloride (500.0 mg, 1.88 mmol). The reaction mixture was stirred at room temperature overnight. The reaction mixture was diluted with dichloromethane (5 mL) and the organic layer was washed with 2 M HCl (10 mL×3). The combined aqueous layers were extracted with dichloromethane (15 mL×3). The organic layers were dried over MgSO_4_, filtered and concentrated in *vacuo*. The crude material was purified over a column of silica (dichloromethane:methanol, 9 : 1) to afford a colourless solid as the title compound (556.0 mg, 93 %). ^1^H NMR (600 MHz, CDCl_3_) δ 7.58 (d, *J*=8.1 Hz, 2H, ArH), 7.30 (d, *J*=8.1 Hz, 2H, ArH), 3.33 (m, 8H, ((CH)_2_)_4_); ^13^C NMR (151 MHz, CDCl_3_) δ 168.1 (C=O), 154.2 (p, *J*=17.7 Hz, ArC‐SF_5_), 138.7 (ArC), 127.4 (2ArC), 126.3 (m, 2ArC), 66.5 (2 C, N‐(CH_2_)_2_, 47.9 (CH_2_), 42.4 (CH_2_); ^19^F NMR (400 MHz, CDCl_3_) δ 83.40 (p, *J*=150.3, F), 62.39 (d, *J*=150.3 Hz, 4F); LCMS Purity (UV)=95 %, *t*_R_ 18.3 min; HRMS‐ESI (*m/z*) found 340.0394, calc. for [C_11_H_12_F_5_NO_2_S] [Na]^+^: 340.0401. IR (neat) v_max_/cm^−1^: 3734 (CH), 1626 (s, C=O), 818 (SF_5_); mp 72–73 °C.

### tert‐Butyl 4‐(4‐pentafluoro‐λ^6^‐sulfanyl) benzoyl piperazine‐ 1‐carboxylate (4 d)

To *N*‐Boc piperazine (385.5 mg, 2.07 mmol), dissolved in dichloromethane (3 mL) was added triethylamine (247.0 mg, 2.44 mmol) and the mixture was stirred for a few minutes. HATU (787.1 mg, 2.07 mmol) was added to this solution followed by dropwise addition of 4‐(pentafluorosulfanyl) benzoyl chloride (500.0 mg, 1.88 mmol). The reaction mixture was stirred at room temperature overnight. The reaction mixture was diluted with dichloromethane (5 mL) and the precipitate formed was discarded after gravity filtration. MP‐Trisamine scavenger resin (520.0 mg, 3 mmol) was added to the filtrate and after 3 hours, the scavenger was removed by gravity filtration. The crude material was purified over a column of silica (dichloromethane:methanol, 9 : 1) to obtain **4** **d** as a white solid (352.0 mg, 76 %). ^1^H NMR (600 MHz, CDCl_3_) δ 7.67 (m, 2H, ArH), 7.37(m, 2H, ArH), 3.78–3.49 (m, 4H), 3.40–3.32 (m, 4H), 1.32 (s, 9H, (CH_3_)_3_); ^13^C NMR (151 MHz, CDCl_3_) δ 168.4 (C=O), 154.6–153.9 (m, 2 C, C=O, ArC‐SF_5_), 138.8 (ArC), 127.4 (2ArC), 126.5 (2ArC), 80.5 (C), 47.4 (2 C), 42.1 (2 C), 28.3 (3 C); ^19^F NMR (400 MHz, CDCl_3_) δ 83.32 (p, *J*=150.2 Hz, F), 62.47 (d, *J*=150.2 Hz, 4F); LCMS Purity (UV)=97 %, *t*_R_ 21.5 min; HRMS ‐ ESI (*m/z*) found: 439.1078, calc. for [C_16_H_21_F_5_N_2_O_3_S][Na]^+^: 439.1085. IR (neat) ν_max_/cm^−1^ 2920, 1637, 1279, 816; mp: 151–153 °C.

### 1‐[4‐[4‐(Pentafluoro‐λ^6^‐sulfanyl)benzoyl]piperazin‐1‐yl] ethanone (4 e)

Compound **4** **d** was dissolved in dichloromethane (2 mL) and treated with HCl in dioxane (4 M, 6.0 equiv.) overnight to remove the Boc group. The reaction was monitored by TLC. Following the completion of the reaction, it was concentrated in *vacuo* to obtain the HCl salt (4‐[4‐(pentafluoro‐λ^6^‐sulfanyl)benzoyl]piperazinyl hydrochloride, **4** **d’**), as a colourless solid. Compound **4** **d’** was used without purification in the following step.

To **4** **d’** (80.0 mg, 0.2 mmol), dissolved in THF (1.0 mL), was added triethylamine (20.0 mg, 0.2 mmol). The reaction mixture was stirred for 30 minutes, followed by which, acetic anhydride (20 mg, 0.2 mmol) was added to the mixture and this was stirred at room temperature overnight. The crude was dissolved in dichloromethane (5 mL) and passed through a hydrophobic frit, to collect the organic layer. Afterwards, the crude material was purified over a column of silica (dichloromethane:methanol; 7 : 3) to obtain the pure product as a colourless solid (59 mg, 82 %). ^1^H NMR (600 MHz, CDCl_3_) δ 7.81 (d, *J*=8.4 Hz, 2ArH), 7.49 (d, *J*=8.4 Hz, 2ArH), 3.89–3.64 (m, 4H, piperazine), 3.56 (s, 3H, CH_3_), 3.47–3.30 (m, 4H, piperazine); ^13^C NMR (151 MHz, CDCl_3_) δ 169.2 (Ar‐C=O), 168.5 (C=O), 154.6 (m, ArC‐SF_5_), 138.4 (ArC), 127.5 (2ArC), 126.6 (2ArC), 47.3 (bs, C), 46.1 (bs, C), 42.1 (bs, C), 41.3 (bs, C), 21.4 (CH_3_) ^19^F NMR (400 MHz, CDCl_3_) δ 83.32 (p, *J*=150.4 Hz, 1F), 62.96 (d, *J*=150.4 Hz, 4F); HPLC Purity (UV)=95 %, *t*_R_ 13.4 min; HRMS‐ESI (*m/z*) found 381.0665, calc. for [C_13_H_15_F_5_N_2_O_2_S][Na]^+^ :381.0667; IR (neat) ν_max_/cm^−1^ 2917(C−H), 1633 (C=O), 1625 (C=O), 820 (S−F); mp 158–160 °C.

### (4‐Pentafluoro‐λ^6^sulfanyl phenyl)(4‐(methylsulfonyl)piperazin‐1‐yl)methanone (4 f)

To crude **4** **d’** (82.0 mg, 0.23 mmol) dissolved in THF (3.0 mL) was added triethylamine (23.0 mg, 0.23 mmol) and the mixture was stirred for 30 minutes. Methanesulfonyl chloride (32.0 mg, 0.28 mmol) was added to the reaction mixture, which was stirred overnight at room temperature. The reaction mixture was quenched with dichloromethane (5 mL) and water (5 mL) then passed through a hydrophobic frit. The crude was purified over column of silica (dichloromethane:methanol; 7 : 3) to get the pure product as a colourless solid (43 mg, 48 %). ^1^H NMR (600 MHz, CDCl_3_) δ 7.83 (d, *J*=8.2 Hz, ArH, 2H), 7.50 (d, *J*=8.2 Hz, ArH, 2H), 3.90 (m, CH_2_, 2H), 3.52 (m, CH_2_, 2H), 3.34 (m, CH_2_, 2H), 3.18 (m, CH_2_, 2H), 2.81 (s, CH_3_, 3H); ^13^C NMR (151 MHz, CDCl_3_) δ 168.4 (C=O), 154.8 (m, C‐SF_5_), 138.1 (ArC), 127.5 (ArC, 2 C), 126.7 (ArC, 2 C), 47.2 (CH_2_), 46.0 (CH_2_), 45.5 (CH_2_), 41.8 (CH_2_), 35.1 (CH_3_); ^19^F NMR (400 MHz, CDCl_3_) δ 82.99 (p, *J*=150.3 Hz, 1F), 62.51 (d, *J*=150.3 Hz, 4F); HRMS ‐ ESI (*m/z*) found 417.0342, calc. for [C_12_H_15_F_5_N_2_O_3_S_2_][Na]^+^:417.0349; Anal. calc (%) for C_12_H_15_F_5_N_2_O_3_S_2_: C, 36.55; H, 3.83; N, 7.10; found (%): C, 36.65; H, 3.69; N, 6.98. IR (neat) ν_max_/cm^−1^2187 (C−H), 1637 (C=O), 1322 (S=O), 821 (S−F); mp 163–164 °C.

### (3Z)‐3‐[(3,5‐Dimethyl‐1H‐pyrrol‐2‐yl)methylidene]‐5‐(pentafluoro‐λ^6^‐sulfanyl)‐2,3‐dihydro‐1H‐indol‐2‐ one (8 a)

In a 10 mL microwave vial equipped with a stirrer bar was added 5‐(pentafluorosulfanyl)‐1,3‐dihydro‐indol‐2‐one (0.096 g, 0.37 mmol), 3,5‐dimethyl‐1H‐pyrrole‐2‐carbaldehyde, ethanol (2.5 mL) and 3 drops of piperidine. The vessel was then sealed using a rubber microwave septum and placed into the microwave cavity. The reaction mixture was subjected to microwave irradiation by ramping to 150 °C with 200 W of power. It was held at that temperature for 30 min. The vessel was then cooled to ambient temperature and solvent removed in *vacuo*. The crude was purified over a column of C18 silica (100 % acetonitrile) to obtain purified **8** **a** as a red solid (0.137 g, 80 %). ^1^H NMR (600 MHz, (CD_3_)_2_CO) δ 13.46 (1H, s, NH), 10.15 (1H, s, NH), 8.21 (1H, s, ArH), 7.91 (1H, s, ArH), 7.60 (1H, d, *J*=8.6 Hz, ArH), 7.10 (1H, d, *J*=8.6 Hz, ArH), 6.06 (1H, s, C=H), 2.38 (3H, s, CH_3_), 2.35 (3H, s, CH_3_); ^13^C NMR (151 MHz, (CD_3_)_2_CO) δ 169.8 (C=O), 147.7 (m, ArC‐SF_5_), 140.0 (ArC=C), 138.0 (ArC), 134.4 (ArC), 127.4 (ArC), 126.6 (ArC), 125.4 (m, ArC‐CSF_5_), 123.0 (ArC), 115.5 (ArC), 113.3 (m, ArC‐CSF_5_), 110.5 (C=C), 108.5 (ArC), 12.9 (CH_3_), 10.7 (CH_3_); ^19^F NMR (400 MHz, (CD_3_)_2_CO) δ 87.2 (1F, m, equatorial), 63.74 (1F, d, *J*=148.1 Hz, axial); LCMS Purity (UV)=100 %, *t*_R_ 23.6 min; HRMS‐ESI (*m/z*) found 365.0737, calc. for [C_15_H_13_F_5_N_2_OS][H]^+^: 365.0742; IR (neat) ν_max_/cm^−1^ 3060 (N−H), 1659 (C=O), 820 (S−F); mp 203–205 °C.

### 5‐(Pentafluoro‐λ^6^‐sulfanyl)‐3‐(propan‐2‐ylidene)‐2,3‐dihydro‐1H‐indol‐2‐one (8 b)

In a 10 mL microwave vial equipped with a stirrer bar was added 5‐(pentafluorosulfanyl)‐1,3‐dihydro‐indol‐2‐one (0.096 g, 0.37 mmol) in acetone (2 mL) followed by 3 drops of piperidine. The vessel was then sealed using a rubber microwave septum and placed into the microwave cavity. The reaction mixture was irradiated with 200 W of power. It was maintained at 100 °C by moderation of power for 20 min. The vessel was then cooled to room temperature. The reaction mixture was extracted with ethyl acetate (3×20 mL), washed with water (20 mL), dried over MgSO_4_, filtered under gravity and concentrated in *vacuo*. The obtained orange solid was used without further purification (0.100 g, 90 %). Crystallization by mixed solvents, dichloromethane and hexane, provided orange crystals of **8** **b**. ^1^H NMR, (600 MHz, (CD_3_OD) δ N−H not observed, 7.87 (1H, s, ArH), 7.65 (1H, d, *J*=8.5 Hz, ArH), 6.94 (1H, d, *J*=8.5 Hz, ArH),2.59 (3H, s, CH_3_), 2.39 (3H, s, CH_3_), ^13^C NMR (600 MHz, CD_3_OD) δ 169.6 (C=O), 159.0 (C=C), 147.6 (ArC‐SF_5_, m), 142.5 (ArC), 125.6 (ArC‐CSF_5_, m), 123.6 (ArC), 121.8 (ArC), 120.7 (C, ArC‐CSF_5_, m), 108.2 (C=C), 24.0 (CH_3_), 21.9 (CH_3_); ^19^F NMR, (400 MHz, CD_3_OD) δ 62.9 (4F, d, *J*=147.8 Hz, equatorial), 85.3(1F, p, *J*=147.8, axial); LCMS Purity (UV)=99 %, *t*_R_ 18.5 min; HRMS‐ESI (*m/z*) found 322.0285, calc. for [C_11_H_10_F_5_NOS][Na]^+^:322.0295; IR (neat) ν_max_/cm^−1^ 3062 (N−H), 2925 (C−H), 1694 (C=O), 1618 (C=C), 813 (S−F); mp 191–194 °C.

### (3Z)‐3‐[(3,5‐Dimethyl‐1H‐pyrrol‐2‐yl)methylidene]‐6‐(pentafluoro‐λ^6^‐sulfanyl)‐2,3‐dihydro‐1H‐indol‐2‐one (9 a)

In a 10 mL microwave vial equipped with a stirrer bar was added 6‐(pentafluorosulfanyl)‐1,3‐dihydro‐indol‐2‐one(0.100 g, 0.4 mmol), 3,5‐dimethyl‐1H‐pyrrole‐2‐carbaldehyde (0.062 g, 0.5 mmol), ethanol (2.5 mL) and 3 drops of piperidine. The vessel was then sealed using a rubber microwave septum and placed into the microwave cavity. The reaction mixture was irradiated with 200 W of power. It was maintained at 150 °C by moderation of power for 30 min. The vessel was then cooled to room temperature and solvent removed in *vacuo*. The aqueous layer was extracted using ethyl acetate (3×20 mL), washed with brine (3×20 mL), dried over MgSO_4_, filtered under gravity and concentrated in *vacuo*. The crude was purified over a column of C18 silica (100 % acetonitrile) (70 mg, 50 %) to obtain an orange solid. ^1^H NMR (600 MHz, (CD_3_)_2_CO) δ 13.52 (1H, s, NH), 10.02 (1H, s, NH), 7.82 (1H, d, *J*=8.5 Hz, ArH), 7.79 (1H, s, ArH), 7.48 (1H, d, *J*=8.5 Hz, ArH), 7.41 (1H, s, ArH), 6.08 (1H, s, C=H), 2.36 (3H, s, CH_3_), 2.38 (3H, s, CH_3_). ^13^C NMR (151 MHz, (CD_3_)_2_CO) δ 169.6 (C=O), 138.7 (ArC‐SF_5_), 137.3 (ArC=C), 135.0 (ArC), 130.2 (ArC), 127.7 (ArC), 126.0 (ArC‐CSF_5_), 118.6 (ArC), 117.0 (ArC), 113.6 (ArC‐CSF_5_), 110.0 (ArC), 106.8. (ArC), 54.1 (C=C), 12.9 (CH_3_), 10.7 (CH_3_). ^19^F NMR (400 MHz, (CD_3_)_2_CO) δ 86.32 (1F, m), 63.74 (4F, d, *J*=148.1 Hz); LCMS Purity (UV)=100 %, *t*_R_ 23.7 min; HRMS‐ESI (*m/z*) found 365.0742, calc. for [C_15_H_13_F_5_N_2_O_3_S][Na]^+^: 365.0742. IR (neat) ν_max_/cm^−1^ 3559, 1650, 814; mp 199–201 °C.

### 5‐Nitro‐3‐(propan‐2‐ylidene)‐2,3‐dihydro‐1H‐indol‐2‐one (10 a)

#### Microwave reaction procedure

In a 10 mL microwave vial equipped with a stirrer bar was added 5‐nitro‐2‐oxindole (0.50 g, 2.85 mmol), acetone (0.25 mL, 3.42 mmol), acetonitrile (5 mL) and 3 drops of piperidine. The vessel was then sealed using a rubber microwave septum and placed into the microwave cavity. The reaction mixture was irradiated with 200 W of power and heated to 100 °C in a CEM Explorer. It was maintained at 100 °C by moderation of power for 20 min. The vessel was then cooled to ambient temperature and the reaction mixture was concentrated in *vacuo*. The crude material was recrystallized in acetonitrile to obtain a brown solid as pure product (0.411 g, 66 %).

#### Solventless reaction procedure

5‐Nitro‐2‐oxindole (242.0 mg, 1.36 mmol), acetone (200.0 mL, 2.71 mmol) and piperidine (40.2 mL, 0.41 mmol) were mechanically activated in a Retsch MM400 vibratory ball‐mill for 90 minutes at 30 Hz in 25 mL zirconia jar with one, 1.2 cm dia. ball. The product was suspended in acetone (10 mL) and filtered to obtain the pure product as a brown solid (229.0 mg, 78 %). ^1^H NMR (600 MHz, (CD_3_)_2_SO) δ 10.96 (1H, s, NH), 8.21 (1H, s, ArH), 8.11 (1H, dd, *J*=8.6, 2.0 Hz, ArH), 6.94 (1H, d, *J*=8.6 Hz, ArH), 2.52 (3H, s, CH_3_), 2.38 (3H, s, ArH).^13^C NMR (151 MHz, (CD_3_)_2_SO) δ 169.0 (C=O), 160.0 (ArC), 146.6 (ArC), 141.9 (ArC), 125.0 (ArC−H), 124.1 (ArC‐NH), 121.5 (C=ArC), 118.9 (ArC−H), 109.4 (ArC−H), 25.5 (CH_3_), 23.2 (CH_3_); HRMS‐ESI (m/z) found 241.0584, calc. for [C_11_H_10_N_2_O_3_][Na]^+^: 241.0584; IR (neat) ν_max_/cm^−1^1694 (C=O), 1509 (N−O stretch); LCMS Purity (UV)=100 %, *t*_R_=14.6 min; mp 228–230 °C.

#### 5‐(Pentafluoro‐λ^6^‐sulfanyl)‐1,2‐dihydrospiro[indole‐3,4′‐ oxan]‐2‐one (11 a)

5‐(Pentafluorosulfanyl)‐1,3‐dihydro‐indol‐2‐one (0.500 g, 1.93 mmol) dissolved in tetrahydrofuran (10 mL) was added to LiHMDS (1 M) in tetrahydrofuran (5.80 mL, 5.80 mmol) at −78 ^°^C. After an hour of stirring, 1‐bromo‐2‐(2‐bromoethoxy)ethane (0.448 g, 1.93 mmol) was added to the mixture which was allowed to warm to ambient temperature. The reaction was stirred for 46 hours, then quenched with water. The reaction mixture was extracted with ethyl acetate (3×20 mL), washed with brine (3×20 mL), dried over MgSO_4_, filtered under gravity and concentrated in *vacuo*. The crude material was purified over a column of silica (hexane:ethyl acetate; 4 : 1) to afford a pink solid as the title compound (210 mg, 33 %). ^1^H NMR (600 MHz, CD_3_OD) δ 7.82 (1H, s, ArH), 7.71 (1H, d, *J*=8.6 Hz, ArH), 7.01 (1H, d, *J*=8.6 Hz, ArH), 4.22 (2H, m, OCH_2_), 3.88 (2H, m, OCH_2_), 1.95 (2H, m, CH_2_), 1.82 (2H, m, CH_2_); ^13^C NMR (151 MHz, (CD_3_)_2_CO) δ 180.7 (C=O), 147.8 (m, ArC‐SF_5_), 144.3 (ArC), 135.2 (ArC), 126.7 (m, ArC‐CSF_5_), 121.2 (m, ArC‐CSF_5_), 109.0 (ArC), 62.0 (2 C), 44.6 (C), 32.5 (2 C);^19^F NMR (400 MHz, (CD_3_)_2_CO) δ 86.41 (4F, m), 64.09 (F, d, *J*=148.1 Hz); LCMS Purity (UV)=98 %, *t*_R_=20.2 min; HRMS‐ESI (*m/z*) found 329.0535, calc. for [C_12_H_11_F_5_NO_2_S][H]^+^: 329.0509; IR (neat) ν_max_/cm^−1^ 3303, 1690, 1101, 818; mp 247–249 °C.

#### 1‐Methyl‐5‐(pentafluoro‐λ^6^‐sulfanyl)‐1,2‐dihydrospiro[indole‐ 3,4′‐oxan]‐2‐one (11 b)

Sodium hydride (11 mg, 0.45 mmol) was added to a round bottomed flask purged with argon. Dry dimethyl formamide (15 mL) was then added to the flask followed by compound **11** **a** (0.1 g, 0.30 mmol) and stirred for 1 hour. To this mixture was added methyl iodide (0.213 g, 1.50 mmol) and stirred overnight at room temperature. The reaction mixture was quenched with water (10 mL), extracted with ethyl acetate (10 mL×3), washed with brine (20 mL), dried over MgSO_4_, filtered, and concentrated in *vacuo*. The crude material was purified over a column of silica (hexane:ethyl acetate;4 : 1) to give **11** **b**, as a yellow solid (68 mg, 66 %). ^1^H NMR (600 MHz, CDCl_3_) δ 7.71 (ArH, dd, *J*=8.6, 1.9 Hz, 1H), 7.68 (ArH, d, *J*=1.9 Hz, 1H), 6.86 (ArH, d, *J*=8.6 Hz, 1H), 4.25 (CH_2_, ddd, *J*=12.0, 9.5, 3.0 Hz, 2H), 3.89 (CH, dt, *J*=12.0, 4.3 Hz, 2H), 3.22 (s, 3H), 1.91 (ddd, *J*=13.4, 9.5, 4.3 Hz, 2H), 1.82 (dt, *J*=13.4, 3.0 Hz, 2H).^13^C NMR (151 MHz, CDCl_3_) δ 179.5 (C=O), 148.8 (ArC, p, ^*2*^ *J_FC_* =17.7, 17.2 Hz), 145.1 (ArC), 134.0 (ArC), 126.7 (ArC−H, p, ^*3*^ *J_FC_* =4.7 Hz), 120.9 (ArC−H, p, ^*3*^ *J_FC_* =4.7 Hz), 107.3 (ArC−H), 62.6 (2 C), ((CH_2_)_2_), 44.4 (C), 32.7 (2 C, (CH_2_)_2_), 26.3 (CH_3_).^19^F NMR (400 MHz, CDCl_3_) δ 86.61–84.84 (p, *J*=150.1 Hz), 64.42 (d, *J*=150.1 Hz); HRMS‐ESI (m/z) found 344.0743, calc. for [C_13_H_14_F_5_NO_2_S][H]^+^: 344.0744; IR (neat) ν_max_/cm^−1^ 2947 (C−H, st), 1727 (C=O), 1258 (N−C, methyl), 828 (S−F); LCMS Purity (UV)=95 %, *t*_R_ 18.7 min; mp: 158–158 °C.

#### Methyl 3‐{2‐[3‐(trifluoromethyl)phenyl]acetamido}pyridine‐4‐carboxylate (13 a)

To methyl 3‐aminopyridine‐4‐carboxylate (100.0 mg, 0.66 mmol) in dichloromethane (5 mL) was added triethylamine (134.0 mg, 1.32 mmol) followed by dropwise addition of 3‐(trifluoromethyl)phenyl acetyl chloride (220.0 mg, 0.99 mmol). The reaction mixture was stirred at room temperature for 48 h and monitored by TLC. After 48 h, it was diluted by dichloromethane (10 mL), washed with 2 M HCl (10 mL×3) and the aqueous layer was extracted with dichloromethane (25 mL). The combined organic layers were washed with sodium bicarbonate (25 mL), brine (15 mL×3), dried over MgSO_4_, filtered and concentrated in *vacuo*. The crude material was purified over a column of silica (dichloromethane:methanol; 19 : 1) to give **13** **a**, as a colourless solid (144 mg, 65 %), which was used as such for the next step. ^1^H NMR (600 MHz, (CD_3_)_2_SO) δ 10.51 (s, 1H, NH), 8.98 (s, 1H, ArH), 8.47 (d, *J*=5.0 Hz, 1H, ArH), 7.73 (s, 1H, ArH), 7.67–7.63 (m, 4H, ArH), 3.87 (s, 2H, CH_2_), 3.67 (s, 3H).

#### Methyl 3‐[2‐(3‐chlorophenyl)acetamido]pyridine‐4‐carboxylate (13 c)

*N,N*‐Diisopropylethylamine (1.28 g, 9.92 mmol) was added to a stirred solution of 2‐(3‐chlorophenyl)acetic acid (0.56 g, 3.29 mmol) and hexafluorophosphate azabenzotrizole tetramethyl uronium (HATU, 1.5 g, 3.95 mmol), in DMF (10 mL). The solution was stirred for 30 min at room temperature and turned green. Methyl 3‐aminoisonicotinate (0.5 g, 3.29 mmol) was then added to the mixture, which was stirred at room temperature overnight. Once the reaction was completed, water (10 mL) was added to the mixture and the product extracted with ethyl acetate (3×10 mL). This extract was then washed with brine (3×10 mL) dried over MgSO_4_ and the solvent removed in vacuo. The resultant beige solid was purified by flash chromatography ethyl acetate, hexane (1 : 1) to yield the target compound (0.66 g, 66 %). ^1^H NMR (600 MHz, (CD_3_)_2_SO) δ 10.47 (s, 1H), 9.04 (s, 1H), 8.46 (d, *J*=4.9 Hz, 1H), 7.65 (d, *J*=4.9 Hz, 1H), 7.44 (s, 1H), 7.40–7.30 (m, 3H), 3.78 (s, 2H), 3.71 (s, 3H). ^13^C NMR (151 MHz, DMSO‐*d*_6_) δ 169.1, 165.8, 145.3, 144.8, 137.5, 132.9, 132.5, 130.2, 129.3, 128.6, 128., 126., 122.3, 52.5, 42.4.

#### 2‐{[3‐(Trifluoromethyl)phenyl]methyl}‐3H,4H‐pyrido[3,4‐d]pyrimidin‐4‐one (14 a)

Product **13** **a** (144 mg, from above) was refluxed with 7 M ammonia in methanol (10 mL) for 48 hours. After cooling, the crude product was diluted with dichloromethane (15 mL) and washed with 2 M HCl (10 mL×2). The combined organic layers were dried over MgSO_4_, filtered, and concentrated in *vacuo*. The crude mixture was purified over a column of silica (dichloromethane:methanol; 97 : 3) to obtain the title compound as a colourless solid (30 mg, 23 %). Crystallization of **14** **a** was achieved by dissolving it in minimum amount of dichloromethane. ^1^H NMR (600 MHz, (CD_3_)_2_SO) δ 12.79 (s, 1H, NH), 8.94 (s, 1H, ArH), 8.59 (d, *J*=5.1 Hz, 1H, ArH), 7.89 (d, *J*=5.1 Hz, 1H, ArH), 7.77 (s, 1H, ArH), 7.67 (d, *J*=7.8 Hz, 1H, ArH), 7.62 (d, *J*=7.8 Hz, 1H, ArH), 7.58–7.52 (m, 1H, ArH), 4.08 (s, 2H, CH_2_); ^13^C NMR (151 MHz, CD_3_OD) δ 161.5 (C=O), 158.1 (ArC), 149.8 (ArC), 145.1 (ArC), 143.9 (ArC), 136.7 (ArC), 132.5 (ArC), 130.6 (q, ^2^ *J*_FC_=32.0 Hz, ArC‐CF_3_), 129.2 (ArC), 126.4 (ArC), 125.5 (q, ^3^ *J*_FC_=4.0 Hz, ArC), 124.2 (q, ^1^ *J*_FC_=271.4 Hz, ArC), 123.7 (q, ^3^ *J*_FC_=4.0 Hz, ArC), 118.4 (ArC), 40.5 (CH_2_); ^19^F NMR (400 MHz, CDCl_3_) δ −62.71 (s, 3F); HRMS‐ESI (*m/z*) found 306.0845, calc. for [C_15_H_10_F_3_N_2_O][H]^+^:306.0869; LCMS Purity (UV)=90 %, *t*_R_ 14.9 min; IR (neat) ν_max_/cm^−1^: 2769 (NH), 1704 (C=O), 1329 (CF_3_).

#### 2‐{[3‐(Pentafluoro‐λ^6^‐sulfanyl)phenyl]methyl}‐3H,4H‐pyrido[3,4‐]‐pyrimidin‐4‐one (14 b)

To 2‐[3‐(pentafluoro‐λ^6^‐sulfanyl)phenyl]acetic acid (120.0 mg, 0.46 mmol) was added thionyl chloride (201.0 μL, 2.76 mmol) and 2 drops of DMF. The reaction was refluxed under argon overnight. The reaction was followed by LCMS and following completion of the reaction, it was concentrated in *vacuo* to obtain 2‐[3‐(pentafluoro‐λ^6^‐sulfanyl)phenyl]acetyl chloride. The crude was taken through to the following steps without purification (115 mg).

To methyl 3‐aminopyridine‐4‐carboxylate **12** (41.0 mg, 0.27 mmol) in dichloromethane (5 mL) was added triethylamine (55.0 mg, 0.54 mmol) followed by dropwise addition of 2‐[3‐(pentafluoro‐λ^6^‐sulfanyl)phenyl]acetyl chloride ((made as above) 115.0 mg, 0.40 mmol). The reaction mixture was stirred at room temperature for 48 h and monitored by TLC. After 48 h, it was diluted with dichloromethane (10 mL), washed with 2 M HCl (10 mL×3) and aqueous layer extracted with dichloromethane (25 mL). The combined organic layers were washed with sodium bicarbonate (25 mL), brine (15 mL×3), dried over MgSO_4_, filtered and concentrated in *vacuo*. The crude material was purified using a column of silica (dichloromethane:methanol; 19 : 1) to obtain crude **13** **b**, as a colourless solid (144 mg, 65 %). The latter was used as such and was refluxed with 7 M ammonia in methanol (10 mL) for 48 hours. After cooling, the crude mixture was diluted with dichloromethane (15 mL) and washed with 2 M HCl (10 mL×2). The combined organic layers were dried over MgSO_4_, filtered, and concentrated in *vacuo*. The crude was purified over a column of silica (dichloromethane:methanol; 97 : 3) to obtain the title compound as a colourless solid (9 mg, 25 %). ^1^H NMR (600 MHz, CDCl_3_) δ 11.85 (s, 1H, NH), 9.20 (s, 1H, ArH), 8.76 (d, *J*=5.1 Hz, 1H, ArH), 8.05 (d, *J*=5.1, 1H, ArH), 7.97 (dd, *J*=8.0 Hz, 1.9 Hz, 1H, ArH), 7.71–7.67 (m, 1H, ArH), 7.64 (d, *J*=7.7 Hz, 1H, ArH), 7.49–7.44 (m, 1H, ArH), 4.19 (s, 2H, CH_2_); ^13^C NMR (151 MHz, CDCl_3_) δ 161.7 (C=O), 156.0 (ArC), 154.3–153.7 (m, ArC‐SF_5_), 151.1 (ArC), 146.3 (ArC), 143.5 (ArC), 136.2 (ArC), 132.3 (ArC), 129.2 (ArC), 126.8 (m, ArC), 126.0 (ArC), 125.1 (m, ArC), 118.1 (ArC), 41.5 (C). ^19^F NMR (400 MHz, CDCl_3_) δ 87.64–85.94 (p, *J*=148.4 Hz, 1F), 65.21 (d, *J*=148.4 Hz, 4F); HRMS‐ESI (*m/z*) found 364.0543, calc. for [C_14_H_10_F_5_N_3_OS][H]^+^:364.0527; LCMS Purity (UV)=92 %, *t*_R_ 16.2 min; IR (neat) ν_max_/cm^−1^: 2854 (NH), 1672 (C=O), 834 (SF_5_).

#### 5‐Methyl‐N‐[4‐(pentafluoro‐λ^6^‐sulfanyl)phenyl]‐1,2‐oxazole‐ 4‐carboxamide (15)

4‐(Pentafluorosulfanyl)aniline (500 mg, 2.28 mmol) was dissolved in isopropanol (IPA) (4 mL) followed by the addition of NaHCO_3_ (201 mg, 2.39 mmol). The mixture was heated to 60 °C and 5‐methyl‐4‐isoxazolecarbonyl chloride (0.2 mL, 2.28 mmol) was added dropwise. The reaction was followed by TLC and after 2 hours of stirring, the reaction was allowed to cool to ambient temperature. The white precipitate formed was filtered under vacuum and washed with water and toluene, then dried to give the product (530 mg, 70 %). ^1^H NMR (600 MHz, CDCl_3_) δ 8.47 (s, 1H, NH), 7.77–7.72 (m, 2H, ArH), 7.67 (d, *J*=8.8 Hz, 2H, ArH), 7.55 (s, 1H, ArH), 2.77 (s, 3H, CH_3_); ^13^C NMR (151 MHz, CDCl_3_) δ 174.3 (C=O), 159.2 (C=N), 149.7 (C‐SF_5_), 147.3 (ArC), 139.8 (ArC), 127.2 (2 C, m, ArC), 119.5 (2 C, ArC), 111.6 (ArC), 12.7 (CH_3_); ^19^F NMR (400 MHz, CDCl_3_) δ 84.62 (p, *J*=150 Hz), 63.34 (d, *J*=150 Hz). LCMS Purity (UV)=97 %, tR 20.12 min; HRMS‐ESI (m/z) found 329.0385, calc. for [C_11_H_9_F_5_N_2_O_2_S][H]^+^: 329.0383; IR (neat) νmax/cm^−1^: 3366 (N−H), 1663 (C=O), 824 (S−F); mp=167–168 °C.

#### (2Z)‐2‐Cyano‐3‐hydroxy‐N‐[4‐(pentafluoro‐λ^6^‐sulfanyl)phenyl] but‐2‐enamide (16)

Compound **15** (304 mg, 0.93 mmol) was suspended in a mixture of IPA (2 mL) and water (2 mL). To the stirred mixture was added dropwise an aqueous solution of NaOH (39 % in water) until the solution reached a pH of *ca*. 12, at which point, all of the starting material dissolved. The solution was then filtered and concentrated. Concentrated HCl was added to the filtrate until pH 3. The resultant precipitate was filtered, washed with water and dried under vacuum to obtain the product as a colourless powder (283 mg, 93 %). ^1^H NMR (600 MHz, CDCl_3_) δ 7.79–7.74 (m, 2H, ArH), 7.62 (d, *J*=8.8 Hz, 2H, ArH), 2.38 (s, 3H, CH_3_); N−H and O−H not found; ^13^C NMR (151 MHz CDCl_3_) δ 189.2 (C=O), 167.5 (CN), 150.2 (C‐SF_5_), 138.6 (ArC), 127.3 (2 C, ArC, m), 120.2 (2 C, ArC), 116.1(C), 80.6 (C), 22.1 (CH_3_); ^19^F NMR (400 MHz, CDCl_3_) δ 84.82 (p, *J*=150.4 Hz), 63.34 (d, *J*=150.4 Hz). HPLC Purity (UV)=99 %, *t*_R_ 17.91 min; HRMS‐ESI (*m/z*) found 327.0247, calc. for [C_11_H_9_F_5_N_2_O_2_S][H]^+^: 327.0227; IR (neat) ν_max_/cm^−1^: 3312 (N−H), 2219 (CN), 1641 (C=O), 1589 (C=C), 825 (S−F); mp=167–170 °C.

## Conflict of interest

The authors declare no conflict of interest.

## Supporting information

As a service to our authors and readers, this journal provides supporting information supplied by the authors. Such materials are peer reviewed and may be re‐organized for online delivery, but are not copy‐edited or typeset. Technical support issues arising from supporting information (other than missing files) should be addressed to the authors.

Supporting Information
