# Supplementary material for: Expanding the Repertoire of Low‐Molecular‐Weight Pentafluorosulfanyl‐Substituted Scaffolds
Source: ChemMedChem. 2022 Feb 22;17(7):e202100641. doi: 10.1002/cmdc.202100641 (PMC9305131; doi:10.1002/cmdc.202100641)

ChemMedChem

Supporting Information

Expanding the Repertoire of Low-Molecular-Weight Pentafluorosulfanyl-Substituted Scaffolds

Arathy Jose, Daniel Guest, Remi LeGay, Graham J. Tizzard, Simon J. Coles, Mariliza Derveni, Edward Wright, Lester Marrison, Alpha A. Lee, Aaron Morris, Matt Robinson, Frank von Delft, Daren Fearon, Lizbé Koekemoer, Tetiana Matviuk, Anthony Aimon, Christopher J. Schofield, Tika R. Malla, Nir London, Barnaby W. Greenland, Mark C. Bagley, John Spencer[+],* and The Covid Moonshot Consortium

Table of Contents

**Table S1**	**P 2-4**
**Experimental Details**	**P 4-8**
**Scanned 1H, 13C, 19F, IR, HRMS**	**P 8-61**

Entry	Compound	
1	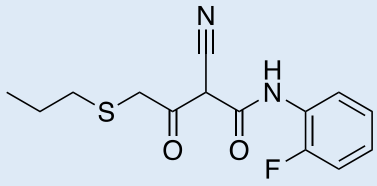	-
2	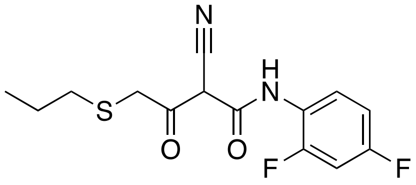	-
3	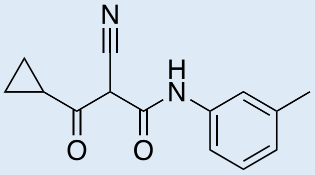	-
4	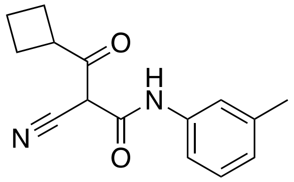	-
5	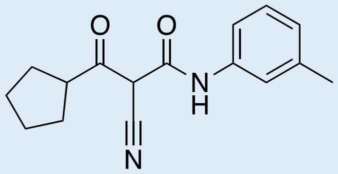	-
6	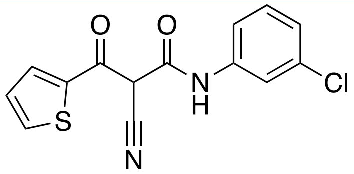	-
7	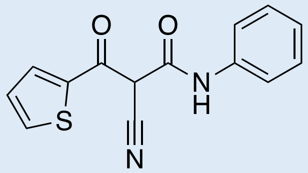	-
8	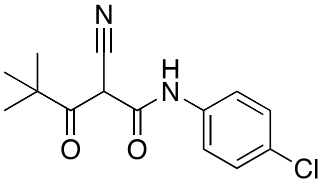	-

9	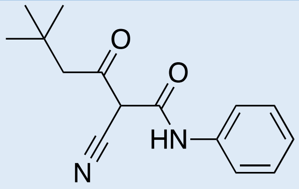	-
10	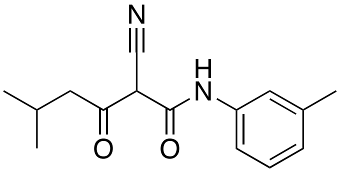	-
11	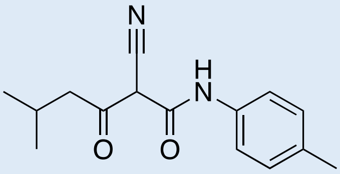	-
12	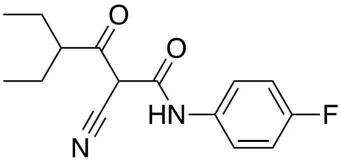	-
13	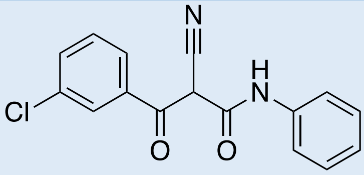	-
14	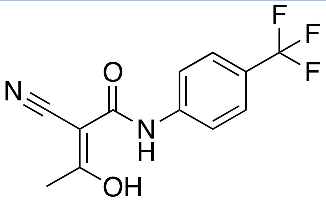	-
15	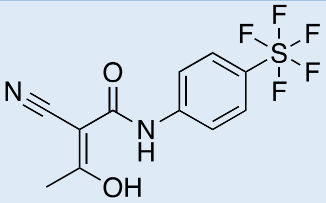	-
16	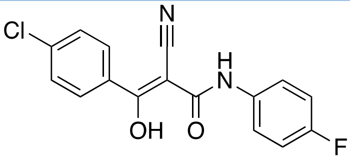 **17**	0.23

		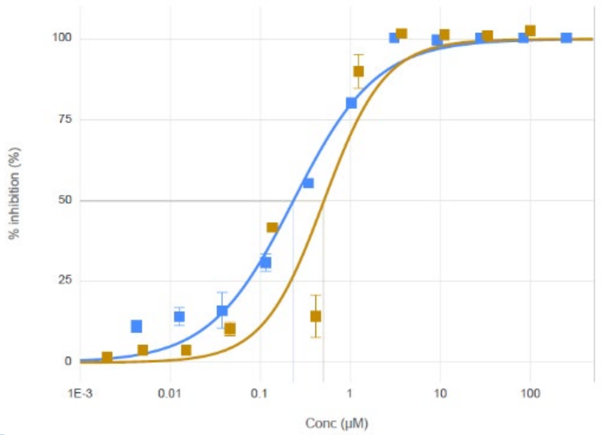

Table S1: Compounds donated to the Covid Moonshot initiative-IC_{50} vs MPro by eMolecules. Drawn optionally in keto or enol tautomeric form.

Effect of teriflunomide compounds on SARS CoV2 infection of a mammalian target cell line.

HEK293T/17 cells were transiently transfected with human ACE2 and TMPRSS2. The three compounds were added to the HEK293T/17 cells in concentrations ranging from 200 to 0.4 □M followed by addition of SARS-CoV-2. Control wells included virus and target cell without ligands/DMSO and target-cell only wells. Furthermore, a toxicity assay (MTS assay) was carried out to assess cell viability. HEK293T/17 at a concentration of $4x10^5$ cells/mL was added to each well containing teriflunomide and its analogues (200 to 0.4 □M concentration range) as well as to a control well with only DMSO. A cell only control well was also set up. After incubating the wells for 48 hours in a 37 ˚C/5% CO_2 incubator they were examined microscopically. 20 mL of MTS reagent was then added to the wells and incubated for 4 hours.

Materials & Methods

Mammalian target cell line

The human kidney HEK293T/17 cell line (ATCC® CRL-11268™) was used in the cytotoxicity and infection inhibition assays. The cells were maintained in Dulbecco’s Modified Eagle’s Medium (DMEM) (Sigma Aldrich – cat. No. D6429) at a 10% concentration of Fetal Bovine Serum (Sigma Aldrich – cat. No. F9665) (10% DMEM).

Pseudotyped SARS CoV2

An in-house produced, HIV-based pseudotyped virus was used in these experiments, displaying the Wuhan-Hu-1 isolate (Genbank accession number MN908947.3) spike S protein of SARS CoV2 and carrying a firefly luciferase reporter gene for infection assessment.

Teriflunomide compound preparation

The three teriflunomide compounds (teriflunomide, SF_5-teriflunomide and F-Ph-Cl) arrived in powder form and initial stocks were prepared using 100% DMSO (50mM for teriflunomide and SF_5-teriflunomide and 25mM for F-Ph-Cl).

Cytotoxicity Assay

The cytotoxicity assay was performed using the CellTitre 96 AQueous One Solution Cell Proliferation assay (MTS) kit from Promega (cat. No. G3582) following the manufacturer's instructions. Briefly, each of the three compounds was serially diluted two-fold into 50 µl 10% DMEM, each in duplicate wells of a clear, 96-well tissue culture microplate (Helena Biosciences – cat. No. 92096), in a concentration range between 200–0.4µM. DMSO containing no compound was used as a negative control, in a concentration range between 0.8–0.02% in 10% DMEM. 50 µl HEK293T/17 at a concentration of $4x10^5$ cells/ml were added to the wells (final cell number/well: $2x10^4$). Control wells with cells but no compound were also set up and the microplate was placed in a 37°C/5% CO_2 incubator for 48 hours. At the end of the incubation period, the cells were examined microscopically before 20 µl of MTS reagent were added to each well and the plate was returned to the incubator for 4 hours. The absorbance (490 mm) was measured using a Promega GloMax microplate reader.

Infection Inhibition Assay

Twenty-four hours prior to the infection inhibition assay, HEK293T/17 cells were transiently transfected with human ACE2 and TMPRSS2 using the polyetylenimine (PEI) method.

The infection assays were performed in white, flat-bottomed 96-well microplates (Fisher Scientific – cat. No. 10072151). Each of the three compounds was serially diluted two-fold into 50 µl 10% DMEM, in duplicate microplate wells, in a concentration range between 200–0.4µM. DMSO containing no compound was used as a negative control, in a concentration range between 0.8–0.02% in 10% DMEM. 50 µl pseudotyped SARS CoV2 in 10% DMEM at an MOI of 0.044 were added to each compound- or DMSO-containing well. Control wells included virus and target cells without compound/DMSO ("maximum" infection controls) and target cell-only wells ("zero" infection controls). After the addition of the virus, the target cells were collected using trypsin (Sigma Aldrich – cat. No. T3924), counted and $2x10^4$ cells were added to each microplate well. The plates were placed in a 37°C/5% CO_2 incubator for 48 hours.

At the end of the incubation period, the culture medium was discarded and the infection efficiency was measured using the Promega Bright Glo luciferase assay system (cat. No. E2650) using a Promega GloMax microplate reader.

Results

Cytotoxicity assay

The cells were microscopically examined prior to the addition of the MTS reagent and appeared healthy (albeit at varying levels of confluency between 60-90%) at concentrations between 50 – 0.4 μM. The cells in all the DMSO-only wells showed no visible difference to the cell-only control wells.

The addition of the MTS reagent revealed the cytostatic effect of the teriflunomide compounds (Figure 1) – the greater the cytostatic effect the lower the absorbance. Teriflunomide was the most toxic with a cytostatic effect at concentrations above 0.78 μM, and F-Ph-Cl the least toxic (cells healthy up to 50 μM)s. SF_5-teriflunomide had a cytostatic effect at concentrations of >3.1 μM and DMSO had no measurable effect.

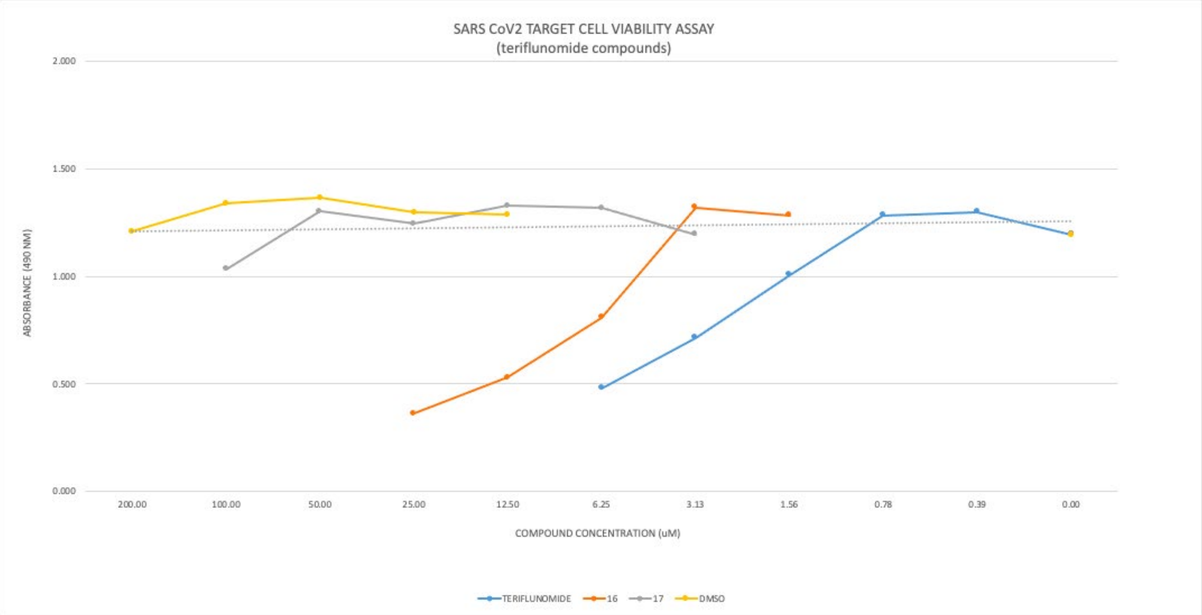

Figure S1: Effect of the teriflunomide compounds on HEK293T/17 viability using a colourimetric (MTS) assay

Infection Inhibition Assay

An infection inhibition assay showed that none of the three compounds had an inhibitory effect on the SARS CoV2 infection of the target cells (Figure 2). After excluding the toxic concentration ranges for each compound and normalising the data against the 100- and 0% infection controls, the teriflunomide/ SF_5-teriflunomide were shown to have no effect on the efficiency of infection and the F-Ph-Cl appeared to have an enhancing effect.

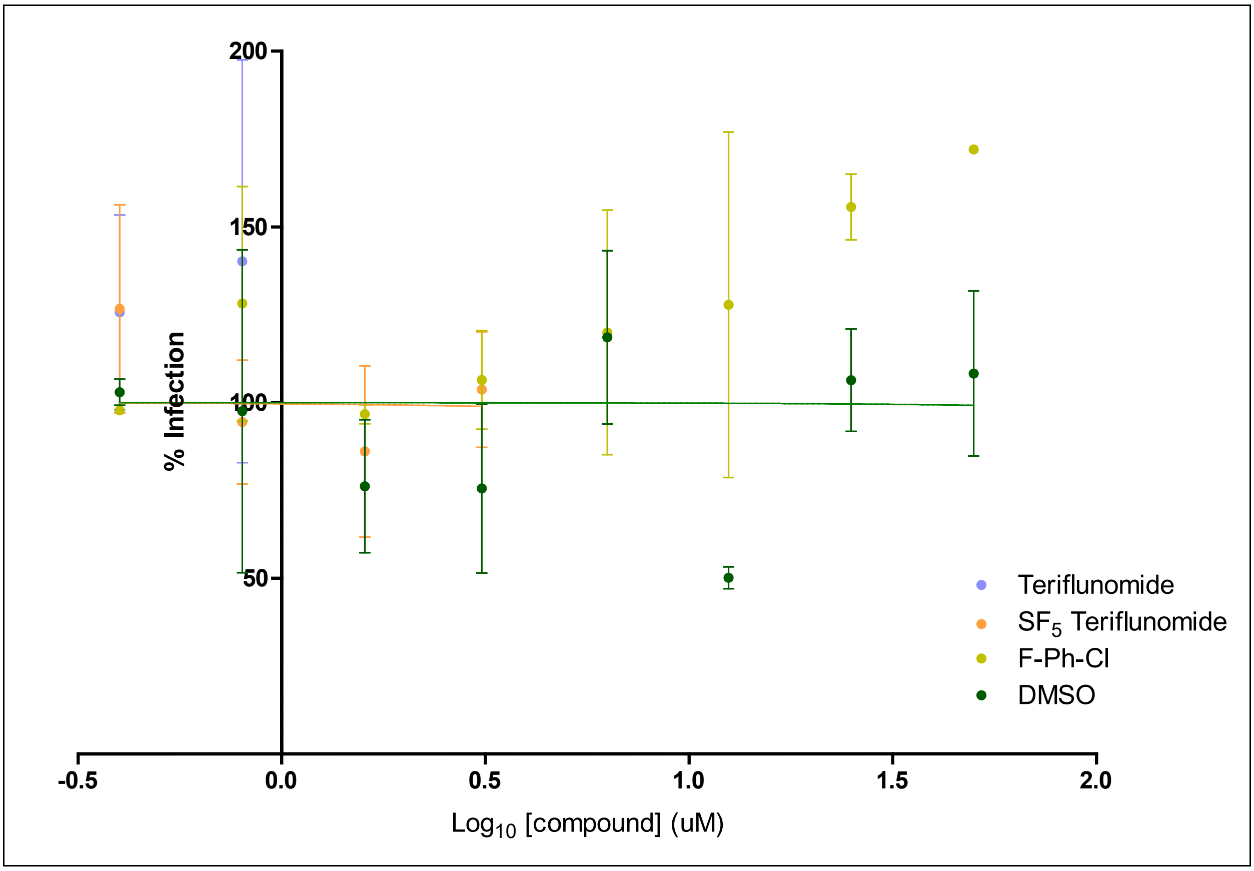

Figure S2: Effect of teriflunomide compounds on SARS CoV2 infection of HEK293T/17

The HEK293T/17 cell line was chosen for our in-house assays after initial testing of a number of human lines for their permissiveness to pseudotyped SARS CoV2 infection; it is possible that a different target cell line would be less sensitive to the effect of the teriflunomide compounds, but in order for the infection inhibition to be studied, the target line should primarily work well with the proposed virus format.

The cytotoxicity assay results showed that the tested compounds are not ideal candidate SARS CoV2 antiviral agents. Both teriflunomide and SF_5-teriflunomide had a strong cytostatic effect on the target cell line and no measurable inhibitory effect at non-toxic concentrations. Conversely, F-Ph-Cl was significantly less cytostatic but also appears to enhance the SARS

CoV2 infection of the target cells. Examination of the DMSO control data showed that the toxicity was not due to the percentage of DMSO present in the compound solutions.

M.S. Methods. The methods for dose response studies has been reported T. R. Malla, et al., *Chem. Commun.* **2021**, *57*, 1430–1433.. In brief, compounds were dry dispensed (100 µM top concentration, 11 point 3 fold dilution series) using an Echo 550 acoustic dispenser (LabCyte). A multidrop Combi liquid handling robot was used to dispense Mpro (0.30 µM, 25 µL/well) across the plate. A TSAVLQ/SGFRK-NH_2 solution (4 µM, 25 µL/wel)) was added following 15 minutes incubation with the compounds at ambient temperature. Reactions were incubated (10 minutes), then quenched by addition of 10% (v/v) aqueous formic acid (5 µL/well). Data were extracted and processed as described for the fluorescence-based Mpro assays. IC_{50} curves were generated using non-linear regression and normalized with respect to the positive and negative inhibition controls (GraphPad Prism 8) and CDD. IC_{50}-values are reported as the mean of technical duplicates (n = 2; mean ± SD).

Scanned spectra

[3-(Pentafluoro-λ^6-sulfanyl)phenyl](piperidin-1-yl)methanone (3a)

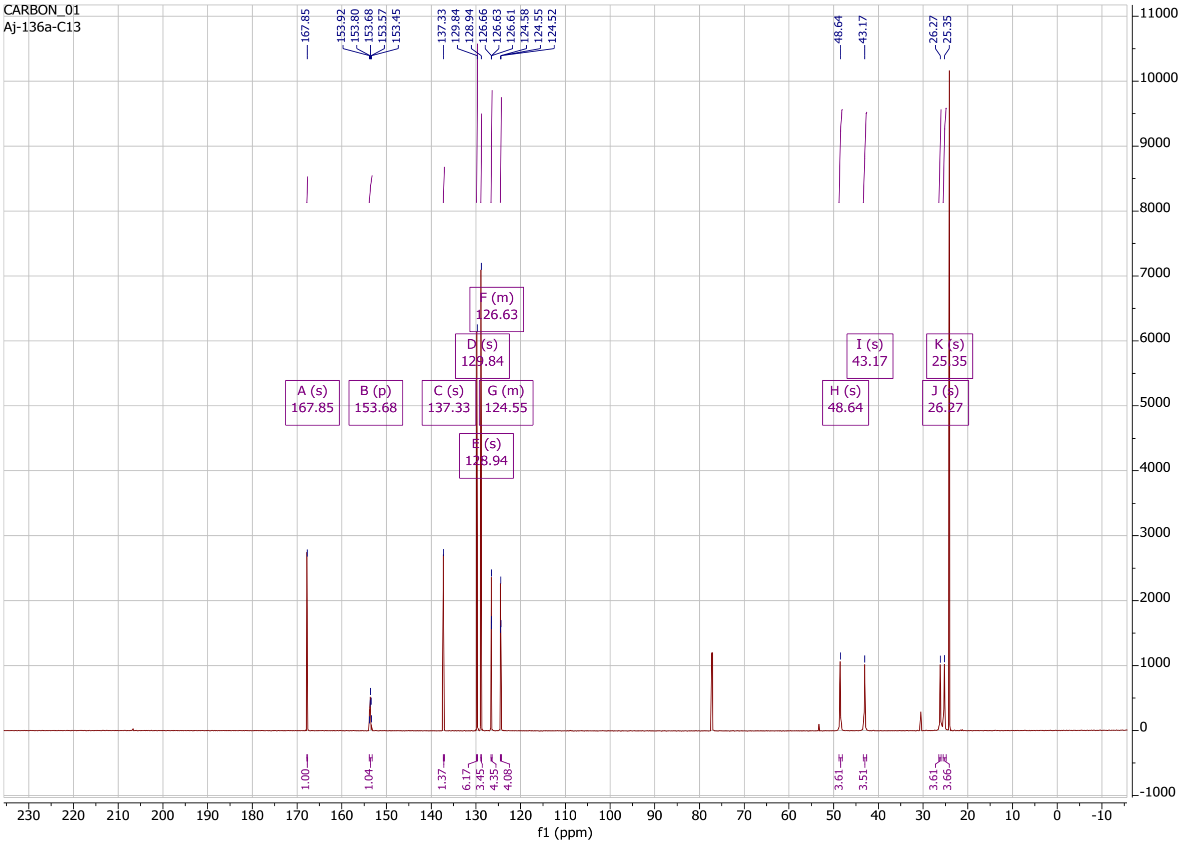

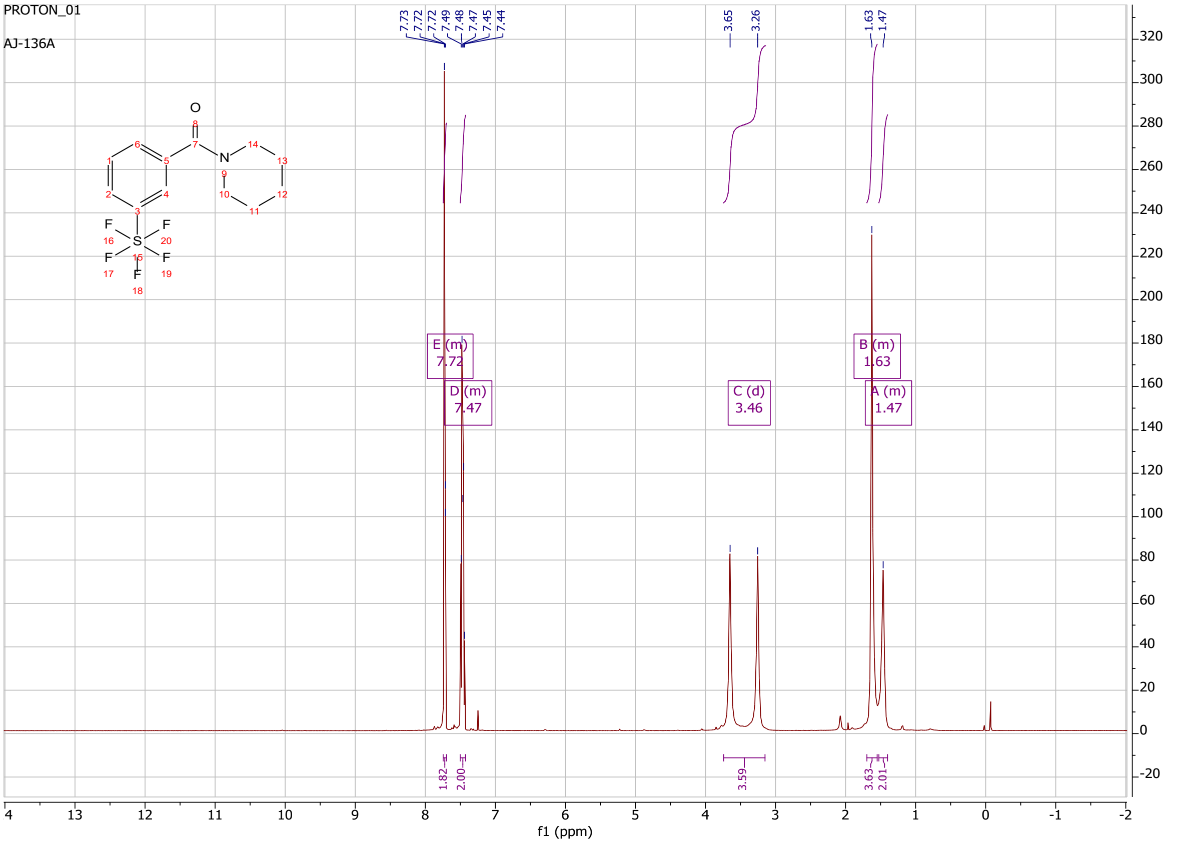

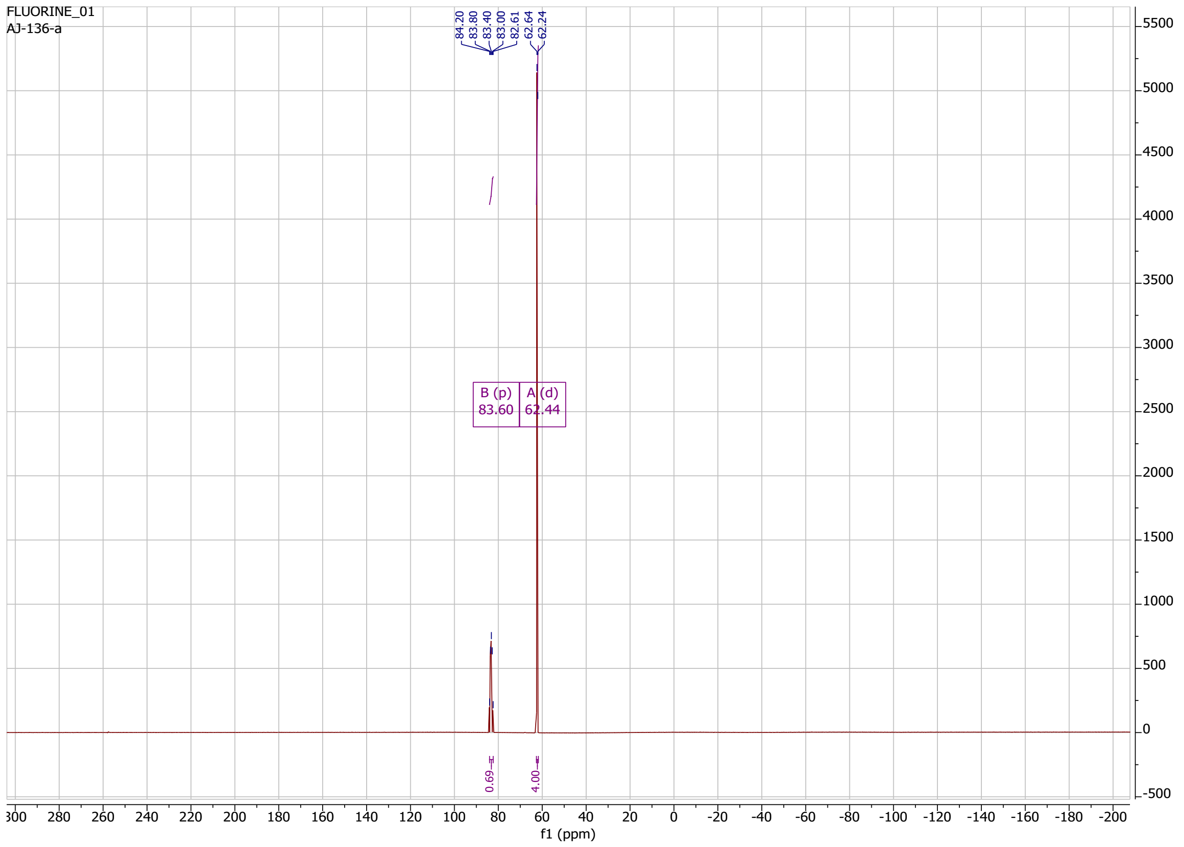
FLUORINE_01
AJ-136-a
84.20
83.80
83.40
83.00
82.61
62.64
62.24
B (p)
83.60
A (d)
62.44
0.69
4.00
f1 (ppm)

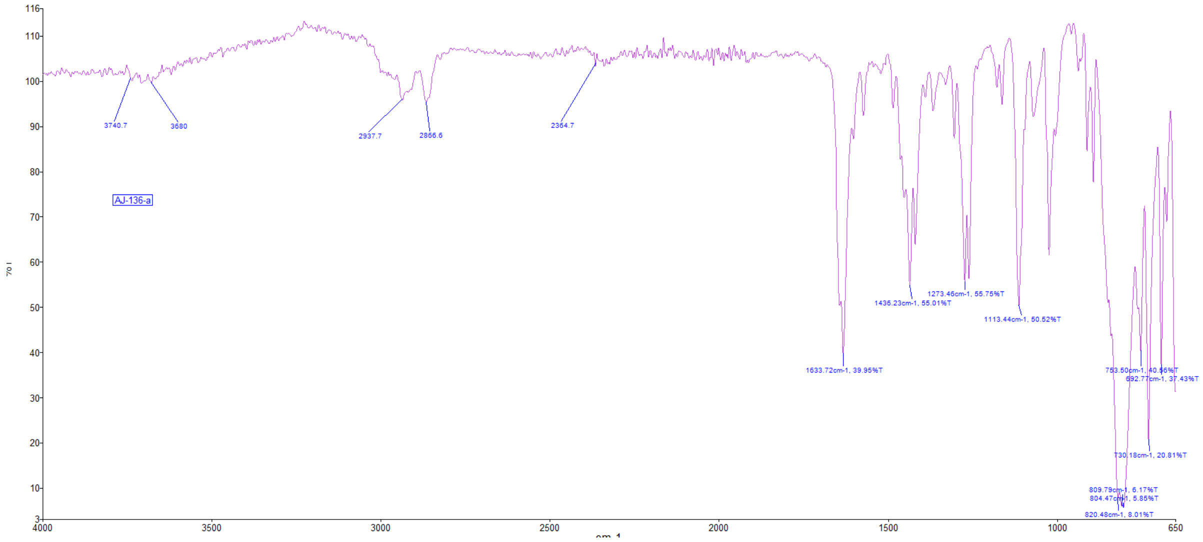
AJ-136-a
3740.7
3680
2937.7
2866.6
2364.7
1633.72cm-1, 39.95%T
1436.23cm-1, 55.01%T
1273.46cm-1, 55.75%T
1113.44cm-1, 50.52%T
753.50cm-1, 40.56%T
692.77cm-1, 37.43%T
730.18cm-1, 20.81%T
809.79cm-1, 6.17%T
804.47cm-1, 5.85%T
820.48cm-1, 8.01%T

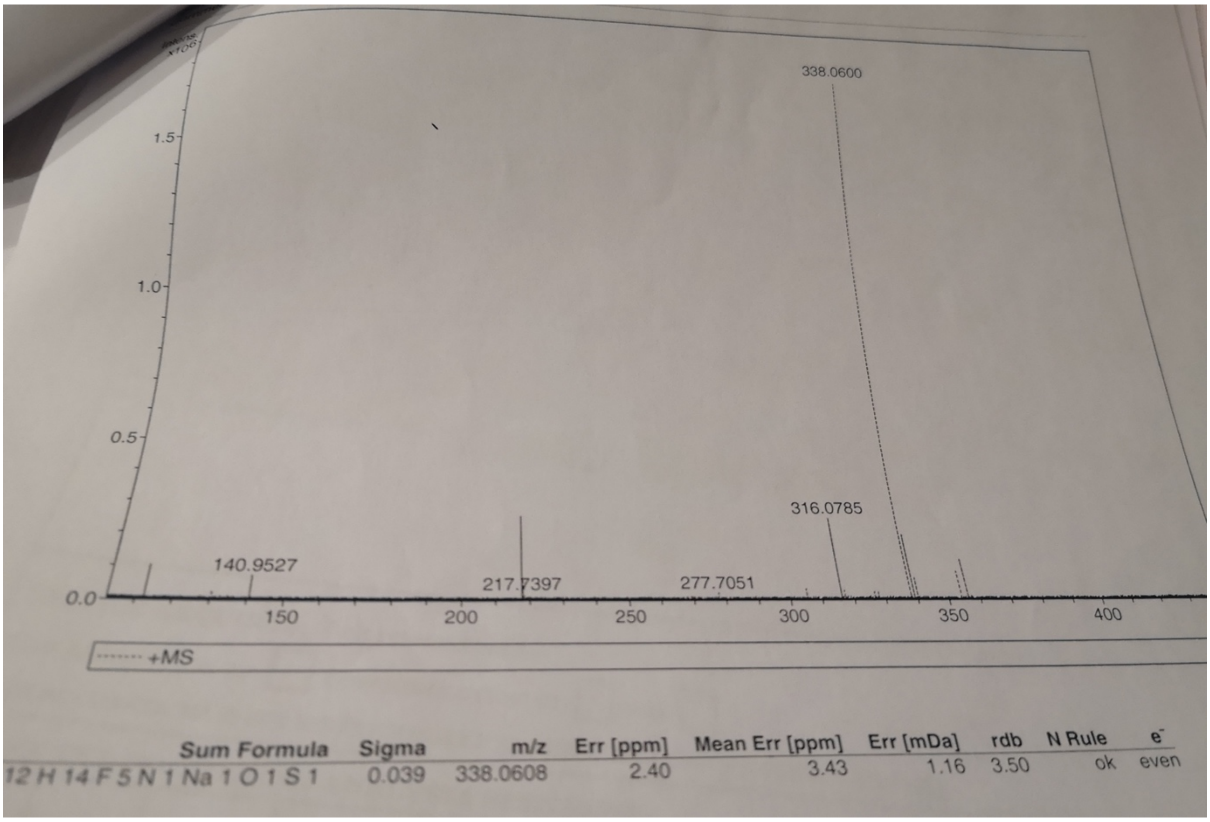

(3-(Pentafluoro-λ^6-sulfanyl)phenyl](4-methylpiperazin-1-yl)methanone (3b)

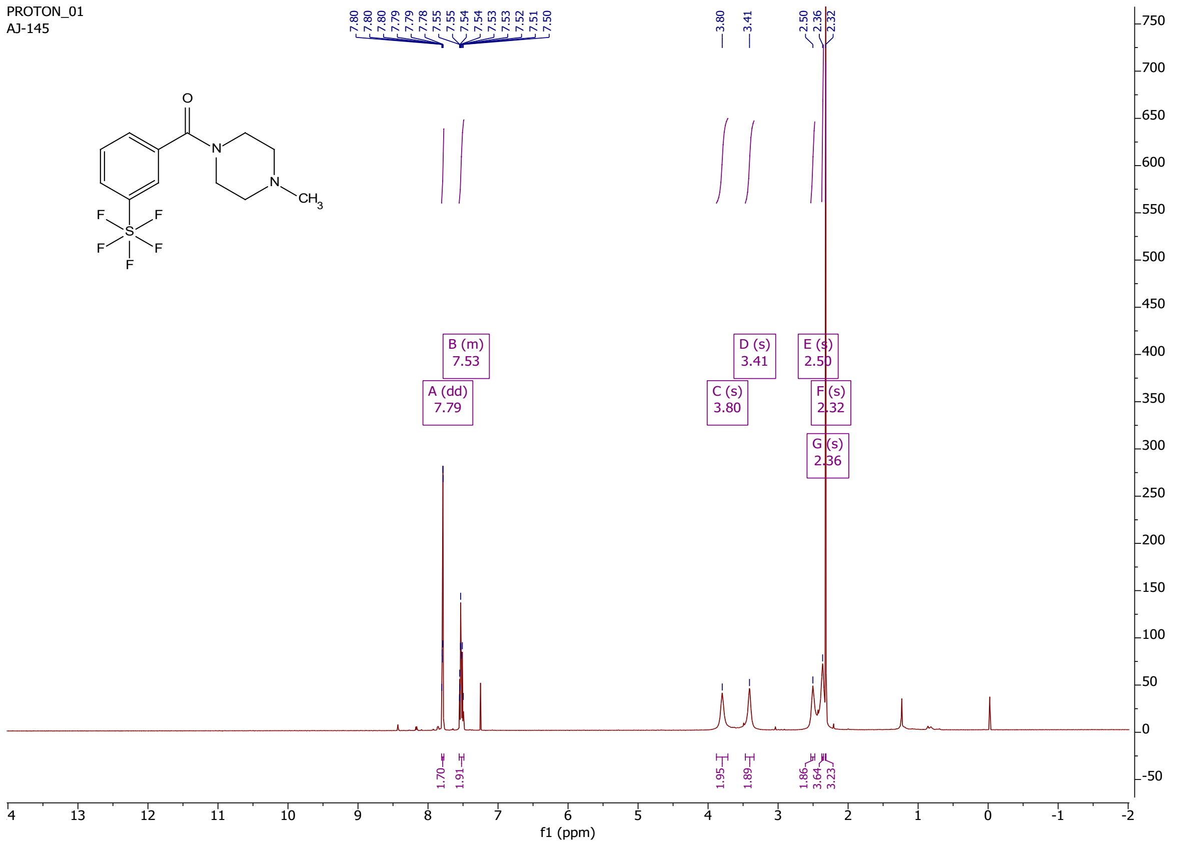

PROTON_01
AJ-145
A (dd) 7.79
B (m) 7.53
C (s) 3.80
D (s) 3.41
E (s) 2.50
F (s) 2.32
G (s) 2.36
f1 (ppm)

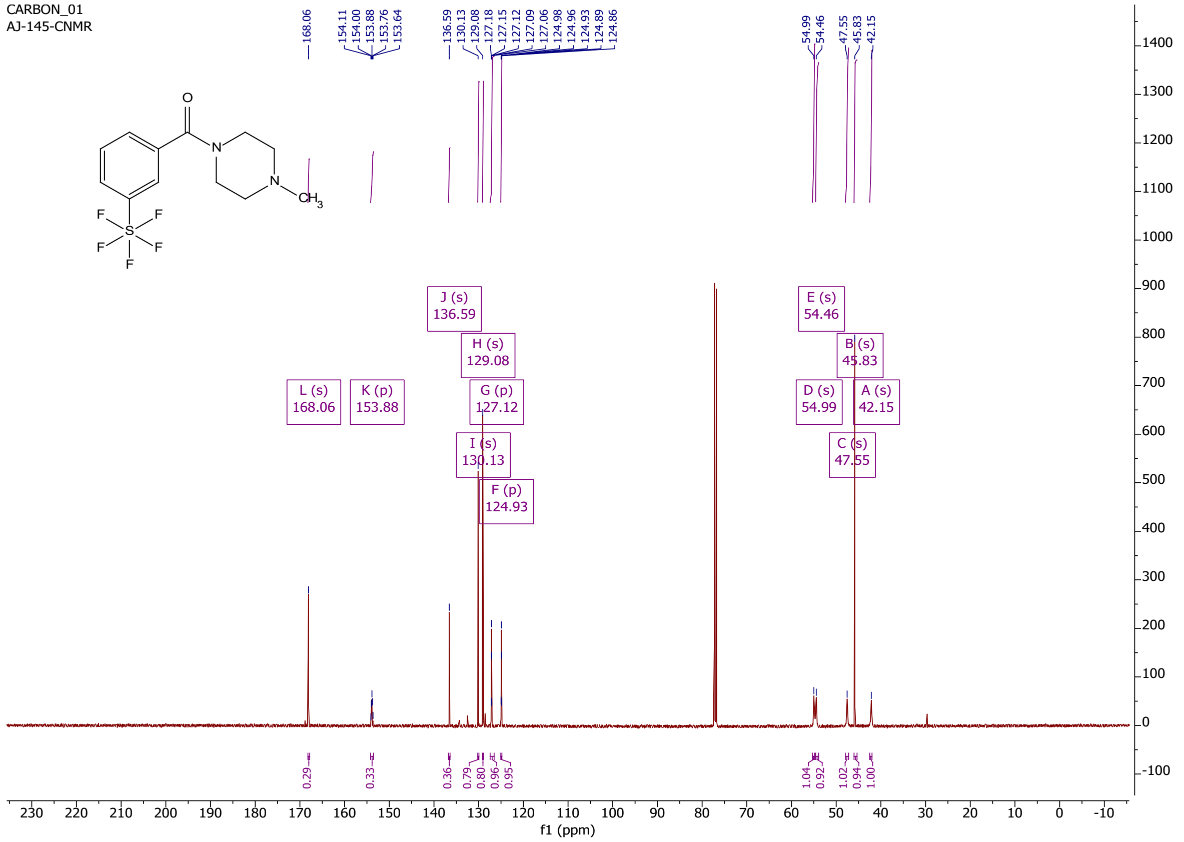

CARBON_01
AJ-145-CNMR
L (s) 168.06
K (p) 153.88
J (s) 136.59
I (s) 130.13
H (s) 129.08
G (p) 127.12
F (p) 124.93
E (s) 54.46
D (s) 54.99
C (s) 47.55
B (s) 45.83
A (s) 42.15
f1 (ppm)

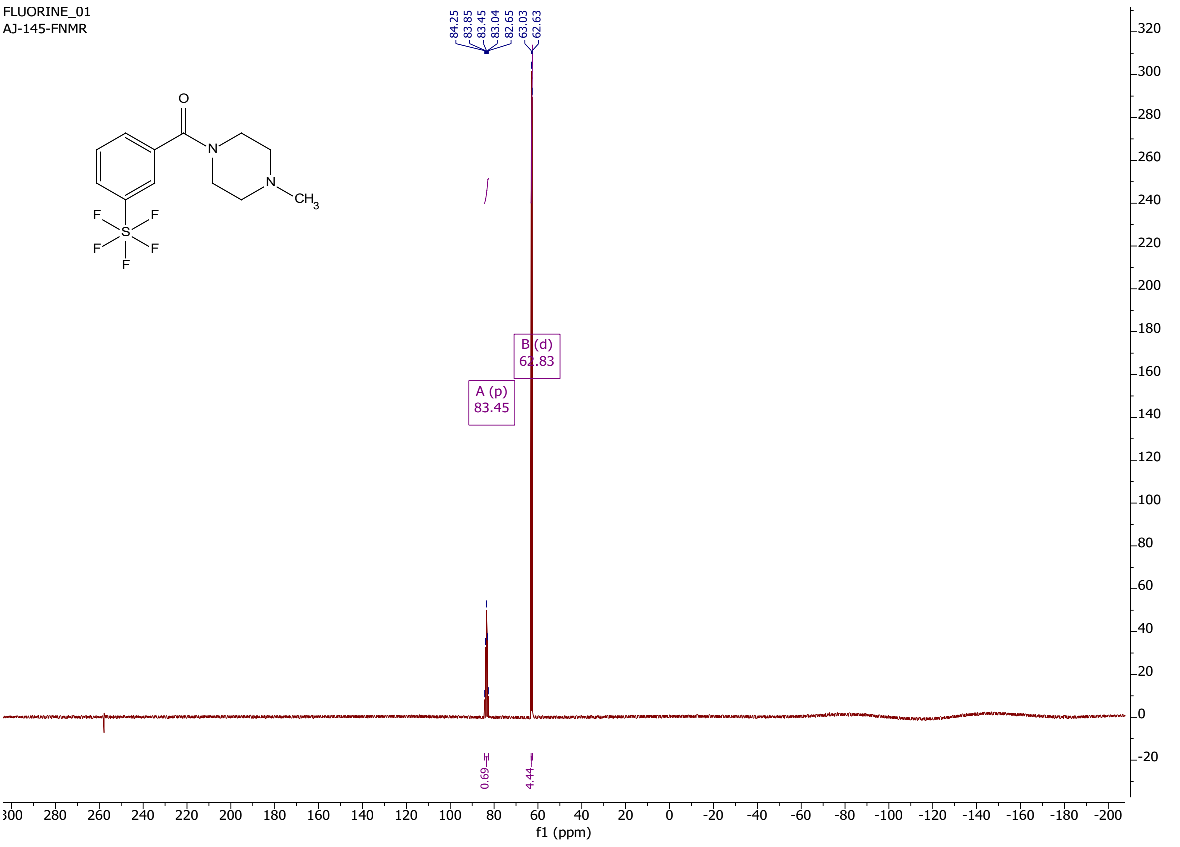
FLUORINE_01
AJ-145-FNMR
84.25
83.85
83.45
83.04
82.65
63.03
62.63
B (d)
62.83
A (p)
83.45
0.69
4.44
f1 (ppm)

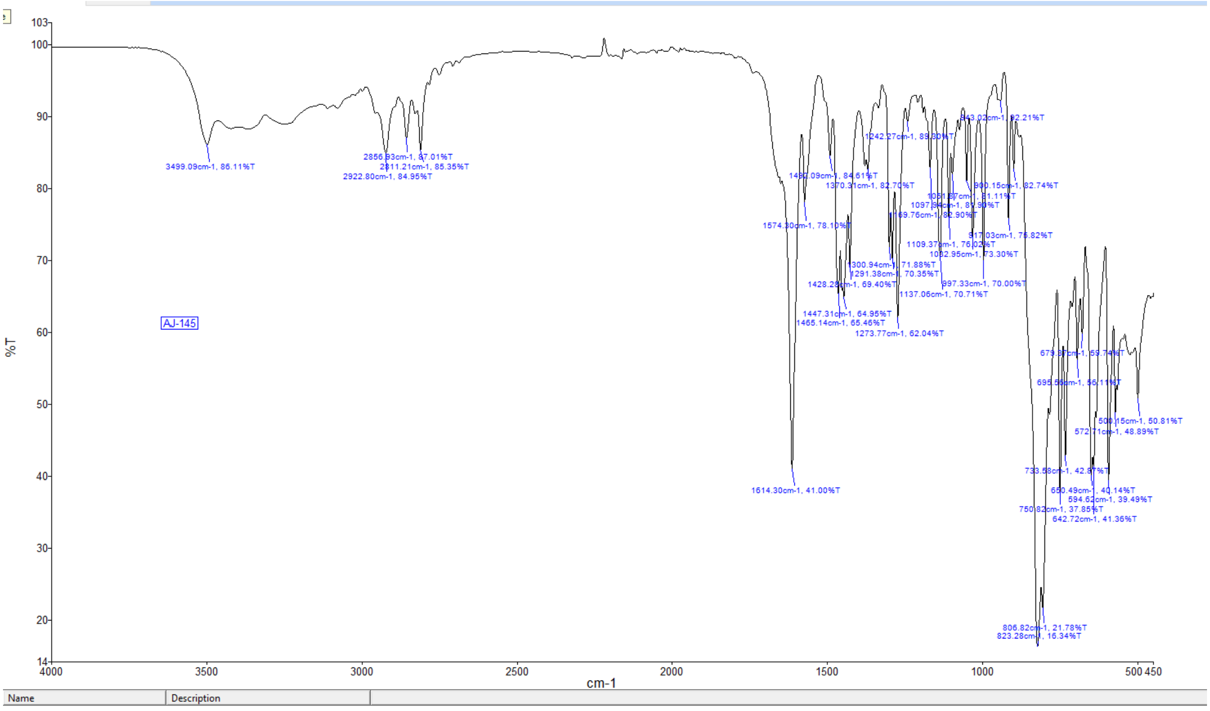
AJ-145
%T
cm-1
3499.09cm-1, 86.11%T
2922.80cm-1, 84.95%T
1614.30cm-1, 41.00%T
1273.77cm-1, 62.04%T
823.28cm-1, 16.34%T
806.82cm-1, 21.78%T
750.82cm-1, 37.85%T
Name
Description

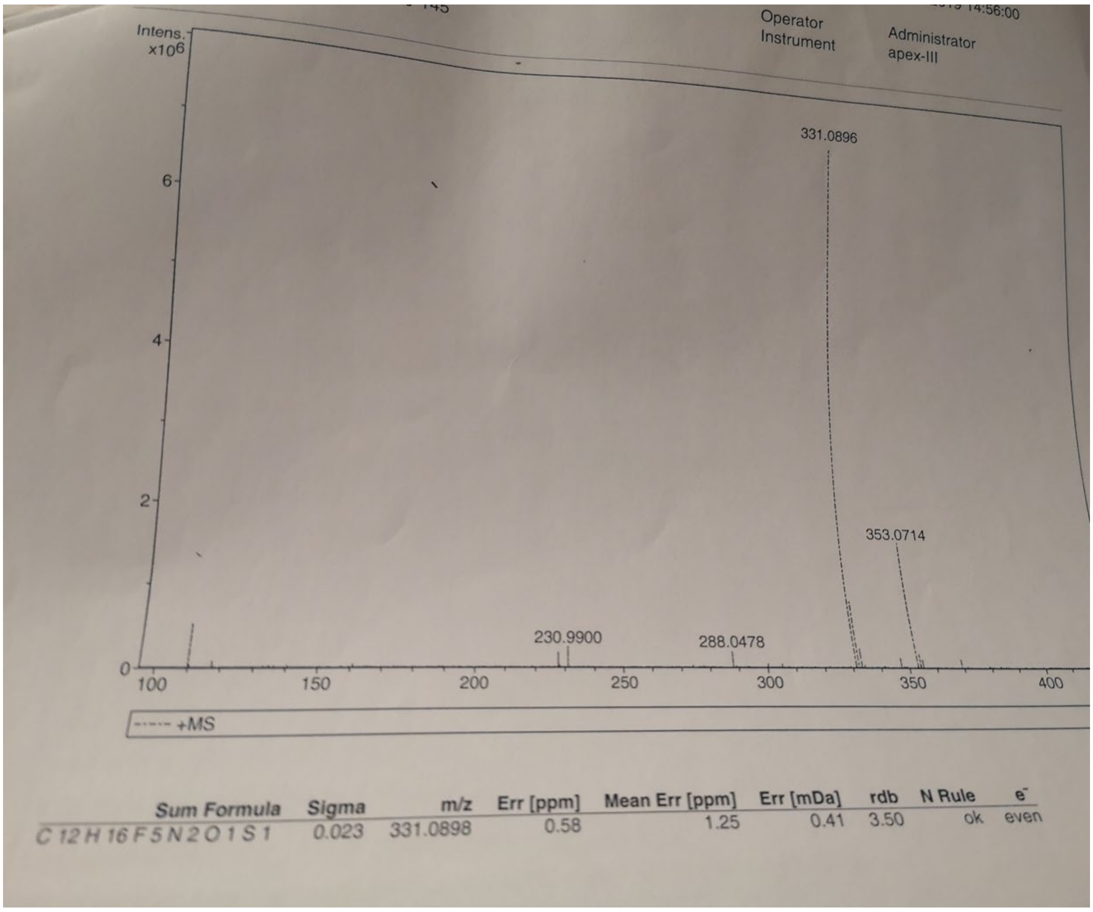

[3-(Pentafluoro-λ^6-sulfanyl)phenyl](morpholino)methanone (3c)

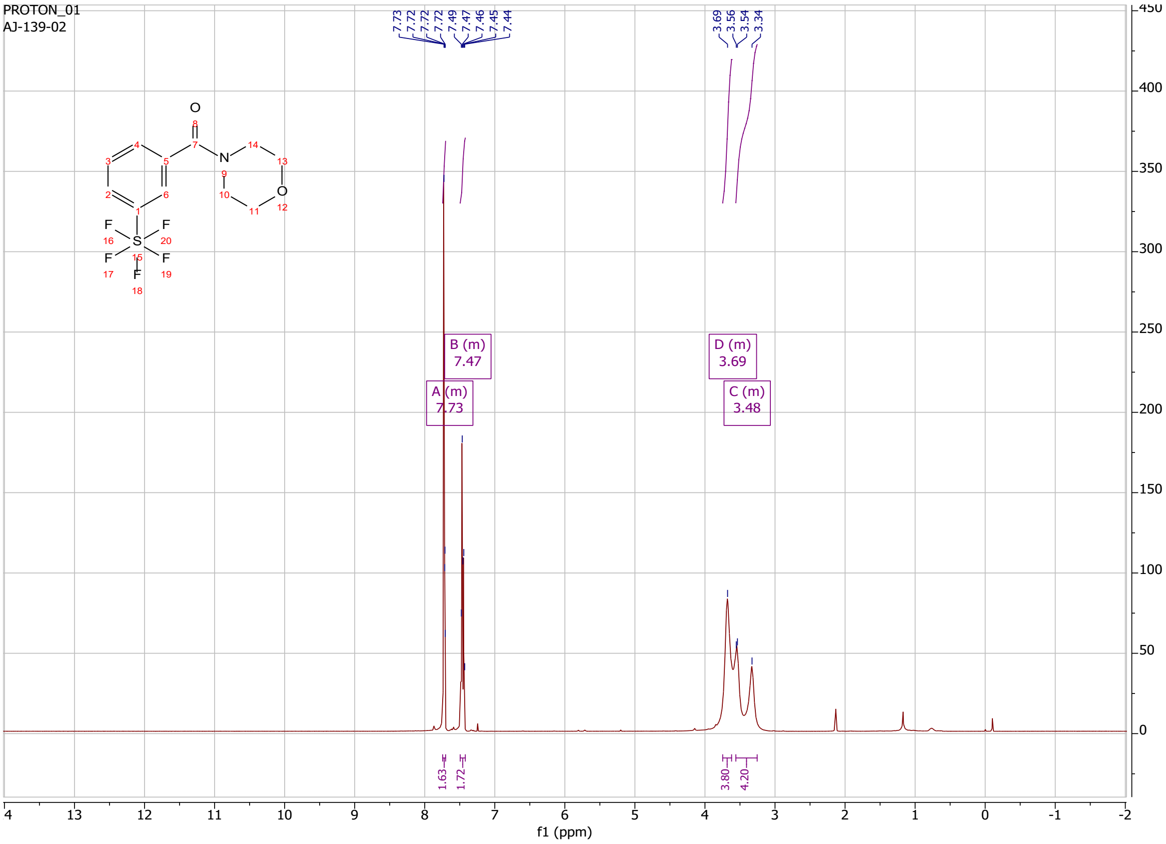

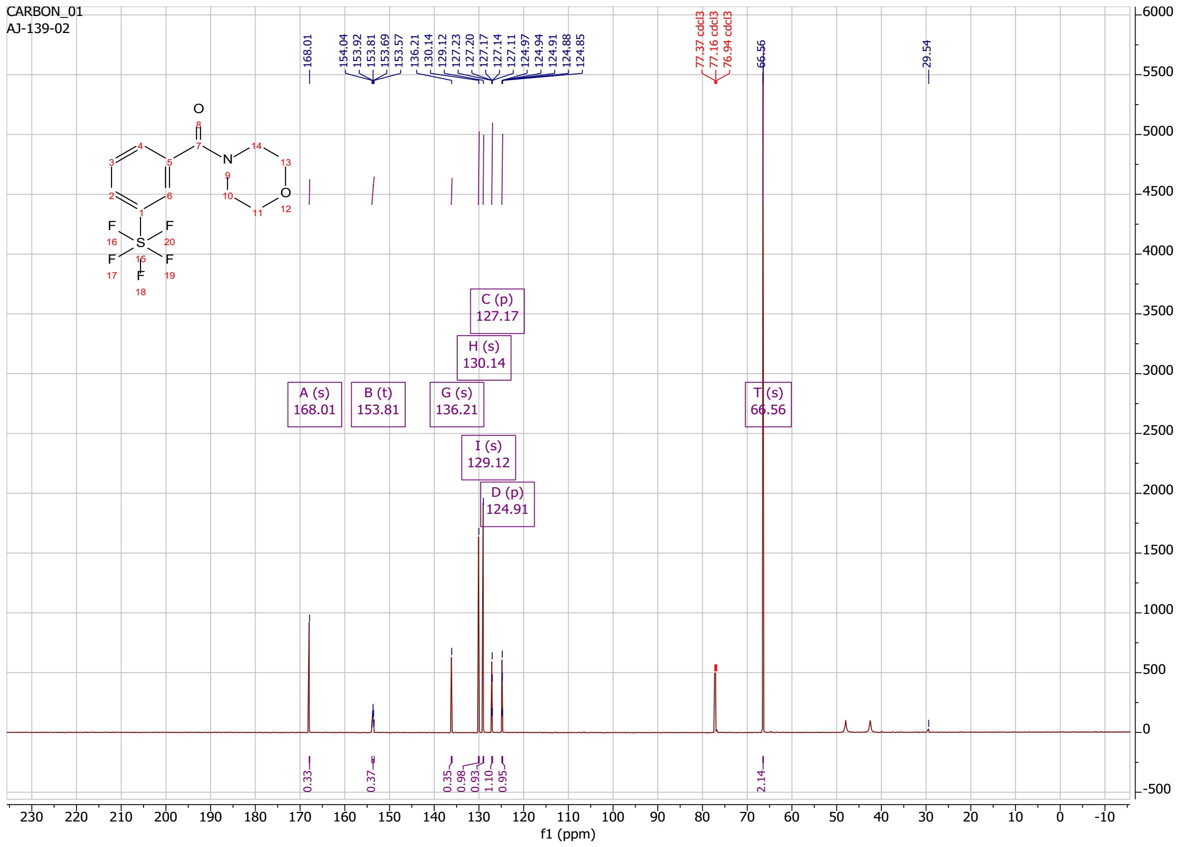
CARBON_01
AJ-139-02
168.01
154.04
153.92
153.81
153.69
153.57
136.21
130.14
129.12
127.23
127.20
127.17
127.14
127.11
124.97
124.94
124.91
124.88
124.85
77.37 cdcl3
77.16 cdcl3
76.94 cdcl3
66.56
29.54
A (s) 168.01
B (t) 153.81
G (s) 136.21
H (s) 130.14
I (s) 129.12
C (p) 127.17
D (p) 124.91
T (s) 66.56
0.33
0.37
0.35
0.98
0.93
1.10
0.95
2.14
f1 (ppm)

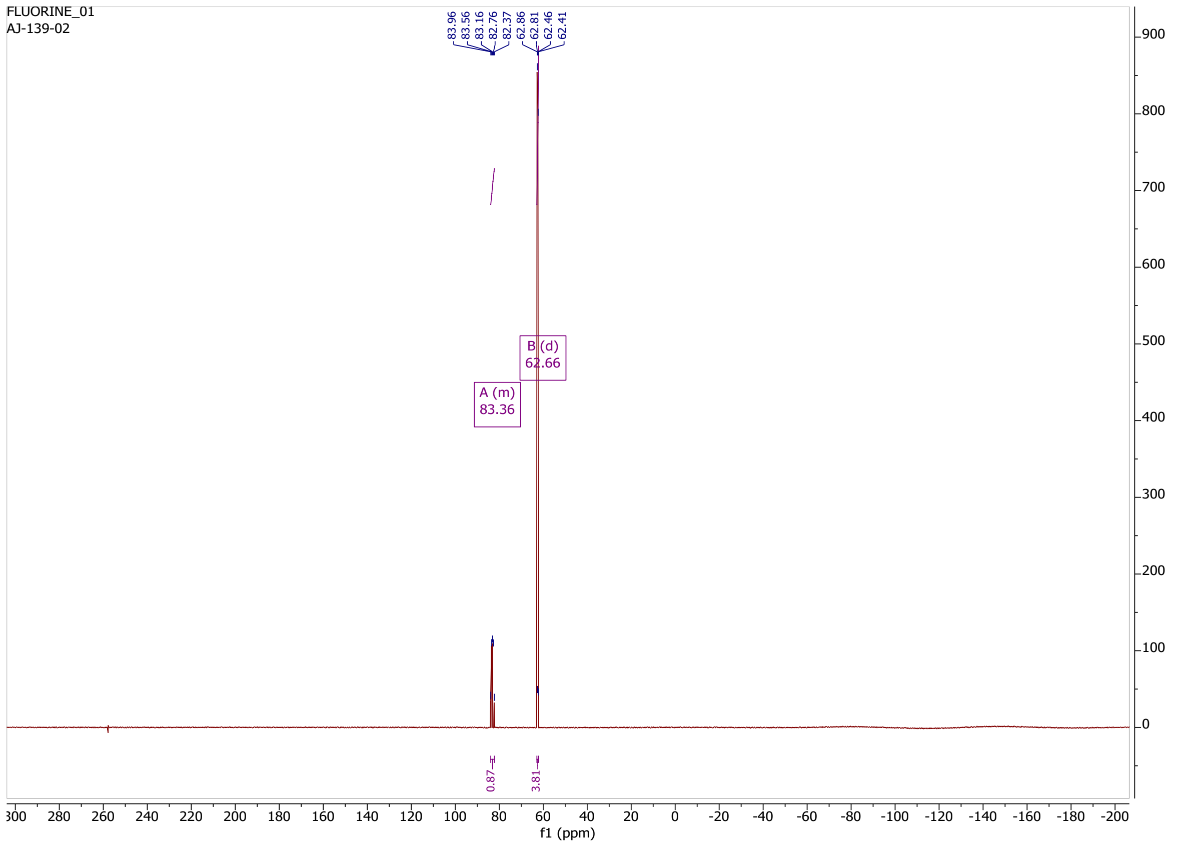
FLUORINE_01
AJ-139-02
83.96
83.56
83.16
82.76
82.37
62.86
62.81
62.46
62.41
A (m) 83.36
B (d) 62.66
0.87
3.81
f1 (ppm)

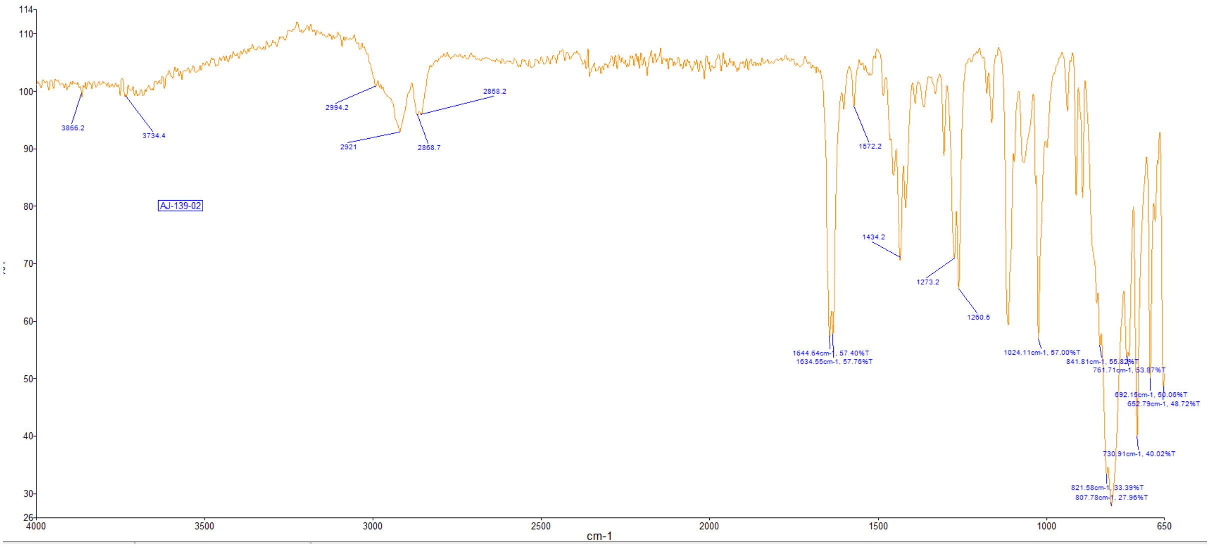

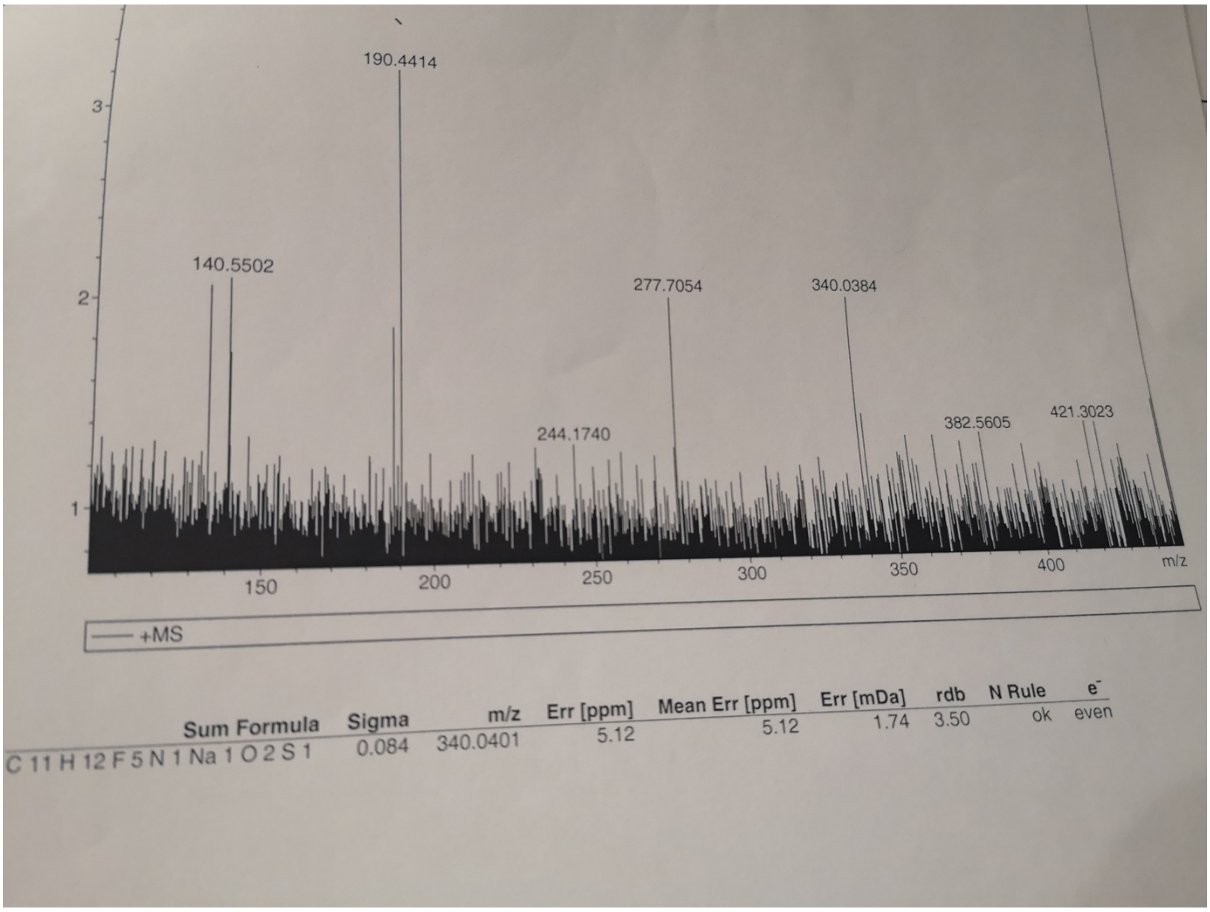

***tert*-Butyl 4-(3-pentafluoro-□^{6}-sulfanyl benzoyl)piperazine-1-carboxylate (3d)**

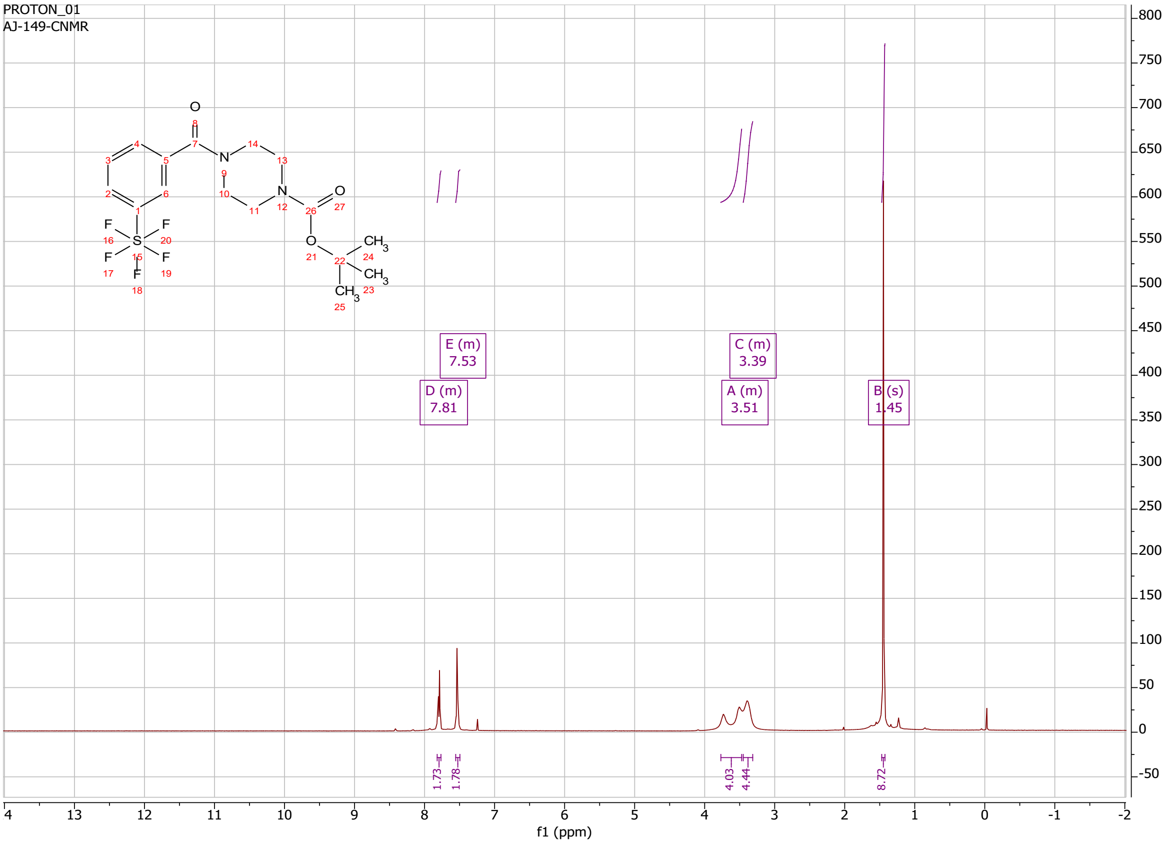

PROTON_01
AJ-149-CNMR
E (m) 7.53
D (m) 7.81
C (m) 3.39
A (m) 3.51
B (s) 1.45
1.73
1.78
4.03
4.44
8.72
f1 (ppm)

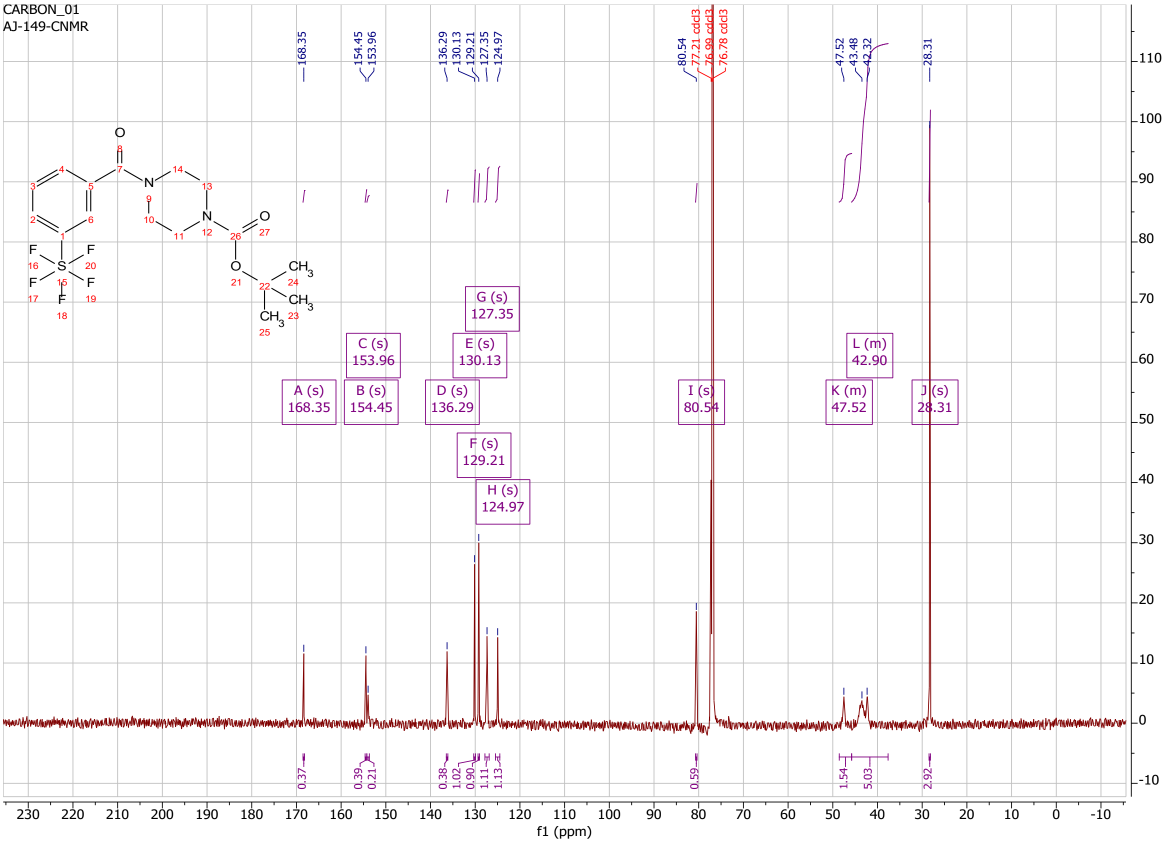

CARBON_01
AJ-149-CNMR
168.35
154.45
153.96
136.29
130.13
129.21
127.35
124.97
80.54
77.21 cdcl3
76.99 cdcl3
76.78 cdcl3
47.52
43.48
42.32
28.31
A (s) 168.35
B (s) 154.45
C (s) 153.96
D (s) 136.29
E (s) 130.13
F (s) 129.21
G (s) 127.35
H (s) 124.97
I (s) 80.54
J (s) 28.31
K (m) 47.52
L (m) 42.90
f1 (ppm)

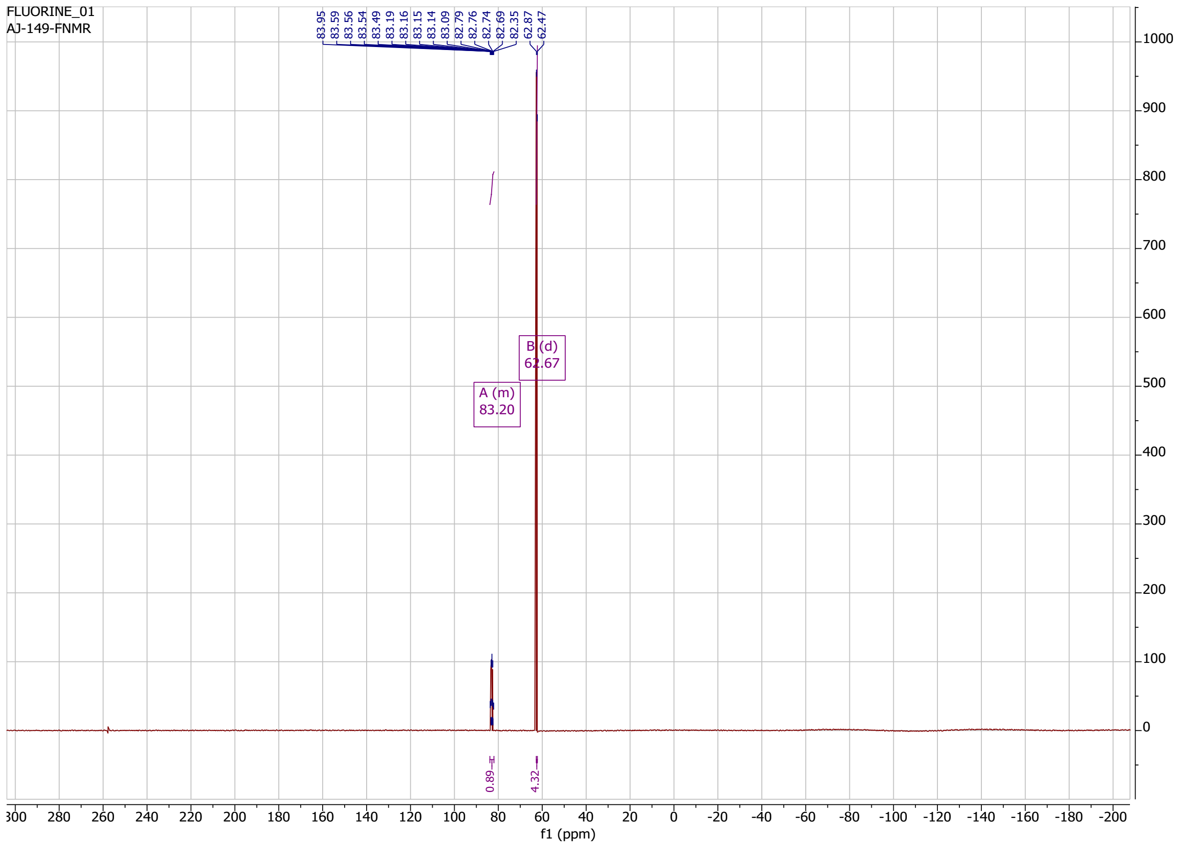

FLUORINE_01
AJ-149-FNMR
A (m)
83.20
B (d)
62.67
f1 (ppm)

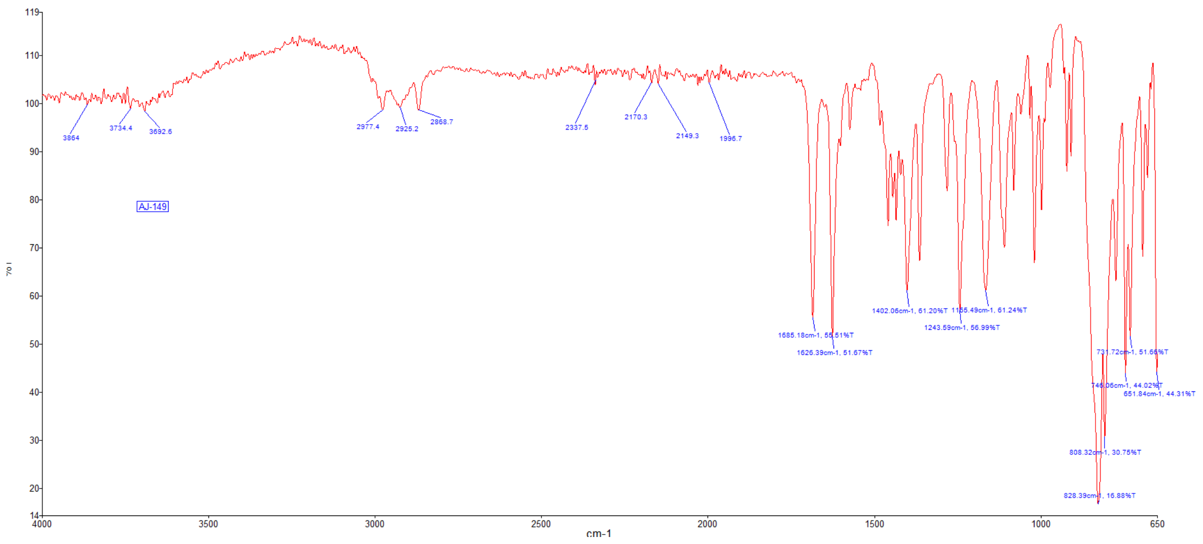

AJ-149
cm-1

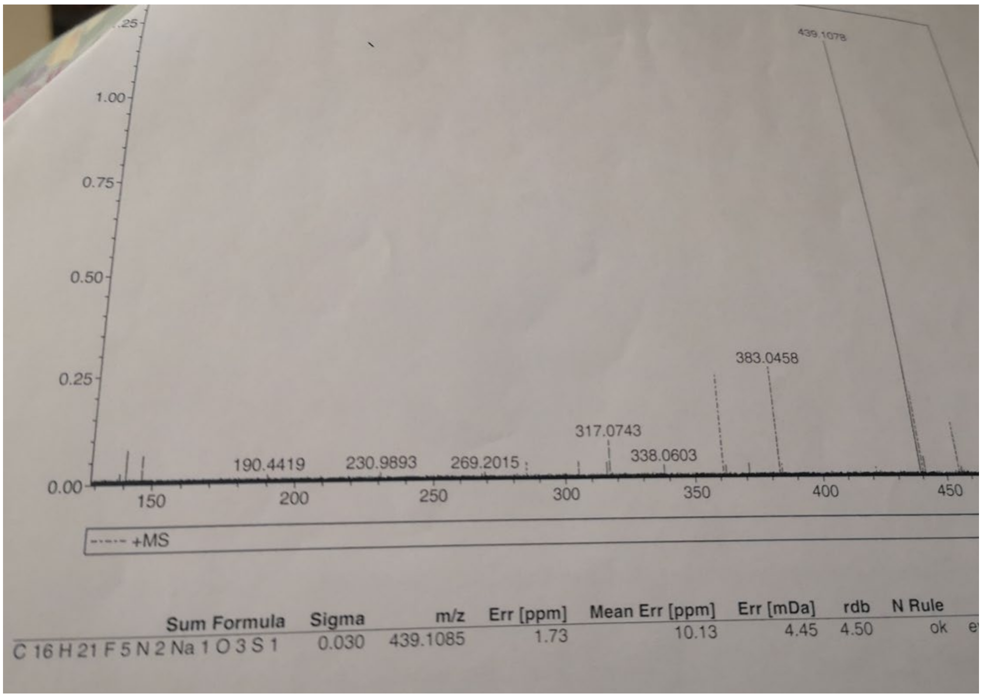

1-[4-[3-(Pentafluoro-λ^6-sulfanyl)benzoyl]piperazin-1-yl]ethanone (3e)

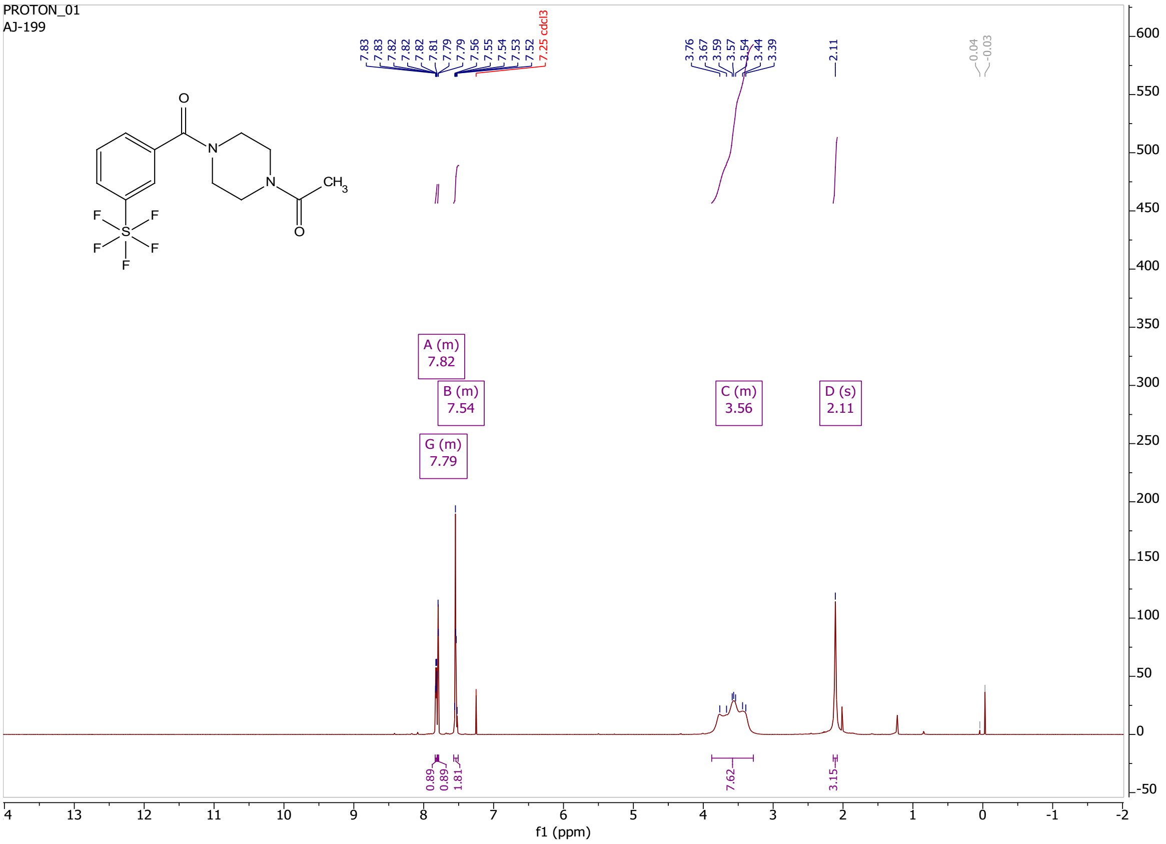

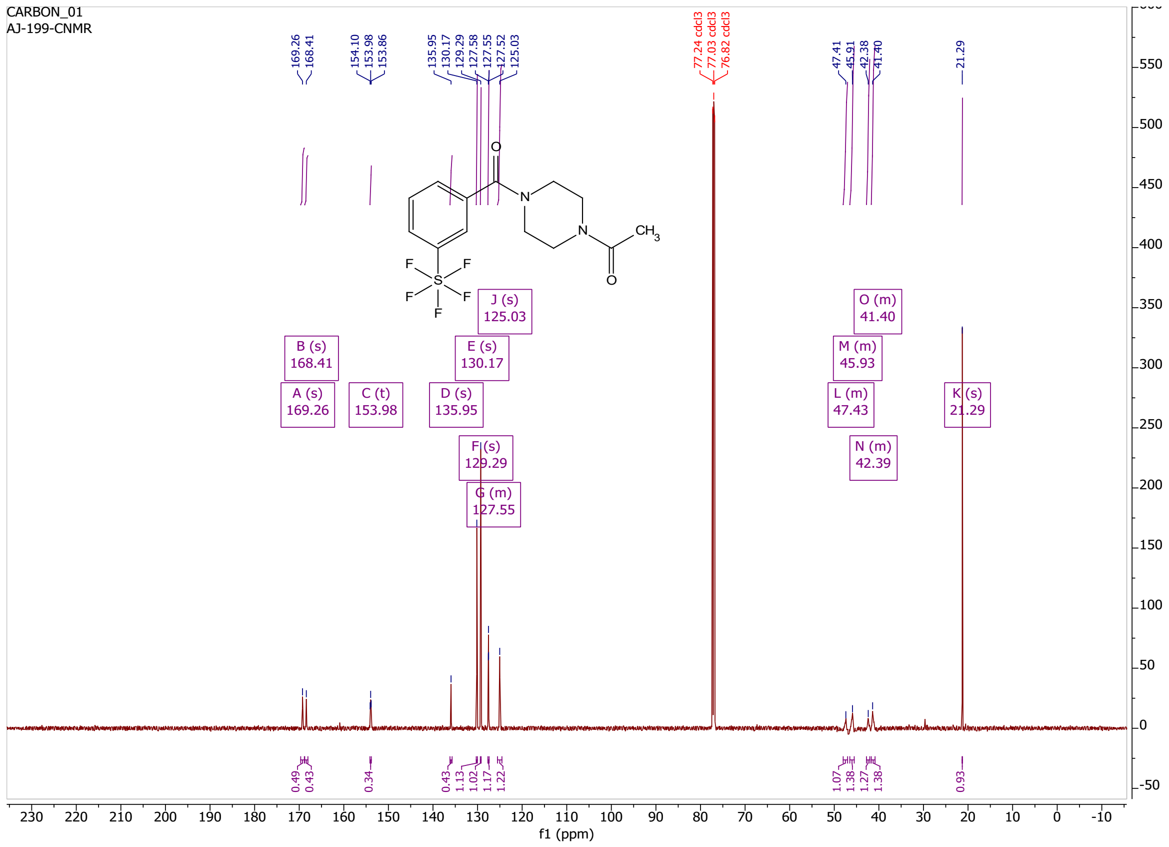
CARBON_01
AJ-199-CNMR
A (s) 169.26
B (s) 168.41
C (t) 153.98
D (s) 135.95
E (s) 130.17
F (s) 129.29
G (m) 127.55
J (s) 125.03
K (s) 21.29
L (m) 47.43
M (m) 45.93
N (m) 42.39
O (m) 41.40
f1 (ppm)

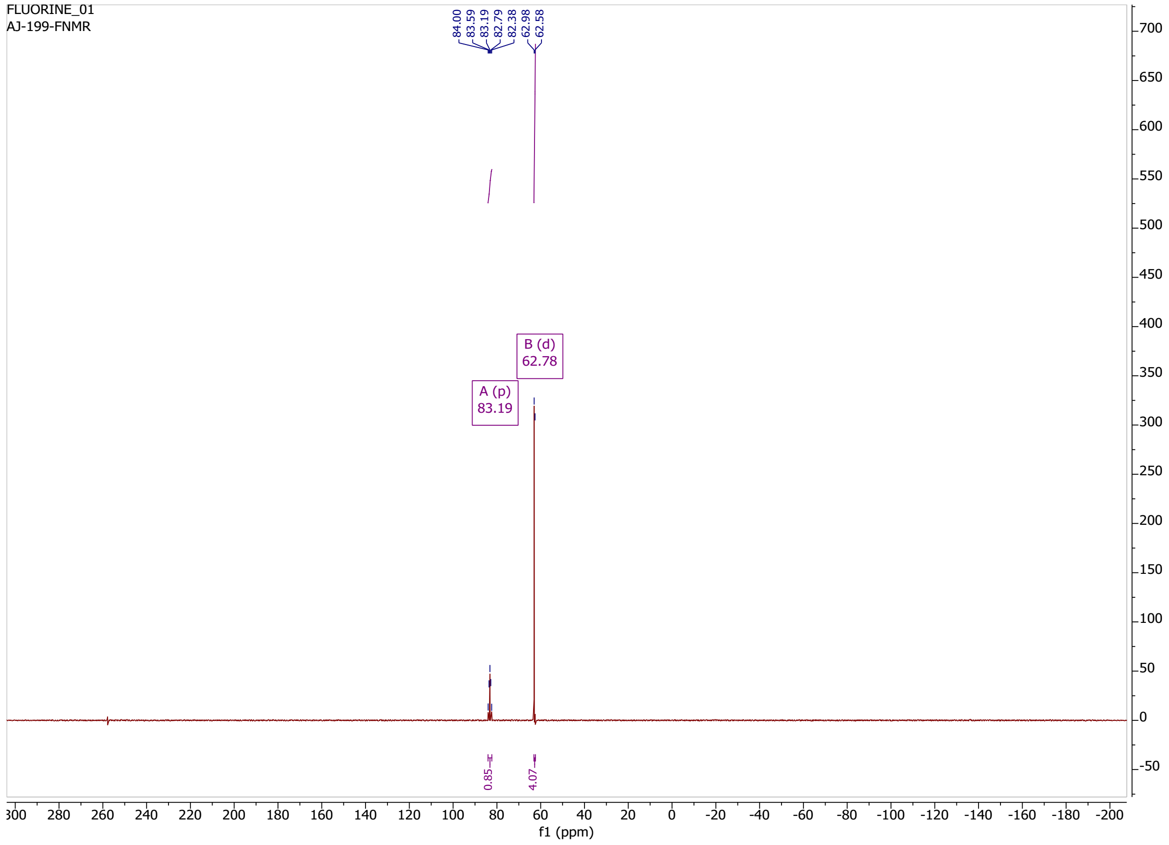
FLUORINE_01
AJ-199-FNMR
A (p) 83.19
B (d) 62.78
f1 (ppm)

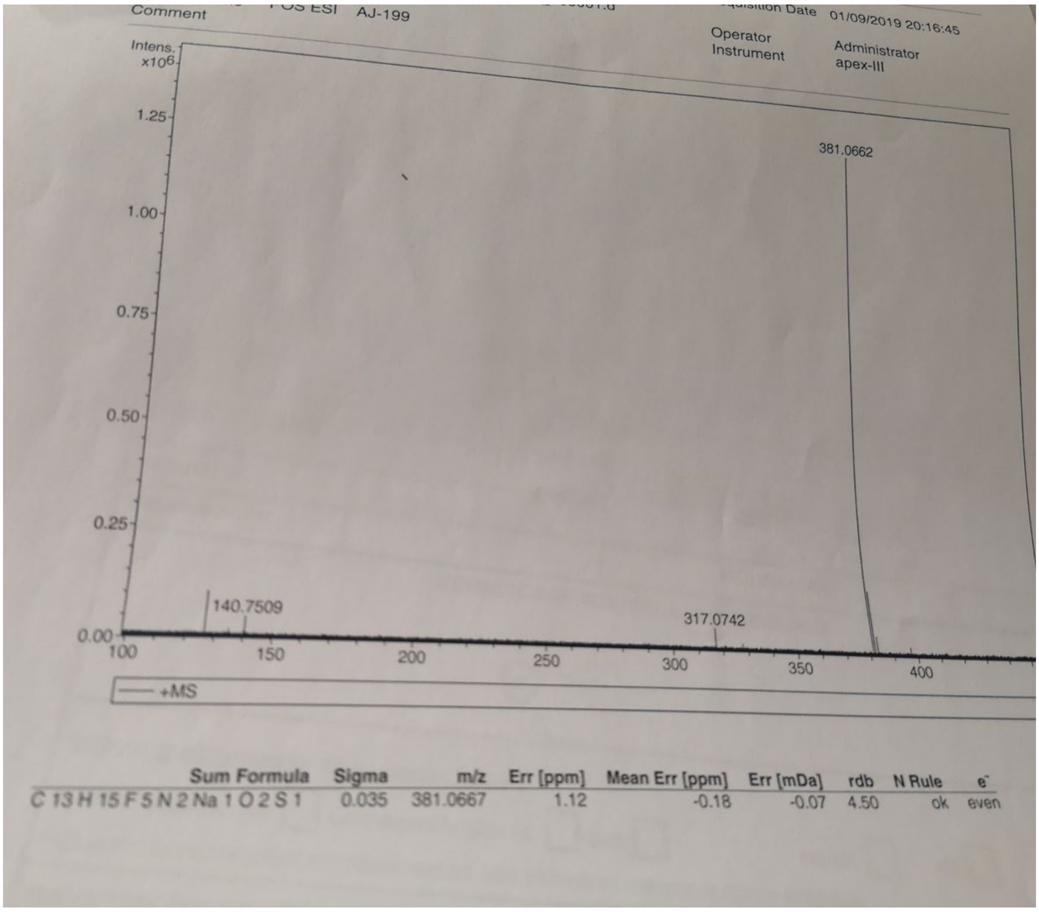

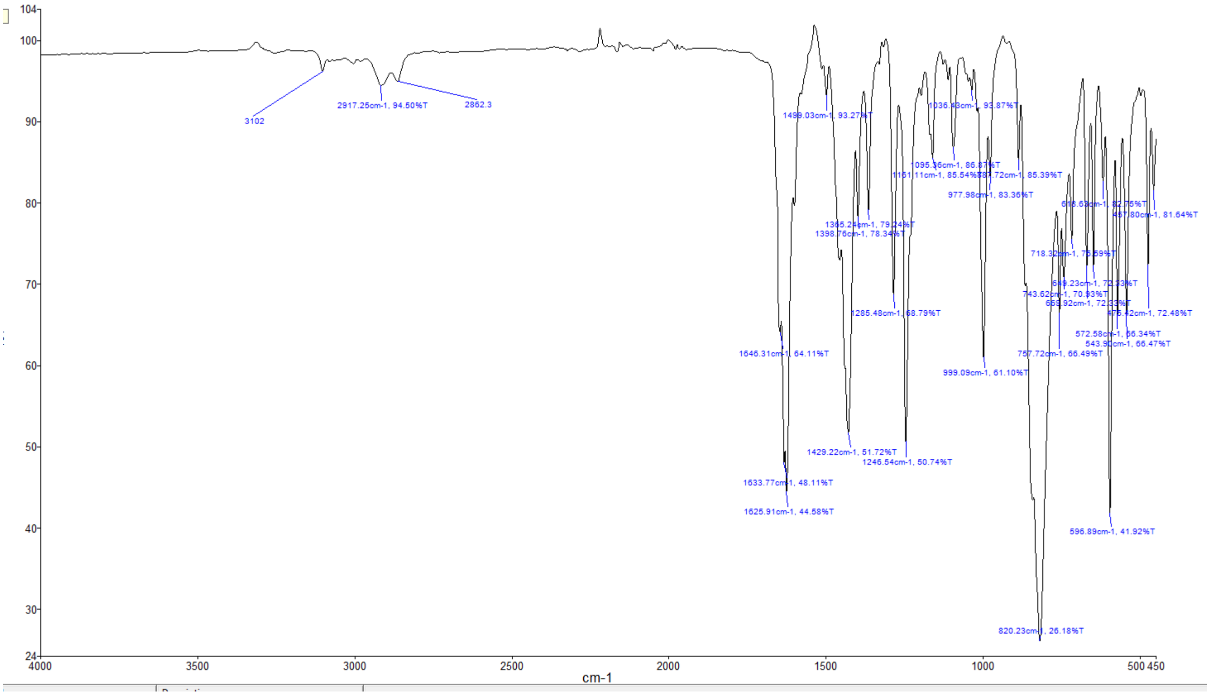

(3-Pentafluorosulfanyl phenyl)(4-(methylsulfonyl)piperazin-1-yl)methanone (3f)

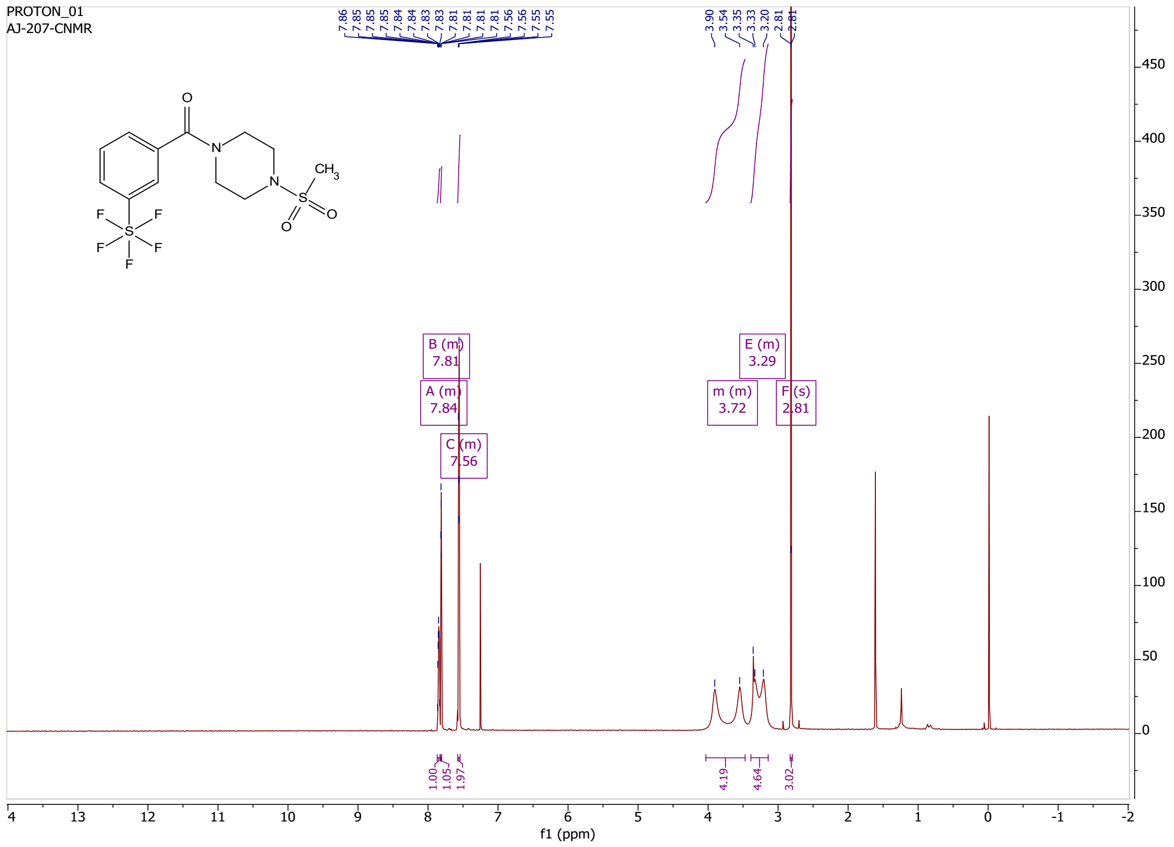
PROTON_01
AJ-207-CNMR
B (m)
7.81
A (m)
7.84
C (m)
7.56
E (m)
3.29
m (m)
3.72
F (s)
2.81
f1 (ppm)

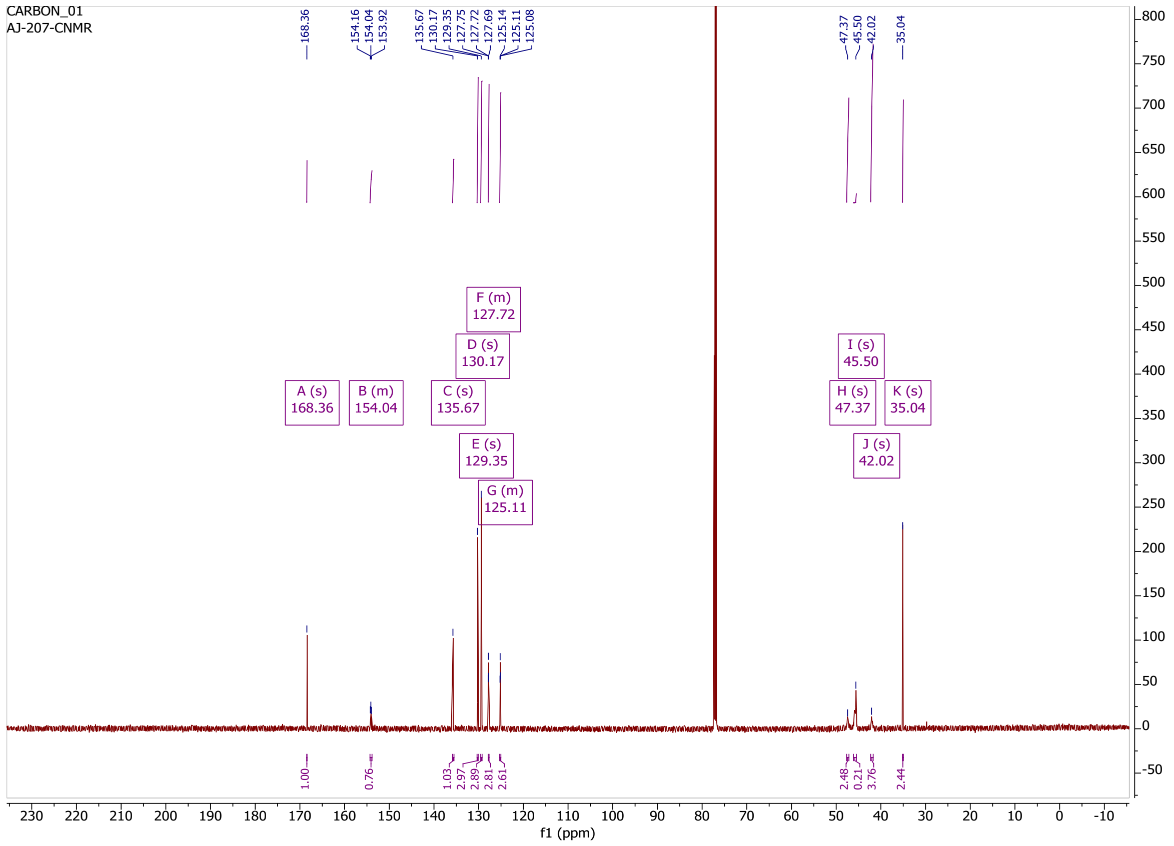
CARBON_01
AJ-207-CNMR
A (s)
168.36
B (m)
154.04
C (s)
135.67
D (s)
130.17
E (s)
129.35
F (m)
127.72
G (m)
125.11
H (s)
47.37
I (s)
45.50
J (s)
42.02
K (s)
35.04
f1 (ppm)

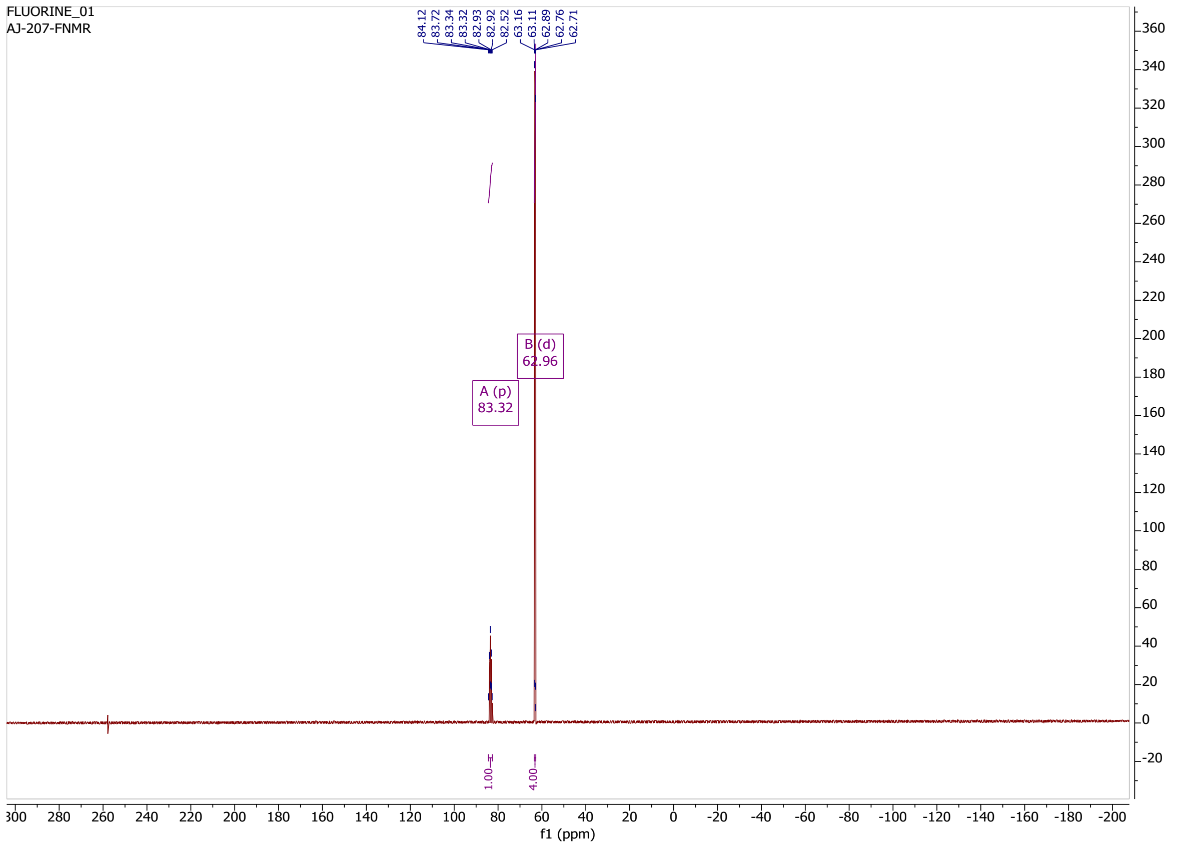
FLUORINE_01
AJ-207-FNMR
84.12
83.72
83.34
83.32
82.93
82.92
82.52
63.16
63.11
62.89
62.76
62.71
B (d)
62.96
A (p)
83.32
1.00
4.00
f1 (ppm)

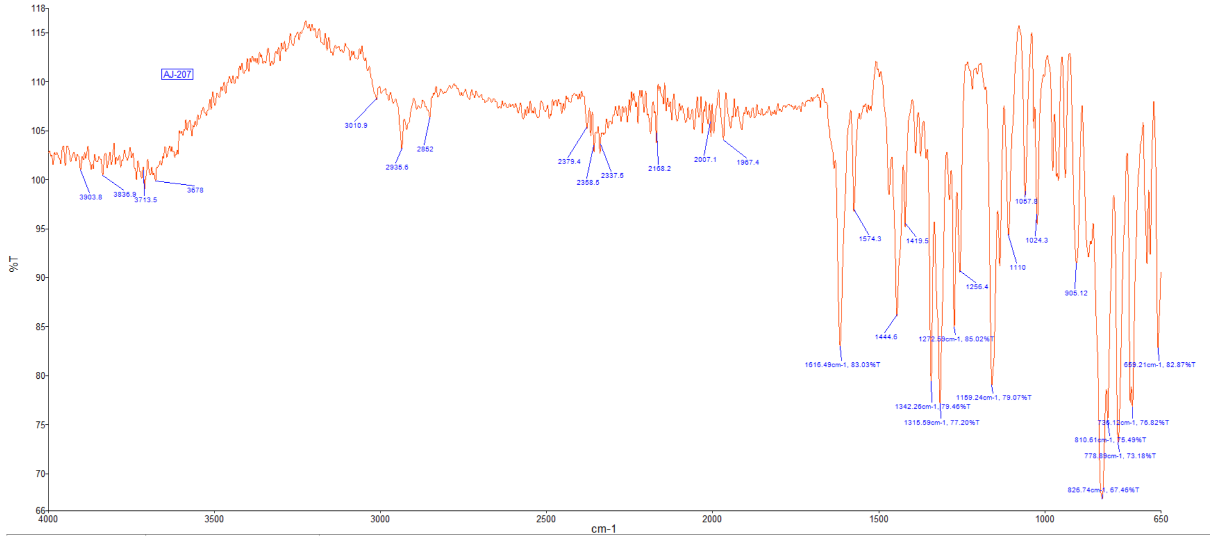
AJ-207
%T
cm-1
3903.8
3836.9
3713.5
3678
3010.9
2935.6
2852
2379.4
2358.5
2337.5
2168.2
2007.1
1967.4
1616.49cm-1, 83.03%T
1574.3
1444.6
1419.5
1342.26cm-1, 79.46%T
1315.59cm-1, 77.20%T
1272.59cm-1, 85.02%T
1256.4
1159.24cm-1, 79.07%T
1110
1057.8
1024.3
905.12
826.74cm-1, 67.46%T
810.61cm-1, 75.49%T
778.89cm-1, 73.18%T
736.12cm-1, 76.82%T
689.21cm-1, 82.87%T

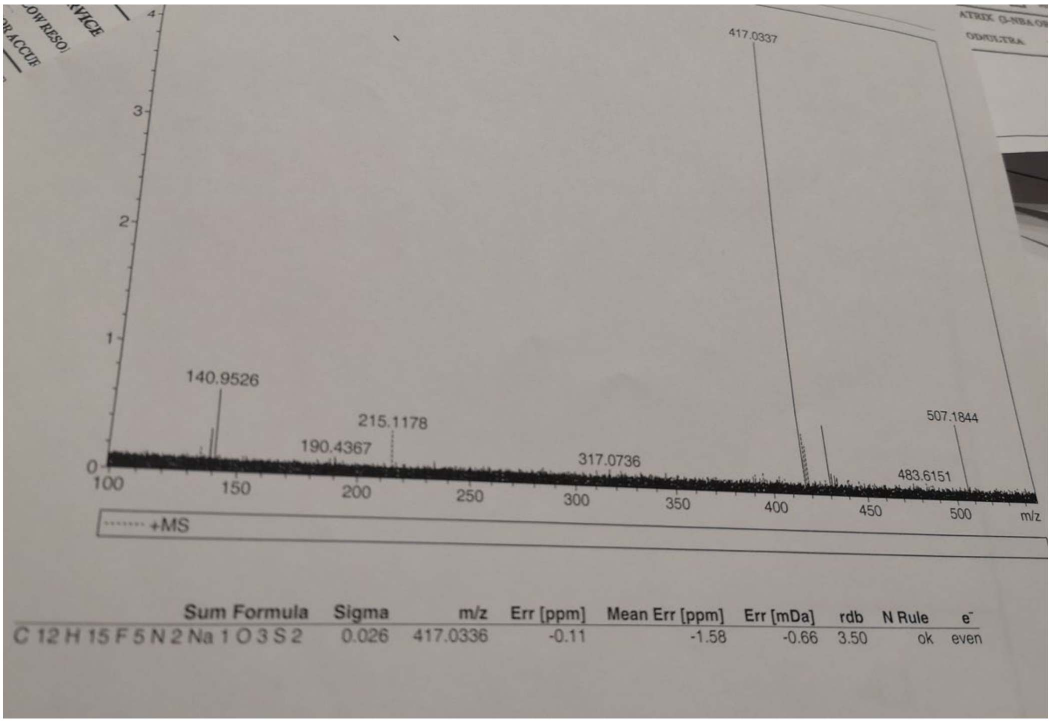

(3Z)-3-[(3,5-Dimethyl-1H-pyrrol-2-yl)methylidene]-5-(pentafluoro-l^{6}-sulfanyl)-2,3-

[4**-(Pentafluoro-λ^{6}-sulfanyl)phenyl](piperidin-1-yl)methanone (4a)**

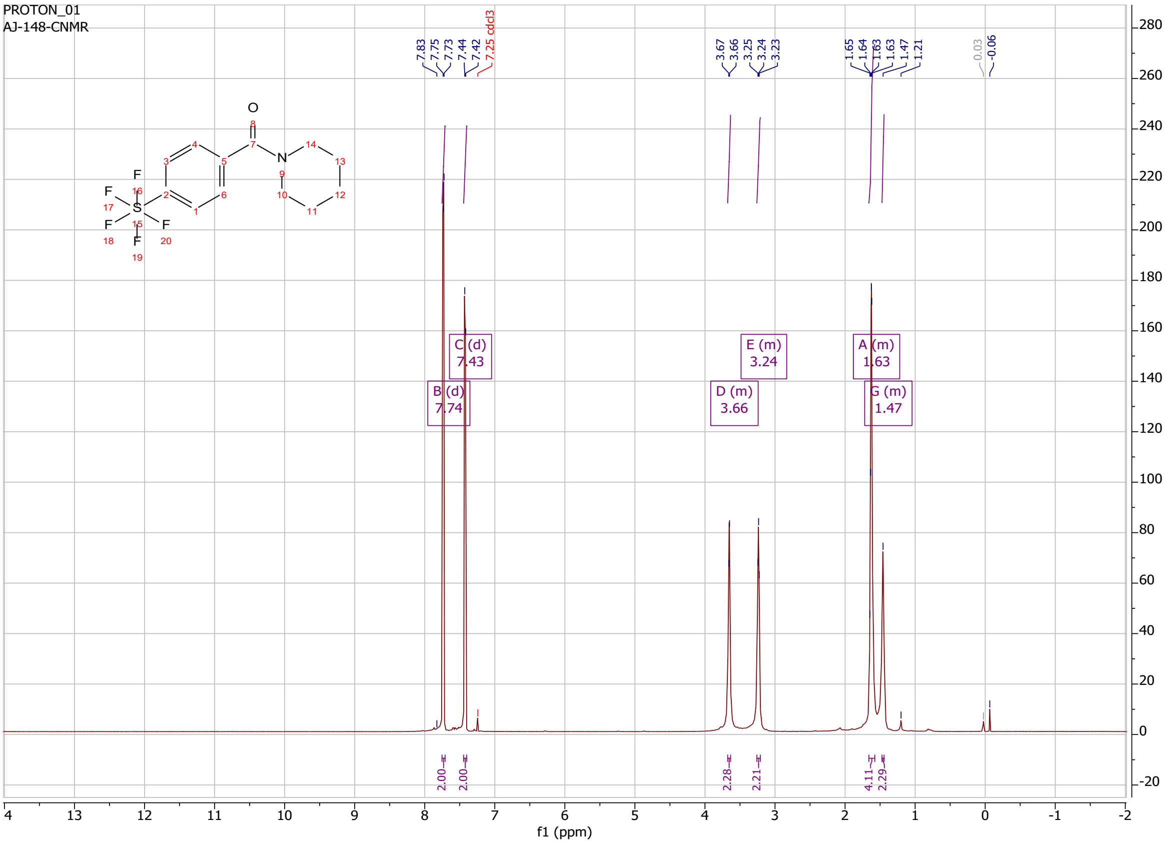

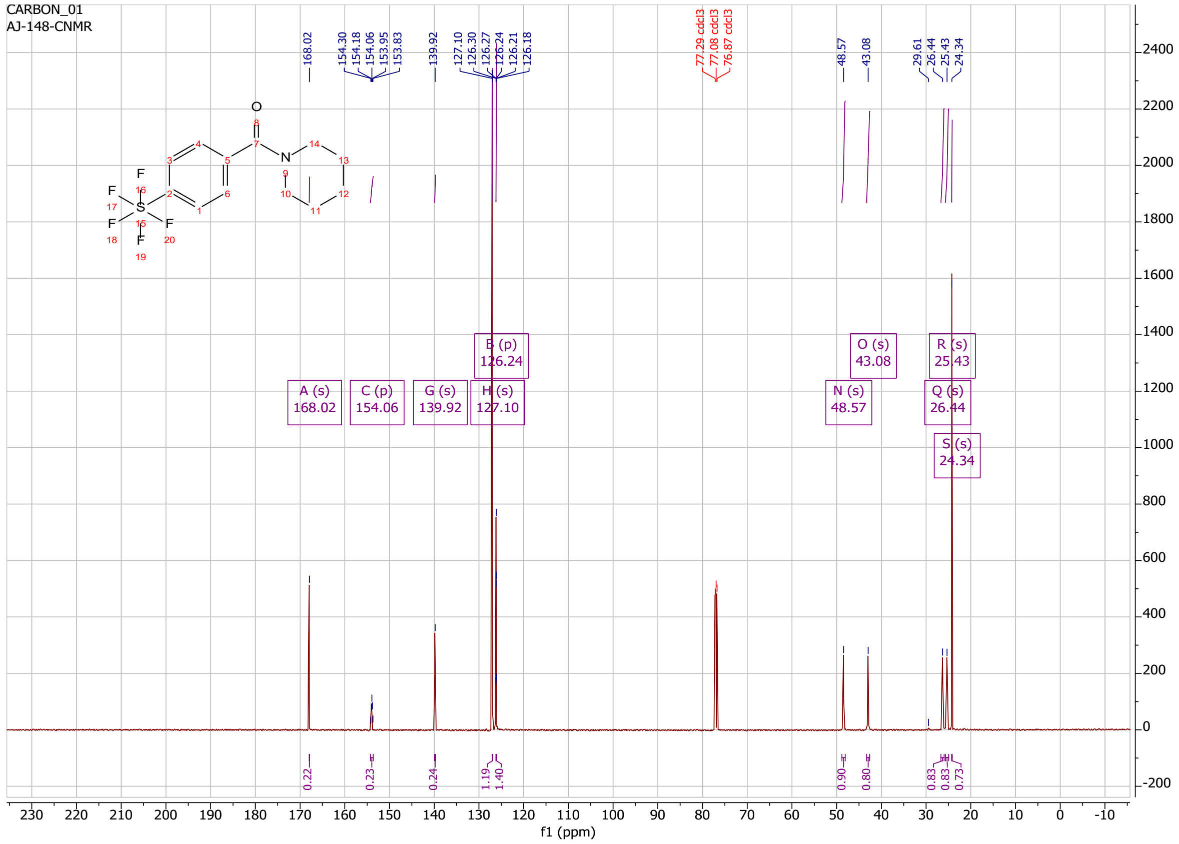
CARBON_01
AJ-148-CNMR
168.02
154.30
154.18
154.06
153.95
153.83
139.92
127.10
126.30
126.27
126.24
126.21
126.18
77.29 cdcl3
77.08 cdcl3
76.87 cdcl3
48.57
43.08
29.61
26.44
25.43
24.34
A (s) 168.02
C (p) 154.06
G (s) 139.92
B (p) 126.24
H (s) 127.10
N (s) 48.57
O (s) 43.08
R (s) 25.43
Q (s) 26.44
S (s) 24.34
0.22
0.23
0.24
1.19
1.40
0.90
0.80
0.83
0.83
0.73
f1 (ppm)

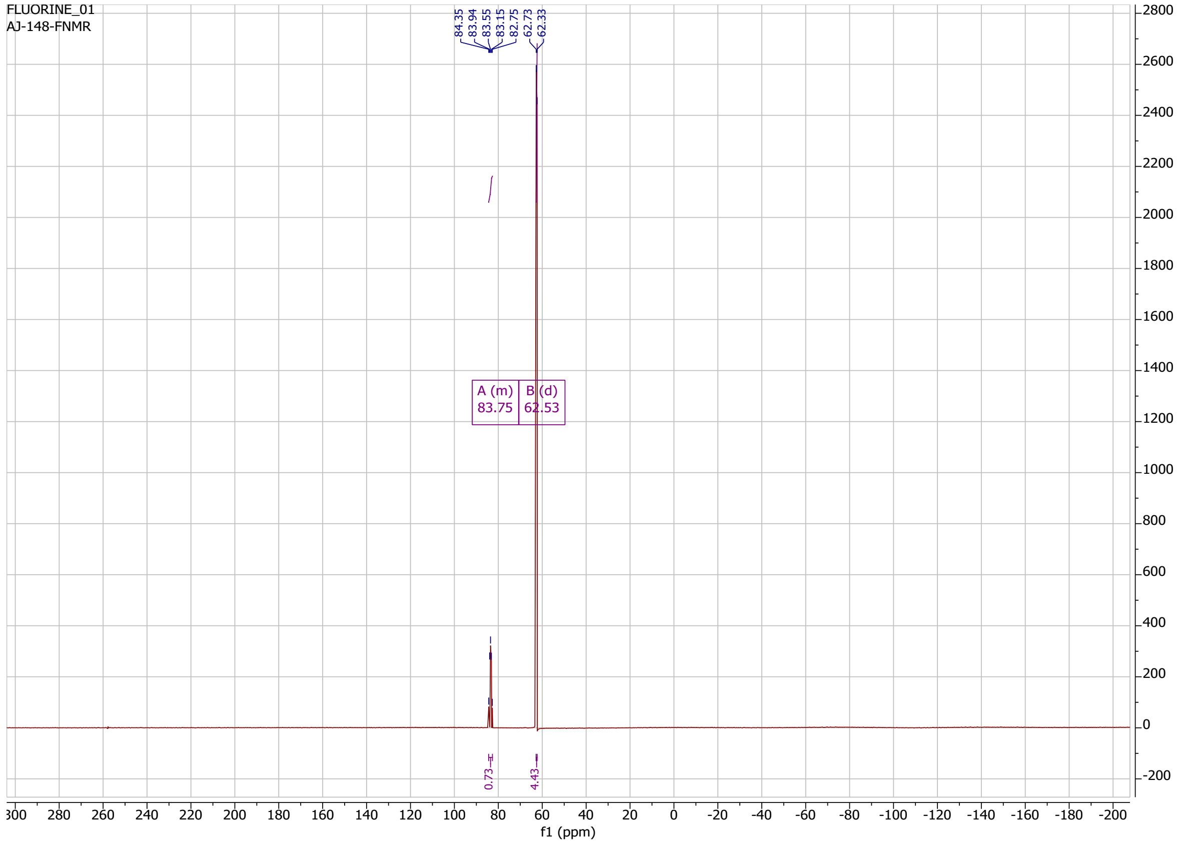
FLUORINE_01
AJ-148-FNMR
84.35
83.94
83.55
83.15
82.75
62.73
62.33
A (m) 83.75
B (d) 62.53
0.73
4.43
f1 (ppm)

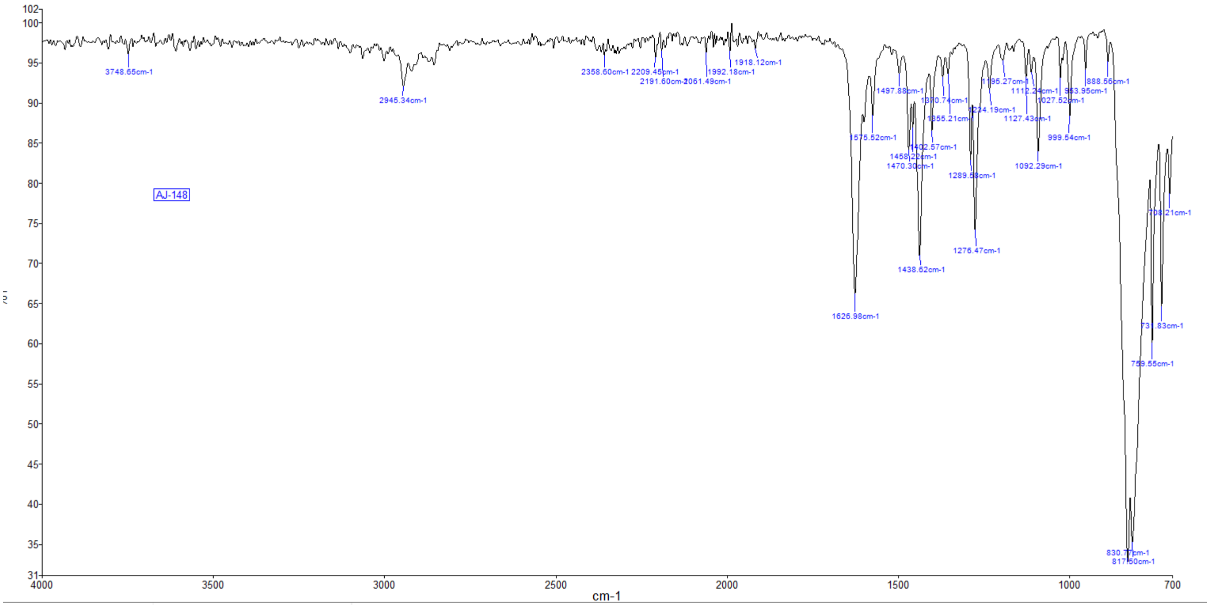

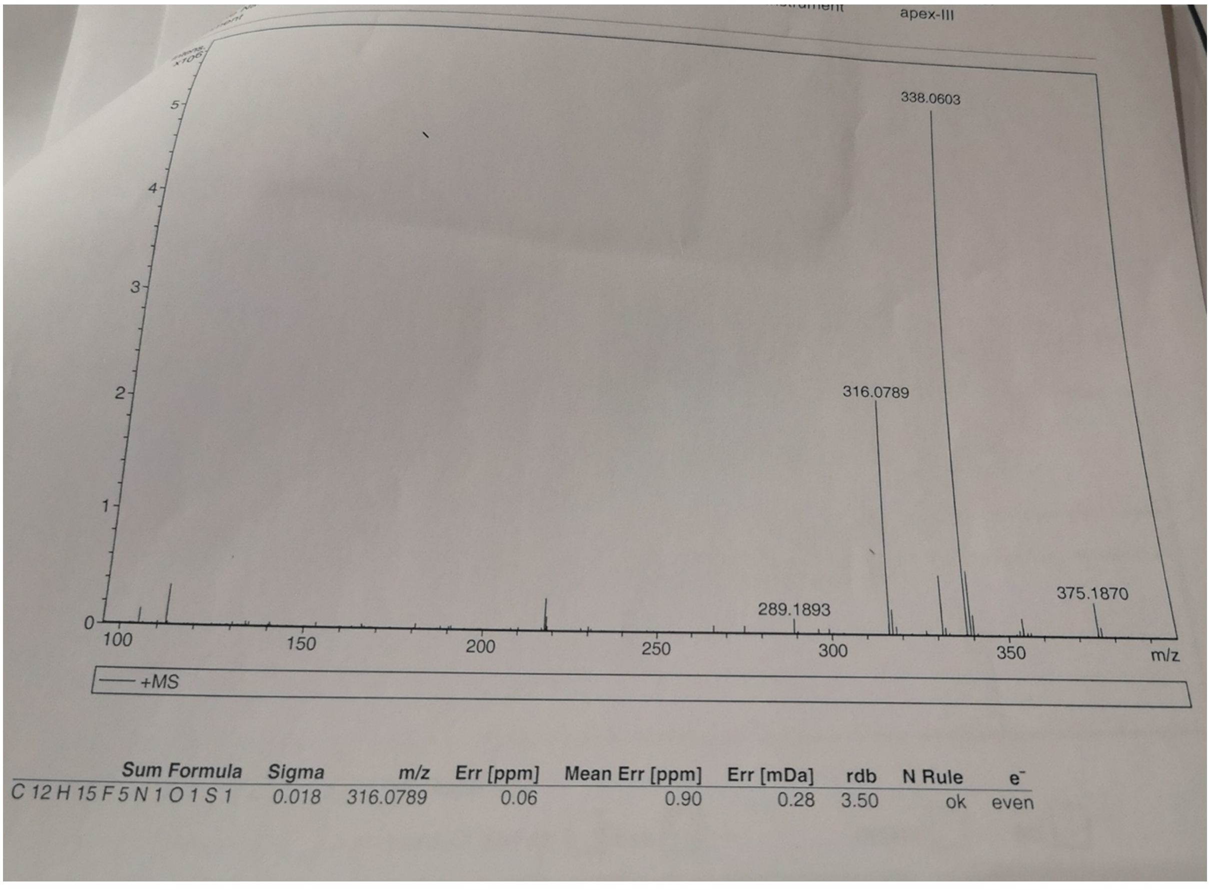

(4-Pentafluoro-λ^6-sulfanylphenyl)(4-methylpiperazin-1-yl)methanone) (4b)

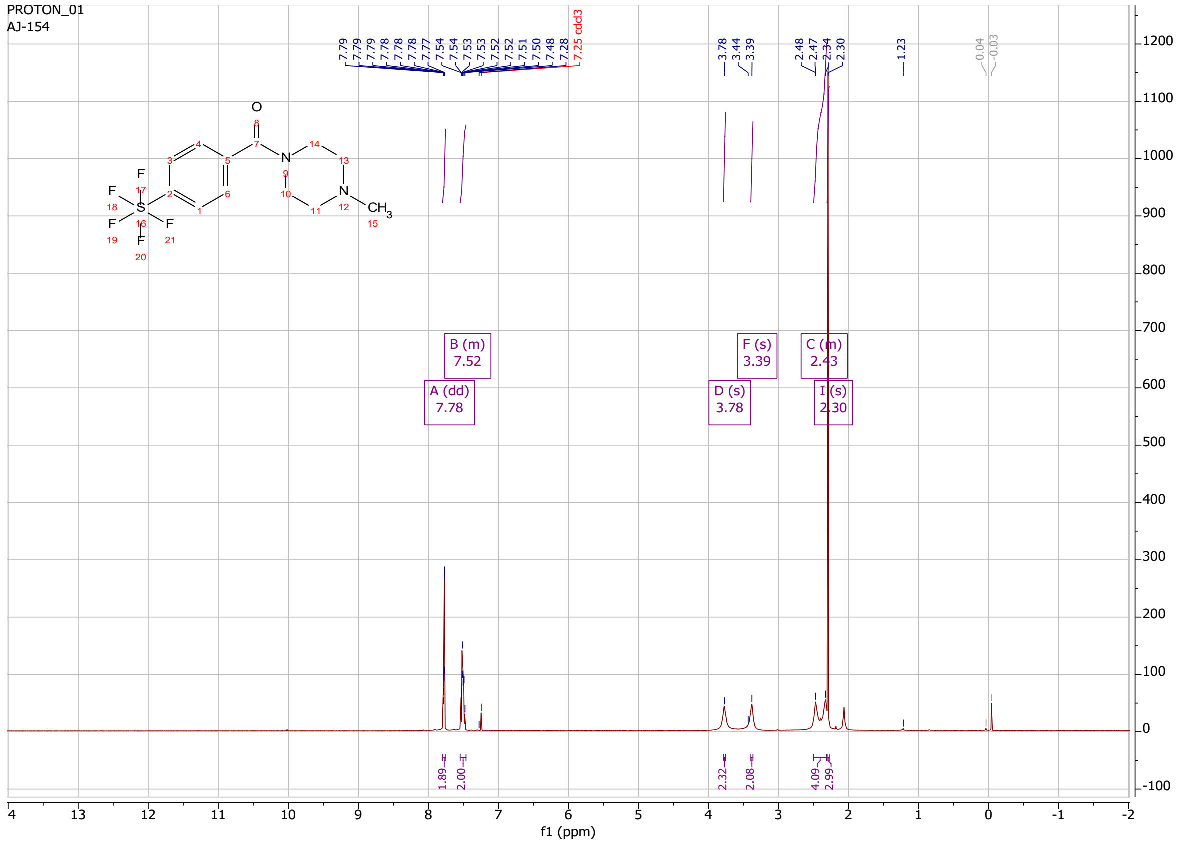
PROTON_01
AJ-154
7.79
7.79
7.79
7.78
7.78
7.78
7.77
7.54
7.54
7.53
7.53
7.52
7.52
7.51
7.50
7.48
7.28
7.25 cdcl3
3.78
3.44
3.39
2.48
2.47
2.34
2.30
1.23
0.04
-0.03
B (m)
7.52
A (dd)
7.78
F (s)
3.39
D (s)
3.78
C (m)
2.43
I (s)
2.30
1.89
2.00
2.32
2.08
4.09
2.99
f1 (ppm)

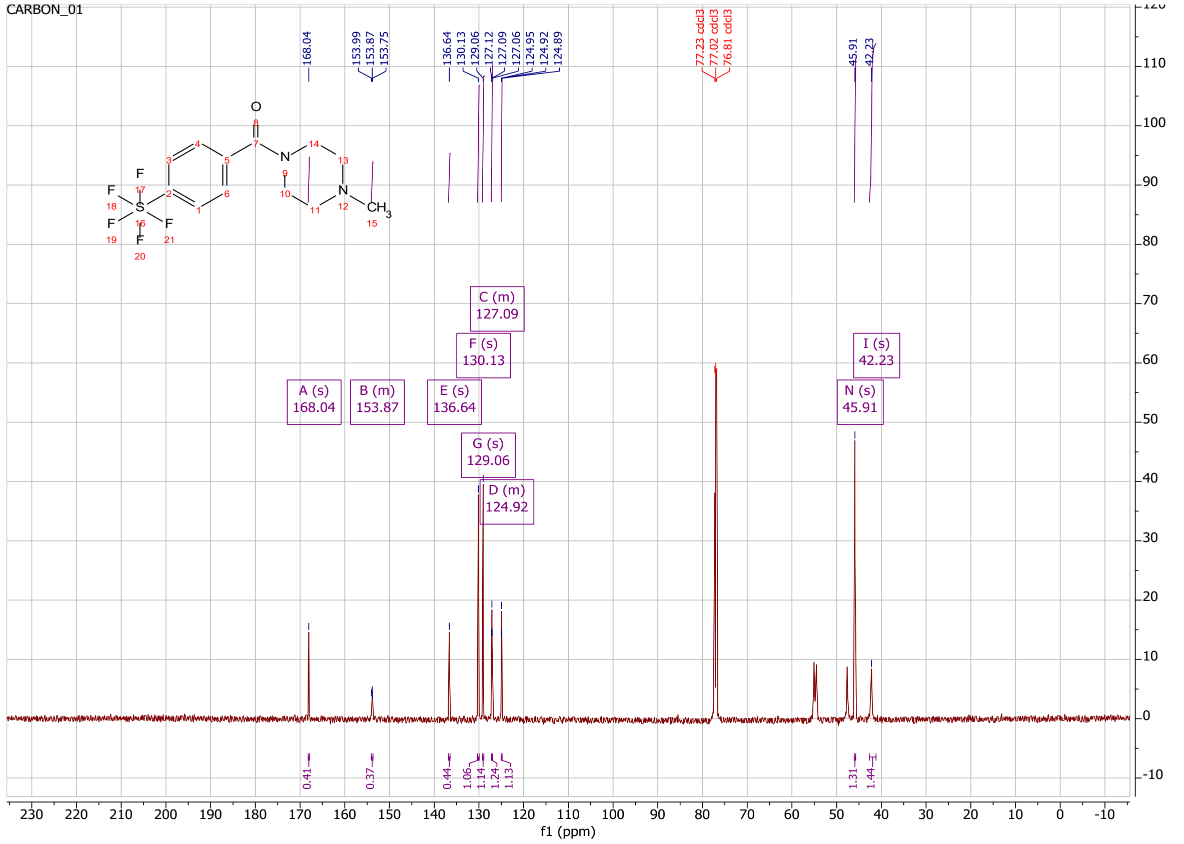
CARBON_01
168.04
153.99
153.87
153.75
136.64
130.13
129.06
127.12
127.09
127.06
124.95
124.92
124.89
77.23 cdcl3
77.02 cdcl3
76.81 cdcl3
45.91
42.23
C (m)
127.09
F (s)
130.13
I (s)
42.23
A (s)
168.04
B (m)
153.87
E (s)
136.64
N (s)
45.91
G (s)
129.06
D (m)
124.92
0.41
0.37
0.44
1.06
1.14
1.24
1.13
1.31
1.44
f1 (ppm)

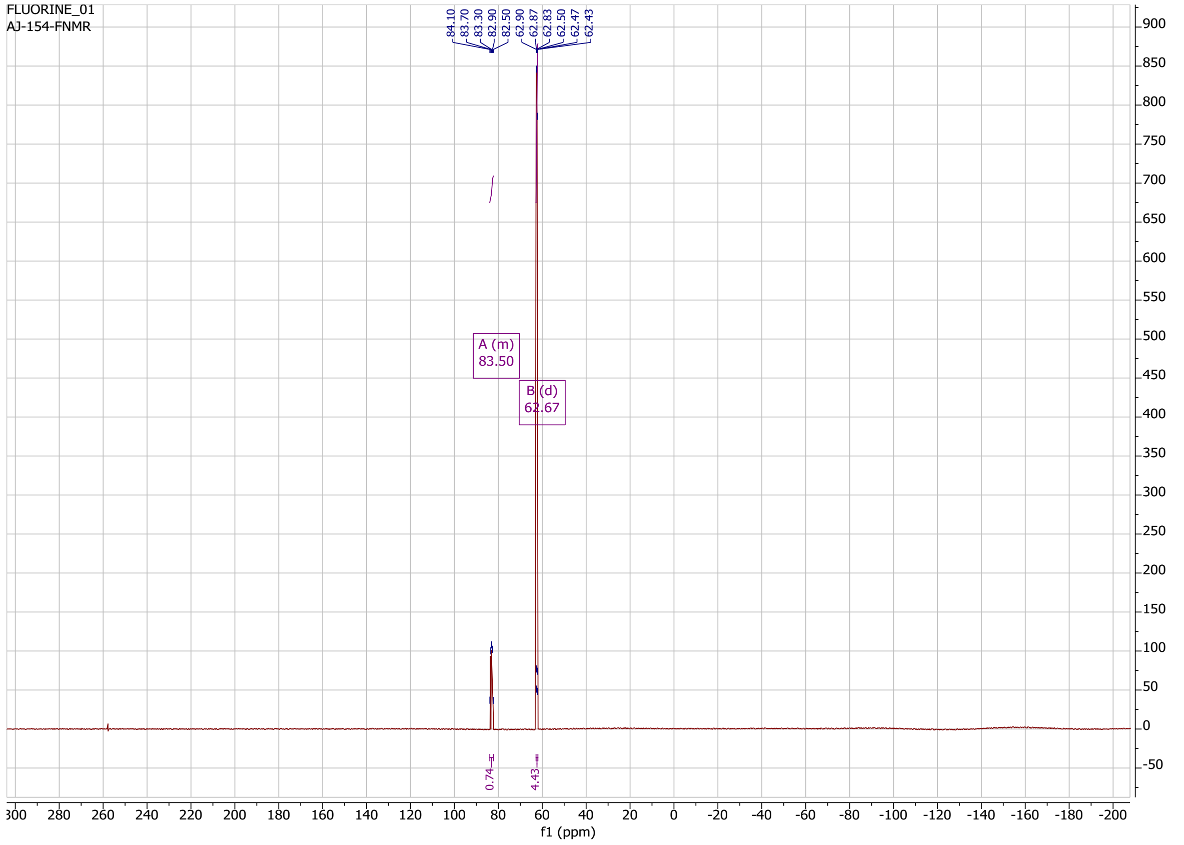
FLUORINE_01
AJ-154-FNMR
A (m)
83.50
B (d)
62.67
0.74
4.43
f1 (ppm)

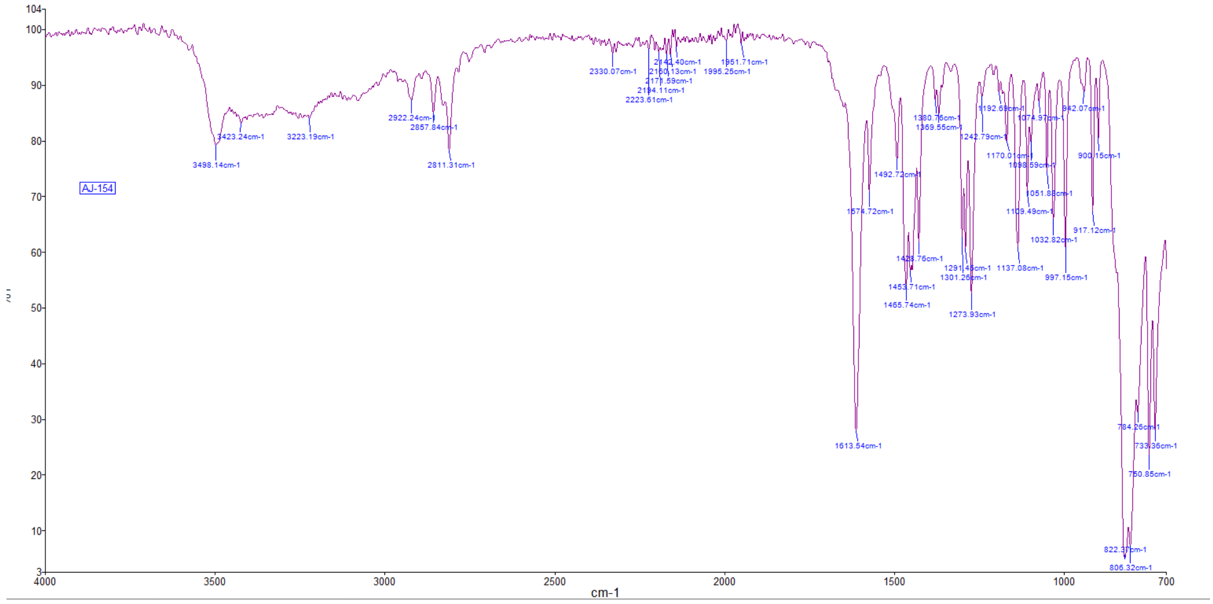
AJ-154
cm-1
1613.54cm-1
806.32cm-1
822.37cm-1
750.85cm-1
733.36cm-1
784.26cm-1
3498.14cm-1
2811.31cm-1

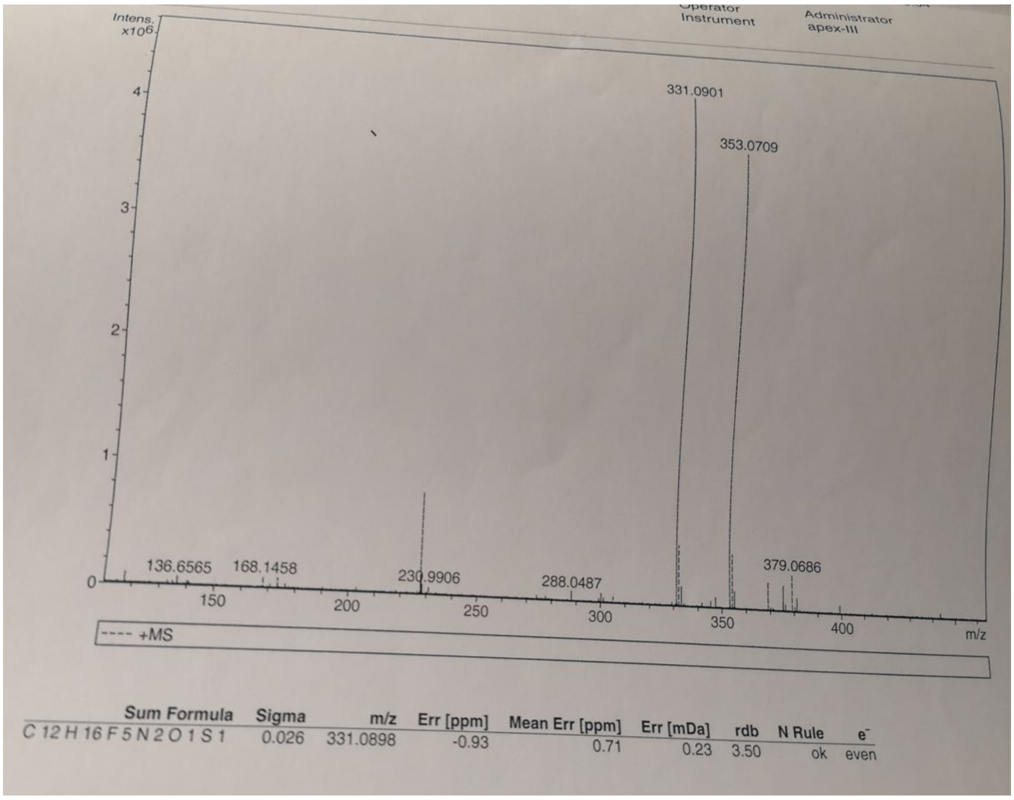

[4-(Pentafluorol-□^6-sulfanyl)phenyl)](morpholino)methanone (4c)

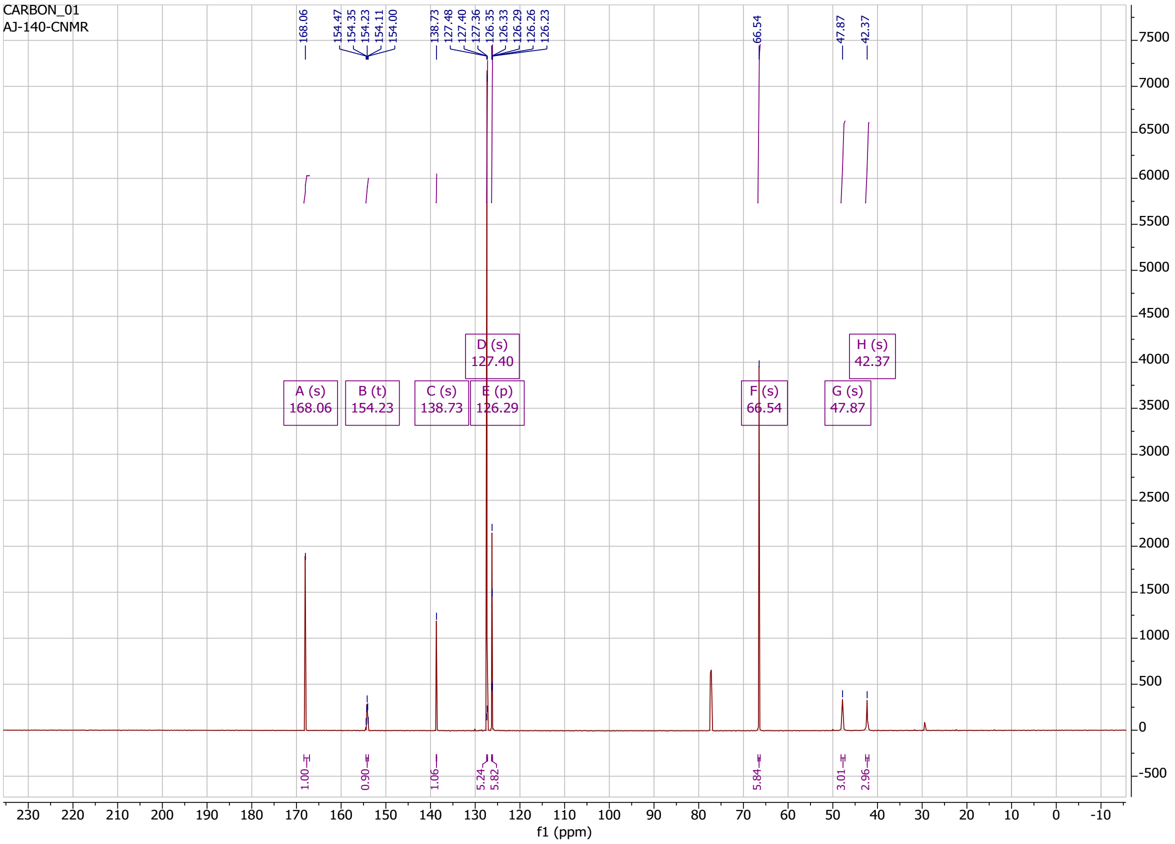

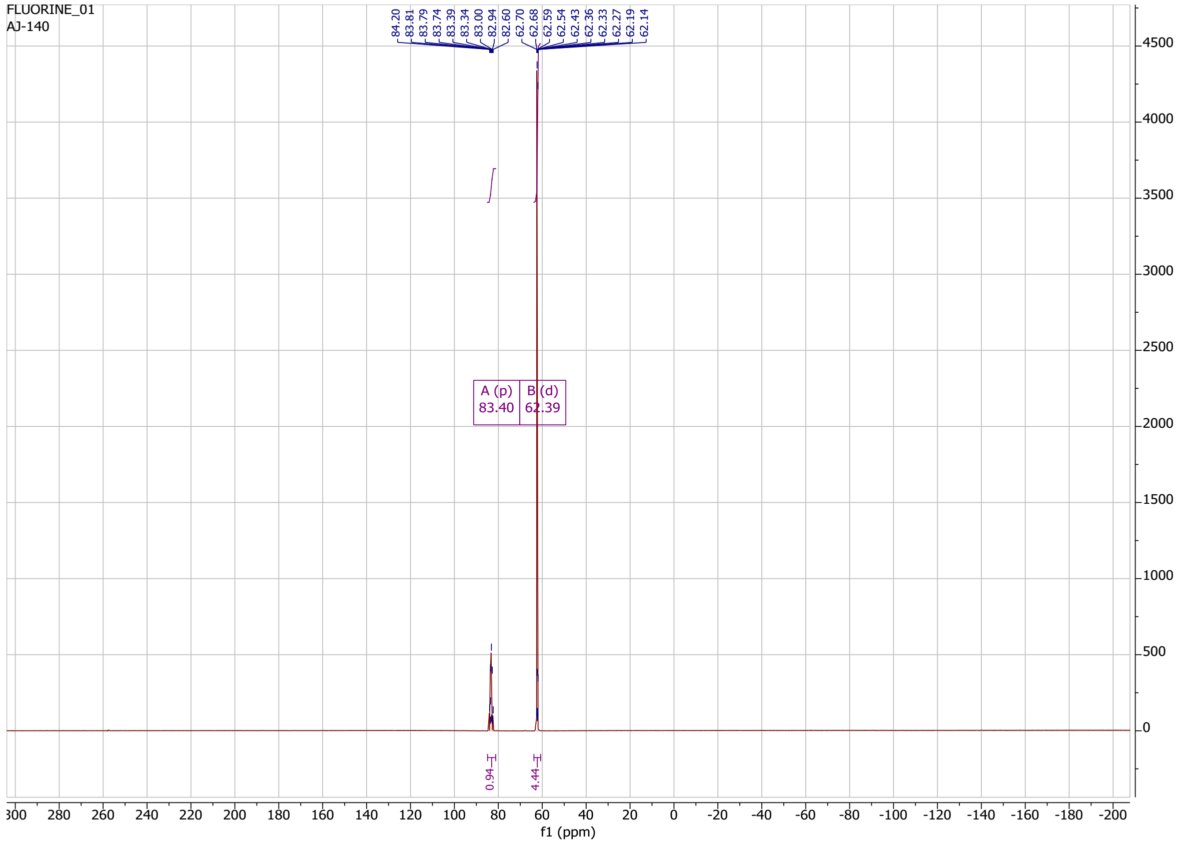

FLUORINE_01
AJ-140
84.20 83.81 83.79 83.74 83.39 83.34 83.00 82.94 82.60 62.70 62.68 62.59 62.54 62.43 62.36 62.33 62.27 62.19 62.14
A (p) 83.40
B (d) 62.39
0.94
4.44
f1 (ppm)

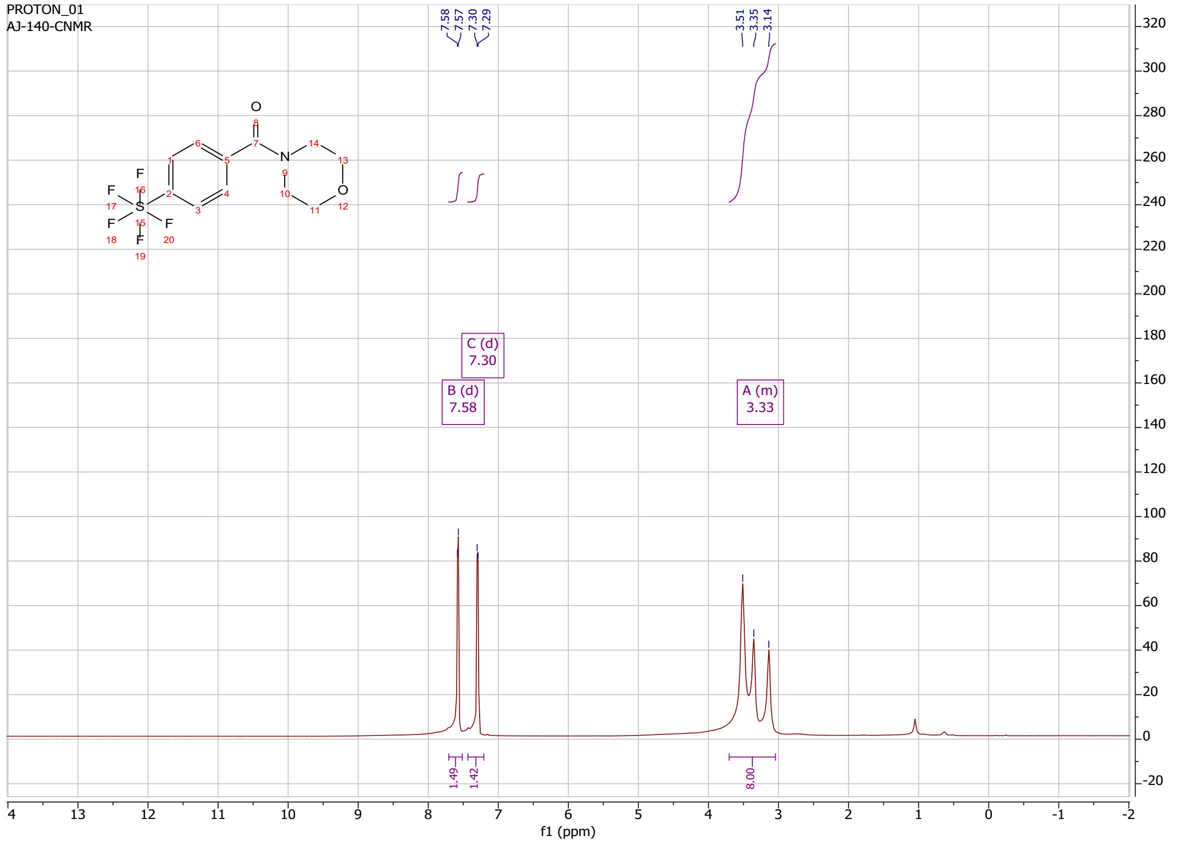

PROTON_01
AJ-140-CNMR
7.58 7.57 7.30 7.29
3.51 3.35 3.14
C (d) 7.30
B (d) 7.58
A (m) 3.33
1.49
1.42
8.00
f1 (ppm)

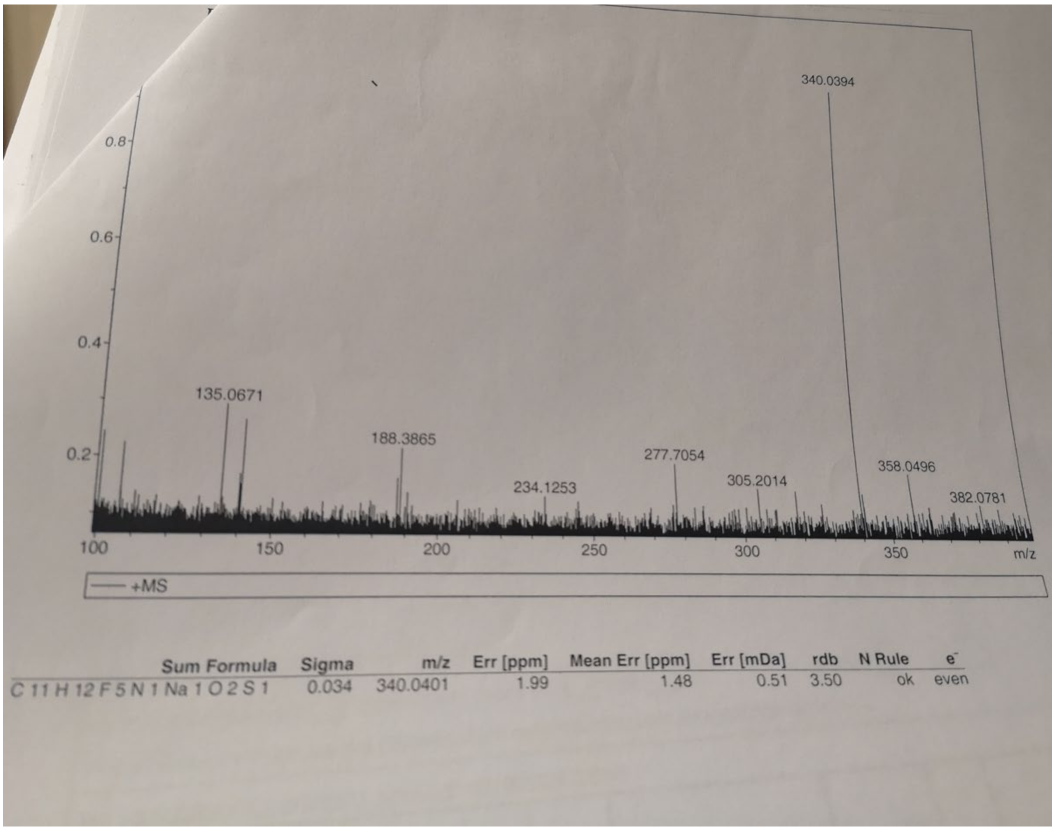

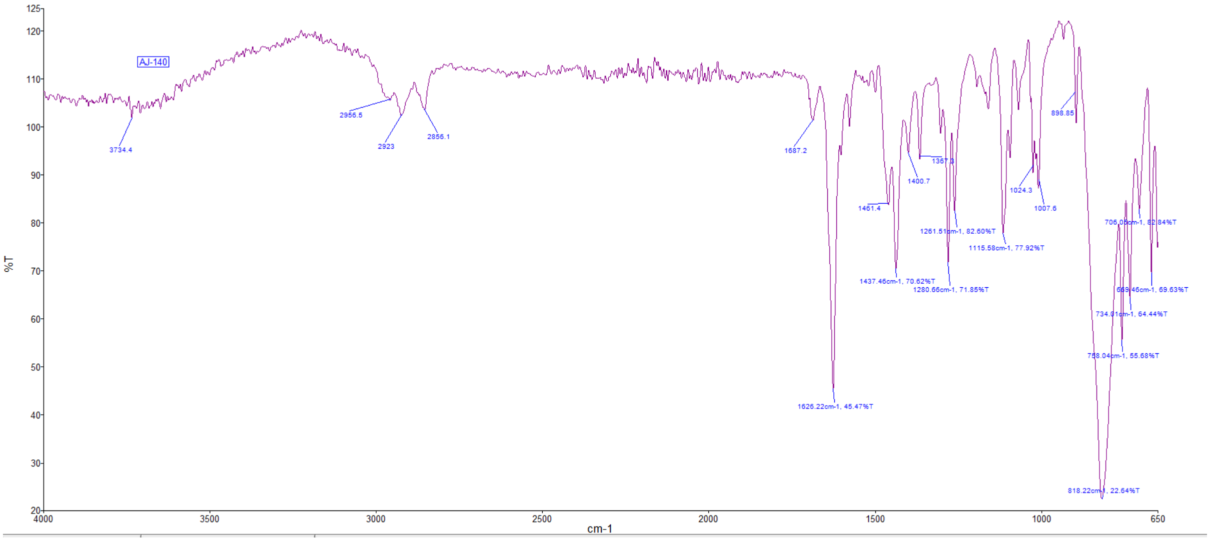

tert-Butyl 4-(4-pentafluoro-λ^{6}-sulfanyl) benzoyl piperazine-1-carboxylate (4d)

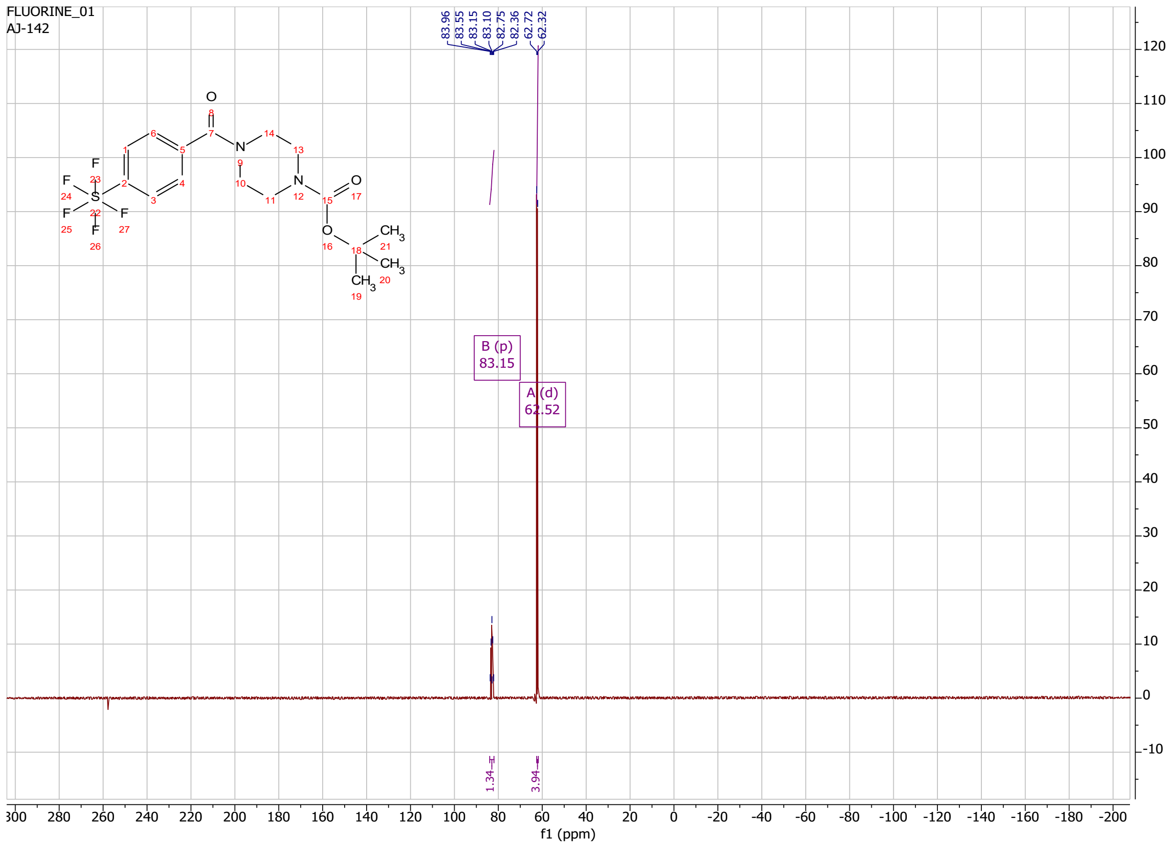
FLUORINE_01
AJ-142
83.96
83.55
83.15
83.10
82.75
82.36
62.72
62.32
B (p)
83.15
A (d)
62.52
1.34
3.94
f1 (ppm)

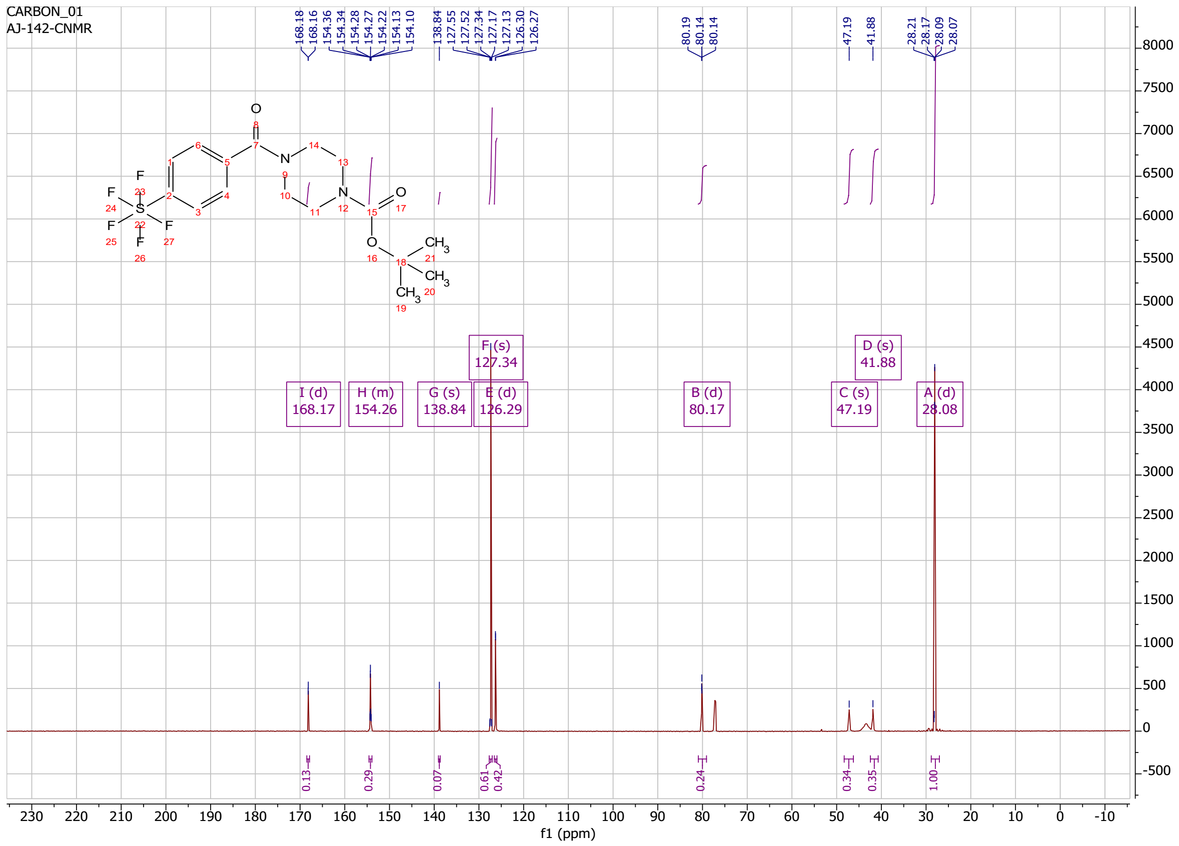
CARBON_01
AJ-142-CNMR
168.18
168.16
154.36
154.34
154.28
154.27
154.22
154.13
154.10
138.84
127.55
127.52
127.34
127.17
127.13
126.30
126.27
80.19
80.14
80.14
47.19
41.88
28.21
28.17
28.09
28.07
F (s)
127.34
D (s)
41.88
I (d)
168.17
H (m)
154.26
G (s)
138.84
E (d)
126.29
B (d)
80.17
C (s)
47.19
A (d)
28.08
0.13
0.29
0.07
0.61
0.42
0.24
0.34
0.35
1.00
f1 (ppm)

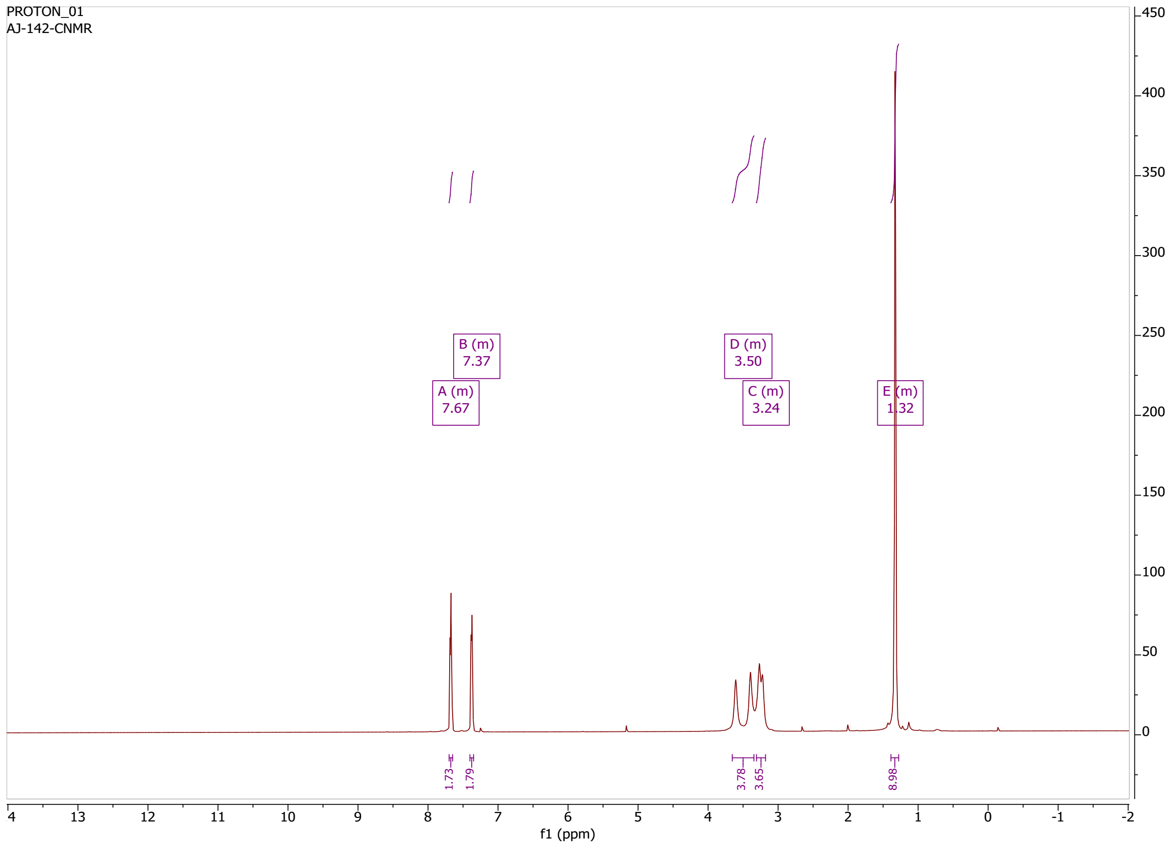

PerkinElmer Spectrum Version 10.03.06
24 September 2019 10:45

Analyst PEService
Date 24 September 2019 10:45

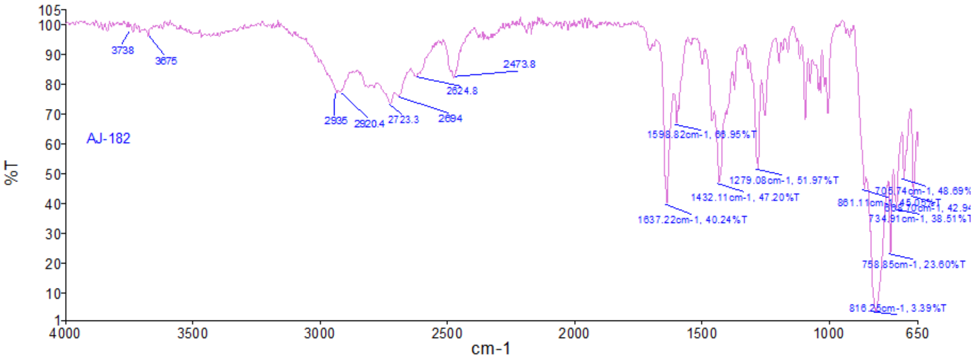

Sample Name	Description	Quality Checks
PEService 27	Sample 027 By PEService Date Monday, September 23 2019	The Quality Checks do not report any warnings for the sample.

Page 1

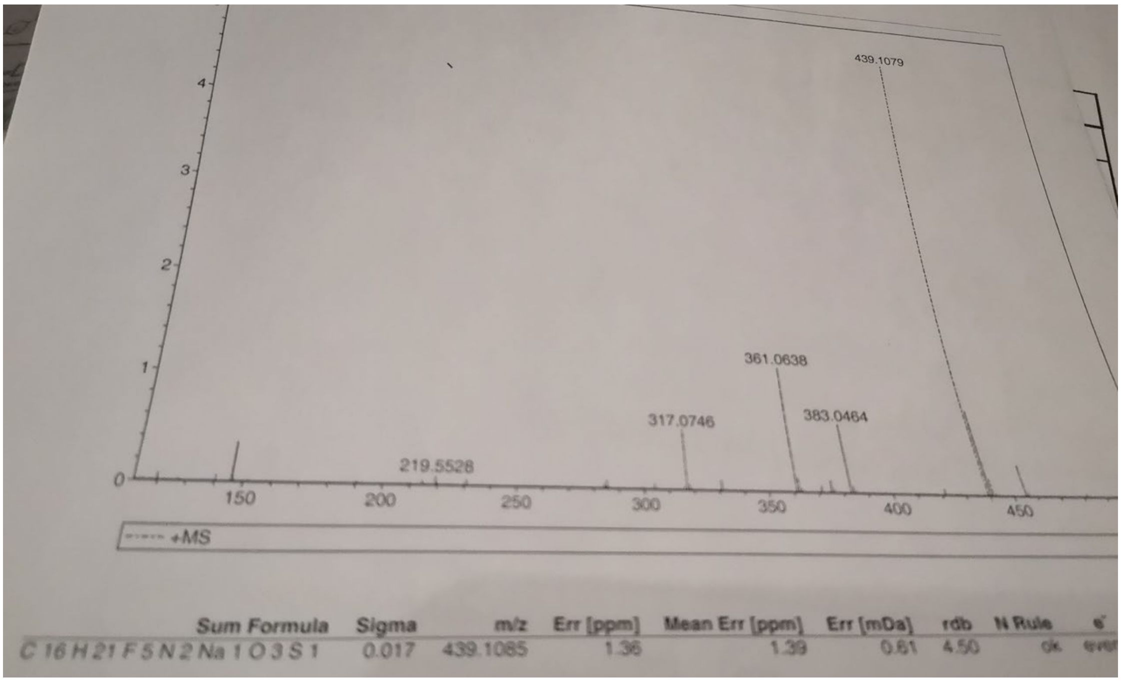

Sum Formula	Sigma	m/z	Err [ppm]	Mean Err [ppm]	Err [mDa]	rdb	N Rule
C 16 H 21 F 5 N 2 Na 1 O 3 S 1	0.017	439.1085	1.36	1.39	0.61	4.50	ok

1-[4-[4-(Pentafluoro-λ^6-sulfanyl)benzoyl]piperazin-1-yl]ethanone (4e)

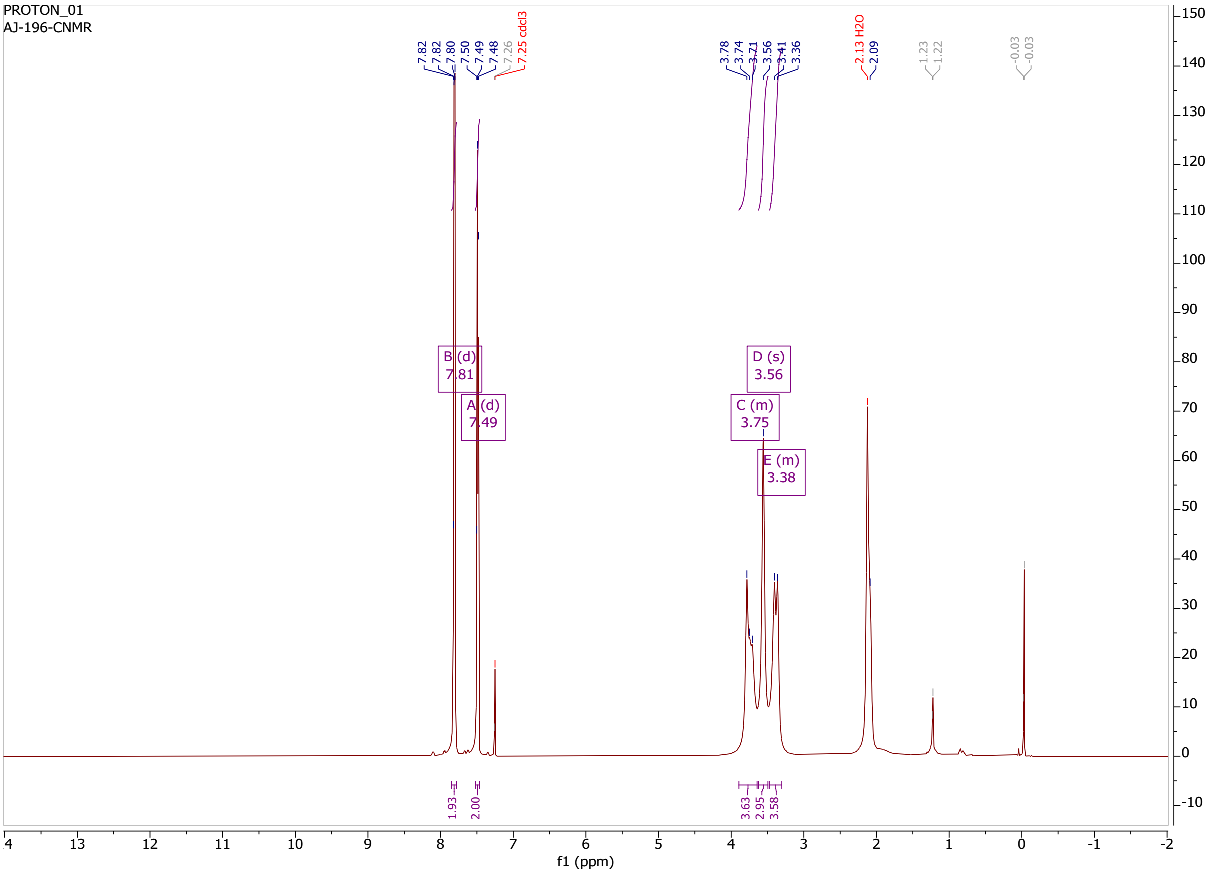

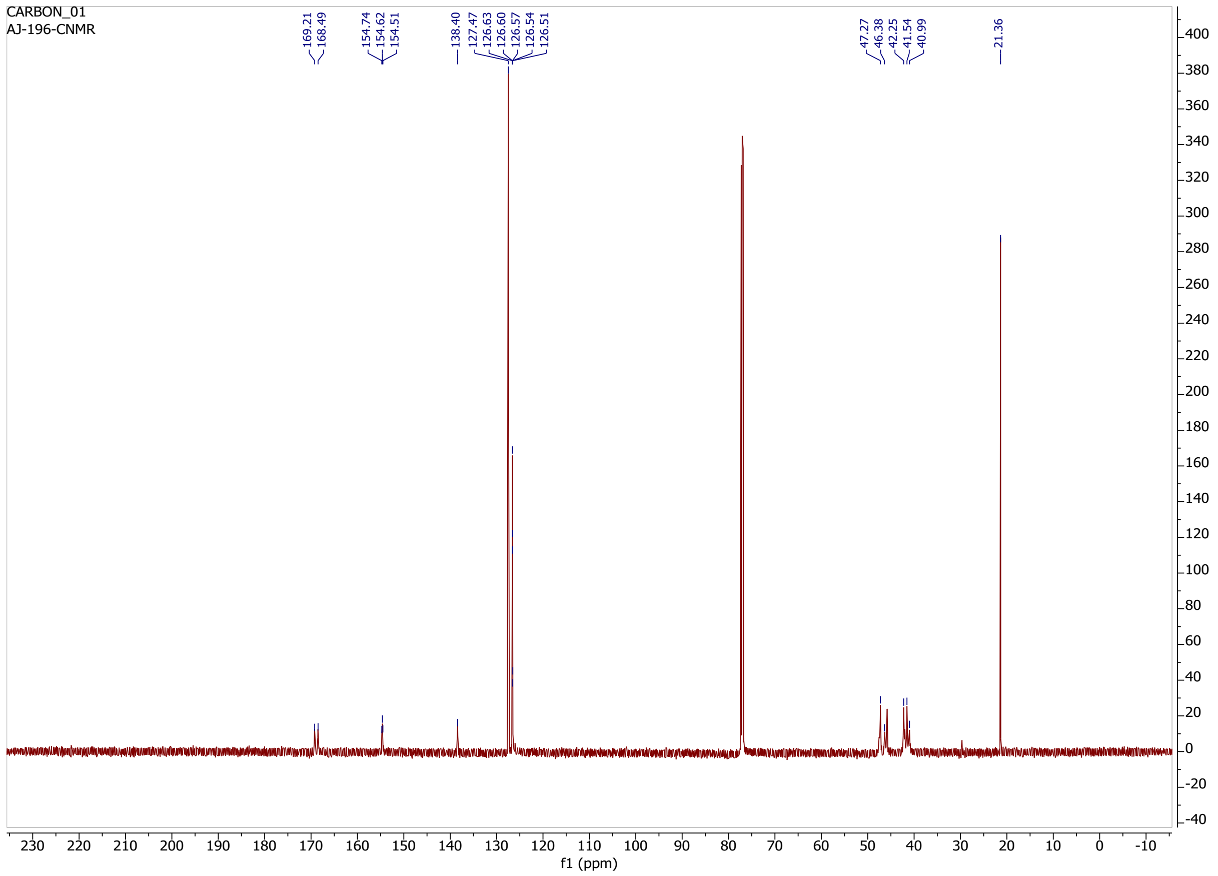
CARBON_01
AJ-196-CNMR
169.21
168.49
154.74
154.62
154.51
138.40
127.47
126.63
126.60
126.57
126.54
126.51
47.27
46.38
42.25
41.54
40.99
21.36
f1 (ppm)

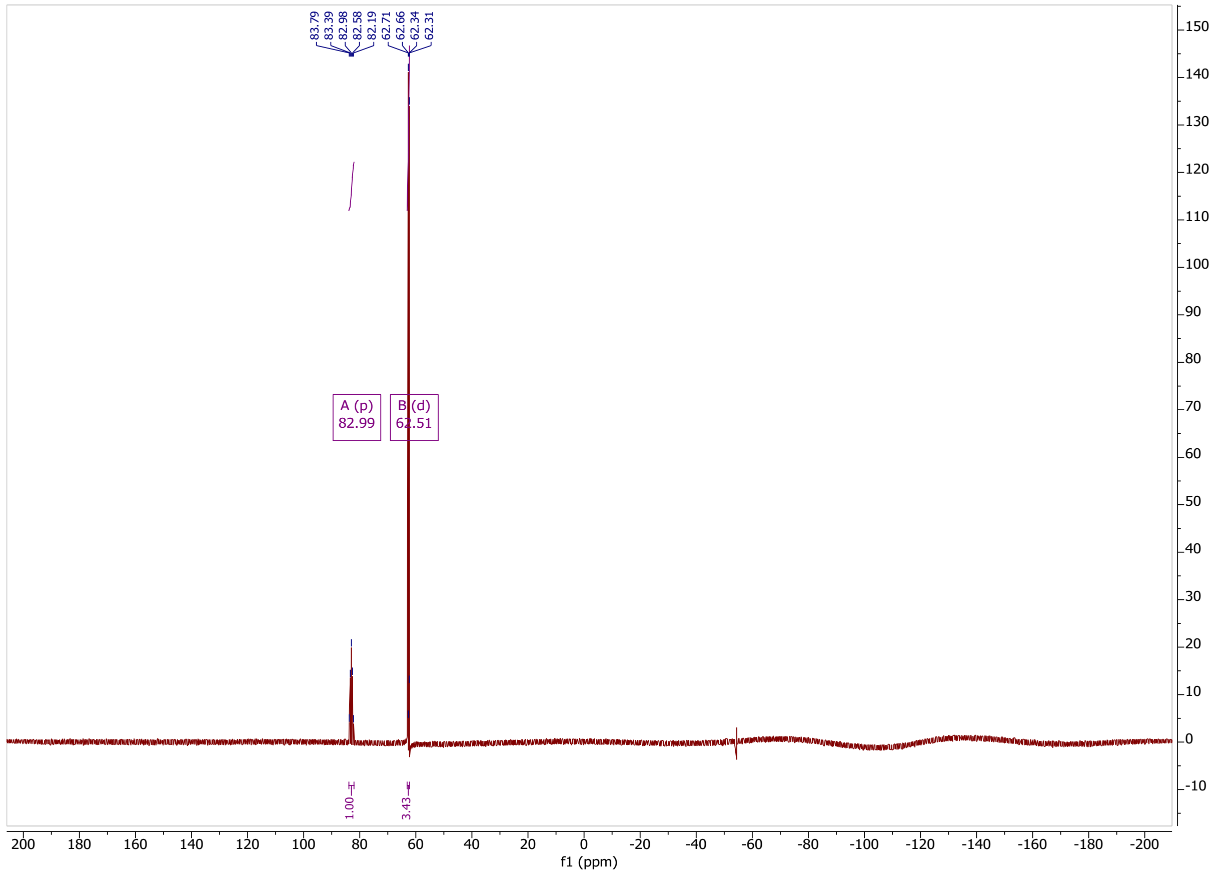
83.79
83.39
82.98
82.58
82.19
62.71
62.66
62.34
62.31
A (p)
82.99
B (d)
62.51
1.00
3.43
f1 (ppm)

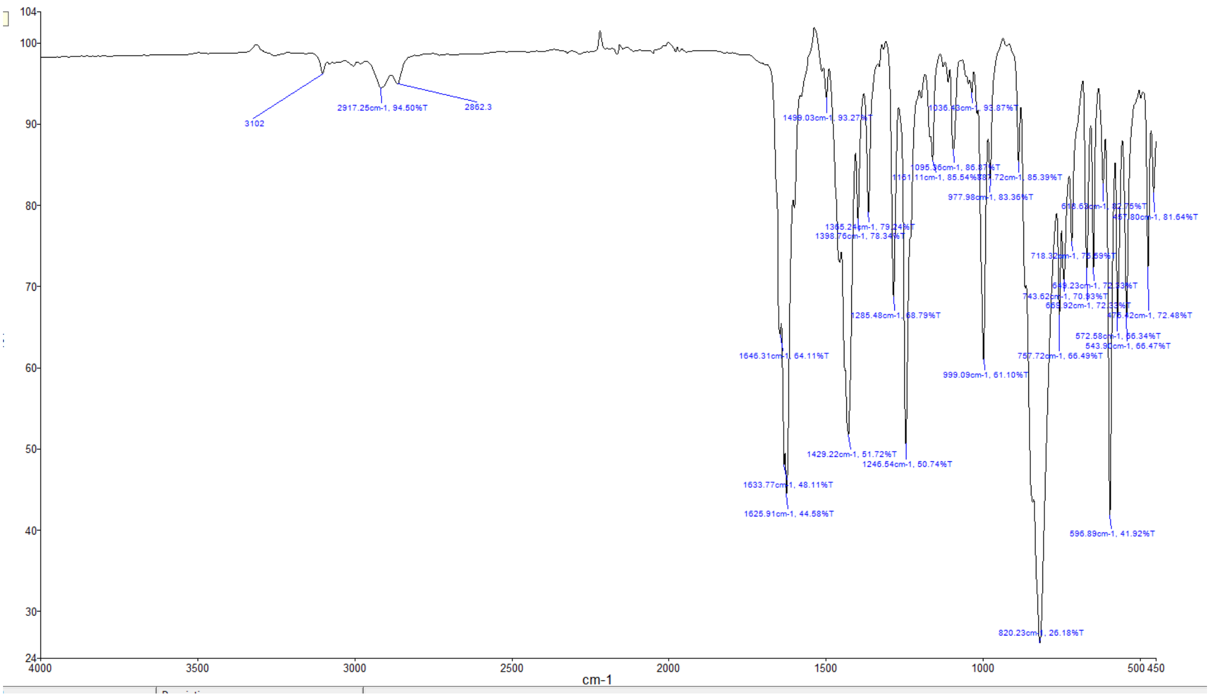

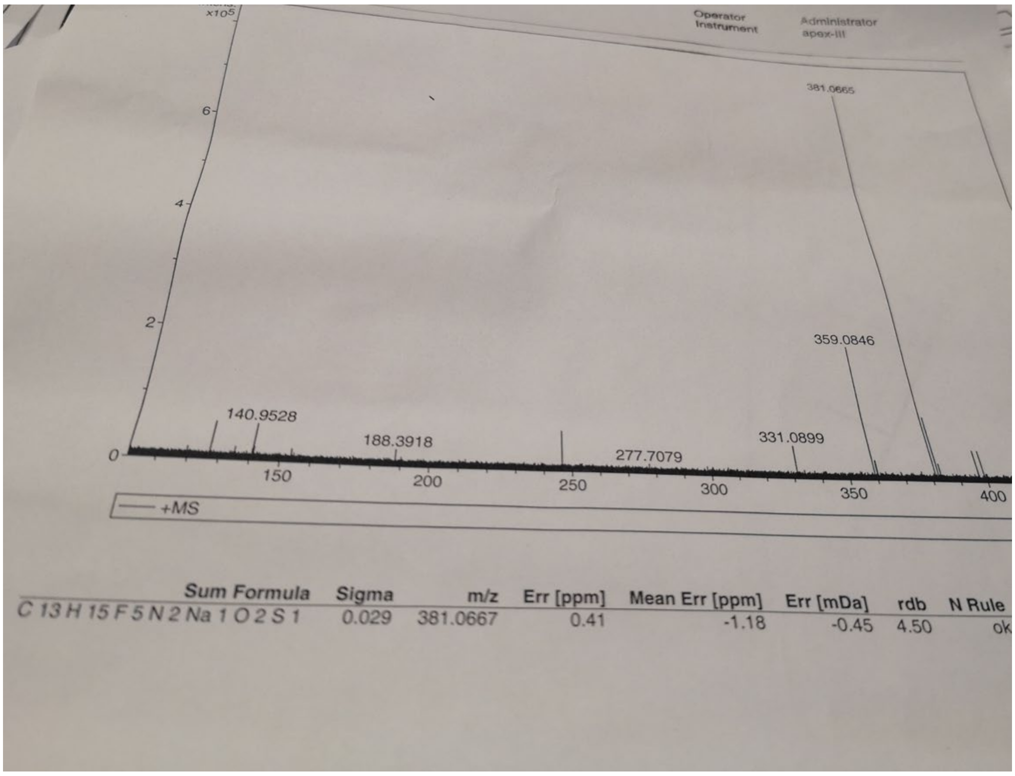

(4-Pentafluoro-□[6]sulfanyl phenyl)(4-(methylsulfonyl)piperazin-1-yl)methanone (4f)

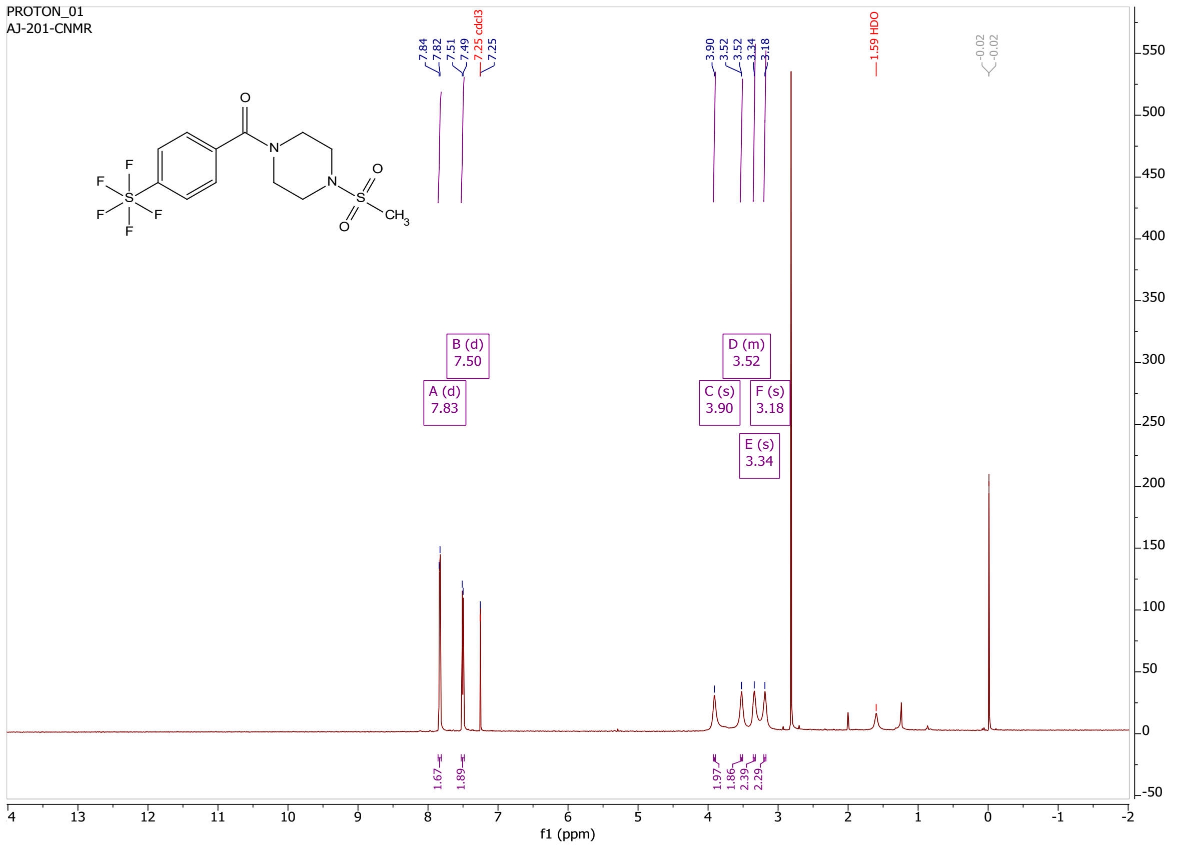
PROTON_01
AJ-201-CNMR
7.84
7.82
7.51
7.49
7.25 cdcl3
7.25
3.90
3.52
3.52
3.34
3.18
1.59 HDO
-0.02
-0.02
B (d)
7.50
A (d)
7.83
D (m)
3.52
C (s)
3.90
F (s)
3.18
E (s)
3.34
1.67
1.89
1.97
1.86
2.39
2.29
f1 (ppm)

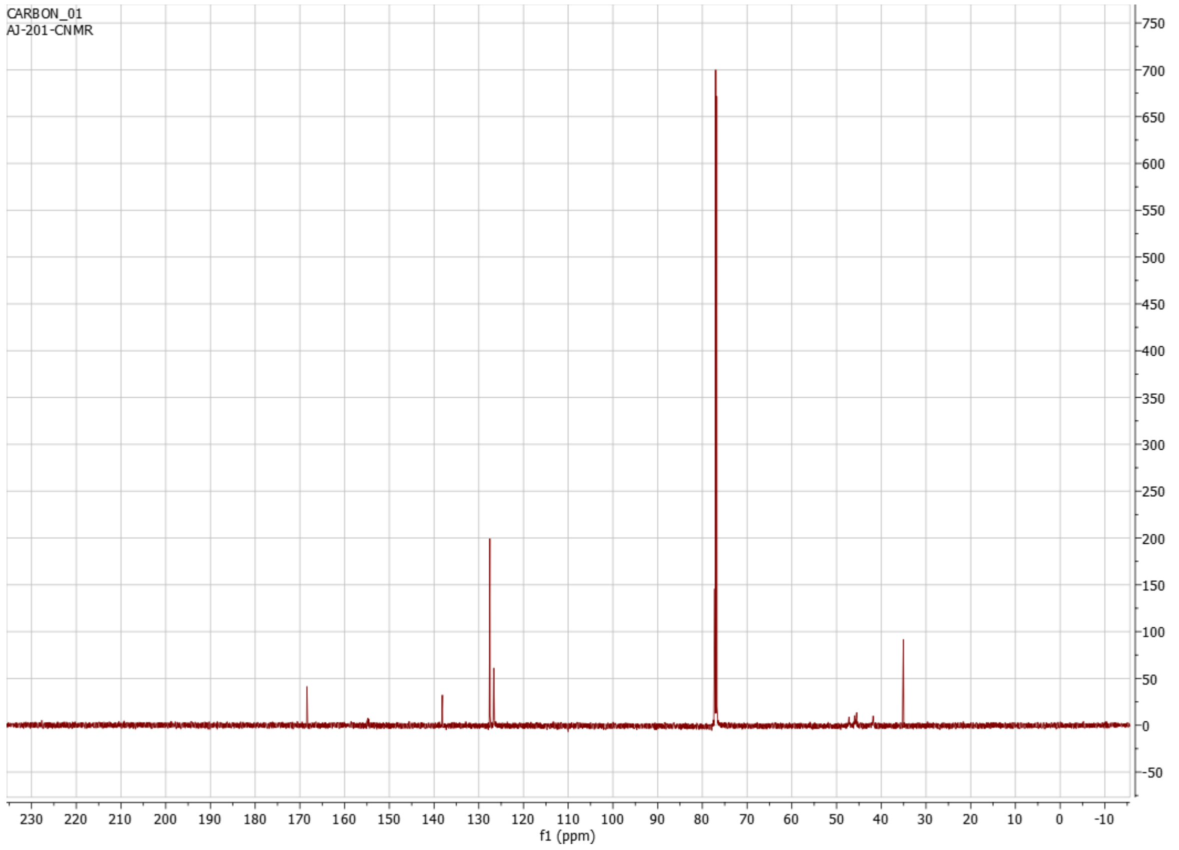
CARBON_01
AJ-201-CNMR
f1 (ppm)

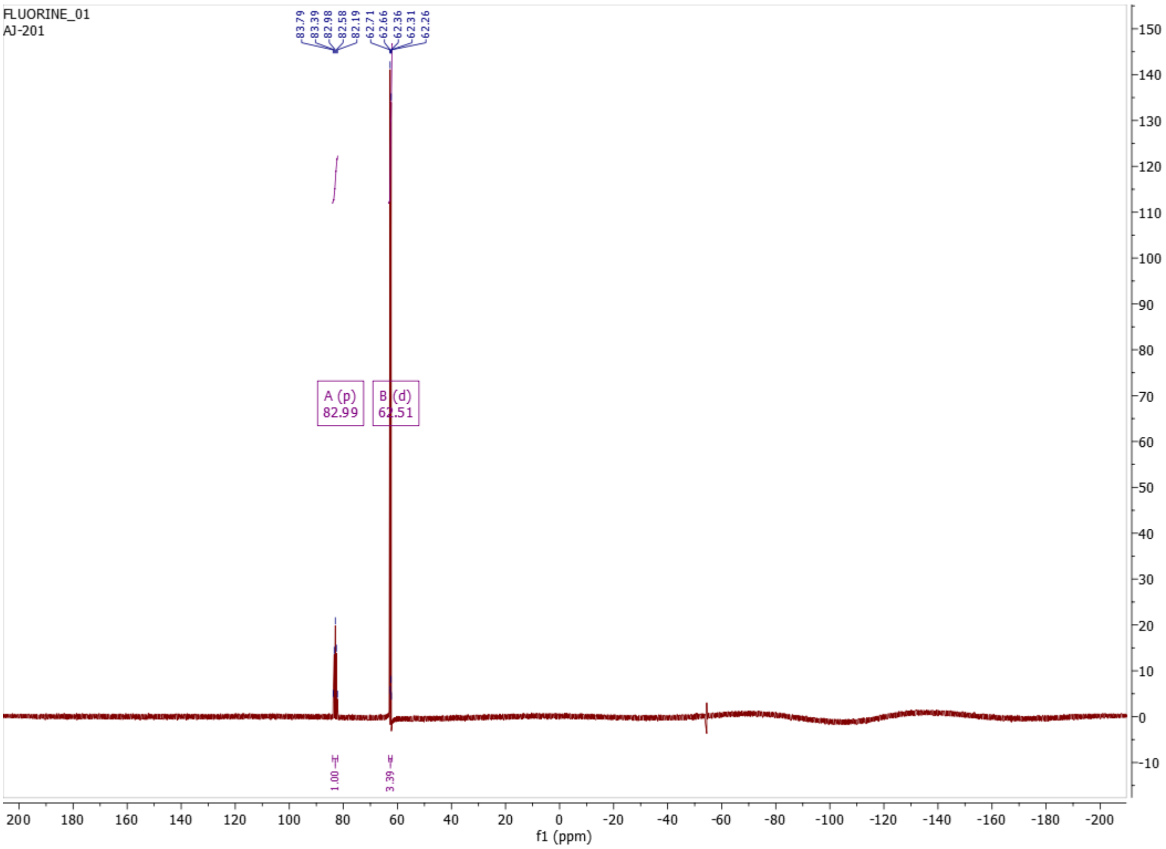

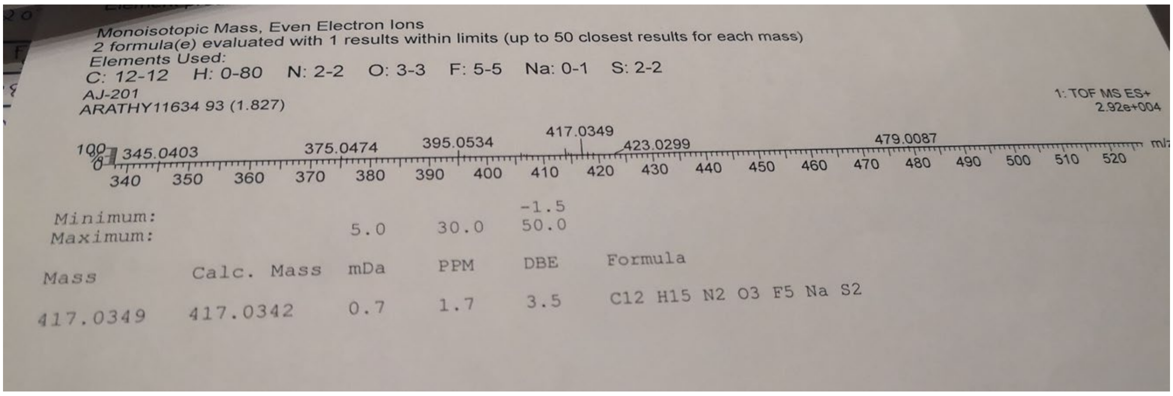

Monoisotopic Mass, Even Electron Ions
2 formula(e) evaluated with 1 results within limits (up to 50 closest results for each mass)
Elements Used:
C: 12-12 H: 0-80 N: 2-2 O: 3-3 F: 5-5 Na: 0-1 S: 2-2
AJ-201
ARATHY11634 93 (1.827)

1: TOF MS ES+
2.92e+004

Minimum:				-1.5	
Maximum:		5.0	30.0	50.0	
Mass	Calc. Mass	mDa	PPM	DBE	Formula
417.0349	417.0342	0.7	1.7	3.5	C12 H15 N2 O3 F5 Na S2

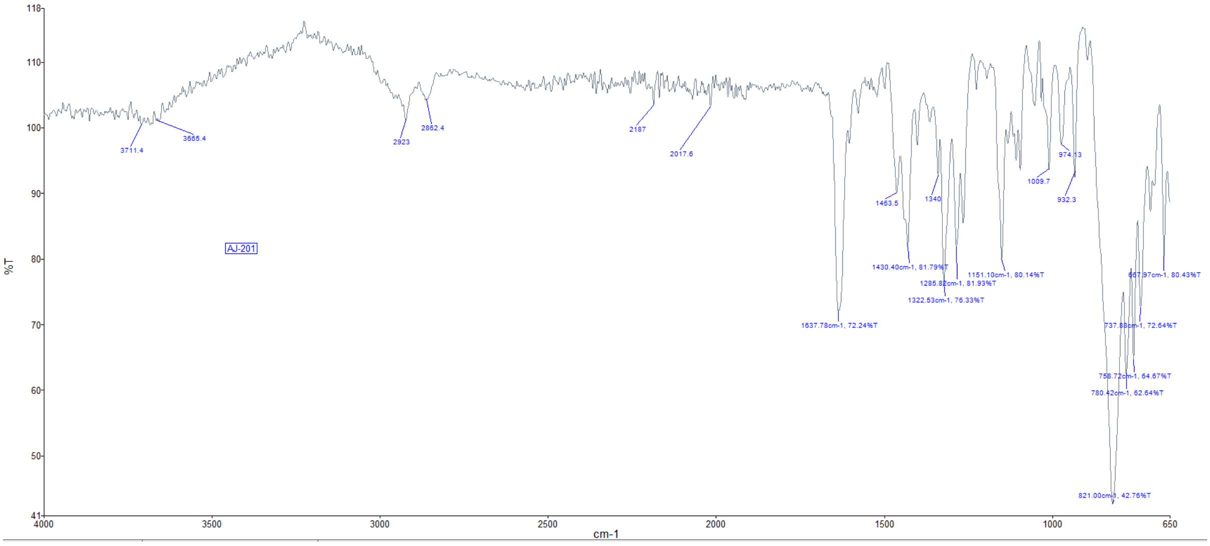

(3Z)-3-[(3,5-Dimethyl-1H-pyrrol-2-yl)methylidene]-5-(pentafluoro-□^{6}-sulfanyl)-2,3-dihydro-1H-indol-2-one (8a)

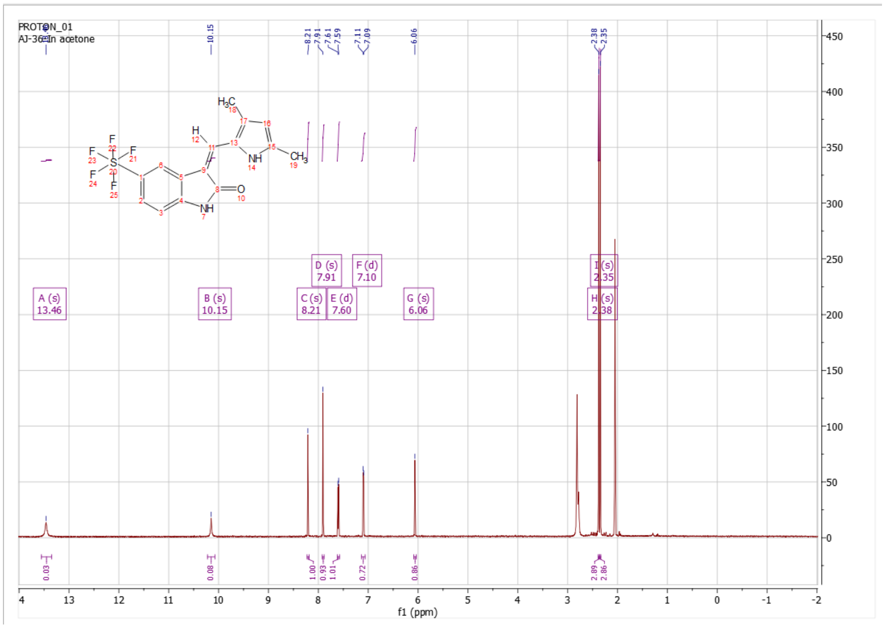

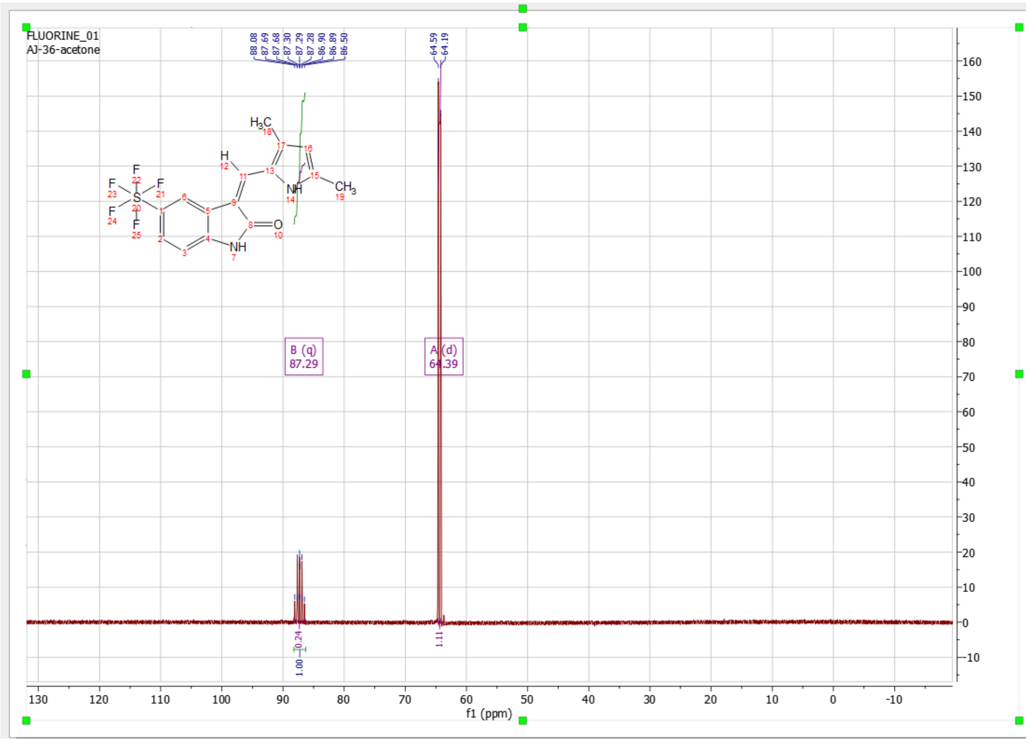

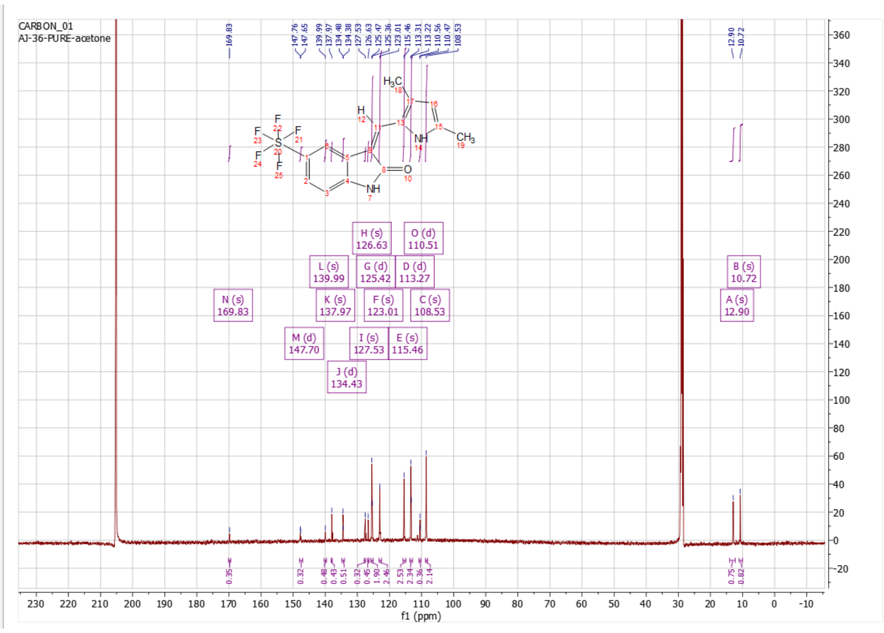

CARBON_01
AJ-36-PURE-acetone
N (s) 169.83
M (d) 147.70
L (s) 139.99
K (s) 137.97
J (d) 134.43
I (s) 127.53
H (s) 126.63
G (d) 125.42
F (s) 123.01
E (s) 115.46
D (d) 113.27
C (s) 108.53
O (d) 110.51
B (s) 10.72
A (s) 12.90
f1 (ppm)

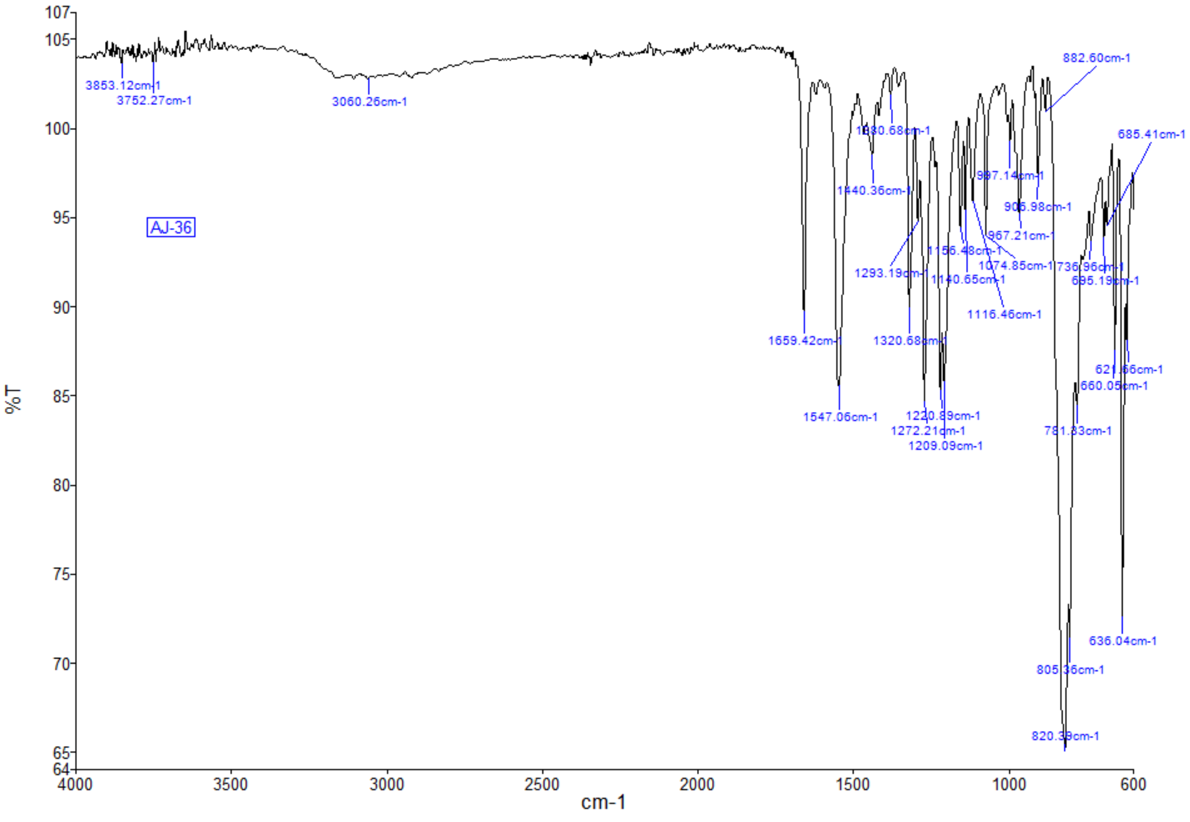

AJ-36
%T
cm-1
3853.12cm-1
3752.27cm-1
3060.26cm-1
1659.42cm-1
1547.06cm-1
1440.36cm-1
1320.68cm-1
1293.19cm-1
1272.21cm-1
1220.89cm-1
1209.09cm-1
1156.48cm-1
1140.65cm-1
1116.46cm-1
1074.85cm-1
987.14cm-1
967.21cm-1
905.98cm-1
882.60cm-1
820.39cm-1
805.36cm-1
781.83cm-1
736.96cm-1
695.19cm-1
685.41cm-1
660.05cm-1
636.04cm-1
621.66cm-1

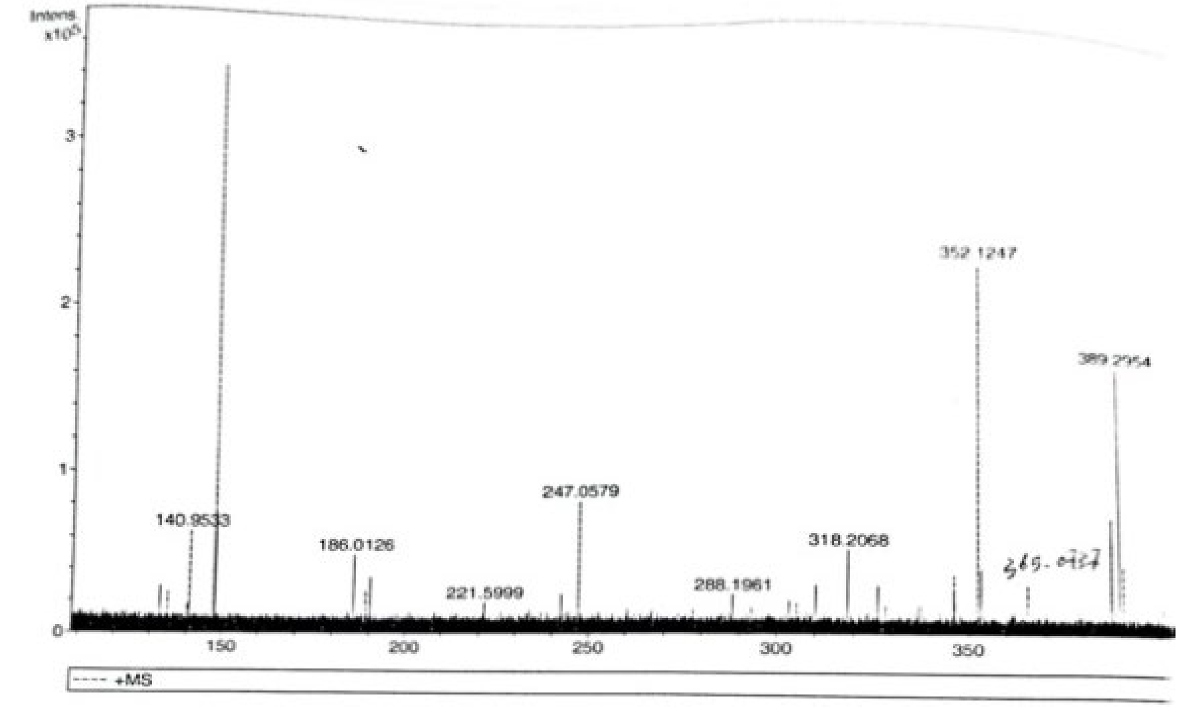

Sum Formula	Sigma	m/z	Err [ppm]	Mean Err [ppm]	Err [mDa]	rdb	N Rule	e⁻
C 15 H 14 F 5 N 2 O 1 S 1	0.195	365.0742	1.33	-110.19	-40.29	7.50	ok	even

5-(Pentafluoro-□^{6}-sulfanyl)-3-(propan-2-ylidene)-2,3-dihydro-1H-indol-2-one (8b)

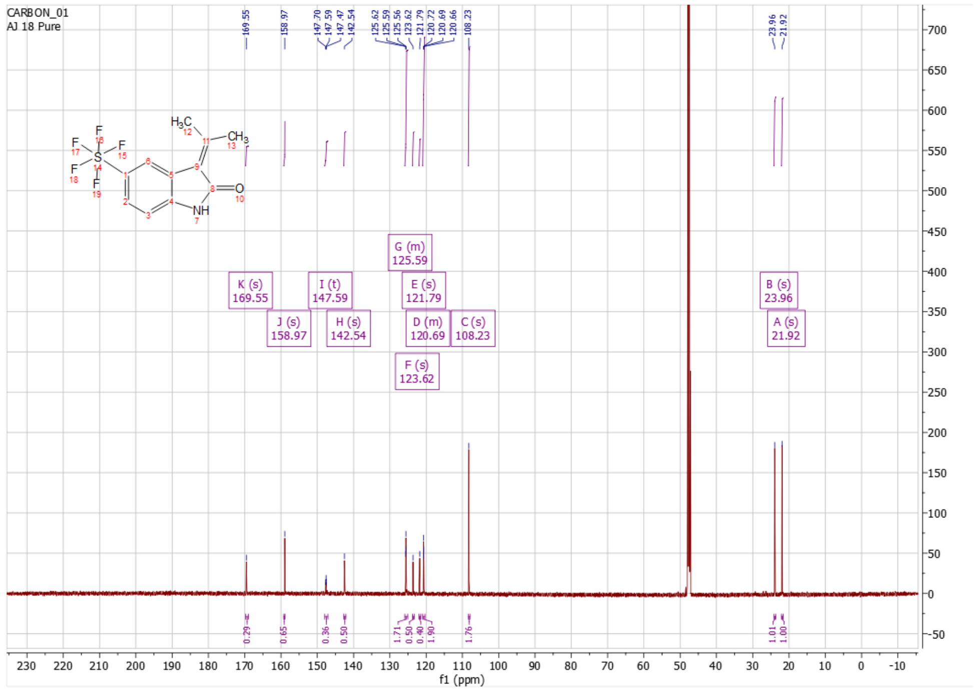

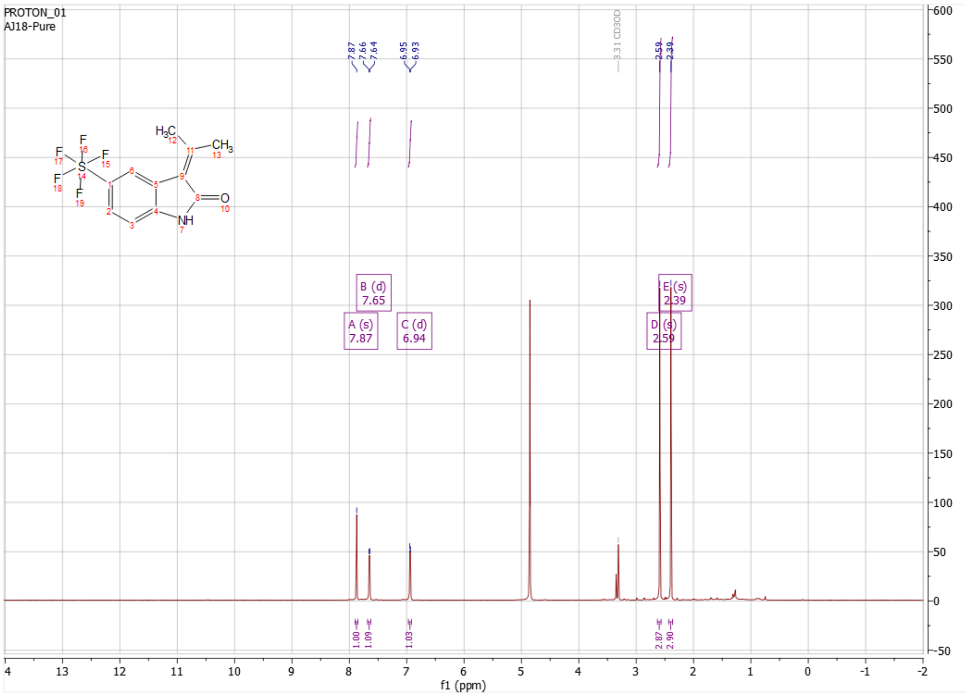
PROTON_01
AJ18-Pure
7.87
7.66
7.64
6.95
6.93
3.31 CD3OD
2.59
2.39
A (s)
7.87
B (d)
7.65
C (d)
6.94
D (s)
2.59
E (s)
2.39
1.00
1.09
1.03
2.87
2.90
f1 (ppm)

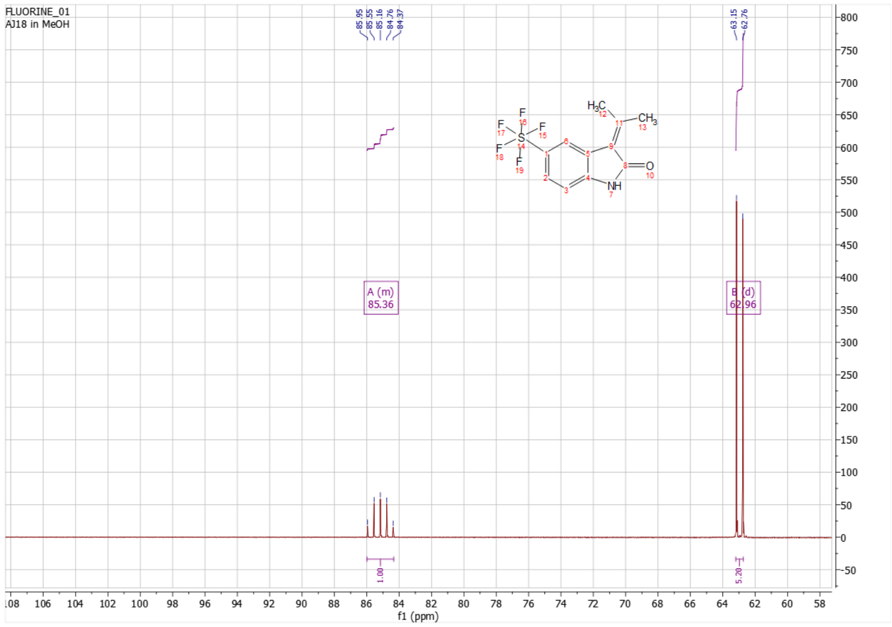
FLUORINE_01
AJ18 in MeOH
85.95
85.55
85.16
84.76
84.37
63.15
62.76
A (m)
85.36
B (d)
62.96
1.00
5.20
f1 (ppm)

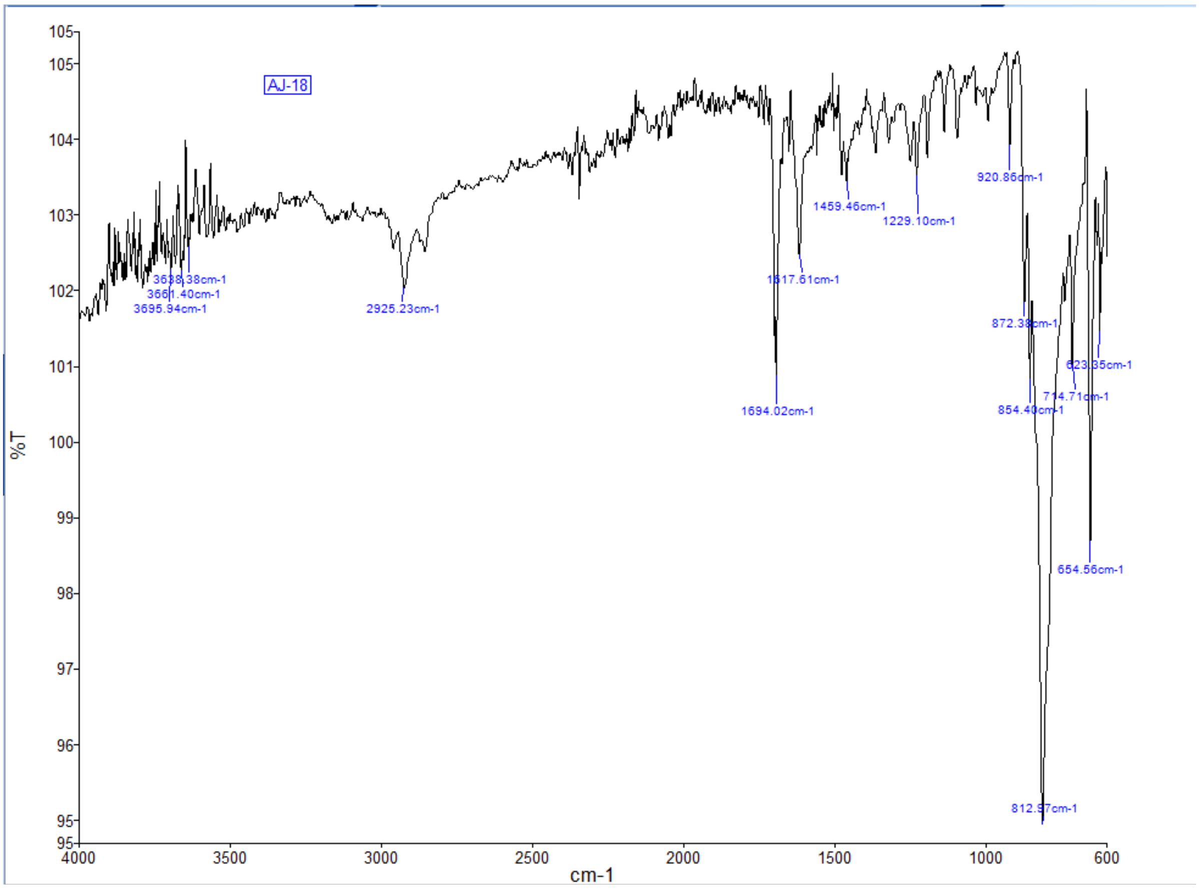
AJ-18
%T
cm-1
3638.38cm-1
3661.40cm-1
3695.94cm-1
2925.23cm-1
1694.02cm-1
1617.61cm-1
1459.46cm-1
1229.10cm-1
920.86cm-1
872.38cm-1
854.40cm-1
823.35cm-1
714.71cm-1
654.56cm-1
812.97cm-1

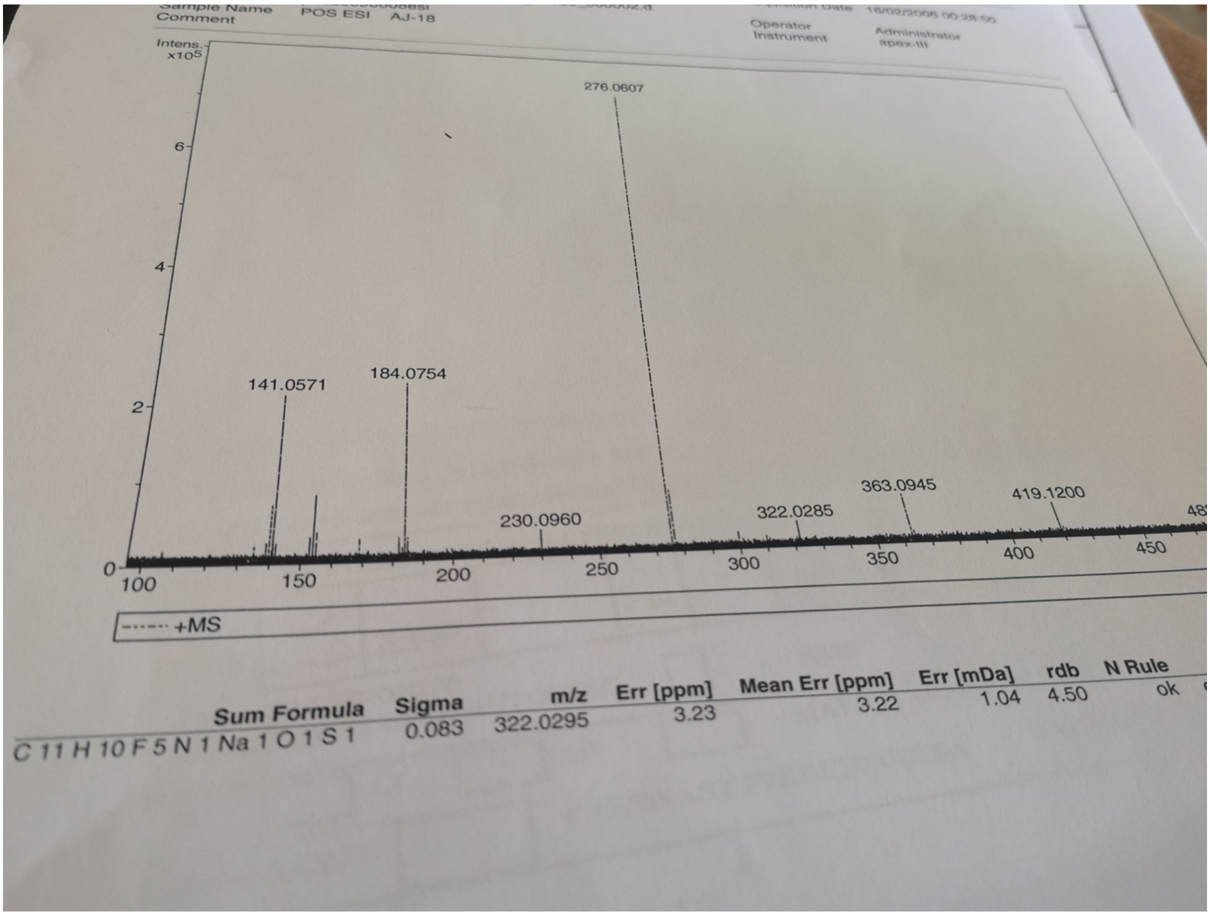
Comment
POS ESI
AJ-18
Operator
Instrument
Administrator
Intens.
x10^5
276.0607
141.0571
184.0754
230.0960
322.0285
363.0945
419.1200
+MS
Sum Formula
Sigma
m/z
Err [ppm]
Mean Err [ppm]
Err [mDa]
rdb
N Rule
C 11 H 10 F 5 N 1 Na 1 O 1 S 1
0.083
322.0295
3.23
3.22
1.04
4.50
ok

(3Z)-3-[(3,5-Dimethyl-1H-pyrrol-2-yl)methylidene]-6-(pentafluoro-□^{6}-sulfanyl)-2,3-dihydro-1H-indol-2-one (9a)

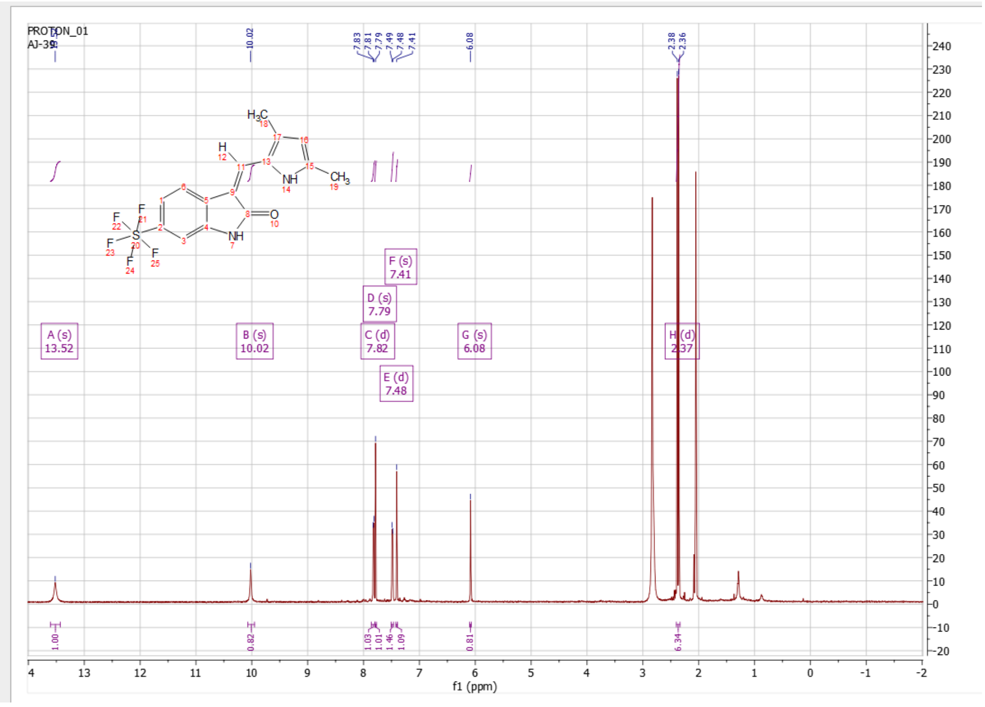

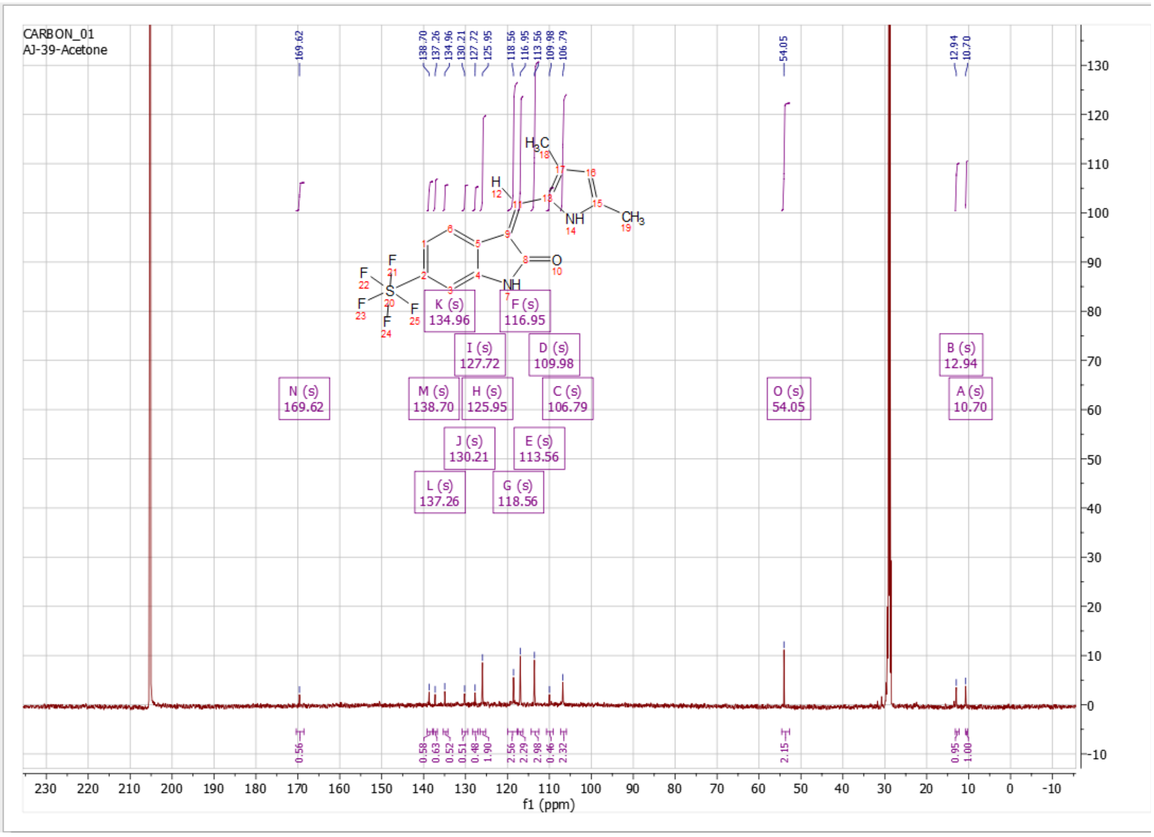

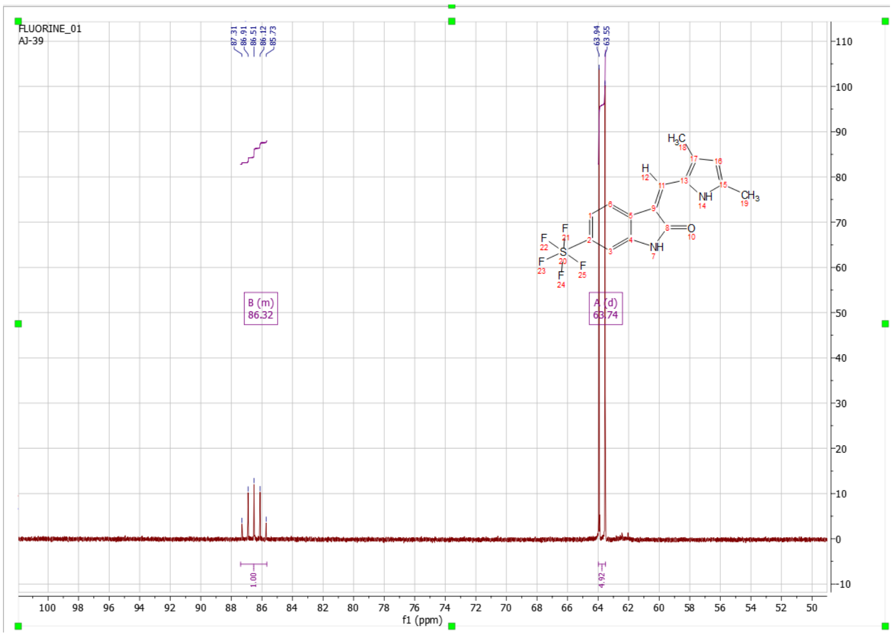

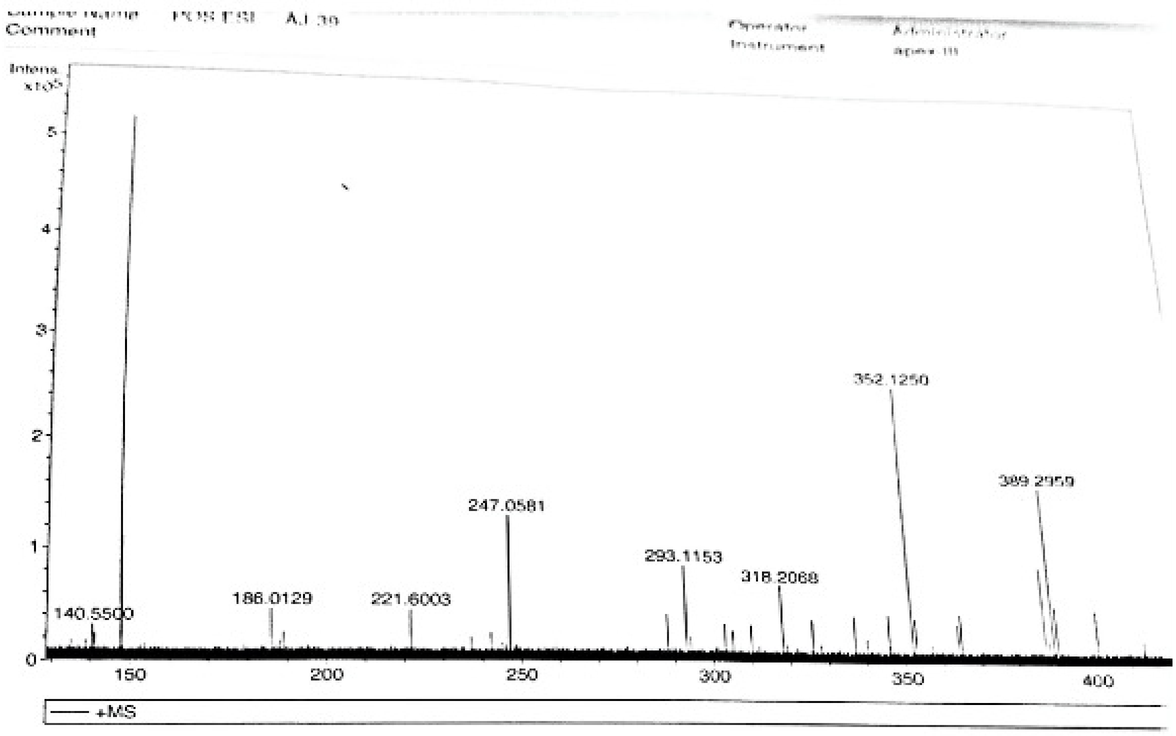

Sum Formula	Sigma	m/z	Err [ppm]	Mean Err [ppm]	Err [mDa]	rdb	N Rule	e⁻
C 15 H 14 F 5 N 2 O 1 S 1	0.095	365.0742	-2.99	-2.99	-1.09	7.50	ok	even

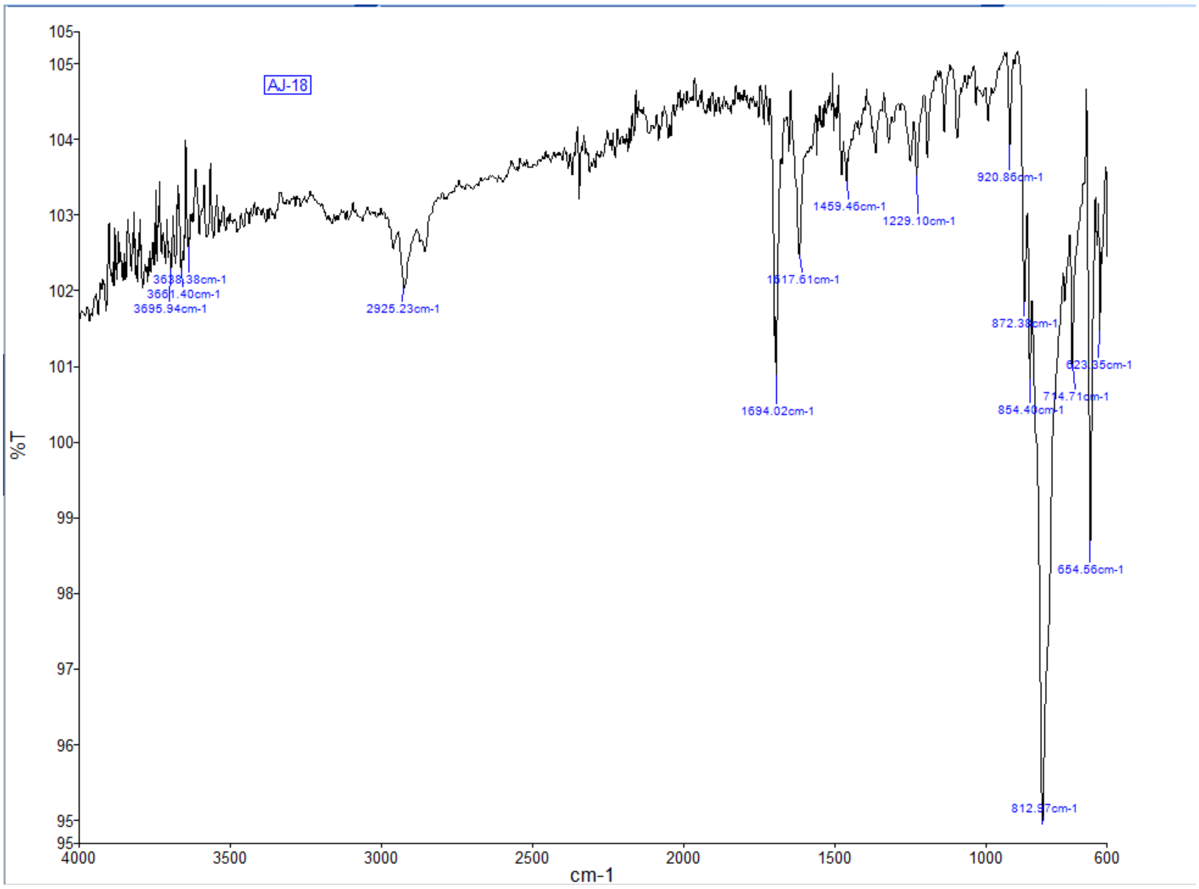

5-Nitro-3-(propan-2-ylidene)-2,3-dihydro-1H-indol-2-one (10a)

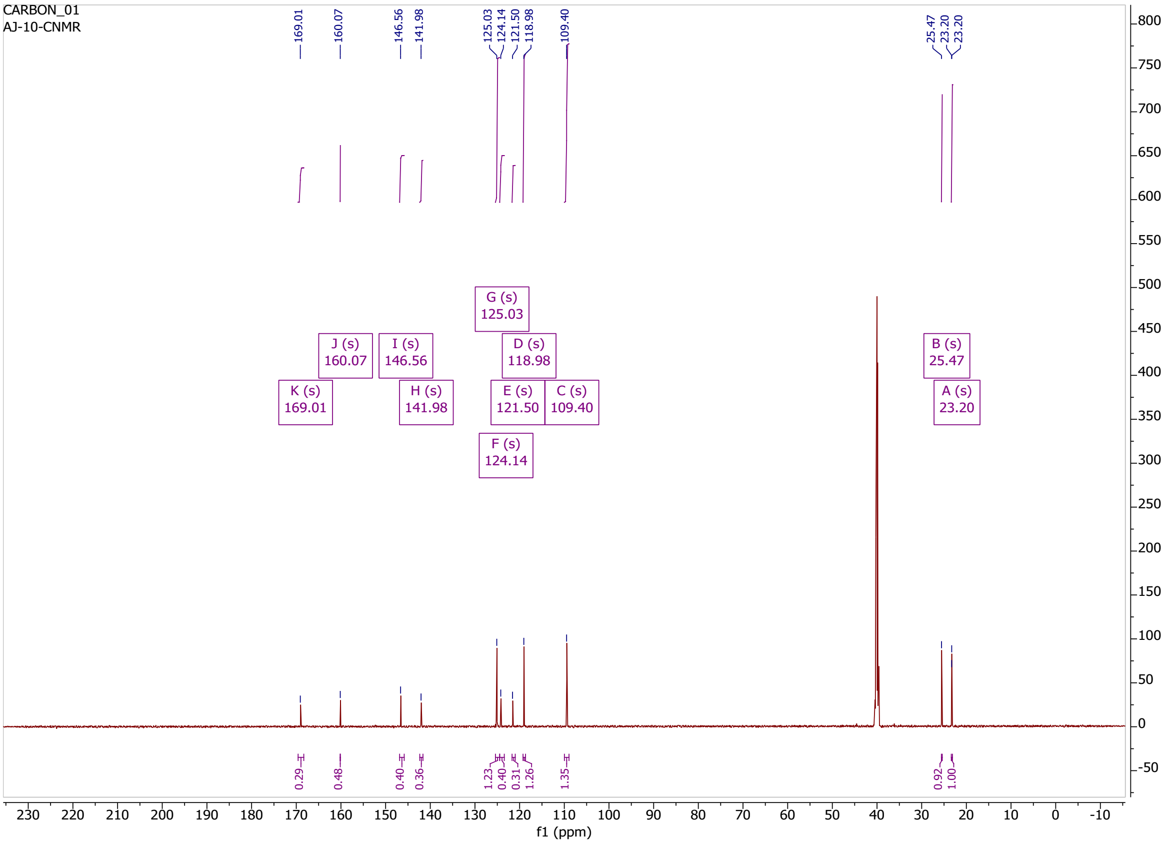

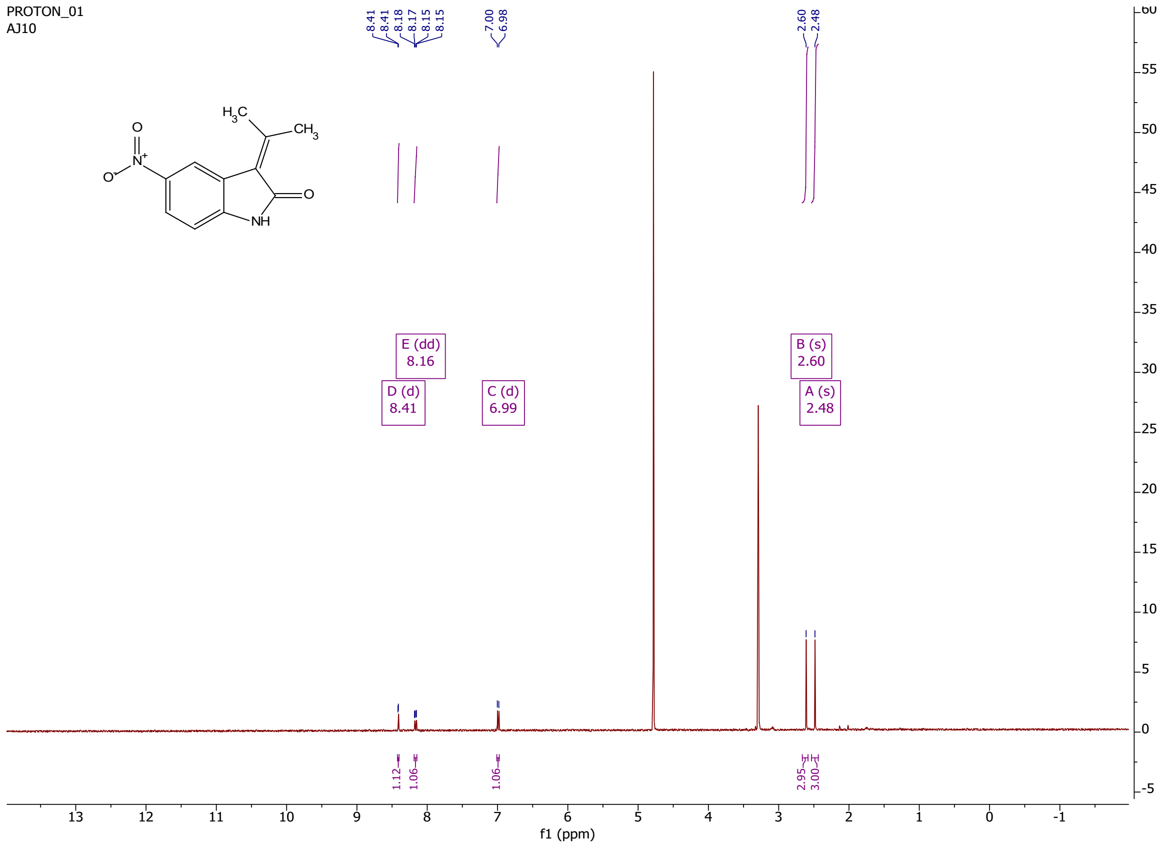

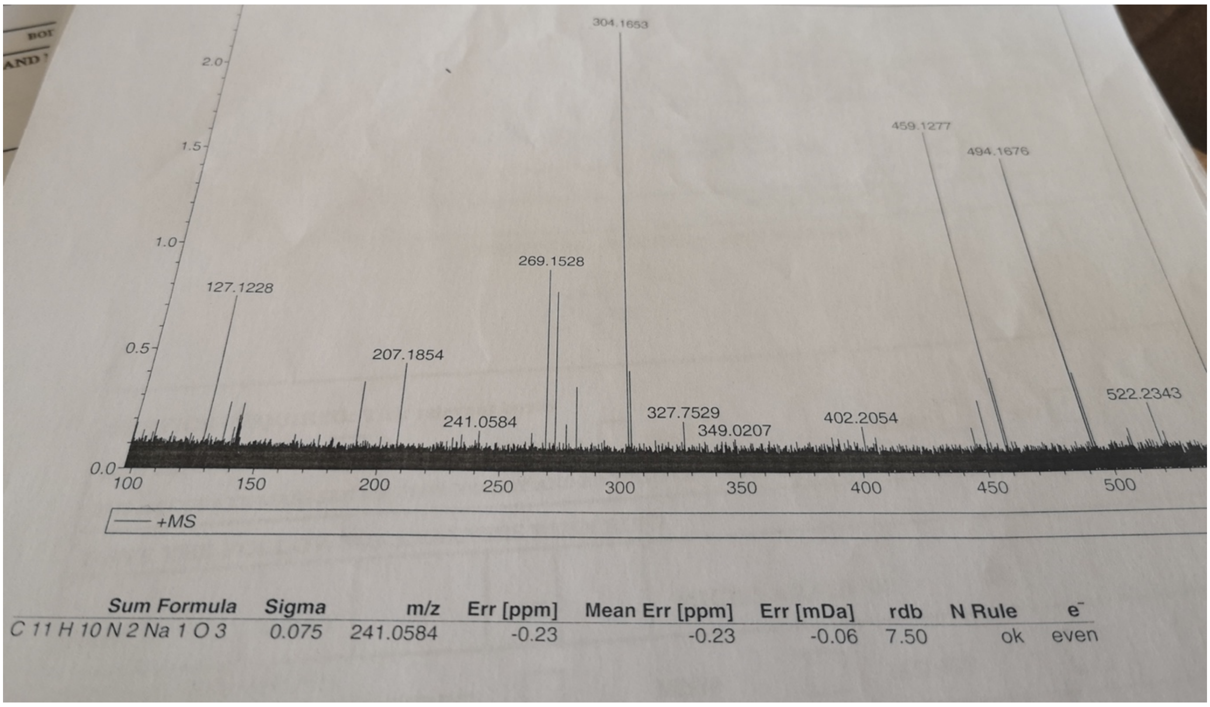

5-(Pentafluoro-□^{6}-sulfanyl)-1,2-dihydrospiro[indole-3,4'-oxan]-2-one (11a)

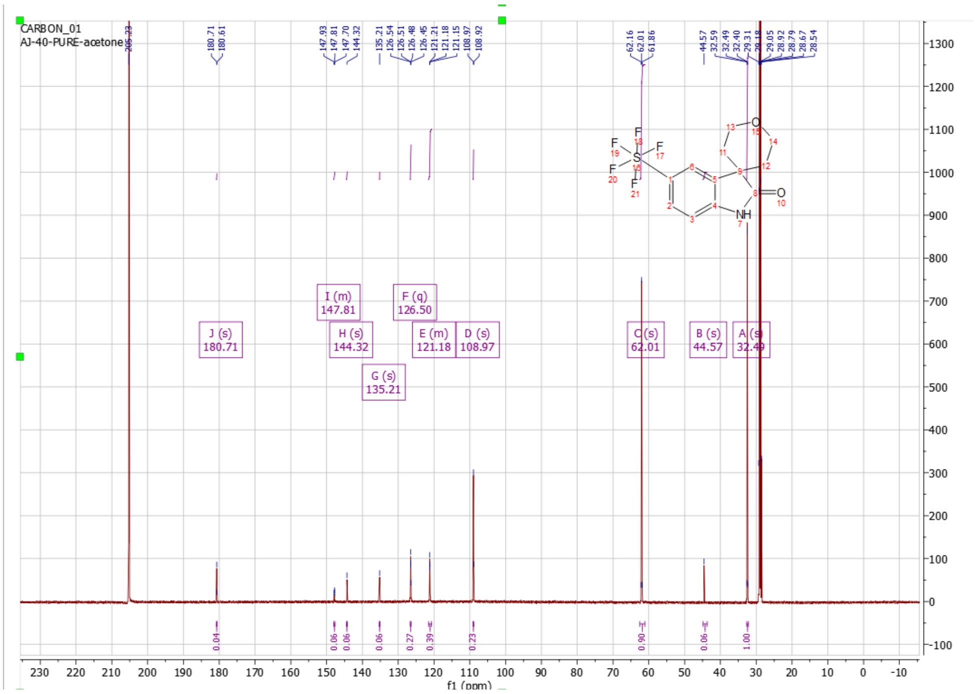
CARBON_01
AJ-40-PURE-acetone
J (s) 180.71
I (m) 147.81
H (s) 144.32
G (s) 135.21
F (q) 126.50
E (m) 121.18
D (s) 108.97
C (s) 62.01
B (s) 44.57
A (s) 32.49
f1 (ppm)

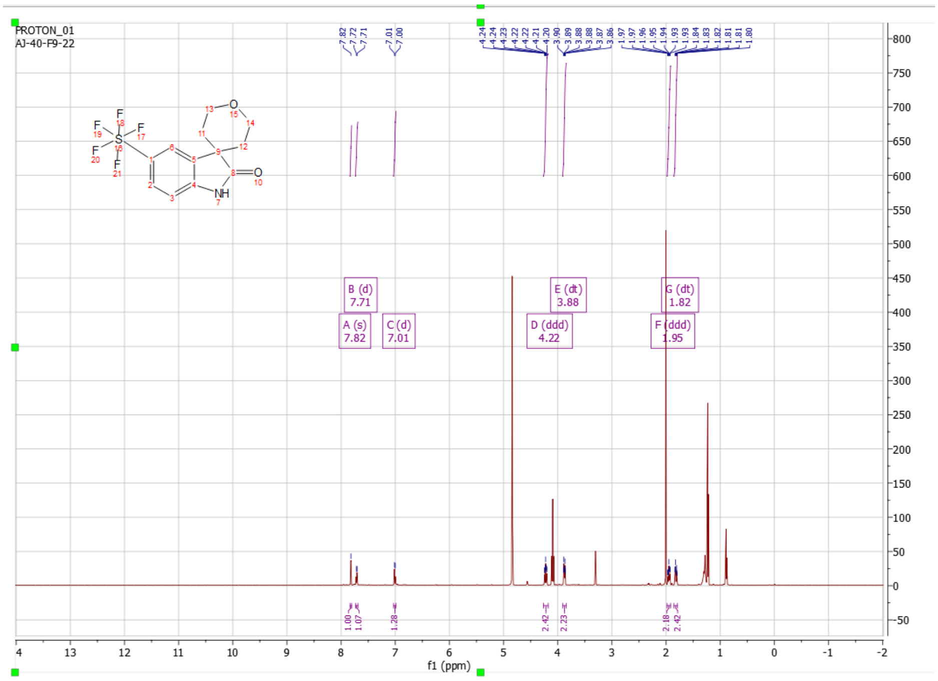
PROTON_01
AJ-40-F9-22
B (d) 7.71
A (s) 7.82
C (d) 7.01
E (dt) 3.88
D (ddd) 4.22
G (dt) 1.82
F (ddd) 1.95
f1 (ppm)

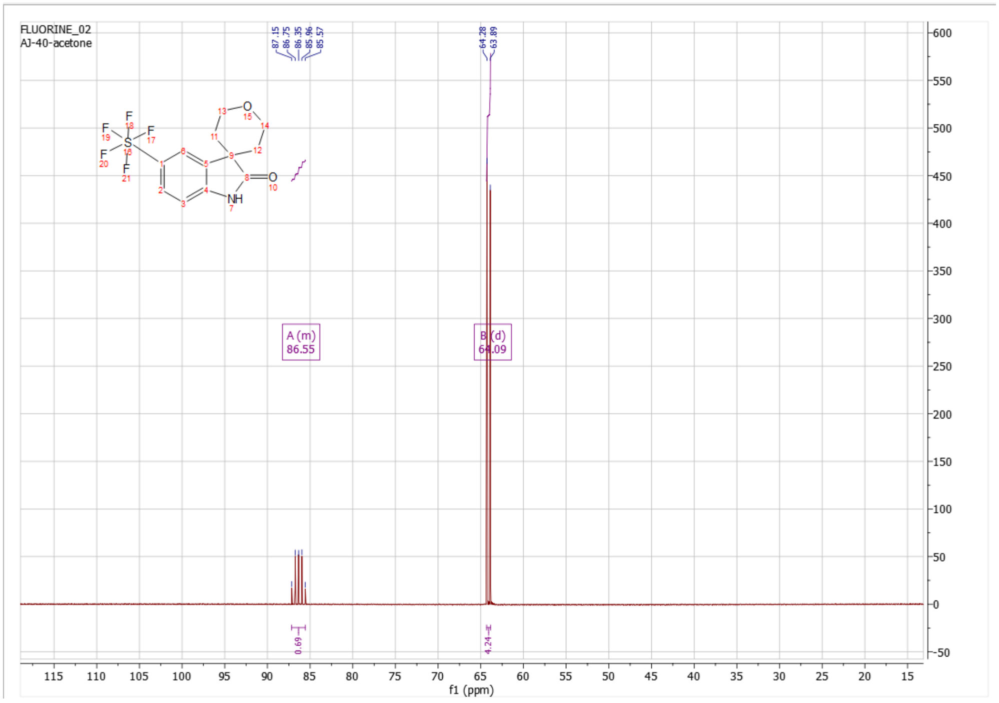

Single Mass Analysis
Tolerance = 50.0 PPM / DBE: min = -1.5, max = 50.0
Selected filters: None

Page 1

Monoisotopic Mass, Odd and Even Electron Ions
13 formula(e) evaluated with 1 results within limits (all results (up to 1000) for each mass)
Elements Used:
C: 0-12 H: 0-1000 N: 0-1 O: 0-2 F: 5-5 S: 0-1
AJ-40
ARATHY10433A 38 (2.975)

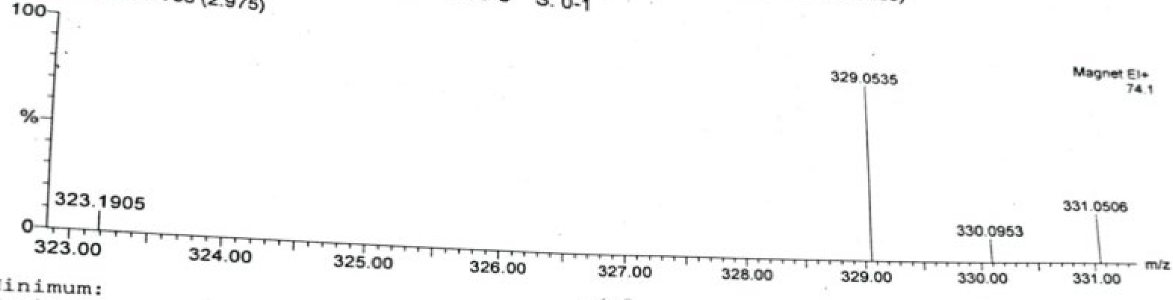

Mass	Calc. Mass	mDa	PPM	DBE	i-FIT	Formula
Minimum:				-1.5		
Maximum:		5.0	50.0	50.0		
329.0535	329.0509	2.6	7.9	5.0	7.1	C12 H12 N O2 F5 S

1-methyl-5-(pentafluoro-□⁶-sulfanyl)-1,2-dihydrospiro[indole-3,4'-oxan]-2-one (11b)

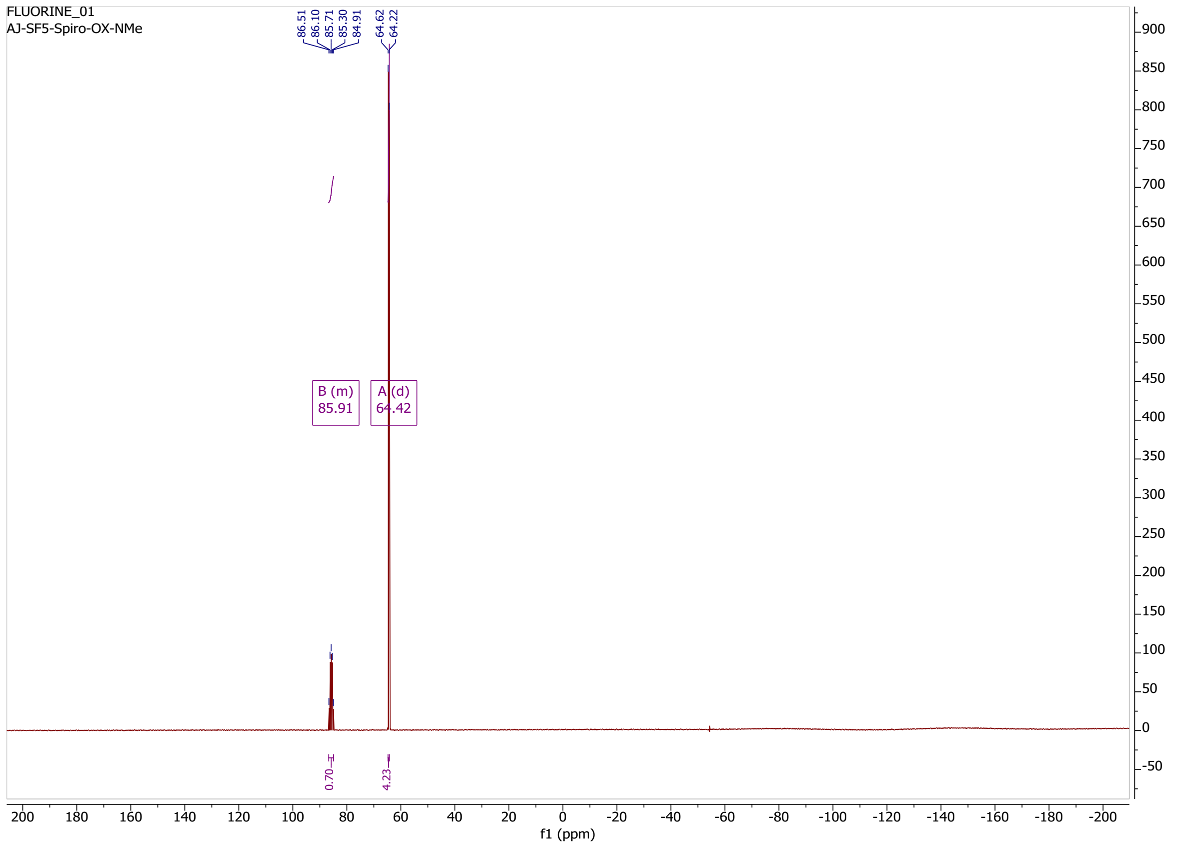
FLUORINE_01
AJ-SF5-Spiro-OX-NMe
86.51
86.10
85.71
85.30
84.91
64.62
64.22
B (m)
85.91
A (d)
64.42
0.70
4.23
f1 (ppm)

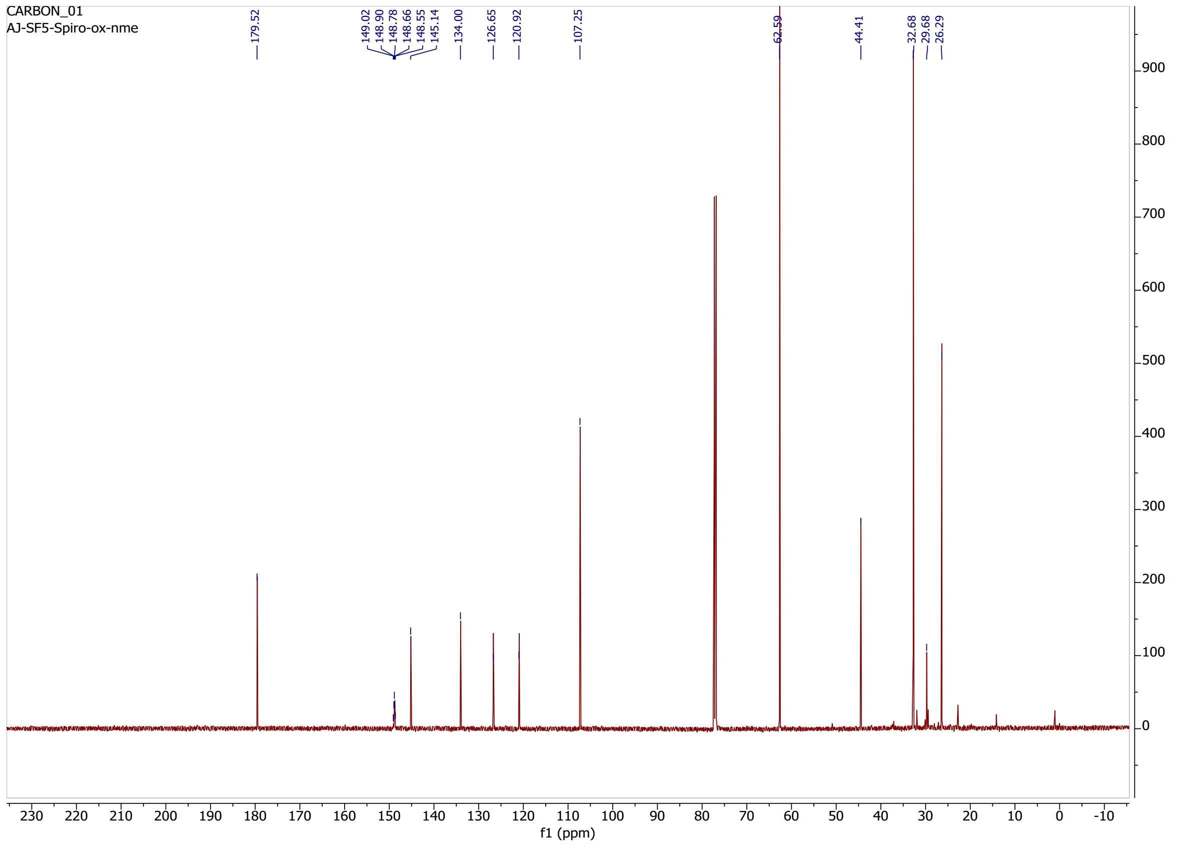
CARBON_01
AJ-SF5-Spiro-ox-nme
179.52
149.02
148.90
148.78
148.66
148.55
145.14
134.00
126.65
120.92
107.25
62.59
44.41
32.68
29.68
26.29
f1 (ppm)

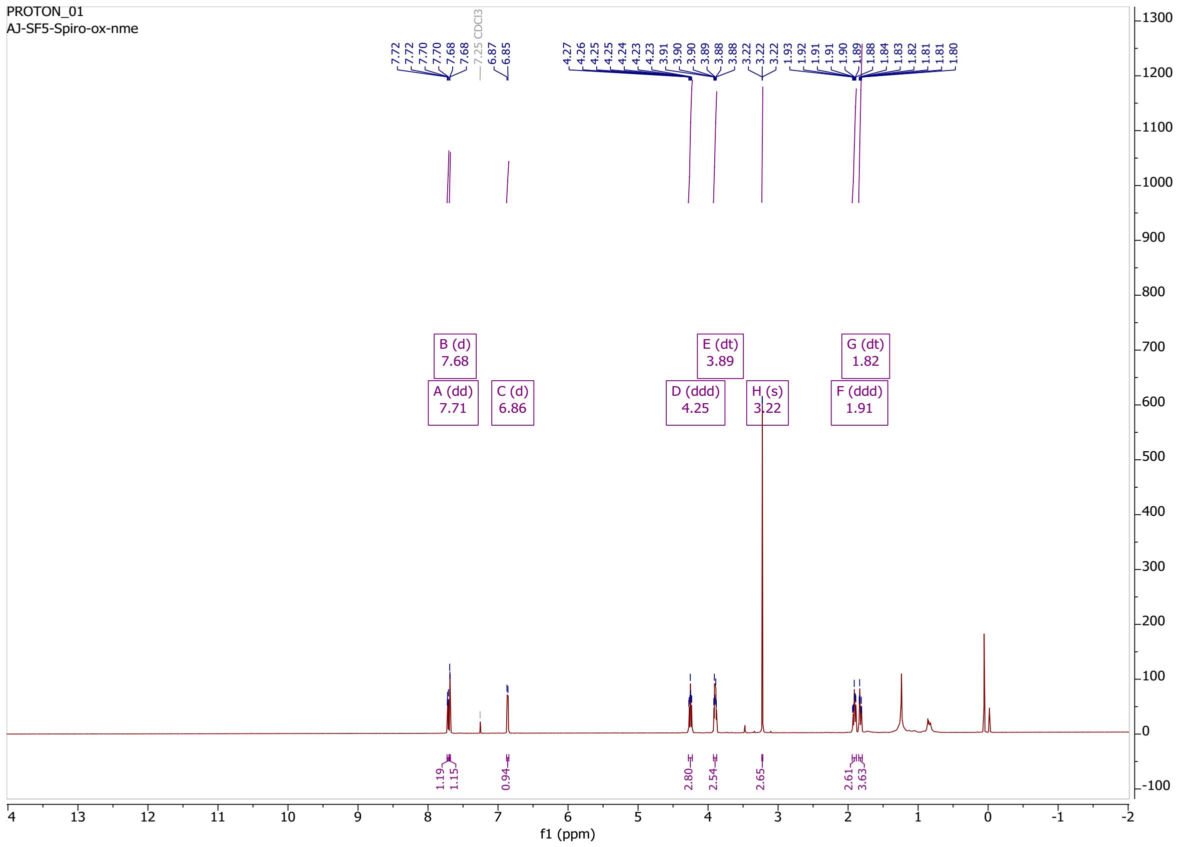

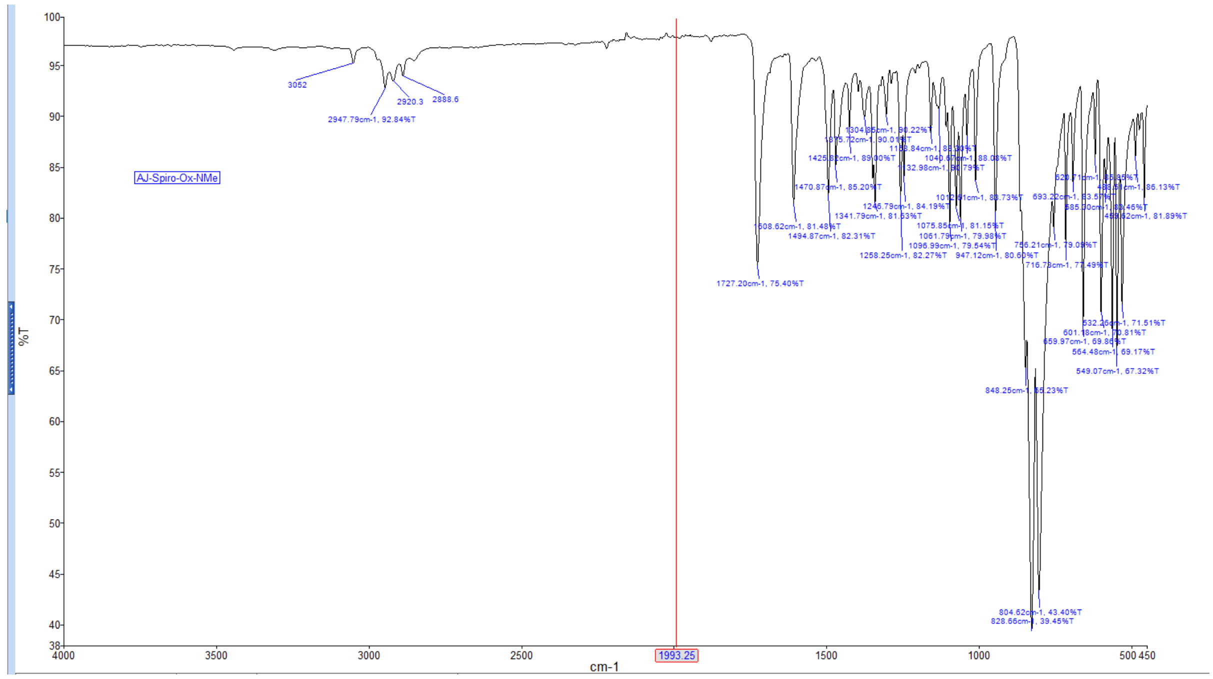

$C_{13}H_{14}F_5NO_2S$ $[M+H]^+$ calc:344.0744; observed:344.0743; Accuracy: -0.3

2-{[3-(Trifluoromethyl)phenyl]methyl}-3H,4H-pyrido[3,4-d]pyrimidin-4-one (16a)

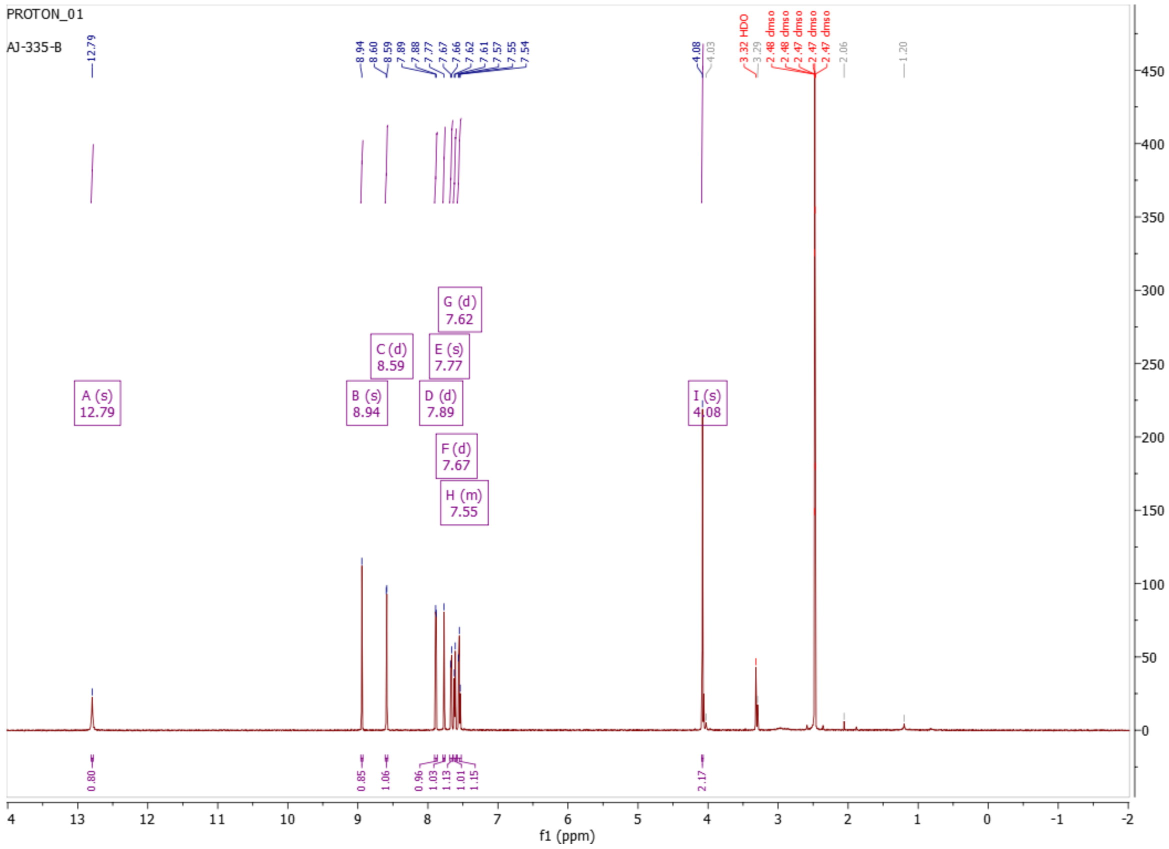
PROTON_01
AJ-335-B
A (s)
12.79
B (s)
8.94
C (d)
8.59
D (d)
7.89
E (s)
7.77
F (d)
7.67
G (d)
7.62
H (m)
7.55
I (s)
4.08
f1 (ppm)

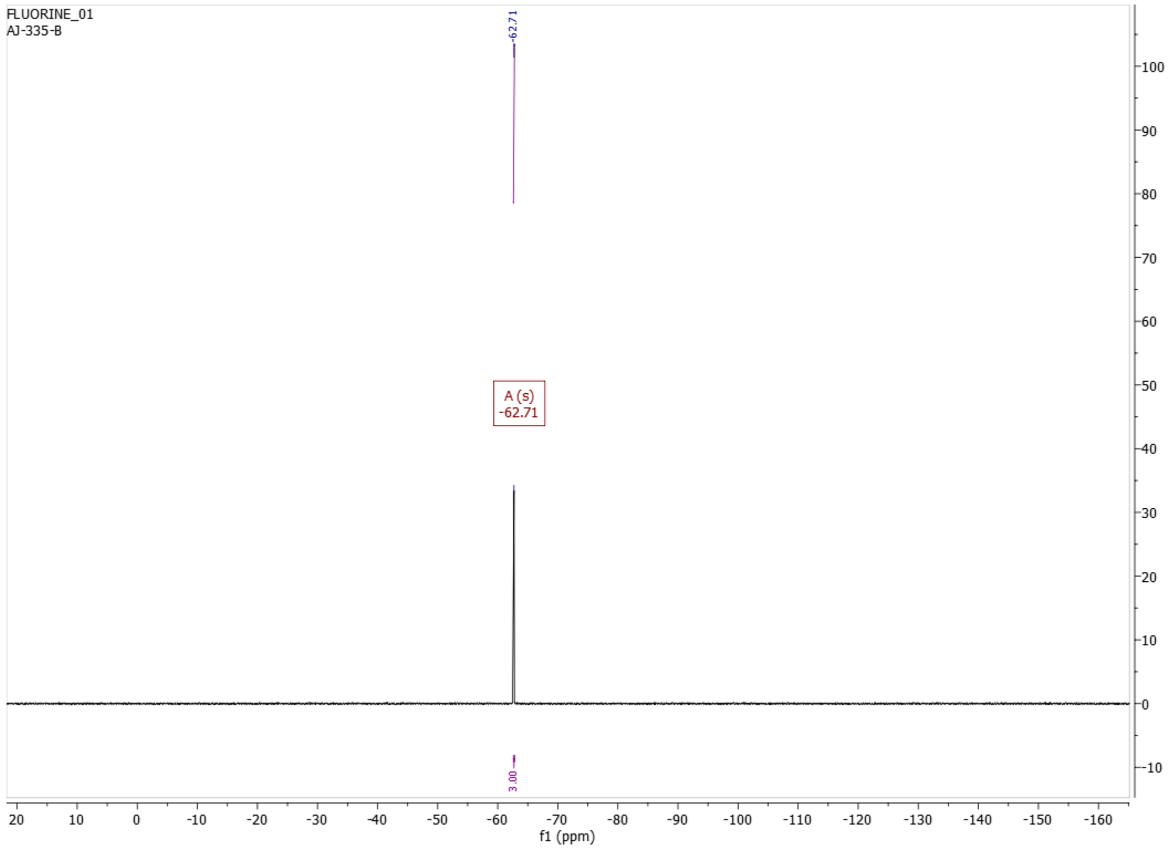
FLUORINE_01
AJ-335-B
A (s)
-62.71
f1 (ppm)

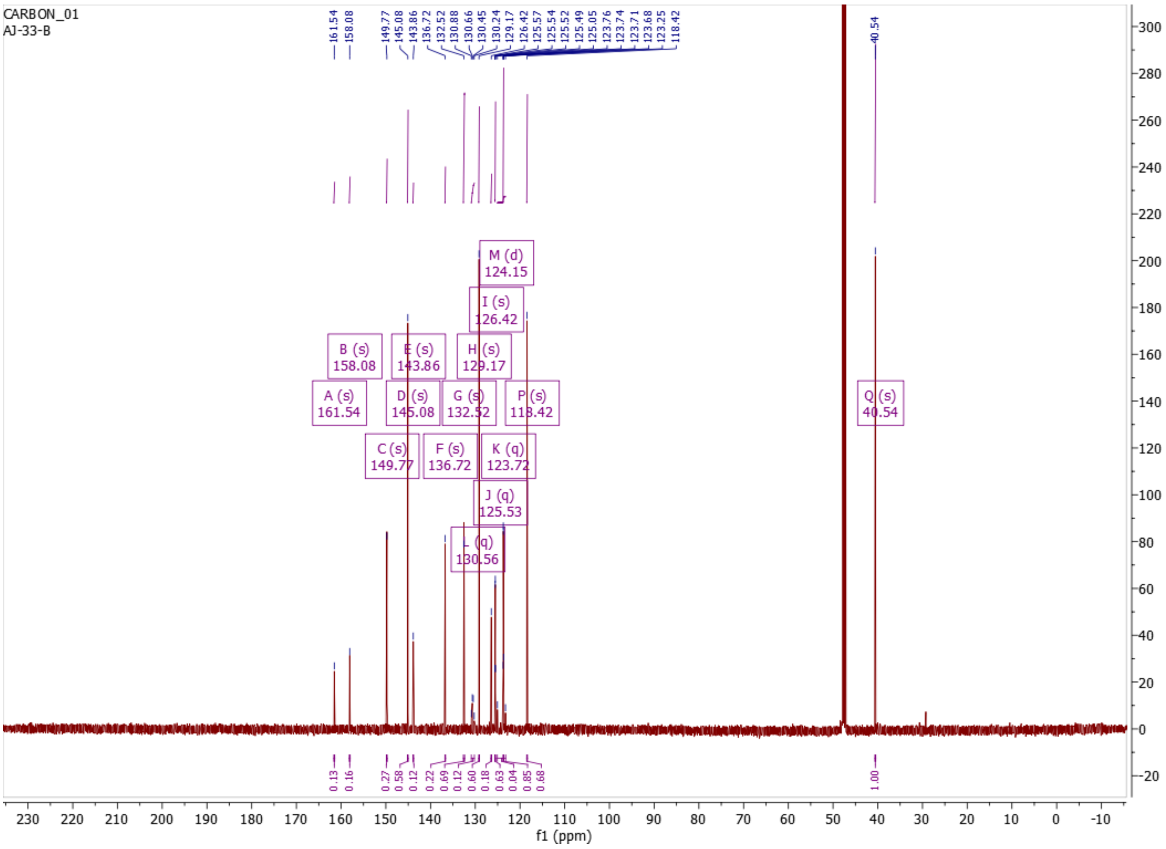

Elemental Composition Report | **Page 1**

Single Mass Analysis
Tolerance = 200.0 PPM / DBE: min = -1.5, max = 50.0
Element prediction: Off

Monoisotopic Mass, Even Electron Ions
1 formula(e) evaluated with 1 results within limits (up to 50 closest results for each mass)
Elements Used:
C: 15-15 H: 0-100 N: 3-3 O: 1-1 F: 3-3
AJ-CF3-MPRO
ARATHY11631 83 (1.759) | 1: TOF MS ES+ 5.78e+005

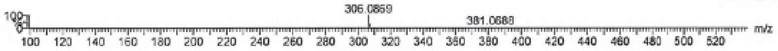

Minimum:				-1.5	
Maximum:		5.0	200.0	50.0	
Mass	Calc. Mass	mDa	PPM	DBE	Formula
306.0869	306.0854	1.5	4.9	10.5	C15 H11 N3 O F3

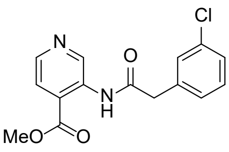

13c

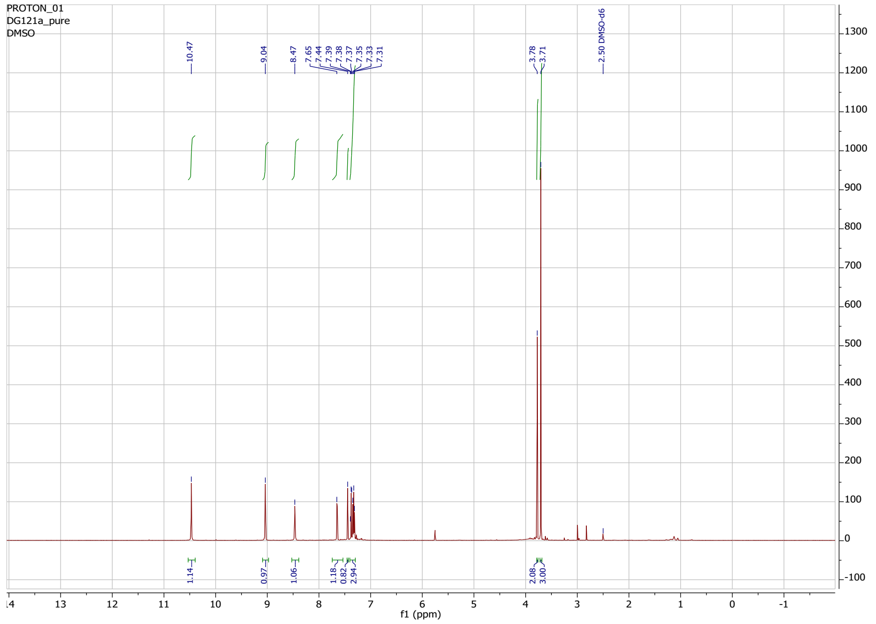
PROTON_01
DG121a_pure
DMSO
10.47
9.04
8.47
7.65
7.44
7.39
7.38
7.37
7.35
7.33
7.31
3.78
3.71
2.50 DMSO-d6
1.14
0.97
1.06
1.18
0.82
2.94
2.08
3.00
f1 (ppm)

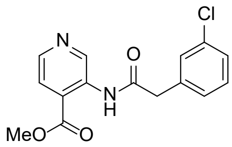
Cl
N
O
N
H
MeO
O

13c

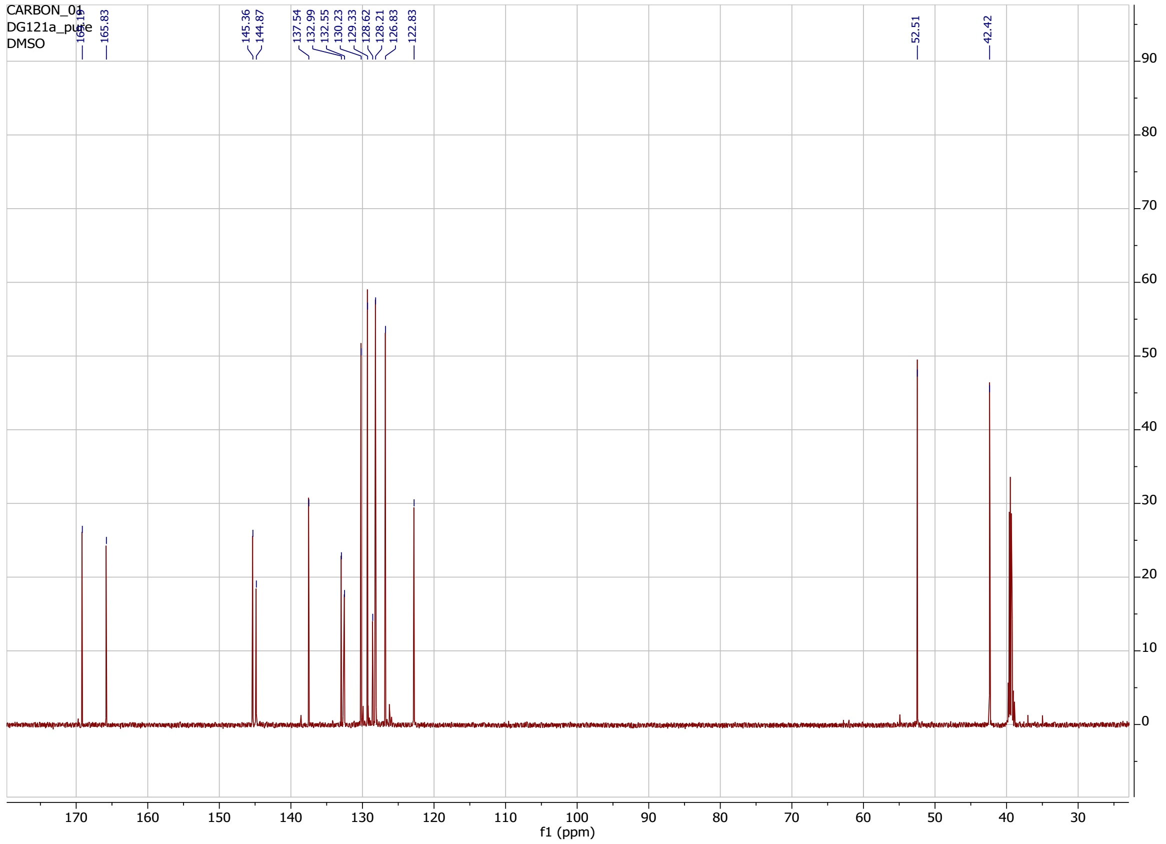
CARBON_01
DG121a_pure
DMSO
165.83
145.36
144.87
137.54
132.99
132.55
130.23
129.33
128.62
128.21
126.83
122.83
52.51
42.42
f1 (ppm)

2-{[3-(Pentafluoro-□^{6}-sulfanyl)phenyl]methyl}-3H,4H-pyrido[3,4-d]pyrimidin-4-one (16b)

E:/AJ-363-B_01/PROTON_01.fid/fid

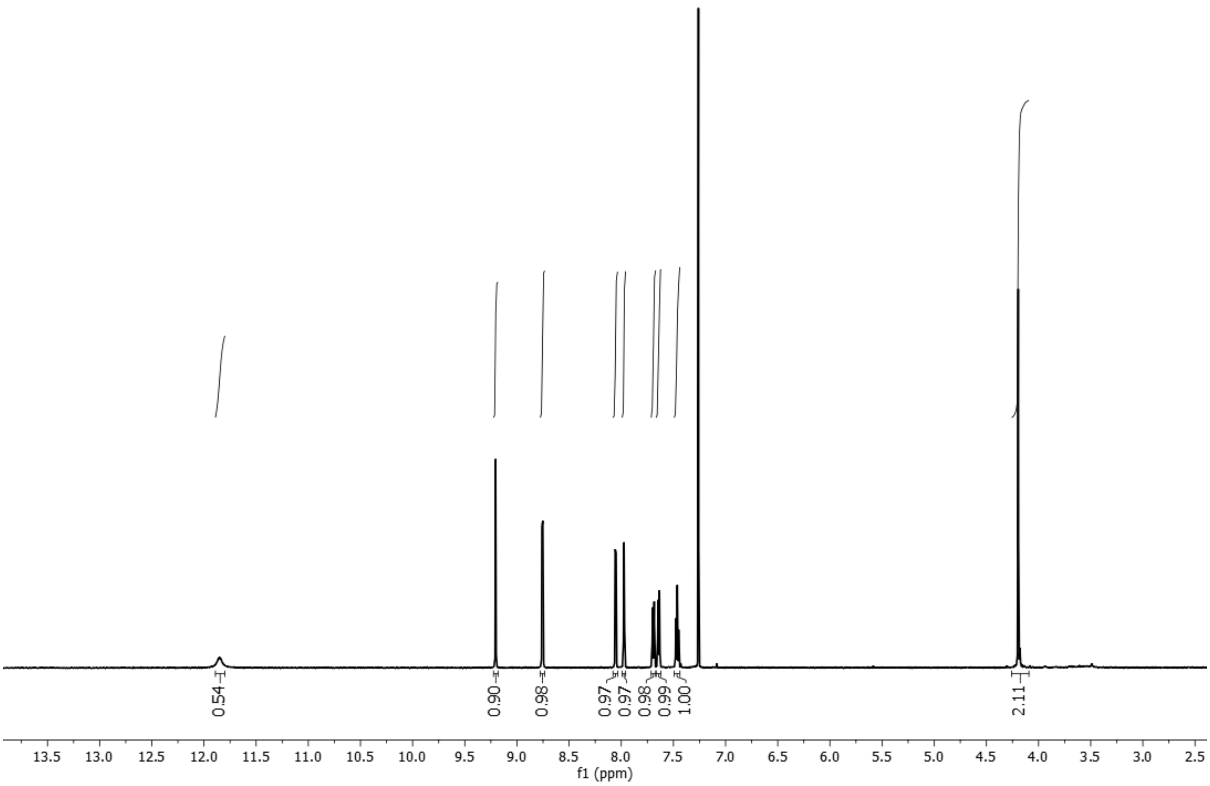

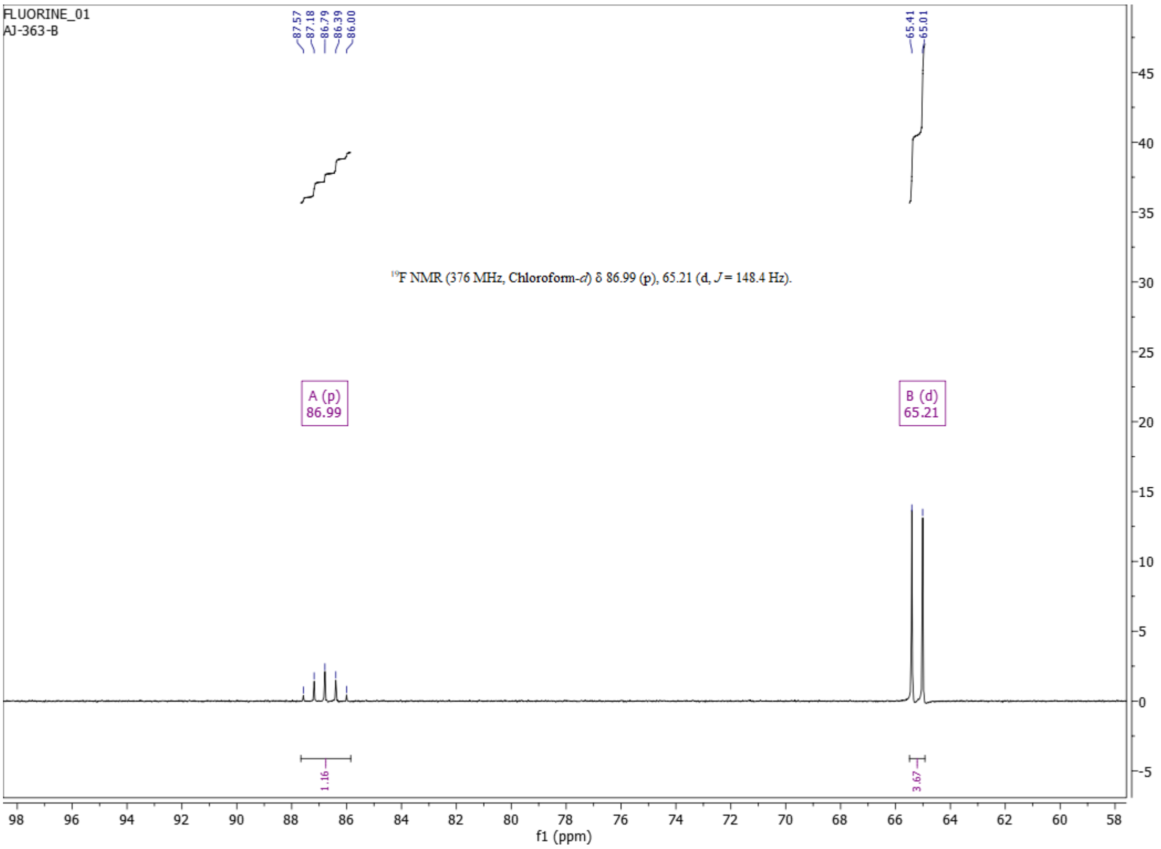

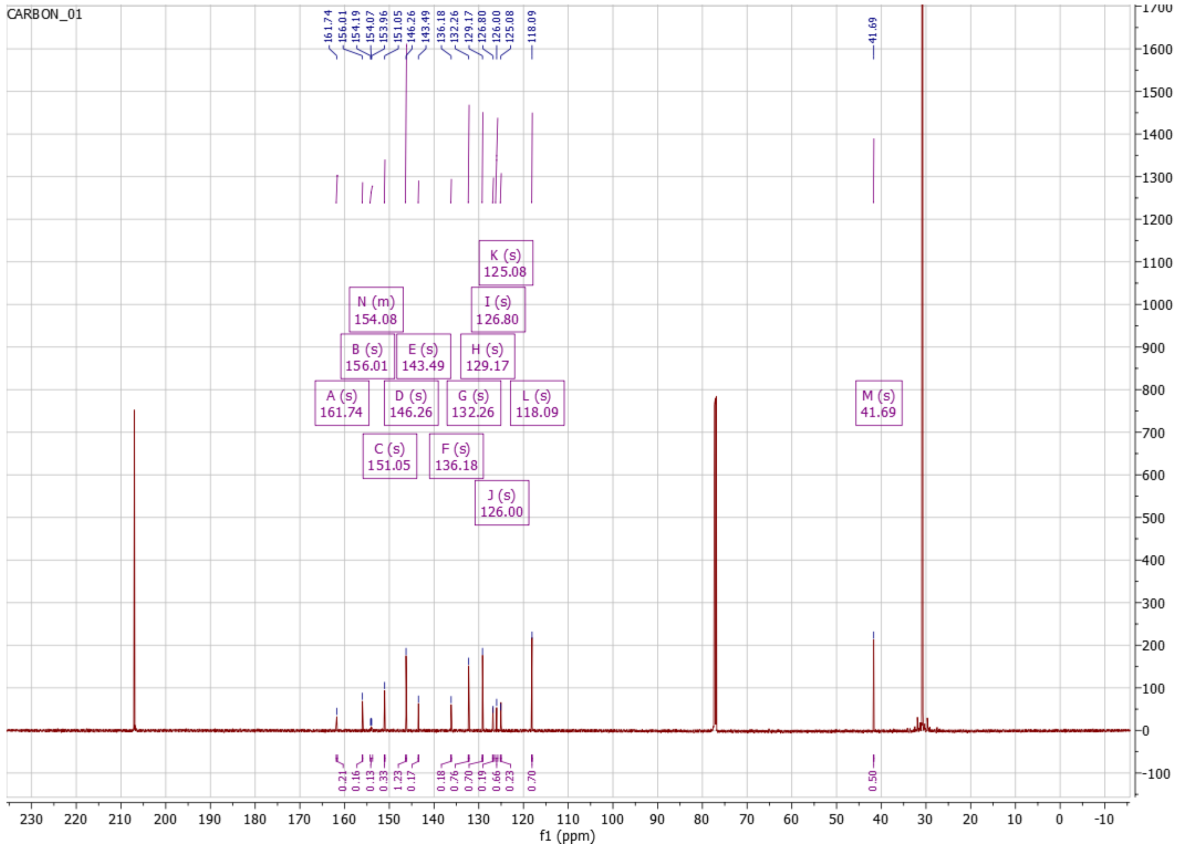

Elemental Composition Report **Page 1**

Single Mass Analysis
Tolerance = 200.0 PPM / DBE: min = -1.5, max = 50.0
Element prediction: Off

Monoisotopic Mass, Even Electron Ions
1 formula(e) evaluated with 1 results within limits (up to 50 closest results for each mass)
Elements Used:
C: 14-14 H: 0-100 N: 3-3 O: 1-1 F: 5-5 S: 1-1
AJ-SF3-MPRO
ARATHY11627 92 (1.810) 1: TOF MS ES+ 2.25e+005

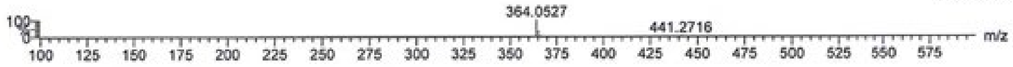

Minimum:				-1.5	
Maximum:		5.0	200.0	50.0	
Mass	Calc. Mass	mDa	PPM	DBE	Formula
364.0527	364.0543	-1.6	-4.4	8.5	C14 H11 N3 O F5 S

5-Methyl-N-[4-(pentafluoro-□^{6}-sulfanyl)phenyl]-1,2-oxazole-4-carboxamide (17)

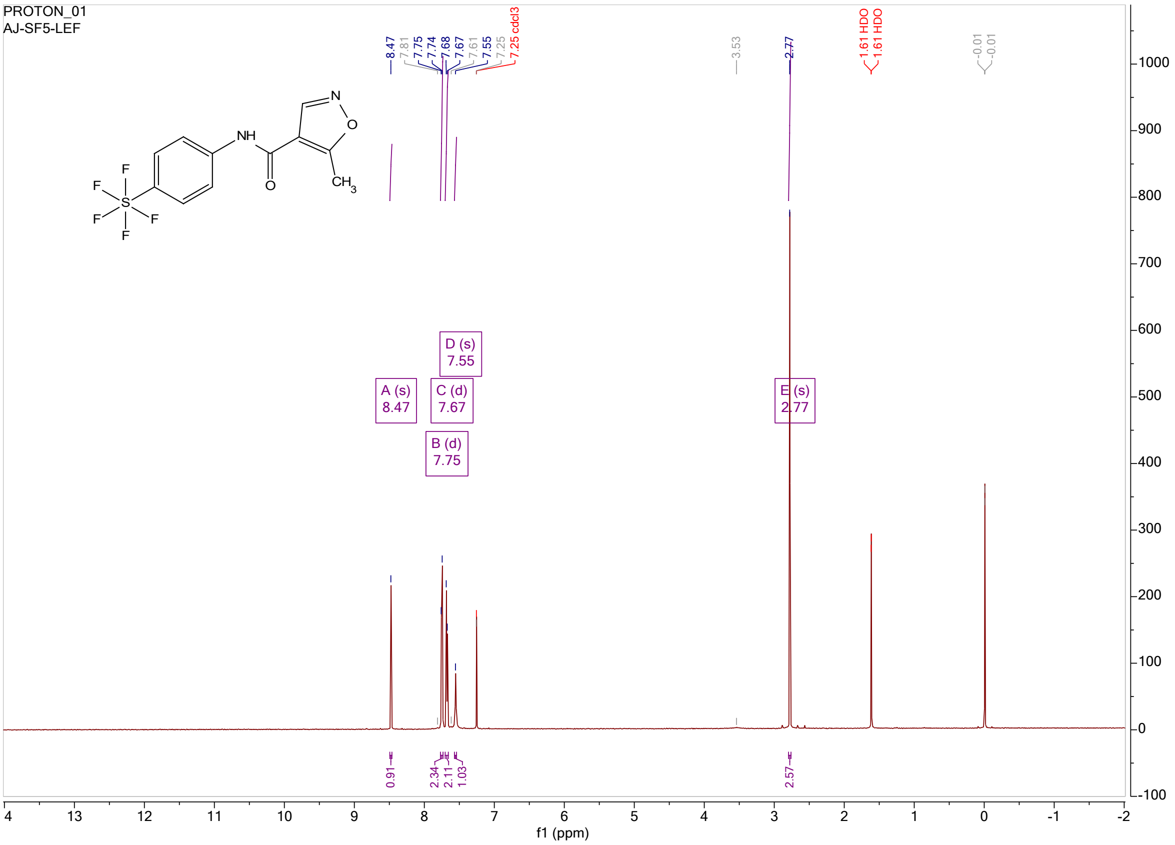
PROTON_01
AJ-SF5-LEF
A (s) 8.47
B (d) 7.75
C (d) 7.67
D (s) 7.55
E (s) 2.77
7.25 cdcl3
1.61 HDO
f1 (ppm)

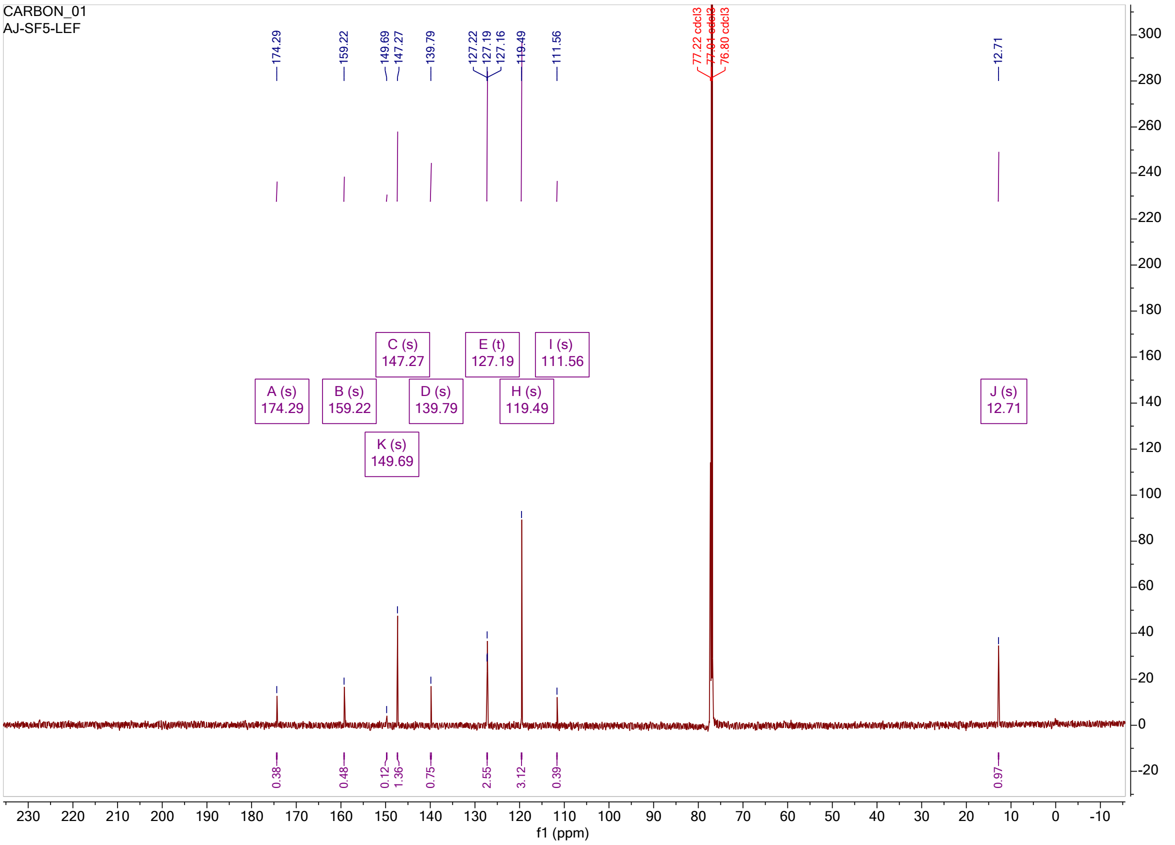
CARBON_01
AJ-SF5-LEF
A (s) 174.29
B (s) 159.22
K (s) 149.69
C (s) 147.27
D (s) 139.79
E (t) 127.19
H (s) 119.49
I (s) 111.56
J (s) 12.71
77.22 cdcl3
76.80 cdcl3
f1 (ppm)

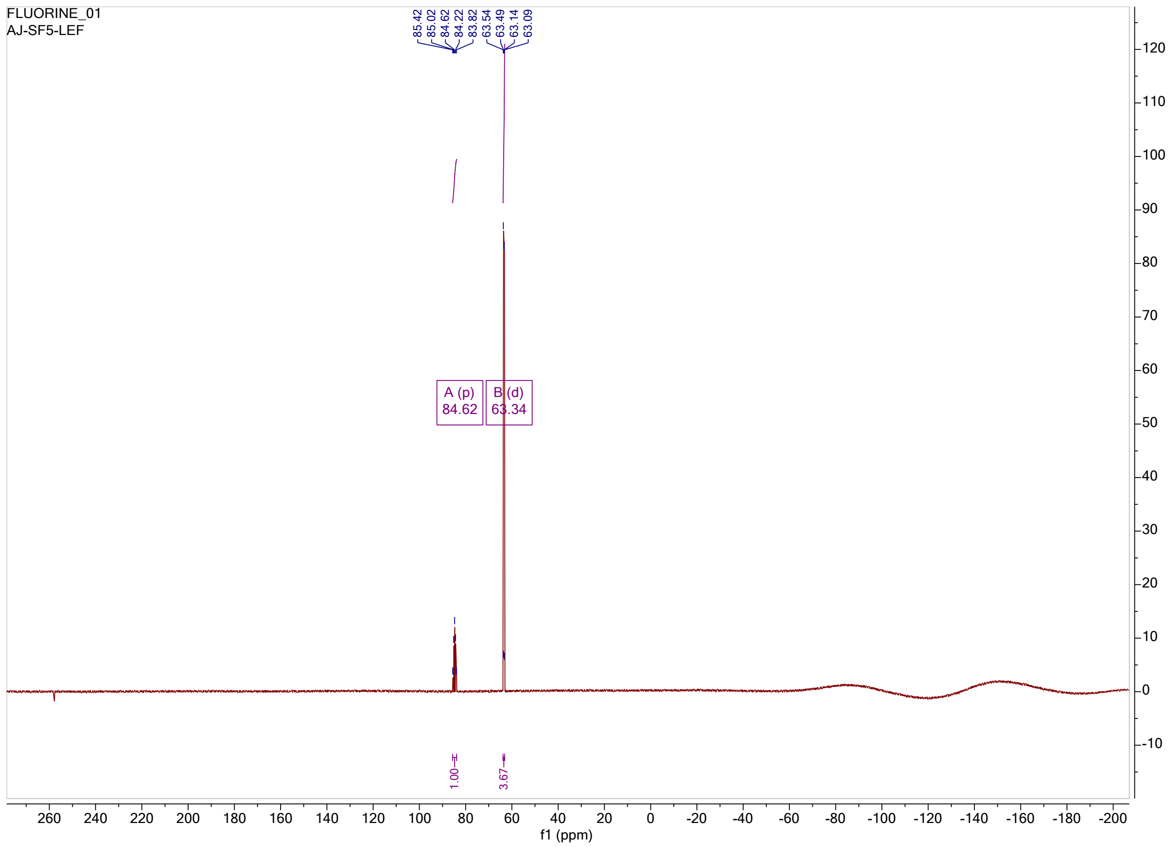
FLUORINE_01
AJ-SF5-LEF
A (p)
84.62
B (d)
63.34
f1 (ppm)

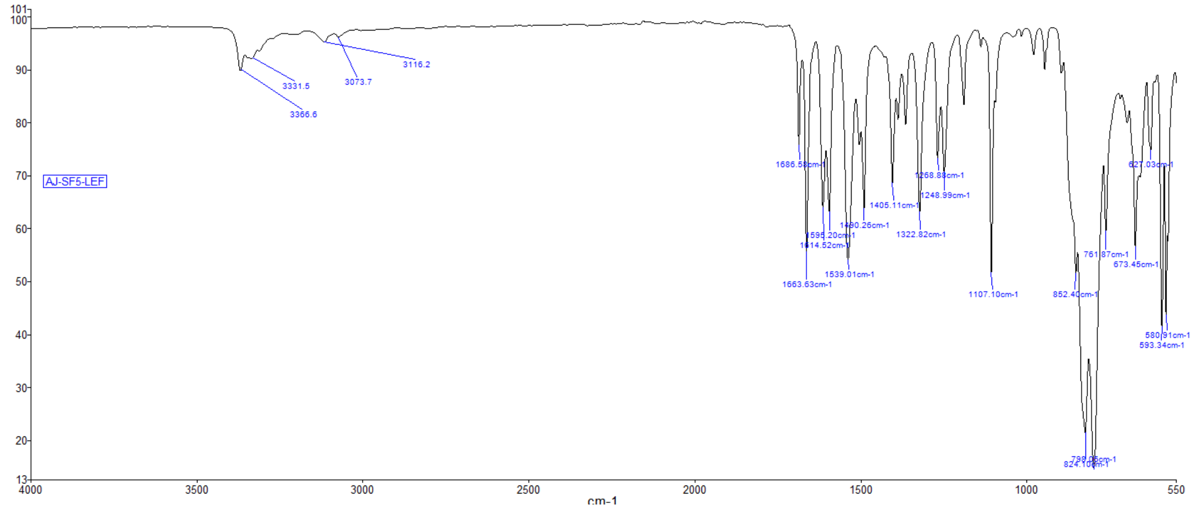
AJ-SF5-LEF
cm-1

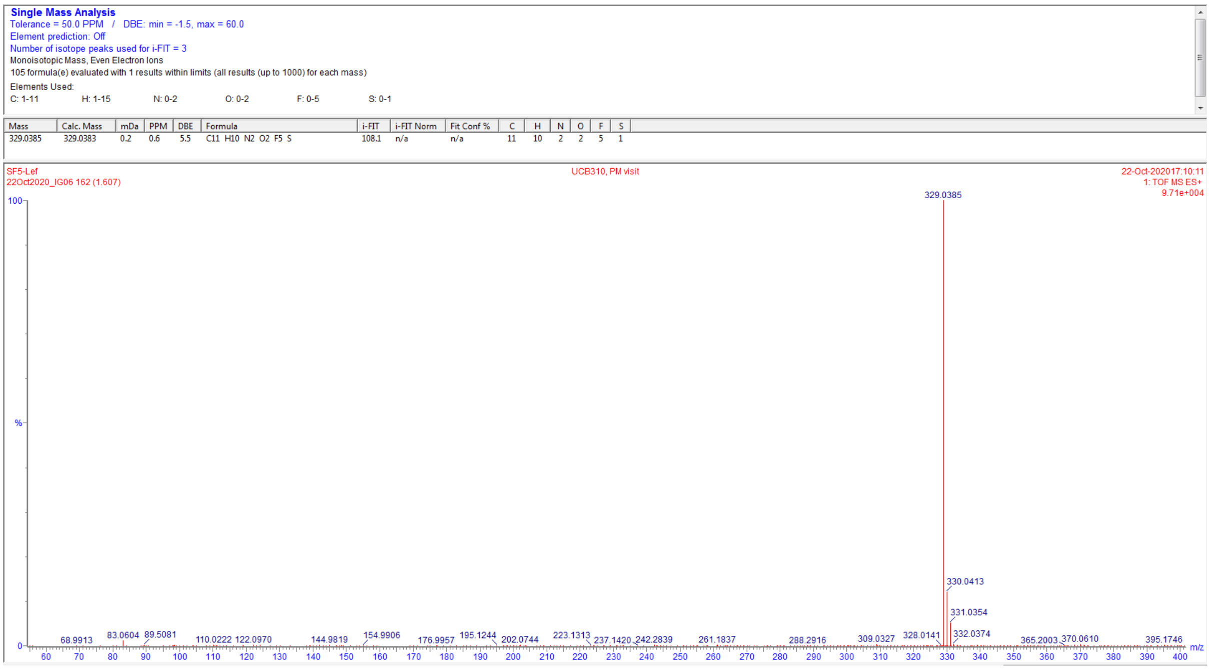

(2Z)-2-Cyano-3-hydroxy-N-[4-(pentafluoro-□[6]-sulfanyl)phenyl]but-2-enamide (18)

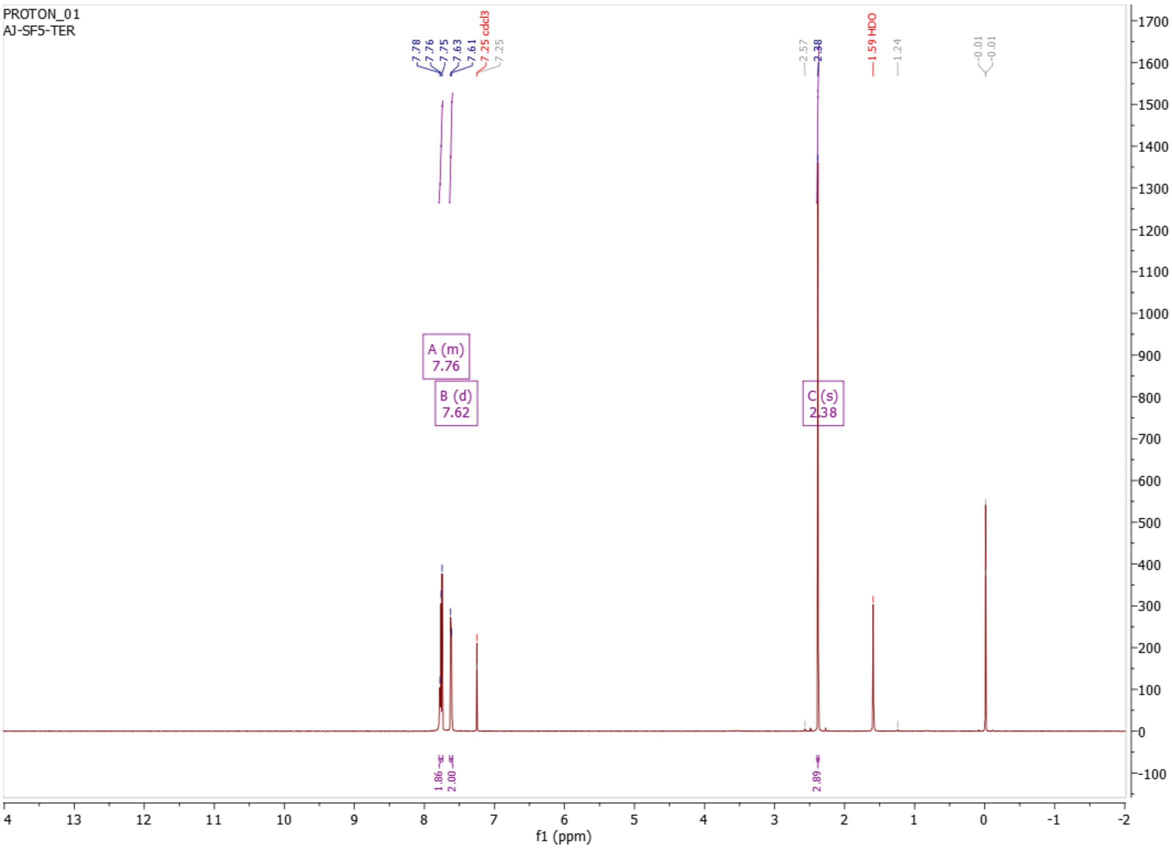

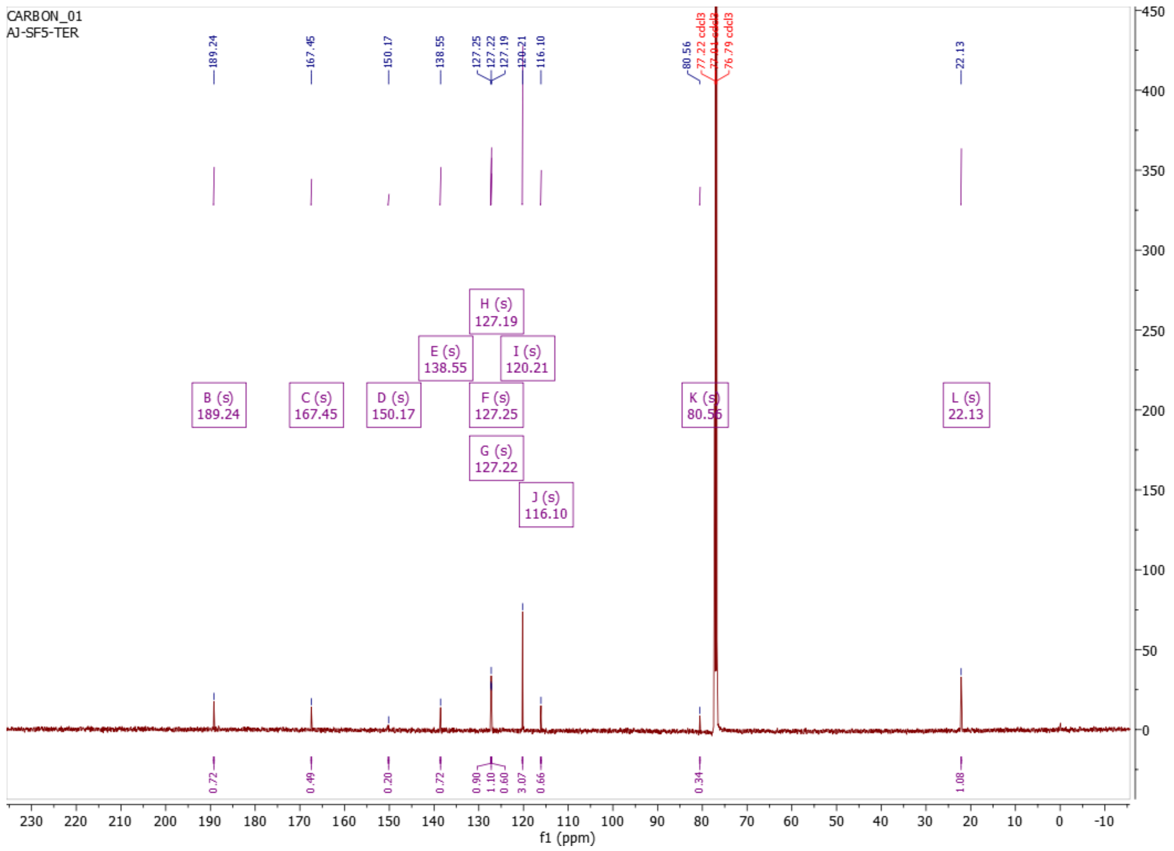
CARBON_01
AJ-SF5-TER
B (s) 189.24
C (s) 167.45
D (s) 150.17
E (s) 138.55
F (s) 127.25
G (s) 127.22
H (s) 127.19
I (s) 120.21
J (s) 116.10
K (s) 80.56
L (s) 22.13
77.22 cdcl3
76.79 cdcl3
f1 (ppm)

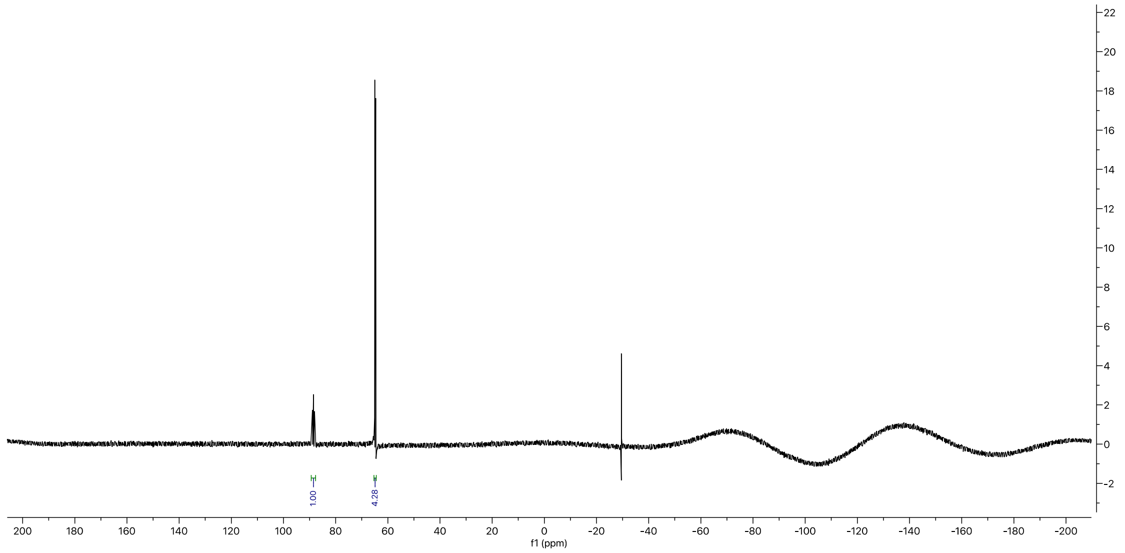
f1 (ppm)

Single Mass Analysis
Tolerance = 3.0 mDa / DBE: min = -1.5, max = 60.0
Element prediction: Off
Number of isotope peaks used for i-FIT = 3
Monoisotopic Mass, Even Electron Ions
110 formula(e) evaluated with 1 results within limits (all results (up to 1000) for each mass)
Elements Used:
C: 1-12 H: 1-30 N: 0-2 O: 0-2 F: 0-5 S: 0-1

Mass	Calc. Mass	mDa	PPM	DBE	Formula	i-FIT	i-FIT Norm	Fit Conf %	C	H	N	O	F	S
327.0247	327.0227	2.0	6.1	6.5	C11 H8 N2 O2 F5 S	823.2	n/a	n/a	11	8	2	2	5	1

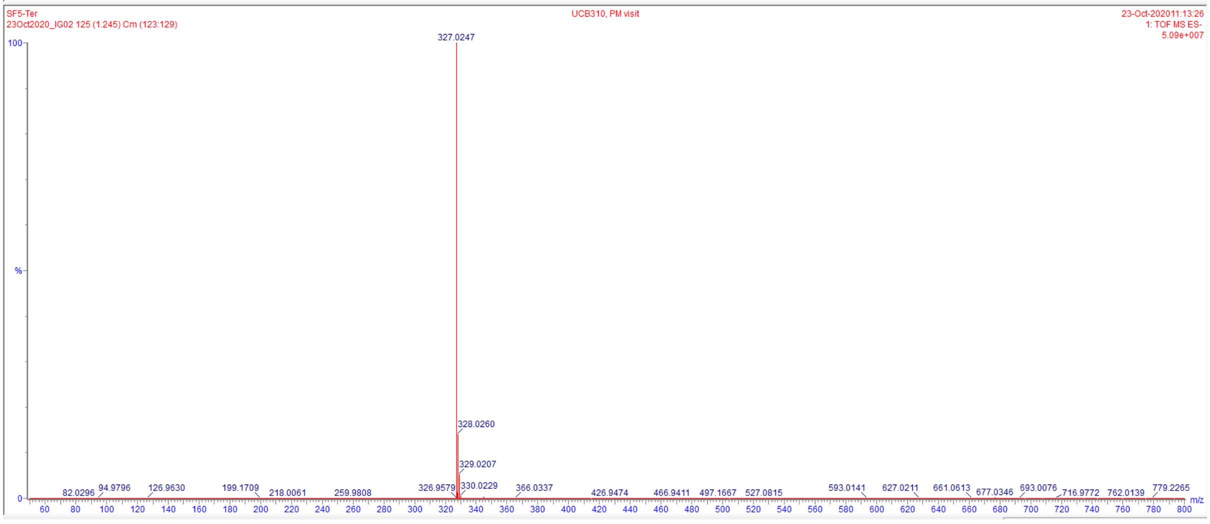

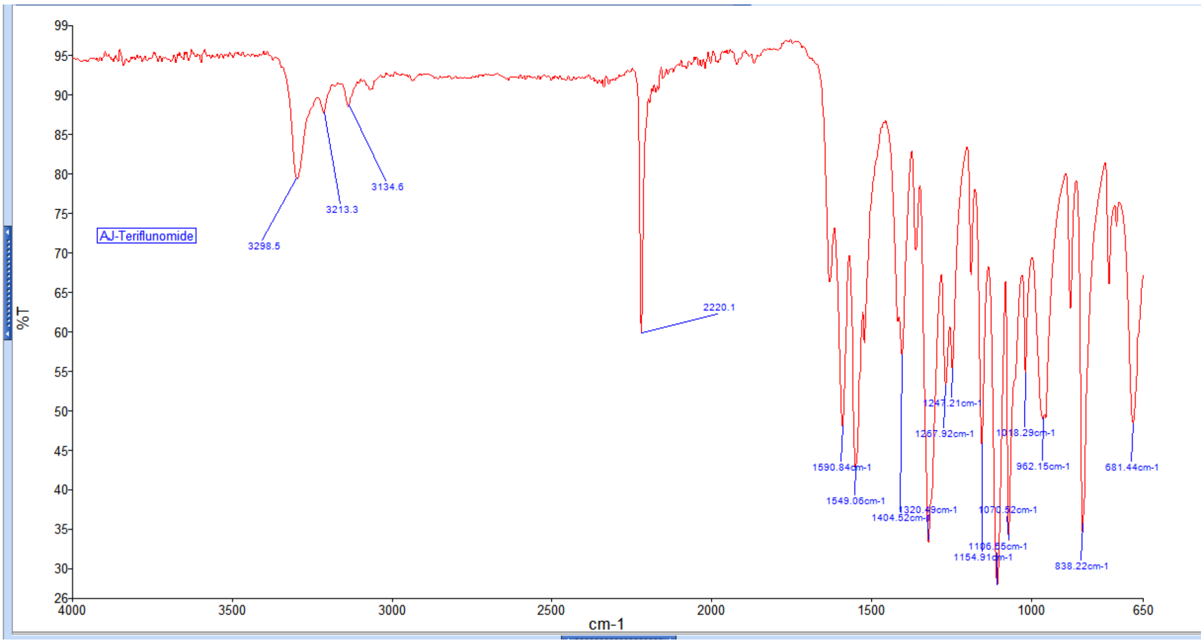

Supplement: Supplementary file 1 — Supporting Information [file CMDC-17-0-s001.pdf]
